# Lung neuroendocrine tumours: deep sequencing of the four World Health Organization histotypes reveals chromatin‐remodelling genes as major players and a prognostic role for TERT, RB1, MEN1 and KMT2D

**DOI:** 10.1002/path.4853

**Published:** 2016-12-29

**Authors:** Michele Simbolo, Andrea Mafficini, Katarzyna O Sikora, Matteo Fassan, Stefano Barbi, Vincenzo Corbo, Luca Mastracci, Borislav Rusev, Federica Grillo, Caterina Vicentini, Roberto Ferrara, Sara Pilotto, Federico Davini, Giuseppe Pelosi, Rita T Lawlor, Marco Chilosi, Giampaolo Tortora, Emilio Bria, Gabriella Fontanini, Marco Volante, Aldo Scarpa

**Affiliations:** ^1^ ARC‐Net Research CentreUniversity and Hospital Trust of VeronaVeronaItaly; ^2^ Department of Diagnostics and Public Health, Section of Anatomical PathologyUniversity and Hospital Trust of VeronaVeronaItaly; ^3^ Department of Surgical and Diagnostic Sciences (DISC)University of Genova and IRCCS S. Martino‐IST University HospitalGenoaItaly; ^4^ Department of Medicine, Section of Medical OncologyUniversity and Hospital Trust of VeronaVeronaItaly; ^5^ Unit of Thoracic SurgeryUniversity and Hospital Trust of PisaPisaItaly; ^6^ Department of Oncology and Haemato‐OncologyUniversità degli Studi di MilanoMilanoItaly; ^7^ Department of Surgical, Medical, Molecular Pathology and Critical AreaUniversity of PisaPisaItaly; ^8^ Department of OncologyUniversity of Turin at San Luigi HospitalOrbassanoTorinoItaly

**Keywords:** lung tumours, neuroendocrine, exome sequencing, targeted sequencing, MEN1, KMT2D, RB1, TERT

## Abstract

Next‐generation sequencing (NGS) was applied to 148 lung neuroendocrine tumours (LNETs) comprising the four World Health Organization classification categories: 53 typical carcinoid (TCs), 35 atypical carcinoid (ACs), 27 large‐cell neuroendocrine carcinomas, and 33 small‐cell lung carcinomas. A discovery screen was conducted on 46 samples by the use of whole‐exome sequencing and high‐coverage targeted sequencing of 418 genes. Eighty‐eight recurrently mutated genes from both the discovery screen and current literature were verified in the 46 cases of the discovery screen, and validated on additional 102 LNETs by targeted NGS; their prevalence was then evaluated on the whole series. Thirteen of these 88 genes were also evaluated for copy number alterations (CNAs). Carcinoids and carcinomas shared most of the altered genes but with different prevalence rates. When mutations and copy number changes were combined, MEN1 alterations were almost exclusive to carcinoids, whereas alterations of TP53 and RB1 cell cycle regulation genes and PI3K/AKT/mTOR pathway genes were significantly enriched in carcinomas. Conversely, mutations in chromatin‐remodelling genes, including those encoding histone modifiers and members of SWI–SNF complexes, were found at similar rates in carcinoids (45.5%) and carcinomas (55.0%), suggesting a major role in LNET pathogenesis. One AC and one TC showed a hypermutated profile associated with a POLQ damaging mutation. There were fewer CNAs in carcinoids than in carcinomas; however ACs showed a hybrid pattern, whereby gains of TERT, SDHA, RICTOR, PIK3CA, MYCL and SRC were found at rates similar to those in carcinomas, whereas the MEN1 loss rate mirrored that of TCs. Multivariate survival analysis revealed RB1 mutation (p = 0.0005) and TERT copy gain (p = 0.016) as independent predictors of poorer prognosis. MEN1 mutation was associated with poor prognosis in AC (p = 0.0045), whereas KMT2D mutation correlated with longer survival in SCLC (p = 0.0022). In conclusion, molecular profiling may complement histology for better diagnostic definition and prognostic stratification of LNETs. © 2016 The Authors. *The Journal of Pathology* published by John Wiley & Sons Ltd on behalf of Pathological Society of Great Britain and Ireland.

## Introduction

Lung neuroendocrine tumours (LNETs) are subclassified into four histological variants: typical carcinoid (TC), atypical carcinoid (AC), large‐cell neuroendocrine carcinoma (LCNEC), and small‐cell lung carcinoma (SCLC) 1. Cytological features, neuroendocrine immunohistochemical markers, the number of mitoses/2 mm^2^ and the presence of necrosis are the main diagnostic criteria 1.

This four‐tier histological spectrum clinically corresponds to a prognostic scheme whereby TCs are low‐grade tumours, with patients having a long life‐expectancy, ACs are intermediate‐grade tumours with varying clinical behaviour, and LCNECs and SCLCs are high‐grade tumours with a dismal prognosis 2, 3, 4, 5, 6. The clinical management of LNETs reflects this prognostic categorization: TC patients are treated with surgery, AC and LCNEC patients are treated with with multimodal approaches involving surgery and/or systemic therapy, and SCLC patients usually undergo systemic therapy 7. The 10‐year overall survival rate of TC patients is reported to be 87%, whereas the median overall survival of patients with untreated SCLC is only 2–3 months, reaching 1 year when they are treated 8.

A better understanding of the genomic changes occurring in LNET is essential to identify new therapeutic targets and markers for better prognostic stratification. Previous molecular studies on LNET 9, 10, 11, 12 have highlighted frequent alterations in the chromatin‐remodelling genes *MEN1*, *PSIP1* and *ARID1A* in carcinoids (ACs and TCs) 9. Conversely, inactivating mutations in *TP53* and *RB1*, amplification of *MYC* family members and genetic alterations in the *PI3K*/*AKT*/*mTOR* pathway have frequently been observed in SCLC 9, 10, 12, 13.

To complement these reports, we performed a direct comparison of the four histotypes by whole‐exome sequencing (WES) and high‐coverage targeted sequencing (HCTS) of 418 genes on a cohort of 46 LNETs, and then validated 88 genes in an independent set of 102 LNETs.

## Materials and methods

### Cases

A cohort of 148 surgically treated LNETs was collected from four Italian institutions (ARC‐Net Research Centre‐Verona; IRCCS San Martino‐Genova; University of Pisa; and AUO Orbassano‐University of Torino), and comprised: 53 TCs, 35 ACs, 27 LCNECs, and 33 SCLCs. The inclusion condition was a concordant diagnosis among five pathologists based on morphological and immunohistochemical criteria of the World Health Organization (WHO) classification 1. No patient received preoperative therapy. Samples had been frozen for 46 cases (23 TCs, 14 ACs, five LCNECs, and four SCLCs), and were formalin‐fixed paraffin‐embedded (FFPE) for 102 cases (28 TCs, 21 ACs, 24 LCNECs, and 29 SCLCs).

### Ethics

Ethics Committee Approval (ECA) was obtained at the four institutions: AUO Orbassano‐University of Torino, ECA no. 161/2012‐prot.17751 (2 October 2012); ARC‐Net Research Centre‐Verona, ECA no. 2173‐prot.26775 (1 June 2012); IRCCS San Martino‐Genova, ECA no. 027/2016LM (16 March 2016); University of Pisa, ECA no. 1040/16 (31 March 2016).

### Discovery screen on 46 LNETs by WES and HCTS of 418 genes

Matched tumour/normal DNA available in high quantity from 20 frozen LNETs (10 TCs; four ACs; three LCNECs; and three SCLCs) was subjected to WES with a HiSeq2000 sequencer (Illumina, Milan, Italy). The same 20 cases and an additional 26 frozen LNETs (13 TCs; 10 ACs; two LCNECs; and one SCLC) with DNA available in low quantity were subjected to HCTS of 418 genes with a customized version of the Ion AmpliSeq Comprehensive Cancer Panel (ThermoFisher, Milan, Italy). Mutational and copy number alteration (CNA) analysis was performed as described in supplementary material, Supplementary materials and methods, and citations therein 9, 10, 12, 13, 14, 15, 16, 17, 18, 19, 20.

### Validation of mutations and CNAs in 102 LNETs by targeted sequencing of 88 genes

Matched tumour/normal DNA from 102 FFPE LNETs was subjected to targeted next‐generation sequencing (NGS) to validate the prevalence of somatic alterations in 88 genes, selected as follows: 42 from the discovery screen, and 46 from current LNET literature 9, 10, 12, 13. To this end, we used four multigene panels (supplementary material, Supplementary materials and methods and Table S1).

### Statistical analysis

One‐way anova, the Kruskal–Wallis test, Fisher's test with Monte Carlo simulation and Fisher's exact test were used as appropriate; multiple comparisons were corrected according to Benjamini–Hochberg. For comparison of Kaplan–Meier survival curves, the Mantel–Cox test was used. For multivariate survival analysis, a stepwise Cox regression with forward algorithm was used. A *p*‐value of <0.05 was considered to be significant. Analyses were performed with Medcalc for Windows v.15.6 (MedCalc Software, Ostend, Belgium) and R v.3.2.1 with survival library v.2.38‐2.

## Results

**Figure 1:**
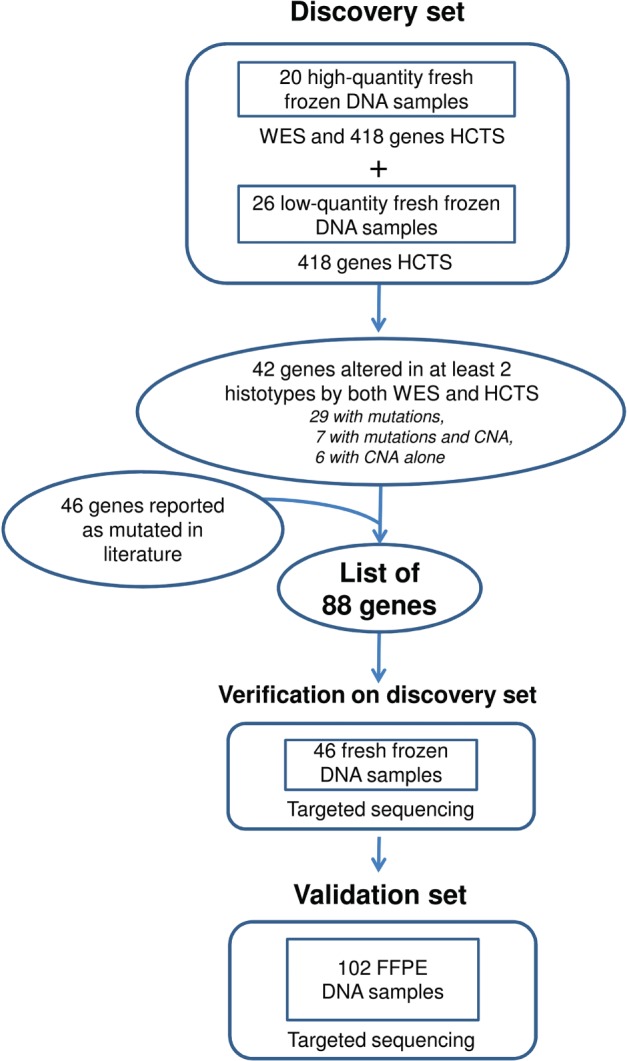
Flow chart of the experiments performed on 148 LNETs. The chart shows the workflow of sequencing analysis, including discovery screen on 46 fresh‐frozen cases and validation on 102 FFPE cases.

**Figure 2:**
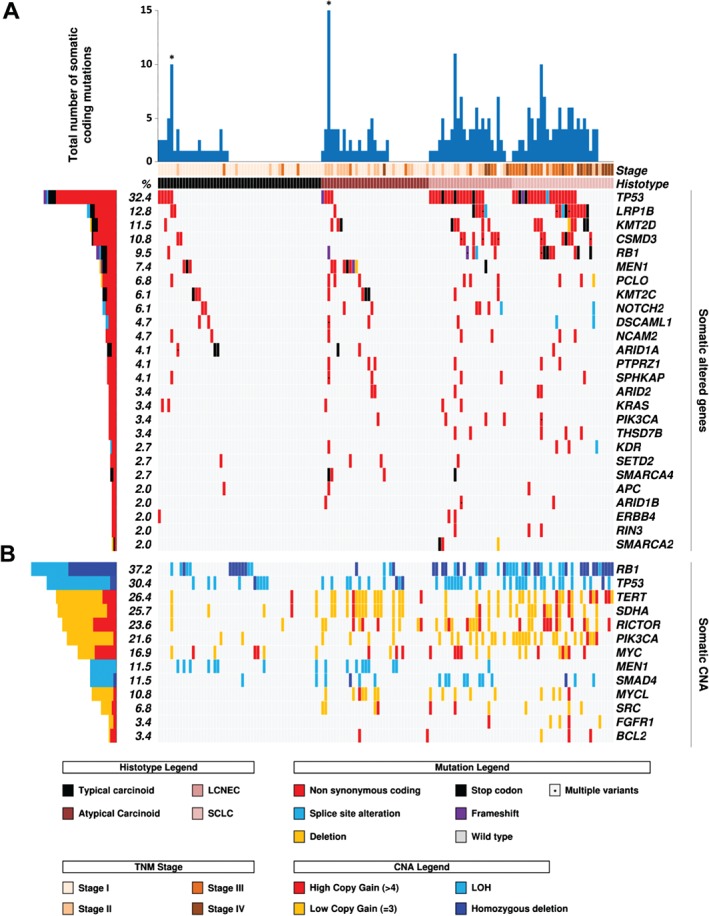
Recurrent alterations in 148 LNETs. Cases are grouped according to the four histotypes defined by the WHO classification. The histograms on the left report the alteration frequency of each gene expressed as a percentage. Alterations are annotated by different colours, according to their impact on the gene product. (A) The upper histogram shows the number of mutations in recurrently altered genes for each sample. Asterisks indicate the two hypermutated cases that emerged from WES. The matrix shows 26 genes that were mutated in at least three cases. (B) The matrix shows the copy number status of 13 genes in the whole cohort.

The study workflow is illustrated in Figure 1. The clinicopathological characteristics of the 148 LNETs are summarized in Table 1 and detailed in supplementary material, Table S2, with indications of the molecular analyses performed. A discovery screen for mutations and CNAs was performed on DNA from 46 frozen samples, with WES on 20 cases (high‐quantity DNA samples) and HCTS of 418 genes on these and an additional 26 (low‐quantity DNA samples). An 88‐gene panel was then selected as follows: 42 genes from this discovery screen, including 13 that showed CNAs, i.e. *BCL2*, *FGFR1*, *MEN1*, *MYC*, *MYCL*, *PIK3CA*, *RB1*, *RICTOR*, *SDHA*, *SMAD4*, *SRC*, *TERT*, and *TP53* (panel 3 of supplementary material, Table S1), and 46 from a literature review 9, 10, 12, 13. These 88 genes were verified by targeted NGS in the discovery set, and then assessed on a validation set of an additional 102 FFPE LNETs. The prevalence of mutations and CNAs was computed on the whole 148 cases cohort. The results of the mutational analysis of 88 genes are detailed in supplementary material, Table S3, and summarized in Figure 2A for the 26 genes mutated in at least three cases. Eleven of these 26 genes met the criteria for statistical analysis of prevalence among LNET histotypes (genes altered in at least 10% of at least one histotype), with seven genes showing a statistically significant difference (Table 2). The results of CNA analysis of 13 genes are shown in Figure 2B and reported in Table 3.

**Table 1:** Comparison of clinicopathological features of 148 lung neuroendocrine tumours across the four histotypes defined by the World Health Organization classification

**Clinicopathological features**	**TC (n = 53)**	**AC (n = 35)**	**LCNEC (n = 27)**	**SCLC (n = 33)**	**p‐Value** *
Mean age (years) ± SD	54.1 ± 15.3	61.2 ± 15.1	70.4 ± 7.2	66.9 ± 9.4	< 0.0001†
Females/males (no.)	33/20	20/15	5/22	10/23	0.0002
Smokers/non‐smokers (no.)	17/13	15/11	16/6	26/1	0.0013
Missing value (no.)	23	9	5	6	
Median tumour size (cm)	2.5	2.8	3.3	2.8	0.10‡
1st–3rd quartile	1.7–3.2	1.6–4.4	2.3–3.8	2.4–3.5	
Missing value (no.)	0	0	1	14	
Mitotic count (mean)	0.6	3.5	36.0	36.7	< 0.0001†
Range	0–1	1–7	11–79	18–130	
Ki67 index (mean)	3.7	12.5	71.2	65.3	< 0.0001†
Range	1–15	2–45	20–95	40–95	
pT (no.)					< 0.0001
T1	35	16	8	8	
T2	15	14	11	10	
T3	2	5	7	3	
T4	1	0	1	12	
pN (no.)					< 0.0001
N0	48	24	18	4	
N1	4	8	3	9	
N2	1	3	5	19	
N3	0	0	1	1	
pM (no.)					< 0.0001
M0	53	34	24	22	
M1	0	1	3	11	
Tumour stage (no.)					< 0.0001
I	46	17	11	2	
II	4	15	8	4	
III	3	2	5	16	
IV	0	1	3	11	

AC, atypical carcinoid; LCNEC, large‐cell neuroendocrine carcinoma; SCLC, small‐cell lung cancer; SD, standard deviation; TC, typical carcinoid.

* Fisher's exact test was used for categorical variables. Fisher's test with Monte Carlo simulation was used for pT, pN, pM, and tumour stage. The Shapiro–Wilk test was used to test the Gaussian distribution of continuous variables.

† The Kruskal–Wallis test was used for age.

‡ One‐way anova on log‐transformed data for tumour size.

**Table 2:** Differences in mutation prevalence for 11 recurrently mutated genes among the four histotypes of 148 lung neuroendocrine tumour (LNET) (related to Figure 2A)

**Gene** *	**TC (*n* = 53), no. (%)**	**AC (*n* = 35), no. (%)**	**LCNEC (*n* = 27), no. (%)**	**SCLC (*n* = 33), no. (%)**	***p*‐Value** †	**Adjusted** ***p*‐value** ‡
*TP53*	5 (9)	4 (11)	18 (67)	21 (64)	4.5E‐11	5.4E‐10
*LRP1B*	2 (4)	1 (3)	5 (19)	11 (33)	0.00015	0.00088
*KMT2D*	1 (2)	3 (9)	5 (19)	8 (24)	0.0039	0.0094
*CSMD3*	2 (4)	0 (0)	7 (26)	7 (21)	0.00027	0.0011
*RB1*	1 (2)	1 (3)	4 (15)	8 (24)	0.0015	0.0047
*MEN1*	3 (6)	7 (20)	1 (4)	0 (0)	0.013	0.022
*PCLO*	1 (2)	3 (9)	3 (11)	3 (9)	0.28	0.28
*KMT2C*	3 (6)	4 (11)	2 (7)	0 (0)	0.23	0.27
*NOTCH2*	2 (4)	2 (6)	4 (15)	1 (3)	0.27	0.28
*PIK3CA*	0 (0)	1 (3)	3 (11)	1 (3)	0.055	0.073
*SMARCA2*	0 (0)	0 (0)	3 (11)	0 (0)	0.0055	0.011

AC, atypical carcinoid; LCNEC, large‐cell neuroendocrine carcinoma; SCLC, small‐cell lung cancer; TC, typical carcinoid.

* Eighty‐eight genes were analysed; 56 of them were found to be mutated in at least one case, and are reported in supplementary material, Table 3. Genes met the criteria for prevalence statistical analysis when mutated in at least 10% of an individual LNET histotype.

† Fisher's exact test.

‡ Correction for multiple comparisons according to Benjamini–Hochberg.

**Table 3:** Copy number alterations detected in 148 lung neuroendocrine tumours by targeted sequencing of 13 genes according to histotype (related to Figure 2B)

**Gene**	**TC (*n* = 53), no. (%)**			**AC (*n* = 35), no. (%)**			**LCNEC (*n* = 27), no. (%)**			**SCLC (*n* = 33), no. (%)**				
	**HD**	**LOH**	**G**	**HD**	**LOH**	**G**	**HD**	**LOH**	**G**	**HD**	**LOH**	**G**	***p*‐Value** *	**Adjusted** † ***p*‐value**
*RB1*	7 (13.2)	6 (11.3)		2 (5.7)	2 (5.7)		9 (33.3)	6 (22.2)		13 (39.4)	10 (30.3)		2.7E‐07	1.9E‐06
*TP53*	2 (3.8)	8 (15.1)		2 (5.7)	8 (22.9)			11 (40.7)			14 (42.4)		0.078	0.084
*TERT*			3 (5.6)			15 (42.9)			5 (18.5)			17 (51.5)	1.3E‐06	5.6E‐06
*SDHA*			5 (9.4)			14 (40.0)			6 (22.2)			13 (39.4)	0.0015	0.0032
*RICTOR*			2 (3.8)			10 (28.6)			10 (37.0)			13 (39.4)	3.7E‐05	0.00012
*PIK3CA*			2 (3.8)			4 (11.4)			9 (33.3)			17 (51.5)	2.9E‐07	1.9E‐06
*MYC*			7 (13.2)			4 (11.4)			6 (22.2)			8 (24.2)	0.37	0.37
*MEN1*		8 (15.1)			8 (22.9)			1 (3.7)					0.0061	0.011
*SMAD4*		2 (3.8)		1 (2.9)	3 (8.6)			8 (29.6)		1 (3.0)	2 (6.1)		0.0079	0.011
*MYCL*						7 (20.0)			5 (18.5)			4 (12.1)	0.0013	0.0032
*SRC*						4 (11.4)			3 (11.1)			3 (9.1)	0.030	0.039
*FGFR1*									1 (3.7)			4 (12.1)	0.0070	0.011
*BCL2*						2 (5.7)						3 (9.1)	0.049	0.058

AC, atypical carcinoid; G, gain; HD, homozygous deletion; LCNEC, large‐cell neuroendocrine carcinoma; LOH, loss of heterozygosity; SCLC, small‐cell lung cancer; TC, typical carcinoid.

* Comparison of copy number alteration frequency among histotype by Fisher's exact test.

† Correction for multiple comparisons according to Benjamini–Hochberg.

### Discovery screen by WES and HCTS of 418 genes

#### Mutational analysis

The 46‐case discovery set was composed of two groups based on the amount of available DNA: 20 high‐quantity DNA samples (10 TCs; four ACs; three LCNECs; and three SCLCs) and 26 low‐quantity DNA samples (13 TCs; 10 ACs; two LCNECs; and one SCLC). Each group was analysed according to DNA availability: WES and HCTS for high‐quantity DNA samples, and HCTS for low‐quantity ones.

WES achieved an average coverage of × 94 (×75–161) in tumour samples and × 72 (×38–161) in normal samples. One thousand three hundred and twenty‐two non‐synonymous somatic mutations were found, including 1184 missense and 88 nonsense mutations, 34 frameshifts, six in‐frame insertions, and 10 in‐frame deletions (supplementary material, Figure S1A and Table S4A). The mean numbers of mutations per sample were 14.6 for TCs, 15.7 for ACs, 64.7 for LCNECs, and 90.7 for SCLCs. One TC (no. 304) and one AC (no. 389) emerged among carcinoids as having a hypermutated profile, as they respectively contained 228 and 434 mutations, i.e. a gross excess of point mutations relative to the same tumour type (supplementary material, Figure S1A). These cases shared inactivating somatic mutations of *POLQ*, encoding polymerase θ (supplementary material, Table S4A), and hypermutated profiles have been associated with mutations of genes encoding polymerases 21, 22.

HCTS of 418 genes on the 46 cases of the discovery set yielded an average coverage of × 864 (×298–1657) in tumour samples and × 686 (×670–1316) in normal samples. Mutations were identified in 36 genes, and were 145 missense mutations, 13 nonsense mutations, two frameshifts, five splice site alterations, and one in‐frame deletion (supplementary material, Table S4B and Figure S1B). The mean numbers of mutations per sample were 0.7 for TCs, 1.8 for ACs, 4.6 for LCNECs, and 5.8 for SCLCs. One hundred and sixteen of the 1322 somatic mutations identified by WES were targetable by HCTS, and all of them were confirmed.

In conclusion, WES and HCTS sequencing on the discovery set of 46 cases identified 36 genes as recurrently mutated (i.e. in at least two cases of different histotypes on both WES and HCTS) (Figure 1; supplementary material, Table S4C).

#### Copy number alterations

CNA analysis of the discovery set was performed with data from both WES on the 20 cases with high DNA quantity and HCTS of 418 genes on the whole discovery set.

**Figure 3:**
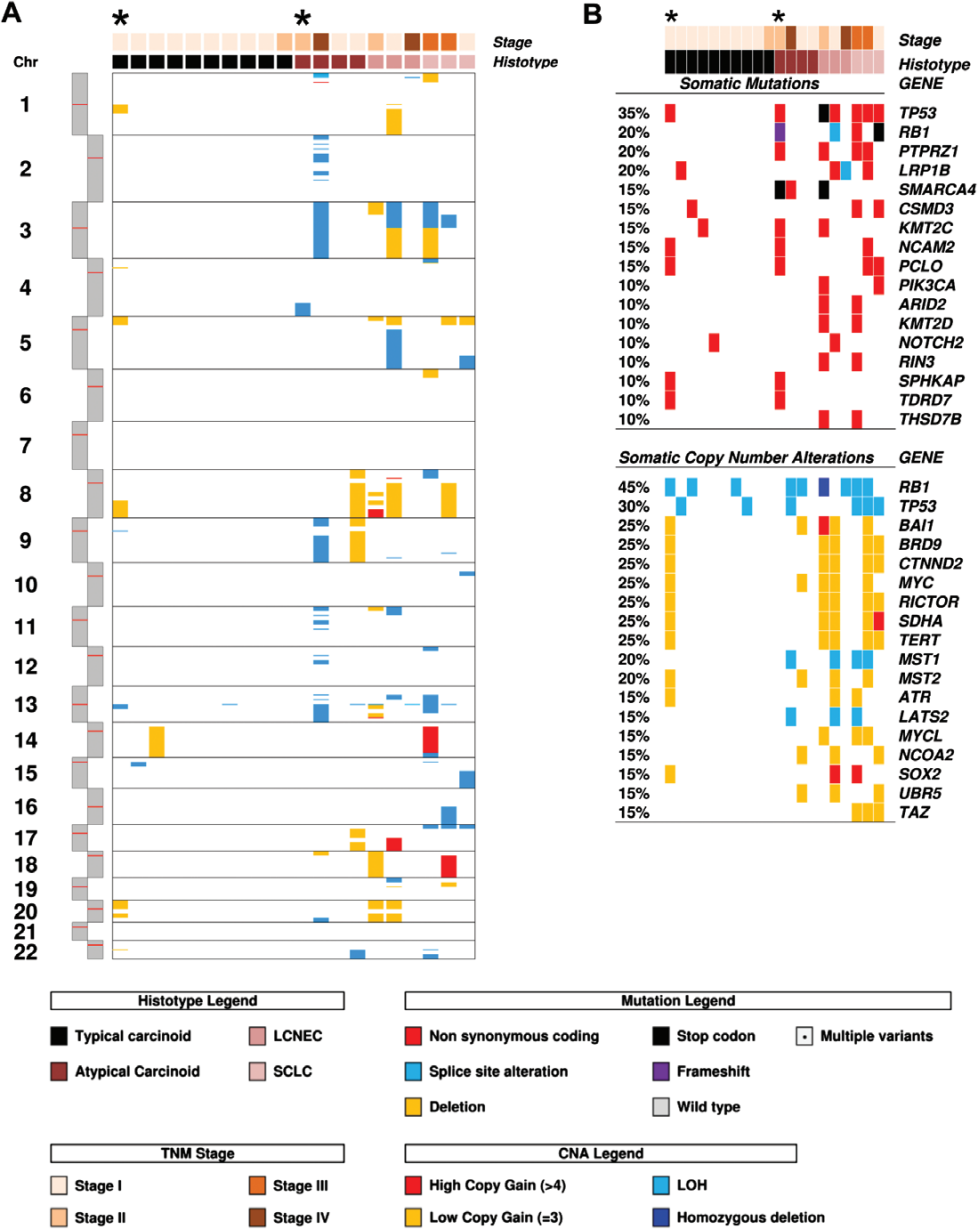
Somatic copy number alterations detected in 20 LNETs by WES. Cases are grouped according to the four histotypes defined by the WHO classification, and arranged from the most to the least altered within each category. Asterisks indicate the two hypermutated cases that emerged from WES. (A) Copy gain (red/yellow) or loss (cyan/blue) of large chromosomal regions. (B) Overview of genes affected by either somatic mutation or copy number alteration. For each case, the upper panel shows somatic mutations, and the lower panel shows copy number alterations, both annotated according to the colour panels at the bottom.

Large structural CNA analysis, which was feasible only for WES cases, showed frequent gains of chromosomes 5p (7/20; 35.0%) and 8 (5/20; 25.0%), and loss of heterozygosity (LOH) of chromosome 3 (5/20; 25.0%) (Figure 3A). Carcinoids showed rare CNAs. However, three cases showed an unusually unstable genomic profile: AC no. 450 and the hypermutated TC no. 304 showed alterations in several chromosomes (Figure 3A), whereas AC no. 034 showed a copy number pattern compatible with chromothripsis of chromosomes 2, 11, and 20 (supplementary material, Figure S2). Six of the eight classification criteria used to define chromothripsis 23 are assessable by WES: five were fulfilled for chromosomes 2 and 11, and four for chromosome 20. Conversely, carcinomas had many large structural CNAs, including copy gains at chromosomes 5p and 8, and LOH at chromosome 13 and 17p, where *RB1* and *TP53*, respectively, are located.

Gene‐level CNAs were investigated on the discovery set with both WES data (Figure 3B; supplementary material, Table S5A) and HCTS data (supplementary material, Figure S1B and Table S5B). The most recurrent copy loss events involved one or both copies of *RB1* (TCs, 13.0%; ACs, 14.3%; LCNECs, 60.0%; SCLCs, 50.0%) and *TP53* (TCs, 8.7%; ACs, 7.1%; LCNECs, 20.0%; SCLCs, 75.0%). Conversely, frequent concurrent gains were observed for *BRD9*, *CTNND2*, and *TERT* (TCs, 8.7%; ACs, 14.3%; LCNECs, 40.0%; SCLCs, 75.0%). Additional frequent gains included *MYC* (TCs, 4.3%; ACs, 7.1%; LCNECs, 80.0%; SCLCs, 50.0%), *SDHA* (TCs, 4.3%; ACs, 7.1%; LCNECs, 60.0%; SCLCs, 75.0%) and *RICTOR* (TCs, 8.7%; ACs, 14.3%; LCNECs, 40.0%; SCLCs, 50.0%).

Although a specific CNA hallmark of each histological subtype was not apparent, the average number of genes affected by CNA progressively increased from TCs to SCLCs (TCs, 1.3; ACs, 2.0; LCNECs, 10.8; SCLCs, 14.7; *p* < 0.0001), with ACs showing a hybrid profile between TCs and carcinomas.

### Prevalence of mutations in 148 LNETs

To validate mutations, verification was performed on the 46 discovery set cases and an additional validation set of 102 FFPE samples (29 TCs; 21 ACs; 24 LCNECs; and 28 SCLCs) with four NGS multigene panels (supplementary material, Table S1) designed to target 88 genes, including 42 identified by our discovery screen and 46 recurrently altered according to the LNET literature 9, 10, 12, 13 (Figure 1). Prevalence was then computed on the entire cohort of 148 LNETs. The results of mutation analysis are summarized in Table 2 and Figure 2, and detailed in supplementary material, Table S3. Overall, carcinomas had more mutations than carcinoids. The mean number of mutations among the four LNET subtypes was as follows: TCs, 0.8; ACs, 1.6; LCNECs, 3.0; and SCLCs, 2.9. At the single‐gene level, the most frequently altered genes were *MEN1* in carcinoids (10/88; 11.4%), particularly in ACs (20%; *p* = 0.022), and *TP53* in carcinomas (TCs, 9.4%; ACs, 11.4%; LCNECs, 67.0%; SCLCs, 63.6%; *p* = 5.4E‐10). A similar distribution was observed for *KMT2D* (TCs, 1.9%; ACs, 8.6%; LCNECs, 18.5%; SCLCs, 24.2%; *p* = 0.0094). It is of note that mutations in *SMARCA2* were exclusive to LCNECs (11.1%; *p* = 0.011). As previously reported 13, 24, we identified sporadic *KRAS* mutations in both carcinoids and carcinomas. but these tumours were not associated with clinicopathological features of aggressiveness.

**Figure 4:**
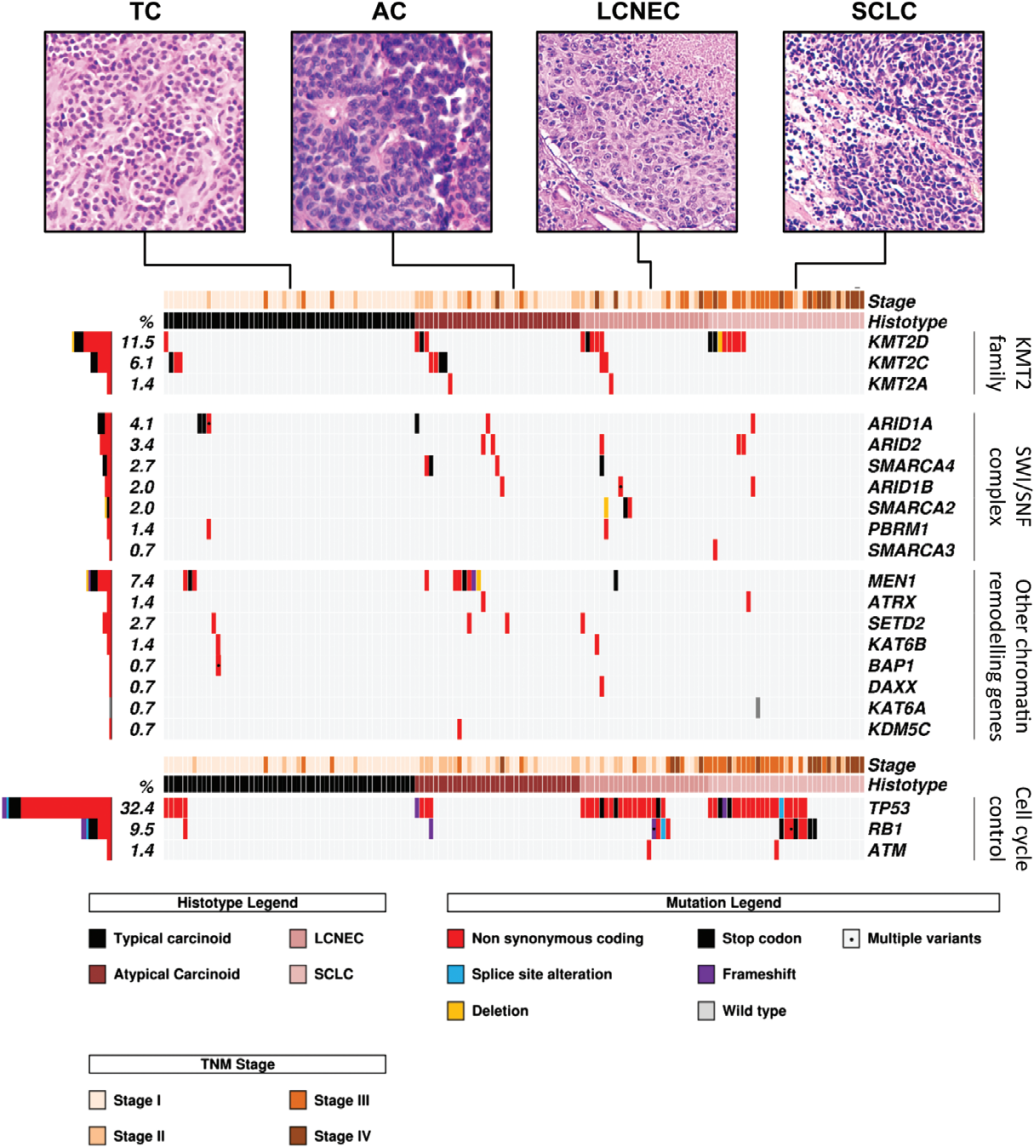
Somatic mutations in chromatin‐remodelling and cell cycle checkpoint genes for 148 LNETs. Cases are grouped according to the four histotypes defined by the WHO classification. Micrographs illustrate a representative case for each histotype. Genes are grouped as per molecular pathway. The histograms on the left show the mutation frequency of each gene expressed as a percentage. Mutations are annotated according to the colour panels at the bottom.

Chromatin‐remodelling genes were mutated in carcinoids (40/88; 45.5%) and carcinomas (33/60; 55.0%) at similar rates (*p* = 0.32, computed from supplementary material, Table S3; Figure 4). Specifically, the *KMT2* family of covalent histone modifiers (*KMT2A*, *KMT2C*, and *KMT2D*) was mutated in 12 of 88 (13.6%) carcinoids versus 16 of 60 (26.7%) carcinomas (*p* = 0.11), and the *ARID* family, involved in the SWI–SNF complex (*ARID1A*, *ARID1B*, and *ARID2*), had mutations in eight of 88 (9.0%) carcinoids versus six of 60 (10.0%) carcinomas (*p* = 1.0). In contrast, the *TP53*, *RB1* and *ATM* cell cycle checkpoint genes showed a significantly higher rate of mutations in carcinomas (42/60; 70.0%) than in carcinoids (9/88; 10.2%; *p* < 0.0001; Figure 4). Similarly, components of the *PI3K*/*AKT*/*mTOR* pathway showed a mutation rate higher in carcinomas than in carcinoids (11.7% versus 2.3%; *p* = 0.031).

### Prevalence of CNAs in 148 LNET

Recurrent CNAs detected in the discovery screen were assessed in the 102 cases of the validation set by targeted sequencing of 13 genes (panel 3, supplementary material, Table S1): *BCL2*, *FGFR1*, *MEN1*, *MYC*, *MYCL*, *PIK3CA*, *RB1*, *RICTOR*, *SDHA*, *SMAD4*, *SRC*, *TERT*, and *TP53*. The prevalence of CNAs was then computed on the entire cohort of 148 LNETs, and is illustrated in Figure 2B and detailed in Table 3.

*RB1* was the gene most frequently affected by losses across all samples, with a different distribution between subtypes (TCs, 24.5%; ACs, 11.4%; LCNECs, 55.6%; SCLCs, 69.7%; *p* = 1.9E‐06), and the highest frequency in SCLCs was mainly attributable to homozygous deletions (13/33, 39.4%). When homozygous deletions were combined with deleterious mutations, the prevalence of *RB1* alterations in SCLCs was 64% (21/33), and when 10 cases with LOH were added, the prevalence was 91% (30/33). *TP53* was the second most frequent gene showing loss of one or both copies (TCs, 18.9%; ACs, 28.6%; LCNECs, 40.7%; SCLCs, 48.5%; *p* = 0.084). LOH in *MEN1* was significantly more frequent in carcinoids than in carcinomas (TCs, 15.1%; ACs, 22.9%; LCNECs, 3.7%; SCLCs, 0%; *p* = 0.011).

*TERT*, *SDHA* and *RICTOR* had the most frequent copy number gains (26.4%, 25.7% and 23.6% of all cases, respectively), with significant enrichment in carcinomas (Table 3). Gains of *PIK3CA* were also frequent (21.6% of all cases), and were distributed differently among subtypes (*p* = 1.9E‐06) (Table 3). Gains involving *MYC* were sporadically distributed among subtypes.

### Survival analysis

Follow‐up was available for 117 cases (TCs, 39; ACs, 28; LCNECs, 22; SCLCs, 28). The median survival time was 74 months, and 39 (33%) subjects died of disease (TCs, 0; ACs, 6; LNECs, 9; SCLCs, 24).

**Figure 5:**
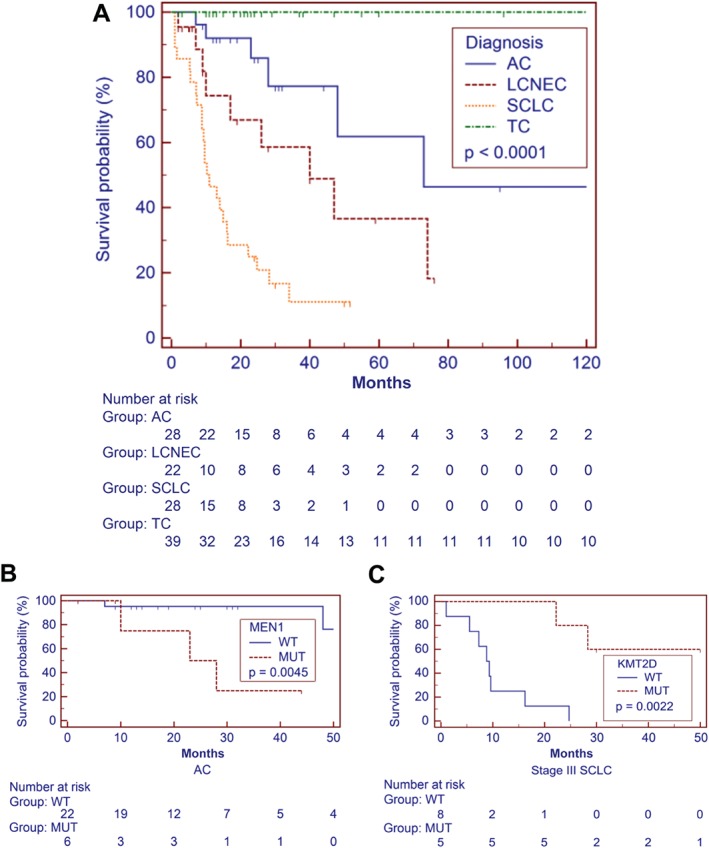
Disease‐specific survival according to pathological and molecular features. (A) Disease‐specific survival of patients with LNET (n = 117) is significantly affected by histology (p < 0.0001). (B, C) The presence of MEN1 mutation in ACs correlates with poor prognosis (p = 0.0045) (B), whereas KMT2D mutation is linked to better prognosis in patients with SCLC (p = 0.0022) (C). Survival time is expressed in months. Kaplan–Meier and log‐rank statistics were used to determine levels of significance. x‐Axes are trimmed for visualization purposes.

On univariate analysis, significant clinicopathological predictors of poorer outcome among the whole cohort were tumour histotype (*p* < 0.0001; Figure 5), tumour stage [stage I hazard ratio (HR) = 1; stage II HR = 2.8; stage III HR = 12.5; stage IV HR = 19.4; *p* < 0.0001], age over 60 years (HR = 5.6; *p* < 0.0001), and male sex (HR = 3.5; *p* = 0.0004). Multivariate analysis confirmed the follwing as main predictors of unfavourable prognosis: histotype (LCNEC HR = 4.7, *p* = 0.0057; SCLC HR = 12.3, *p* < 0.0001) and tumour stage III (HR = 2.7; *p* = 0.041) and IV (HR = 6.1; *p* = 0.0004).

To identify prognostic molecular markers in all LNETs, we screened the following common alterations among the three subtypes showing disease‐specific mortality (ACs, LCNECs, and SCLCs): *KMT2D* mutation, *MYC* copy gain, *PIK3CA* copy gain, *RB1* mutation, *RB1* homozygous deletion, *RICTOR* copy gain, *TERT* copy gain, *TP53* mutation, and *TP53* LOH. TC cases were excluded from this analysis, given that their features (i.e. no death of disease after long follow‐up) would have represented a confounding effect on analysis of the impact of molecular markers on the rest of the series. Each marker yielding a *p*‐value of <0.2 on univariate survival analysis (supplementary material, Table S6 and Figure S3) was tested in a multivariate model against the significant clinicopathological predictors of tumour stage and histotype. The final analysis confirmed *RB1* mutation, *TERT* copy gain, histotype and tumour stage as independent factors for poor prognosis (Table 4).

**Table 4:** Multivariate analysis on 117 lung neuroendocrine tumours showed mutation in RB1 and gain in TERT as independent prognostic markers of poor prognosis

**Covariate**	**Univariate hazard ratio**	**95% CI**	**Multivariate hazard ratio**	**95% CI**	***p*‐Value** *
Histotype = LCNEC	2.45	0.91–6.61	3.84	1.28–11.52	0.016
Histotype = SCLC	6.53	2.45–17.40	5.41	1.55–18.93	0.0082
Stage = III	12.47	4.88–31.88	3.90	1.40–10.87	0.0091
Stage = IV	19.45	5.60–67.49	9.14	3.18–26.29	<0.0001
*RB1*: mutation†	3.29	0.96–11.30	5.76	2.14–15.50	0.0005
*TERT*: gain†	1.55	0.80–3.02	2.55	1.19–5.44	0.016

CI, confidence interval; LCNEC, large‐cell neuroendocrine carcinoma; SCLC, small‐cell lung cancer.

* Cox proportional‐hazards regression analysis. Selection of the best model was performed with the ‘forward’ algorithm.

† Univariate analysis of *RB1* mutation and *TERT* copy gain includes only atypical carcinoids, LCNECs, and SCLCs. Typical carcinoid cases were excluded from univariate analysis of molecular alterations, given that their features (i.e. no death from disease after long follow‐up) would have represented a confounding effect on analysis of the impact of molecular markers on the rest of the series.

We also performed a stratified univariate analysis for mutations clustering to specific tumour histotypes, which showed a significant association of *MEN1* mutation with poor prognosis in ACs (*p* = 0.0045), in which *MEN1* mutations were mainly confined to early‐stage tumours. Among SCLCs, *KMT2D* mutation was almost exclusive to stage III tumours [five mutated of 13 stage III (38.5%) versus one mutated of 11 stage IV (9%)], and correlated with longer survival (*p* = 0.0022).

## Discussion

Although the WHO classification defines clinically relevant subgroups of LNET, there is still a need for better diagnostic definition and prognostic stratification within subtypes. Therefore, we performed a direct comparison of molecular features of the four LNET subtypes, which to date has been lacking, on a cohort of 148 cases classified by five independent pathologists according to WHO criteria 1, including morphology, appropriate immunohistochemistry, and proliferation rate.

The results of our study can be summarized as follows: (1) carcinoids and carcinomas shared most of the altered genes, but with different prevalence rates among subtypes; (2) when mutations and copy number changes were combined, *MEN1* alterations were almost exclusive to carcinoids, whereas alterations of the *TP53* and *RB1* cell cycle regulation genes and *PI3K*/*AKT*/*mTOR* pathway genes were significantly enriched in carcinomas; (3) chromatin‐remodelling genes showed comparably high mutation rates in carcinoids and carcinomas; (4) rare carcinoids showed a hypermutated phenotype, which was associated with damaging *POLQ* mutations; (5) the prevalence of CNA was lower in carcinoids than in carcinomas – however, ACs showed an intermediate profile, with gains of several genes at rates similar to those in carcinomas, and loss of *MEN1* at a frequency comparable to that in TCs; and (6) *RB1* mutation and *TERT* gain were independent unfavourable prognostic markers in all LNET series, whereas *MEN1* mutation was associated with poor prognosis in ACs, and *KMT2D* mutation was associated with longer survival in SCLCs.

No genetic alteration was exclusive to a specific LNET subtype, but the differences between carcinoids and carcinomas reside in the prevalence rates of the most frequently mutated genes, with the exception of *SMARCA2*, which was altered in LCNECs only (*p* = 0.011). Overall, this is in line with previous reports, and further demonstrates that the phenotypic classification of LNETs into carcinoids (ACs and TCs) and carcinomas (LCNECs and SCLCs) is supported by the different molecular landscapes of these tumours 9, 12, 25.

When mutations and CNAs were combined, inactivating alterations of *TP53* and *RB1* were enriched in carcinomas (*p* < 0.0001), whereas *MEN1* alterations were almost exclusive to carcinoids (*p* = 0.0004). However, the fact that the same gene alterations found in carcinomas are identified in low‐grade tumours at lower prevalence rates may suggest the existence of a progression of malignancy, with the development of secondary high‐grade neuroendocrine tumours from pre‐existing carcinoids. This has been suggested on molecular grounds in pancreatic and thymus neuroendocrine tumours 26, 27, reinforcing the biological relevance of intratumour heterogeneity.

Chromatin‐remodelling genes, which include those encodig covalent histone modifiers and subunits of the SWI–SNF complex, emerged from the present study as the group of genes with the highest mutation rate in both carcinoids (45%) and carcinomas (55%). Fernandez‐Cuesta *et al* reported a similar mutation frequency of chromatin‐remodelling genes (52%) in their study of 44 carcinoids, including 35 TCs and nine ACs 9. A more recent study by Rekhtman *et al* reported a 78% mutation frequency of chromatin modifiers in 45 LCNECs 25; in the same publication, the authors also re‐analysed data of 42 SCLCs from Hyman *et al* 28 and Paik *et al* 29, and showed that 64% of SCLCs have chromatin‐remodelling gene alterations. Our direct comparison of all subtypes complements these previous observations, and suggests that chromatin modifiers are major players in the pathogenesis of all LNETs. However, their role in carcinoids may be more relevant, as these tumours have a low mutational background, whereas carcinomas have a high mutational background and feature inactivation of genes involved in cell cycle control, such as *TP53* and *RB1* (carcinomas versus carcinoids, *p* < 0.0001). Carcinomas are also enriched in mutations of the oncogenic *PI3K*/*AKT*/*mTOR* pathway (carcinomas versus carcinoids, *p* = 0.0313), a potential therapeutic target to be investigated in prospective trials 10.

In our mutational analysis, carcinoids showed a lower mean number of mutations (TCs, 0.7; ACs, 1.8) than carcinomas (LCNECs, 4.6; SCLCs, 5.8), in line with current literature. However, two carcinoids (one TC and one AC) showed a hypermutated phenotype, which is defined as a gross excess of point mutations relative to the same tumour type 30. The total numbers of mutations of these two cases were nine in the TC and 14 in the AC. Moreover, the total numbers of mutations determined on WES were 228 for the TC and 434 for the AC, well beyond the average mutational burden found on WES in our carcinoids (14.9 mutations/case) and those reported in the literature (14.0 mutations/case, as calculated from Fernandez‐Cuesta *et al*) 9. Common to these cases was the presence of inactivating mutations of *POLQ* that could be linked to this peculiar genomic profile. *POLQ* encodes DNA polymerase θ, an enzyme implicated in DNA repair 31: *POLQ*‐defective mammalian cells are susceptible to DNA double‐strand breaks, and show increased rates of homologous recombination 21, 32. Interestingly, one of these cases also showed CNAs in several chromosomes, suggestive of defective DNA repair. The hypermutated carcinoids had proliferation rates within the range of their respective WHO categories. Mitotic counts were TC = 1 and AC = 6 per 2 mm^2^, with the WHO criteria setting being 0–1 for TC and 2–10 for AC 1. The Ki67 proliferation index was TC = 7% and AC = 15%, which fits in the range of their respective categories 33. Moreover, these cases had no clinicopathological evidence of aggressiveness, and both patients were disease‐free at 21 months (TC) and 17 months (AC). As this is a short follow‐up for carcinoids, we have no definitive indication as to the clinical significance of this phenotype. A third carcinoid showed CNAs consistent with chromothripsis of chromosomes 2, 11, and 20. Chromothripsis has been recently described in a stage III atypical carcinoid of a smoker patient 9, whereas our case was a stage IV atypical carcinoid of a non‐smoker patient.

There were fewer CNAs in carcinoids than in carcinomas. Within individual subtypes, losses of *MEN1* in ACs and losses of *RB1* in SCLCs were the most frequent events, in agreement with previous literature 12, 34. However, an increase in the number of CNA events was observed from low‐grade to high‐grade tumours, especially for concomitant gains of *TERT*, *SDHA* and *RICTOR* located on the p‐arm of chromosome 5. On WES analysis, we observed gain of chromosome 5p, with *SDHA* and *RICTOR* being located at the edges of and *TERT* within the altered chromosomal region. Therefore, as the limited material available did not allow us to perform fluorescence *in situ* hybridization or WES analysis on the whole LNET series, these three genes were included in the validation NGS panels, and their simultaneous CNAs were assessed as a surrogate of 5p gains. Gain of 5p has already been reported as a common event in all lung cancer types, suggesting that genes located on this chromosomal arm might be involved in development of all lung tumour types 35. Of those genes, *RICTOR* encodes for a protein of the mTORC2 complex, whose amplification has recently been demonstrated to define the sensitivity of lung cancer cell lines to mTORC1/2 inhibitors 36. When mutational and CNA analysis were combined, ACs were shown to be hybrid tumours sharing molecular features of both low‐grade (TC) and high‐grade (LCNEC and SCLC) tumours. In particular, ACs had frequencies of *MEN1* losses and mutations comparable to those in TCs, and their overall mutational burden was 2.6 times higher than that in TCs and 2.9 times lower than that in carcinomas. However, they shared with carcinomas a similar prevalence of copy gains in several genes, including the chromosome 5p genes *TERT*, *SDHA*, and *RICTOR*, but also *SRC* and *MYCL*.

Survival analysis highlighted the prognostic relevance of *RB1* and *TERT* regardless of subtype, whereas *KMT2D* was prognostically relevant for SCLCs, and *MEN1* for ACs. Mutation of *RB1* and copy gain of *TERT* are reported herein for the first time as independent predictors of poor prognosis in patients with LNETs. A correlation between alterations of these genes and poor prognosis has been observed for *RB1* in non‐small‐cell lung cancers 37 and anaplastic astrocytoma 38, and increased mRNA expression of *TERT* was previously reported as a poor prognostic marker in breast 39 and lung 40 cancers. We report for the first time that mutations affecting *KMT2D* in SCLC patients correlate with longer survival, as previously observed for pancreatic adenocarcinoma 41. The value of *MEN1* mutation as an unfavourable prognostic predictor in patients with AC is in line with our previous finding of a significant association between inactivation of *MEN1* and poor prognosis 34.

The fact that our discovery set included a higher number of carcinoids than of carcinomas was attributable to the following: (1) the availability of high‐quality DNA from adequate amounts of frozen material – this is obtainable from surgically treated tumours, and SCLCs and LCNECs are rarely surgically resected; and (2) permission to use tissue for research, which is limited by the need to preserve material for clinical use. This could be considered a weakness of the study, as it might have led to a low‐grade‐driven validation gene list. However, to overcome this potential bias, we increased the gene list derived from the discovery screen with genes identified as altered in the current LNET literature 9, 10, 12, 13.

In conclusion, our comprehensive molecular study encompassing all four histological variants of LNET indicated that a featured molecular portrait stands behind the commonly adopted histological classification, with a mixture of genetic similarities and differences among the four WHO LNET histotypes. This was mirrored by survival analysis, in which alterations in *RB1* and *TERT* were common to all subtypes, and showed an independent poor prognostic role in all LNETs, whereas mutations of *MEN1* and *KMT2D* were specifically enriched and showed a prognostic impact in ACs and SCLCs, respectively. Other genes that we found to be altered in LNETs are, instead, amenable to therapeutic targeting, including alterations leading to *PI3K*/*AKT*/*mTOR* pathway activation, namely *PIK3CA* mutations and copy gains of *PIK3CA* and *RICTOR* 10. Our data suggest that molecular profiling may complement histology to provide better diagnostic definition and prognostic stratification of LNETs that would be helpful for their clinical management.

### Author contributions statement

The authors contributed in the following way: AS, MV: conceived the study; RF, SP, LM, FG, GF, EB: collected materials and clinical data; MF, BR, MV, GP, AS: reviewed the cases according to the WHO classification; CV: performed immunohistochemical analysis; RTL: coordinated patients and sample data management, and supervised ethical protocols; MF, BR: microdissected samples; MS, KS, AM, SB: carried out sequencing and raw data analysis; KS, AM, SB, MS performed bioinformatic analysis; MS, KS: designed the multigene panels; VC: supervised the validation experiments; MS, AM, MF, KS: drafted the manuscript; AM, MF, EB, VC, GT, MV, GP, RTL: revised the manuscript; AS: finalized the manuscript. All authors approved the submitted version.

**SUPPLEMENTARY MATERIAL ONLINE**

**Supplementary materials and methods**

**Supplementary figure legends**

**Figure S1A.** Somatic mutations identified by whole exome sequencing of 20 LNETs.

**Figure S1B.** Somatic mutations and copy number alterations identified by high coverage targeted sequencing of 418 genes on 46 LNETs.

**Figure S2.** Atypical carcinoid affected by chromothripsis.

**Figure S3.** Screening for molecular predictors of poor prognosis in LNET.

**Table S1.** Ampliseq custom panels used for validation of mutations in 88 genes and copy number alterations in 13 genes.

**Table S2.** Clinicopathological features of 148 lung neuroendocrine tumours and analyses executed.

**Table S3.** Prevalence of mutations in 88 genes for 148 lung neuroendocrine tumours. Related to Figure S2A and Table 2.

**Table S4A.** Discovery screen, whole exome sequencing: list of mutations found in 20 lung neuroendocrine tumours. Related to Supplementary Figure S1A.

**Table S4B.** Discovery screen, high coverage targeted sequencing of 418 genes: list of mutations found in 46 lung neuroendocrine tumours. Related to Supplementary Figure S1B.

**Table S4C.** Discovery screen, integration of whole exome sequencing and high coverage targeted sequencing of 418 genes: list of 36 genes mutated in at least two cases of 46 lung neuroendocrine tumours. Related to Supplementary Figures S1A and S1B.

**Table S5A.** Discovery screen, copy number alterations by whole exome sequencing: histotype‐specific distribution in 20 lung neuroendocrine tumours. Related to Figure 3.

**Table S5B.** Discovery screen, copy number alterations by high coverage targeted sequencing of 418 genes: histotype‐specific distribution in 46 lung neuroendocrine tumours. Related to Supplementary Figure S1B.

**Table S6.** Univariate analysis on 78 lung neuroendocrine tumours for selection of candidate molecular prognostic predictors to be included in multivariate survival analysis. Related to Supplementary Figure S3.

## Supporting information


**Supplementary materials and methods**

**Supplementary figure legends**

**Supplementary Figure S1A. Somatic mutations identified by whole exome sequencing of 20 LNET.** Cases are grouped according to the four histotypes as defined by the WHO classification. The histogram on top shows the total number of mutations identified. The driver plot displays genes that were mutated in at least two cases. The histogram on the left reports the alteration frequency of each gene, expressed as a percentage. Alterations are annotated according to the colour panel on the right. Asterisks indicate the TC and the AC hypermutated cases. Related to Figure 3B.
**Supplementary Figure S1B. Somatic mutations and copy number alterations identified by high coverage targeted sequencing of 418 genes on 46 LNET.** Cases are grouped according to the four histotypes as defined by the WHO classification. The histogram on top shows the total number of mutations identified, where asterisks indicate the TC and the AC hypermutated cases. The upper matrix shows mutated genes, the lower matrix shows copy number alterations. Histograms on the left report the alteration frequency of each gene. Alterations are annotated according to the colour panel at the right of the plot.

**Supplementary Figure S2. Atypical carcinoid affected by chromothripsis.** The log2RSeqC (log2 of normalized coverage) from whole exome sequencing is shown for case 034 (atypical carcinoid). The pattern is compatible with chromothripsis of chromosome 2, 11, and 20.

**Supplementary Figure S3. Screening for molecular predictors of poor prognosis in LNET.** For each alteration, univariate analysis, and multivariate analysis against tumour stage and histotype with the relative p‐value is reported. Log‐rank test and Cox proportional hazards regression were used to select candidate predictors (p<0.2) at univariate and multivariate analysis, respectively. X‐axes are trimmed for visualization purposes.

**Table S1.** Ampliseq custom panels used for validation of mutations in 88 genes and copy number alterations in 13 genes.

**Table S2.** Clinicopathological features of 148 lung neuroendocrine tumours and analyses executed.

**Table S3.** Prevalence of mutations in 88 genes for 148 lung neuroendocrine tumours. Related to Figure S2A and Table 2.

**Table S4A.** Discovery screen, whole exome sequencing: list of mutations found in 20 lung neuroendocrine tumours. Related to Supplementary Figure S1A.
**Table S4B.** Discovery screen, high coverage targeted sequencing of 418 genes: list of mutations found in 46 lung neuroendocrine tumours. Related to Supplementary Figure S1B.
**Table S4C.** Discovery screen, integration of whole exome sequencing and high coverage targeted sequencing of 418 genes: list of 36 genes mutated in at least two cases of 46 lung neuroendocrine tumours. Related to Supplementary Figures S1A and S1B.

**Table S5A.** Discovery screen, copy number alterations by whole exome sequencing: histotype‐specific distribution in 20 lung neuroendocrine tumours. Related to Figure 3.
**Table S5B.** Discovery screen, copy number alterations by high coverage targeted sequencing of 418 genes: histotype‐specific distribution in 46 lung neuroendocrine tumours. Related to Supplementary Figure S1B.

**Table S6.** Univariate analysis on 78 lung neuroendocrine tumours for selection of candidate molecular prognostic predictors to be included in multivariate survival analysis. Related to Supplementary Figure S3.
