# Supplementary material for: Lung neuroendocrine tumours: deep sequencing of the four World Health Organization histotypes reveals chromatin‐remodelling genes as major players and a prognostic role for TERT, RB1, MEN1 and KMT2D
Source: J Pathol. 2016 Dec 29;241(4):488–500. doi: 10.1002/path.4853 (PMC5324596; doi:10.1002/path.4853)
Supplement: Supplementary file 1 — Supplementary materials and methods [file PATH-241-488-s004.docx]

**SUPPLEMENTARY MATERIALS AND METHODS**

References are numbered according to the main text list.

**DNA extraction and qualification**

DNA was obtained from frozen samples using QIAmp DNA mini Kits (Qiagen) and from FFPE samples using QIAamp DNA FFPE Tissue Kits (Qiagen), after enrichment for neoplastic cellularity to at least 70% by manual microdissection. DNA was quantified and quality assessed as reported [14,15].

**Whole-exome sequencing**

*Illumina HiSeq2000*

Library preparation was performed using the Illumina® TruSeq DNA Sample Prep kit v2. In brief, 2 μg of genomic DNA were sheared using an ultrasonicator (Covaris E220). Overhanging DNA fragments were subsequently end-repaired, adenylated, ligated to indexing adapters and finally enriched by PCR, following the manufacturer’s instructions. TruSeq DNA libraries were validated by running an aliquot on a 2% agarose gel to verify the proper size of PCR-enriched fragments, and quantified by Qubit dsDNA BR assay (Thermo Fisher). Pools of six TruSeq DNA libraries (500 ng each) were processed using Illumina® TruSeq Exome Enrichment kit to select target regions, by following the manufacturer’s instructions. Enriched libraries were validated by running an aliquot on Agilent Bioanalyzer High Sensitivity chips, and quantified by both Bioanalyzer and Qubit dsDNA HS assay (Thermo Fisher). Finally, 10 pM dilutions of enriched libraries were sequenced on Illumina HiSeq2000.

Forward and reverse reads from de-multiplexed runs were 3'-end trimmed for nucleotides of quality <20 using the sickle algorithm (https://github.com/najoshi/sickle). Reads trimmed down to less than 30 nt were discarded. Reads that passed trimming were subjected to additional quality control using the Fast QC program (http://www.bioinformatics.babraham.ac.uk/projects/fastqc/). Genomic analysis was performed using the Genome analysis toolkit (GATK, https://software.broadinstitute.org/gatk/). Forward and reverse reads were aligned to the human genome (GATK repository, build 37) using BWA and saved in the BAM file format [16]. BAM files corresponding to single sequencing runs were subjected to PCR duplicate removal. Realignment around insertions/deletions and base recalibration were subsequently performed using GATK tools (release 2.2). On-target BAM files for tumour and normal samples were obtained by intersecting the base-recalibrated BAMs with the Illumina Truseq BED file, and used to compute alignment statistics and depth of on-target coverage (GATK tools).

*Variant calling*

Single nucleotide variant calling was performed using MuTect (https://www.broadinstitute.org/ cancer/cga/mutect) on tumour and normal on-target BAM files for each patient. Insertions and deletions variants were called using the GATK Somatic Indel Detector. Raw variant calls were annotated for presence in the dbSNP and COSMIC databases using SnpSift and mutation effect on gene function was predicted using SnpEff [17].

Raw MuTect call filtering involved retention of variants i) flagged “KEEP”; ii) with effect in the protein-coding regions or affecting splice sites; iii) without a matching entry in the dbSNP unless a COSMIC match found simultaneously; iv) mapping to proteins lacking repetitive/polymorphic domains; v) without a matching entry in the 1000 genomes variant callset; vi) with variant allele frequency ≥0.2; vii) representing single alteration in a given gene. Additionally we filtered out variants in genes reported as possible false positives [18].

Raw Somatic Indel Detector call filtering involved retention of variants: i) with effect in the protein-coding regions or affecting splice sites; ii) at least 10nt far from the read’s ends; iii) with p>0.02 for strand bias (Fisher’s test); iv) at least 50nt far from the Truseq interval borders; v) without a matching entry in the dbSNP or with a simultaneous match in dbSNP and COSMIC; vi) mapping to proteins lacking repetitive/polymorphic domains; vi) without a matching entry in the 1000 genomes variant callset; vii) with variant allele frequency ≥0.2; viii) representing single alteration in a given gene in the same patient if matching an entry on the Fuentes Fajardo list [18].

**High coverage targeted sequencing**

High coverage targeted sequencing (HCTS) of 418 genes was performed using a customized version of the AmpliSeq Comprehensive Cancer Panel (CCP) including the standard 409 genes and 9 additional genes deriving from whole exome sequencing data: DSCAML1, NCAM2, PCLO, PTPRZ1, RAI1, RIN3, SPHKAP, TDRD7, THSD7B. The panel was applied to the 46 samples included in the discovery screen to identify both mutations and copy number alterations (CNA). Forty nanograms of DNA were used for each multiplex PCR amplification. The quality of the obtained libraries was evaluated by the Agilent 2100 Bioanalyzer on-chip electrophoresis (Agilent Technologies). Emulsion PCR was performed with the OneTouch2 system (Thermo Fisher). Sequencing was run on the Ion Proton (PI, Thermo Fisher) loaded with Ion PI Chip v2. Data analysis, including alignment to the hg19 human reference genome, variant and CNA calling and annotation, was done using Ion Reporter v.5.0 and the paired samples workflow to match tumour and normal samples (Thermo Fisher). Additionally, alignments were visually verified with the Integrative Genomics Viewer (IGV) v2.3 [19] to further confirm the presence of mutations and CNA identified.

**Validation of frequently altered genes by next-generation targeted sequencing**

Matched tumour/normal DNA from 102 FFPE LNET was subjected to targeted-NGS to validate the prevalence of somatic alterations in 88 genes, selected as follows: 42 from the discovery screen, 46 genes from literature review [9,10,12,13]. The 42 genes selected from the discovery screen included: 36 recurrently (i.e. more than once) mutated genes, 7 of which also displayed CNA, and 6 genes with only recurrent CNA from the discovery screen;

To this end, we used four multigene panels: i) the commercially available 50-gene Ion AmpliSeq Cancer Hotspot panel v2 (Thermo Fisher; details on target regions of the commercial panel are at http://www.thermofisher.com), ii) three AmpliSeq custom panels targeting the entire coding sequence of 45 genes. Seven genes (FGFR1, PIK3CA, PTEN, RB1, SMAD4, SRC and TP53) are included in both the custom and the hotspot panels. The custom panels 1 and 2 were designed for mutational analysis and panel 3 for mutational and CNA analysis. Panel 1 and 2 include the following 32 genes: *ARID1A, ARID1B, ARID2, ATRX, CSMD3, DAXX, DSCAML1, EIF1AX, KAT6A, KAT6B, KMT2A, KMT2C, KMT2D, LRP1B, NCAM2, NOTCH2, PCLO, PSIP1, PTEN, PTPRZ1, RAI1, RIN3, SETD2, SMARCA1, SMARCA2, SMARCA3, SMARCA4, SPHKAP, TDRD7, THSD7B, TSC1, TSC2*. Target regions of genes *CSMD3, MEN1, PTPRZ1* and *TSC2* were split among two custom panels to optimize sequence coverage. Panel 3 targets the following 13 genes: *BCL2, FGFR1, MEN1, MYC, MYCL, PIK3CA, RB1, RICTOR, SDHA, SMAD4, SRC, TERT, TP53*. Details of the custom panels are in Supplementary Table S1. Custom panels were designed using the AmpliSeq designer software v1.2 (ThermoFisher).

Twenty nanograms of DNA were used for each multiplex PCR amplification. Emulsion PCR was performed with the OneTouch2 system (Life Technologies). The quality of the obtained libraries was evaluated by the Agilent 2100 Bioanalyzer on-chip electrophoresis (Agilent Technologies). Sequencing was run on the Ion Proton (PI, Life Technologies) loaded with Ion PI Chip v2. Data analysis, including alignment to the hg19 human reference genome and variant calling, was done using the Torrent Suite Software v.5.0 (Life Technologies). Filtered variants were annotated using a custom pipeline based on vcflib (https://github.com/ekg/vcflib), SnpSift [17], the Variant Effect Predictor (VEP) software [20] and NCBI RefSeq database. Alignments were visually verified with the Integrative Genomics Viewer (IGV) v2.3 [19].

CNA analysis was performed on IonReporter 5.0 software (ThermoFisher) with the CNV single sample workflow. A specific baseline was built using normal and well characterised DNA without genomic structural aberrations (10 male DNA extracted from normal tissues). The baseline was matched to the sequences of the validation cohort LNET samples to obtain CNA status of the genes analysed.
