# Supplementary material for: Lung neuroendocrine tumours: deep sequencing of the four World Health Organization histotypes reveals chromatin‐remodelling genes as major players and a prognostic role for TERT, RB1, MEN1 and KMT2D
Source: J Pathol. 2016 Dec 29;241(4):488–500. doi: 10.1002/path.4853 (PMC5324596; doi:10.1002/path.4853)
Supplement: Supplementary file 2 — Supplementary figure legends [file PATH-241-488-s011.doc]

**Supplementary Figure S1A. Somatic mutations identified by whole exome sequencing of 20 LNET.** Cases are grouped according to the four histotypes as defined by the WHO classification. The histogram on top shows the total number of mutations identified. The driver plot displays genes that were mutated in at least two cases. The histogram on the left reports the alteration frequency of each gene, expressed as a percentage. Alterations are annotated according to the colour panel on the right. Asterisks indicate the TC and the AC hypermutated cases. Related to Figure 3B.

**Supplementary Figure S1B. Somatic mutations and copy number alterations identified by high coverage targeted sequencing of 418 genes on 46 LNET.** Cases are grouped according to the four histotypes as defined by the WHO classification. The histogram on top shows the total number of mutations identified, where asterisks indicate the TC and the AC hypermutated cases. The upper matrix shows mutated genes, the lower matrix shows copy number alterations. Histograms on the left report the alteration frequency of each gene. Alterations are annotated according to the colour panel at the right of the plot.

**Supplementary Figure S2. Atypical carcinoid affected by chromothripsis.** The log2RSeqC (log2 of normalized coverage) from whole exome sequencing is shown for case 034 (atypical carcinoid). The pattern is compatible with chromothripsis of chromosome 2, 11, and 20.

**Supplementary Figure S3. Screening for molecular predictors of poor prognosis in LNET.** For each alteration, univariate analysis, and multivariate analysis against tumour stage and histotype with the relative p-value is reported. Log-rank test and Cox proportional hazards regression were used to select candidate predictors (*p*<0.2) at univariate and multivariate analysis, respectively. X-axes are trimmed for visualization purposes.
