# Supplementary material for: Lung neuroendocrine tumours: deep sequencing of the four World Health Organization histotypes reveals chromatin‐remodelling genes as major players and a prognostic role for TERT, RB1, MEN1 and KMT2D
Source: J Pathol. 2016 Dec 29;241(4):488–500. doi: 10.1002/path.4853 (PMC5324596; doi:10.1002/path.4853)
Supplement: Supplementary file 6 — Table S1. Ampliseq custom panels used for validation of mutations in 88 genes and copy number alterations in 13 genes. [file PATH-241-488-s008.pdf]

Supplementary Table S1 : Ampliseq custom panels used for validation of mutations in 88 genes and copy number alterations (CNA) in 13 genes

LNET Custom Panel 1
for mutational analysis

GENE	CHR	START	END
ARID1A	chr1	27023412	27023521
ARID1A	chr1	27024045	27024151
ARID1A	chr1	27055998	27056119
ARID1A	chr1	27056108	27056238
ARID1A	chr1	27056216	27056335
ARID1A	chr1	27056308	27056406
ARID1A	chr1	27057575	27057695
ARID1A	chr1	27057684	27057817
ARID1A	chr1	27057809	27057940
ARID1A	chr1	27057916	27058047
ARID1A	chr1	27058069	27058149
ARID1A	chr1	27059115	27059243
ARID1A	chr1	27059232	27059333
ARID1A	chr1	27087282	27087405
ARID1A	chr1	27087371	27087442
ARID1A	chr1	27087477	27087606
ARID1A	chr1	27087599	27087705
ARID1A	chr1	27087816	27087941
ARID1A	chr1	27087936	27088019
ARID1A	chr1	27088576	27088700
ARID1A	chr1	27088658	27088752
ARID1A	chr1	27088741	27088860
ARID1A	chr1	27089349	27089478
ARID1A	chr1	27089445	27089557
ARID1A	chr1	27089602	27089710
ARID1A	chr1	27089672	27089751
ARID1A	chr1	27092654	27092779
ARID1A	chr1	27092768	27092863
ARID1A	chr1	27092852	27092932
ARID1A	chr1	27092921	27093035
ARID1A	chr1	27093022	27093107
ARID1A	chr1	27094226	27094352

LNET Custom Panel 2
for mutational analysis

GENE	CHR	START	END
CSMD3	chr8	113236965	113237091
CSMD3	chr8	113237080	113237179
CSMD3	chr8	113240930	113241041
CSMD3	chr8	113241030	113241097
CSMD3	chr8	113243758	113243875
CSMD3	chr8	113246540	113246623
CSMD3	chr8	113246612	113246705
CSMD3	chr8	113246701	113246810
CSMD3	chr8	113249405	113249480
CSMD3	chr8	113249469	113249590
CSMD3	chr8	113253902	113254006
CSMD3	chr8	113253995	113254098
CSMD3	chr8	113256567	113256692
CSMD3	chr8	113256681	113256796
CSMD3	chr8	113259193	113259293
CSMD3	chr8	113259282	113259394
CSMD3	chr8	113266437	113266526
CSMD3	chr8	113266515	113266595
CSMD3	chr8	113267468	113267542
CSMD3	chr8	113267531	113267607
CSMD3	chr8	113267596	113267710
CSMD3	chr8	113275814	113275935
CSMD3	chr8	113275926	113276049
CSMD3	chr8	113277629	113277727
CSMD3	chr8	113277716	113277829
CSMD3	chr8	113293312	113293436
CSMD3	chr8	113293426	113293505
CSMD3	chr8	113293494	113293610
CSMD3	chr8	113299202	113299316
CSMD3	chr8	113299303	113299377
CSMD3	chr8	113299366	113299487
CSMD3	chr8	113301474	113301598

LNET Custom Panel 3
for mutational and CNA analysis

GENE	CHR	START	END
BCL2	chr18	60795735	60795844
BCL2	chr18	60795833	60795937
BCL2	chr18	60795928	60796058
BCL2	chr18	60985202	60985322
BCL2	chr18	60985309	60985439
BCL2	chr18	60985428	60985538
BCL2	chr18	60985518	60985612
BCL2	chr18	60985627	60985744
BCL2	chr18	60985761	60985854
BCL2	chr18	60985843	60985965
FGFR1	chr8	38271444	38271567
FGFR1	chr8	38271685	38271812
FGFR1	chr8	38272269	38272345
FGFR1	chr8	38275047	38275130
FGFR1	chr8	38279309	38279403
FGFR1	chr8	38281234	38281342
FGFR1	chr8	38282140	38282254
FGFR1	chr8	38283567	38283689
FGFR1	chr8	38285430	38285526
FGFR1	chr8	38285848	38285945
FGFR1	chr8	38285851	38285975
FGFR1	chr8	38287084	38287208
FGFR1	chr8	38289028	38289147
FGFR1	chr8	38292214	38292289
FGFR1	chr8	38293010	38293121
FGFR1	chr8	38299647	38299726
FGFR1	chr8	38306706	38306803
FGFR1	chr8	38308209	38308324
FGFR1	chr8	38308589	38308683
FGFR1	chr8	38314987	38315089
FGFR1	chr8	38315534	38315644
MEN1	chr11	64571750	64571847

ARID1A	chr1	27094333	27094458	CSMD3	chr8	113301587	113301698	MEN1	chr11	64571847	64571973
ARID1A	chr1	27094447	27094540	CSMD3	chr8	113301687	113301801	MEN1	chr11	64571973	64572103
ARID1A	chr1	27097521	27097634	CSMD3	chr8	113303704	113303817	MEN1	chr11	64572141	64572263
ARID1A	chr1	27097623	27097709	CSMD3	chr8	113303806	113303916	MEN1	chr11	64572181	64572304
ARID1A	chr1	27097698	27097780	CSMD3	chr8	113303916	113304035	MEN1	chr11	64572475	64572602
ARID1A	chr1	27097777	27097876	CSMD3	chr8	113304747	113304848	MEN1	chr11	64572601	64572693
ARID1A	chr1	27098849	27098969	CSMD3	chr8	113304837	113304959	MEN1	chr11	64573066	64573197
ARID1A	chr1	27098958	27099081	CSMD3	chr8	113308042	113308111	MEN1	chr11	64573197	64573279
ARID1A	chr1	27099070	27099157	CSMD3	chr8	113308100	113308213	MEN1	chr11	64573658	64573788
ARID1A	chr1	27099250	27099334	CSMD3	chr8	113308202	113308290	MEN1	chr11	64573788	64573923
ARID1A	chr1	27099323	27099432	CSMD3	chr8	113313967	113314047	MEN1	chr11	64574457	64574583
ARID1A	chr1	27099421	27099537	CSMD3	chr8	113314036	113314116	MEN1	chr11	64574603	64574734
ARID1A	chr1	27099719	27099846	CSMD3	chr8	113314105	113314218	MEN1	chr11	64575058	64575171
ARID1A	chr1	27099835	27099966	CSMD3	chr8	113316907	113316975	MEN1	chr11	64575328	64575456
ARID1A	chr1	27099964	27100058	CSMD3	chr8	113316964	113317060	MEN1	chr11	64575453	64575577
ARID1A	chr1	27100047	27100153	CSMD3	chr8	113317049	113317154	MEN1	chr11	64577098	64577212
ARID1A	chr1	27100142	27100260	CSMD3	chr8	113318222	113318325	MEN1	chr11	64577212	64577323
ARID1A	chr1	27100218	27100303	CSMD3	chr8	113318314	113318433	MEN1	chr11	64577317	64577437
ARID1A	chr1	27100388	27100460	CSMD3	chr8	113323160	113323263	MEN1	chr11	64577435	64577548
ARID1A	chr1	27100745	27100876	CSMD3	chr8	113323252	113323341	MEN1	chr11	64577539	64577670
ARID1A	chr1	27100855	27100984	CSMD3	chr8	113323330	113323436	MYC	chr8	128748773	128748860
ARID1A	chr1	27101006	27101125	CSMD3	chr8	113326074	113326188	MYC	chr8	128748849	128748926
ARID1A	chr1	27101097	27101178	CSMD3	chr8	113326183	113326264	MYC	chr8	128750342	128750453
ARID1A	chr1	27101162	27101283	CSMD3	chr8	113326262	113326365	MYC	chr8	128750445	128750545
ARID1A	chr1	27101310	27101442	CSMD3	chr8	113326578	113326682	MYC	chr8	128750534	128750648
ARID1A	chr1	27101330	27101462	CSMD3	chr8	113326671	113326762	MYC	chr8	128750615	128750738
ARID1A	chr1	27101538	27101669	CSMD3	chr8	113326751	113326860	MYC	chr8	128750734	128750866
ARID1A	chr1	27101660	27101761	CSMD3	chr8	113330969	113331087	MYC	chr8	128750855	128750934
ARID1A	chr1	27101996	27102119	CSMD3	chr8	113331076	113331196	MYC	chr8	128750914	128751032
ARID1A	chr1	27102106	27102190	CSMD3	chr8	113332106	113332232	MYC	chr8	128751050	128751175
ARID1A	chr1	27102179	27102253	CSMD3	chr8	113347526	113347616	MYC	chr8	128751171	128751284
ARID1A	chr1	27105423	27105539	CSMD3	chr8	113347605	113347715	MYC	chr8	128751274	128751349
ARID1A	chr1	27105528	27105633	CSMD3	chr8	113348807	113348931	MYC	chr8	128752582	128752704
ARID1A	chr1	27105621	27105737	CSMD3	chr8	113348931	113349002	MYC	chr8	128752693	128752788
ARID1A	chr1	27105726	27105849	CSMD3	chr8	113348991	113349095	MYC	chr8	128752844	128752935
ARID1A	chr1	27105838	27105963	CSMD3	chr8	113349759	113349876	MYC	chr8	128752914	128753030
ARID1A	chr1	27105955	27106049	CSMD3	chr8	113349865	113349985	MYC	chr8	128753008	128753107
ARID1A	chr1	27106046	27106167	CSMD3	chr8	113353597	113353714	MYC	chr8	128753096	128753175
ARID1A	chr1	27106156	27106286	CSMD3	chr8	113353728	113353826	MYC	chr8	128753164	128753254
ARID1A	chr1	27106263	27106384	CSMD3	chr8	113353815	113353929	MYCL1	chr1	40362935	40363054

ARID1A	chr1	27106382	27106514	CSMD3	chr8	113358235	113358354	MYCL1	chr1	40363027	40363101
ARID1A	chr1	27106506	27106625	CSMD3	chr8	113358346	113358452	MYCL1	chr1	40363156	40363272
ARID1A	chr1	27106616	27106732	CSMD3	chr8	113363294	113363407	MYCL1	chr1	40363183	40363300
ARID1A	chr1	27106684	27106772	CSMD3	chr8	113363438	113363552	MYCL1	chr1	40363403	40363511
ARID1A	chr1	27106837	27106951	CSMD3	chr8	113364622	113364697	MYCL1	chr1	40363489	40363588
ARID1A	chr1	27106865	27106981	CSMD3	chr8	113364686	113364782	MYCL1	chr1	40363615	40363693
ARID1A	chr1	27107058	27107151	CSMD3	chr8	113392563	113392677	MYCL1	chr1	40366400	40366517
ARID1A	chr1	27107140	27107217	CSMD3	chr8	113392632	113392702	MYCL1	chr1	40366490	40366599
ARID1A	chr1	27107206	27107297	CSMD3	chr8	113395733	113395833	MYCL1	chr1	40366572	40366697
ARID1A	chr1	27023113	27023236	CSMD3	chr8	113395822	113395930	MYCL1	chr1	40366666	40366772
ARID1A	chr1	27023236	27023335	CSMD3	chr8	113402813	113402931	MYCL1	chr1	40366856	40366992
ARID1A	chr1	27023355	27023491	CSMD3	chr8	113402923	113403030	MYCL1	chr1	40366897	40367010
ARID1A	chr1	27023413	27023517	CSMD3	chr8	113418754	113418818	PIK3CA	chr3	178870451	178870541
ARID1A	chr1	27023684	27023776	CSMD3	chr8	113418807	113418886	PIK3CA	chr3	178870885	178870997
ARID1A	chr1	27023697	27023836	CSMD3	chr8	113418875	113418991	PIK3CA	chr3	178873968	178874080
ARID1A	chr1	27024034	27024137	CSMD3	chr8	113420499	113420606	PIK3CA	chr3	178878790	178878899
ARID1A	chr1	27056119	27056237	CSMD3	chr8	113420595	113420713	PIK3CA	chr3	178881262	178881358
ARID1A	chr1	27056232	27056362	CSMD3	chr8	113421124	113421197	PIK3CA	chr3	178882865	178882976
ARID1A	chr1	27057577	27057702	CSMD3	chr8	113421186	113421279	PIK3CA	chr3	178884569	178884673
ARID1A	chr1	27057702	27057827	CSMD3	chr8	113484798	113484914	PIK3CA	chr3	178898207	178898277
ARID1A	chr1	27057827	27057961	CSMD3	chr8	113504619	113504744	PIK3CA	chr3	178910028	178910134
ARID1A	chr1	27057956	27058065	CSMD3	chr8	113504733	113504834	PIK3CA	chr3	178910292	178910352
ARID1A	chr1	27058060	27058143	CSMD3	chr8	113504823	113504936	PIK3CA	chr3	178910848	178910935
ARID1A	chr1	27059099	27059226	CSMD3	chr8	113515948	113516057	PIK3CA	chr3	178916679	178916785
ARID1A	chr1	27059226	27059320	CSMD3	chr8	113516046	113516115	PIK3CA	chr3	178916775	178916881
ARID1A	chr1	27087254	27087381	CSMD3	chr8	113516104	113516216	PIK3CA	chr3	178916844	178916930
ARID1A	chr1	27087378	27087463	CSMD3	chr8	113518872	113518951	PIK3CA	chr3	178916931	178917035
ARID1A	chr1	27087463	27087593	CSMD3	chr8	113518940	113519018	PIK3CA	chr3	178917406	178917494
ARID1A	chr1	27087846	27087928	CSMD3	chr8	113519007	113519124	PIK3CA	chr3	178917416	178917537
ARID1A	chr1	27087928	27088027	CSMD3	chr8	113529212	113529315	PIK3CA	chr3	178918889	178918972
ARID1A	chr1	27088591	27088717	CSMD3	chr8	113529304	113529396	PIK3CA	chr3	178920590	178920694
ARID1A	chr1	27088715	27088825	CSMD3	chr8	113529378	113529489	PIK3CA	chr3	178921464	178921570
ARID1A	chr1	27089430	27089556	CSMD3	chr8	113562879	113562984	PIK3CA	chr3	178922252	178922365
ARID1A	chr1	27089548	27089662	CSMD3	chr8	113562973	113563075	PIK3CA	chr3	178927405	178927525
ARID1A	chr1	27089661	27089788	CSMD3	chr8	113563064	113563163	PIK3CA	chr3	178927901	178927986
ARID1A	chr1	27092652	27092766	CSMD3	chr8	113564787	113564867	PIK3CA	chr3	178928069	178928160
ARID1A	chr1	27092766	27092871	CSMD3	chr8	113564856	113564970	PIK3CA	chr3	178936023	178936105
ARID1A	chr1	27092879	27093004	CSMD3	chr8	113568911	113569032	PIK3CA	chr3	178936052	178936179
ARID1A	chr1	27093004	27093107	CSMD3	chr8	113569020	113569122	PIK3CA	chr3	178938787	178938918
ARID1A	chr1	27094197	27094298	CSMD3	chr8	113569111	113569200	PIK3CA	chr3	178938831	178938960

ARID1A	chr1	27094298	27094424	CSMD3	chr8	113585714	113585826	PIK3CA	chr3	178944129	178944243
ARID1A	chr1	27094424	27094537	CSMD3	chr8	113585815	113585916	PIK3CA	chr3	178944326	178944439
ARID1A	chr1	27097584	27097698	CSMD3	chr8	113599182	113599299	PIK3CA	chr3	178945307	178945413
ARID1A	chr1	27097698	27097779	CSMD3	chr8	113599299	113599413	PIK3CA	chr3	178947818	178947896
ARID1A	chr1	27097779	27097903	CSMD3	chr8	113599403	113599518	PIK3CA	chr3	178947985	178948083
ARID1A	chr1	27098908	27099036	CSMD3	chr8	113649031	113649155	PIK3CA	chr3	178951914	178951991
ARID1A	chr1	27099036	27099164	CSMD3	chr8	113649144	113649268	PIK3CA	chr3	178951996	178952097
ARID1A	chr1	27099250	27099376	CSMD3	chr8	113650908	113651006	PIK3CA	chr3	178952140	178952237
ARID1A	chr1	27099376	27099485	CSMD3	chr8	113650995	113651085	RB1	chr13	48878051	48878181
ARID1A	chr1	27099829	27099944	CSMD3	chr8	113651074	113651168	RB1	chr13	48878193	48878331
ARID1A	chr1	27099939	27100026	CSMD3	chr8	113657250	113657368	RB1	chr13	48881337	48881436
ARID1A	chr1	27100047	27100153	CSMD3	chr8	113657357	113657464	RB1	chr13	48881430	48881538
ARID1A	chr1	27100151	27100274	CSMD3	chr8	113662275	113662395	RB1	chr13	48881536	48881656
ARID1A	chr1	27100271	27100408	CSMD3	chr8	113662384	113662475	RB1	chr13	48916693	48916804
ARID1A	chr1	27100757	27100894	CSMD3	chr8	113662464	113662588	RB1	chr13	48916793	48916898
ARID1A	chr1	27100894	27101000	CSMD3	chr8	113668359	113668484	RB1	chr13	48919129	48919237
ARID1A	chr1	27101000	27101091	CSMD3	chr8	113668444	113668514	RB1	chr13	48919225	48919319
ARID1A	chr1	27101088	27101192	CSMD3	chr8	113678399	113678522	RB1	chr13	48919324	48919439
ARID1A	chr1	27101191	27101310	CSMD3	chr8	113678511	113678620	RB1	chr13	48921963	48922047
ARID1A	chr1	27101310	27101441	CSMD3	chr8	113678609	113678709	RB1	chr13	48923021	48923134
ARID1A	chr1	27101436	27101545	CSMD3	chr8	113694602	113694673	RB1	chr13	48923123	48923192
ARID1A	chr1	27101545	27101642	CSMD3	chr8	113694662	113694781	RB1	chr13	48934100	48934221
ARID1A	chr1	27101641	27101750	CSMD3	chr8	113694770	113694886	RB1	chr13	48934210	48934282
ARID1A	chr1	27102059	27102138	CSMD3	chr8	113697561	113697679	RB1	chr13	48936960	48937044
ARID1A	chr1	27102138	27102219	CSMD3	chr8	113697668	113697794	RB1	chr13	48937033	48937120
ARID1A	chr1	27105423	27105540	CSMD3	chr8	113697783	113697883	RB1	chr13	48938955	48939069
ARID1A	chr1	27105540	27105641	CSMD3	chr8	113697872	113697960	RB1	chr13	48939034	48939130
ARID1A	chr1	27105640	27105756	CSMD3	chr8	113697964	113698083	RB1	chr13	48941602	48941717
ARID1A	chr1	27105756	27105848	CSMD3	chr8	113702075	113702196	RB1	chr13	48941706	48941808
ARID1A	chr1	27105848	27105962	CSMD3	chr8	113702185	113702289	RB1	chr13	48942595	48942699
ARID1A	chr1	27105962	27106049	CSMD3	chr8	113812311	113812434	RB1	chr13	48942688	48942783
ARID1A	chr1	27106046	27106173	CSMD3	chr8	113812423	113812518	RB1	chr13	48947516	48947611
ARID1A	chr1	27106172	27106284	CSMD3	chr8	113841837	113841952	RB1	chr13	48947620	48947733
ARID1A	chr1	27106282	27106363	CSMD3	chr8	113841941	113842046	RB1	chr13	48950955	48951067
ARID1A	chr1	27106363	27106494	CSMD3	chr8	113871315	113871406	RB1	chr13	48951059	48951169
ARID1A	chr1	27106494	27106573	CSMD3	chr8	113871395	113871507	RB1	chr13	48951161	48951274
ARID1A	chr1	27106571	27106663	CSMD3	chr8	113933797	113933903	RB1	chr13	48953704	48953766
ARID1A	chr1	27106661	27106760	CSMD3	chr8	113933892	113934012	RB1	chr13	48953755	48953872
ARID1A	chr1	27106760	27106852	CSMD3	chr8	113959936	113960060	RB1	chr13	48954206	48954289
ARID1A	chr1	27106850	27106958	CSMD3	chr8	113960086	113960205	RB1	chr13	48954209	48954330

ARID1A	chr1	27106956	27107069	CSMD3	chr8	113966869	113966978	RB1	chr13	48955301	48955421
ARID1A	chr1	27107068	27107181	CSMD3	chr8	113966964	113967053	RB1	chr13	48955410	48955525
ARID1A	chr1	27107181	27107284	CSMD3	chr8	113988046	113988143	RB1	chr13	48955514	48955594
ARID1B	chr6	157099152	157099269	CSMD3	chr8	113988132	113988257	RB1	chr13	49027102	49027198
ARID1B	chr6	157099279	157099391	CSMD3	chr8	113988246	113988368	RB1	chr13	49027187	49027261
ARID1B	chr6	157099393	157099518	CSMD3	chr8	113988357	113988468	RB1	chr13	49030324	49030440
ARID1B	chr6	157099755	157099854	CSMD3	chr8	114031248	114031329	RB1	chr13	49030427	49030504
ARID1B	chr6	157099842	157099942	CSMD3	chr8	114031318	114031436	RB1	chr13	49033732	49033845
ARID1B	chr6	157100608	157100728	CSMD3	chr8	114110959	114111086	RB1	chr13	49033834	49033928
ARID1B	chr6	157150309	157150399	CSMD3	chr8	114111075	114111173	RB1	chr13	49033917	49033991
ARID1B	chr6	157150388	157150487	CSMD3	chr8	114185925	114186044	RB1	chr13	49037806	49037916
ARID1B	chr6	157150469	157150585	CSMD3	chr8	114186040	114186150	RB1	chr13	49037898	49037965
ARID1B	chr6	157192657	157192777	CSMD3	chr8	114186139	114186240	RB1	chr13	49039027	49039142
ARID1B	chr6	157192766	157192894	CSMD3	chr8	114290800	114290918	RB1	chr13	49039140	49039220
ARID1B	chr6	157222444	157222564	CSMD3	chr8	114290908	114291025	RB1	chr13	49039209	49039275
ARID1B	chr6	157222554	157222686	CSMD3	chr8	114326780	114326897	RB1	chr13	49039301	49039408
ARID1B	chr6	157222637	157222727	CSMD3	chr8	114326886	114326984	RB1	chr13	49039397	49039515
ARID1B	chr6	157256528	157256650	CSMD3	chr8	114326973	114327089	RB1	chr13	49047382	49047501
ARID1B	chr6	157256635	157256704	CSMD3	chr8	114388871	114388989	RB1	chr13	49047511	49047583
ARID1B	chr6	157405702	157405830	CSMD3	chr8	114388978	114389082	RB1	chr13	49050800	49050910
ARID1B	chr6	157405819	157405934	CSMD3	chr8	114448856	114448967	RB1	chr13	49050903	49051028
ARID1B	chr6	157405993	157406080	CSMD3	chr8	114448956	114449065	RB1	chr13	49051420	49051523
ARID1B	chr6	157406060	157406147	CSMD3	chr8	114449054	114449164	RB1	chr13	49051514	49051614
ARID1B	chr6	157431547	157431677	DSCAML1	chr11	117298987	117299112	RB1	chr13	49054107	49054232
ARID1B	chr6	157431658	157431748	DSCAML1	chr11	117299301	117299408	RICTOR	chr5	38942337	38942464
ARID1B	chr6	157454099	157454182	DSCAML1	chr11	117299403	117299529	RICTOR	chr5	38942453	38942583
ARID1B	chr6	157454171	157454288	DSCAML1	chr11	117301363	117301498	RICTOR	chr5	38942875	38942963
ARID1B	chr6	157454277	157454359	DSCAML1	chr11	117301478	117301590	RICTOR	chr5	38942952	38943047
ARID1B	chr6	157454348	157454432	DSCAML1	chr11	117301579	117301700	RICTOR	chr5	38943036	38943156
ARID1B	chr6	157469605	157469728	DSCAML1	chr11	117301682	117301787	RICTOR	chr5	38944435	38944558
ARID1B	chr6	157469717	157469843	DSCAML1	chr11	117302178	117302287	RICTOR	chr5	38944550	38944659
ARID1B	chr6	157469842	157469960	DSCAML1	chr11	117302284	117302414	RICTOR	chr5	38944648	38944752
ARID1B	chr6	157469949	157470068	DSCAML1	chr11	117302400	117302507	RICTOR	chr5	38944912	38945028
ARID1B	chr6	157470029	157470151	DSCAML1	chr11	117302953	117303081	RICTOR	chr5	38945017	38945124
ARID1B	chr6	157488024	157488141	DSCAML1	chr11	117303075	117303177	RICTOR	chr5	38945113	38945220
ARID1B	chr6	157488130	157488254	DSCAML1	chr11	117303154	117303265	RICTOR	chr5	38945505	38945602
ARID1B	chr6	157488249	157488369	DSCAML1	chr11	117303838	117303948	RICTOR	chr5	38945591	38945711
ARID1B	chr6	157495070	157495197	DSCAML1	chr11	117303921	117304031	RICTOR	chr5	38945700	38945818
ARID1B	chr6	157495186	157495303	DSCAML1	chr11	117304053	117304162	RICTOR	chr5	38945817	38945920
ARID1B	chr6	157502007	157502127	DSCAML1	chr11	117304151	117304227	RICTOR	chr5	38946479	38946575

ARID1B	chr6	157502109	157502191	DSCAML1	chr11	117306270	117306377	RICTOR	chr5	38946564	38946670
ARID1B	chr6	157502180	157502309	DSCAML1	chr11	117306352	117306481	RICTOR	chr5	38946656	38946756
ARID1B	chr6	157502298	157502398	DSCAML1	chr11	117306470	117306564	RICTOR	chr5	38947302	38947425
ARID1B	chr6	157505223	157505338	DSCAML1	chr11	117307815	117307935	RICTOR	chr5	38947414	38947513
ARID1B	chr6	157505327	157505436	DSCAML1	chr11	117307877	117308006	RICTOR	chr5	38947491	38947558
ARID1B	chr6	157505422	157505511	DSCAML1	chr11	117308032	117308141	RICTOR	chr5	38949336	38949451
ARID1B	chr6	157505495	157505626	DSCAML1	chr11	117308490	117308623	RICTOR	chr5	38949445	38949557
ARID1B	chr6	157510636	157510743	DSCAML1	chr11	117308639	117308735	RICTOR	chr5	38949546	38949634
ARID1B	chr6	157510732	157510824	DSCAML1	chr11	117308723	117308808	RICTOR	chr5	38949729	38949835
ARID1B	chr6	157510796	157510920	DSCAML1	chr11	117308791	117308901	RICTOR	chr5	38949824	38949943
ARID1B	chr6	157510909	157510983	DSCAML1	chr11	117309526	117309653	RICTOR	chr5	38949932	38950055
ARID1B	chr6	157511103	157511229	DSCAML1	chr11	117309928	117310041	RICTOR	chr5	38950044	38950167
ARID1B	chr6	157511218	157511332	DSCAML1	chr11	117310017	117310113	RICTOR	chr5	38950156	38950278
ARID1B	chr6	157511321	157511402	DSCAML1	chr11	117310102	117310213	RICTOR	chr5	38950267	38950386
ARID1B	chr6	157517235	157517351	DSCAML1	chr11	117310484	117310593	RICTOR	chr5	38950375	38950475
ARID1B	chr6	157517339	157517417	DSCAML1	chr11	117310582	117310695	RICTOR	chr5	38950456	38950571
ARID1B	chr6	157517399	157517472	DSCAML1	chr11	117314567	117314697	RICTOR	chr5	38950560	38950671
ARID1B	chr6	157517461	157517561	DSCAML1	chr11	117314686	117314769	RICTOR	chr5	38950661	38950776
ARID1B	chr6	157519845	157519952	DSCAML1	chr11	117321219	117321340	RICTOR	chr5	38950765	38950875
ARID1B	chr6	157519948	157520071	DSCAML1	chr11	117321314	117321422	RICTOR	chr5	38952181	38952300
ARID1B	chr6	157521727	157521819	DSCAML1	chr11	117329356	117329489	RICTOR	chr5	38952291	38952390
ARID1B	chr6	157521808	157521929	DSCAML1	chr11	117329488	117329598	RICTOR	chr5	38952379	38952500
ARID1B	chr6	157521918	157522037	DSCAML1	chr11	117329617	117329708	RICTOR	chr5	38952491	38952606
ARID1B	chr6	157522023	157522134	DSCAML1	chr11	117332094	117332175	RICTOR	chr5	38953000	38953069
ARID1B	chr6	157522123	157522242	DSCAML1	chr11	117332164	117332262	RICTOR	chr5	38953058	38953136
ARID1B	chr6	157522231	157522360	DSCAML1	chr11	117332255	117332354	RICTOR	chr5	38953125	38953243
ARID1B	chr6	157522299	157522425	DSCAML1	chr11	117335630	117335758	RICTOR	chr5	38953407	38953520
ARID1B	chr6	157522448	157522566	DSCAML1	chr11	117335755	117335834	RICTOR	chr5	38953551	38953629
ARID1B	chr6	157522555	157522677	DSCAML1	chr11	117335817	117335922	RICTOR	chr5	38953618	38953715
ARID1B	chr6	157524866	157524990	DSCAML1	chr11	117340496	117340622	RICTOR	chr5	38954821	38954941
ARID1B	chr6	157524979	157525089	DSCAML1	chr11	117340622	117340714	RICTOR	chr5	38954960	38955083
ARID1B	chr6	157525078	157525183	DSCAML1	chr11	117342523	117342656	RICTOR	chr5	38955592	38955719
ARID1B	chr6	157527223	157527336	DSCAML1	chr11	117342649	117342762	RICTOR	chr5	38955708	38955825
ARID1B	chr6	157527325	157527449	DSCAML1	chr11	117351063	117351201	RICTOR	chr5	38955793	38955864
ARID1B	chr6	157527433	157527523	DSCAML1	chr11	117351263	117351358	RICTOR	chr5	38957621	38957735
ARID1B	chr6	157527512	157527631	DSCAML1	chr11	117351829	117351924	RICTOR	chr5	38957724	38957818
ARID1B	chr6	157527626	157527707	DSCAML1	chr11	117351911	117352010	RICTOR	chr5	38957807	38957920
ARID1B	chr6	157527694	157527820	DSCAML1	chr11	117352617	117352706	RICTOR	chr5	38958464	38958582
ARID1B	chr6	157527801	157527899	DSCAML1	chr11	117352691	117352806	RICTOR	chr5	38958571	38958651
ARID1B	chr6	157527888	157528004	DSCAML1	chr11	117352787	117352903	RICTOR	chr5	38958637	38958752

ARID1B	chr6	157527993	157528099	DSCAML1	chr11	117374442	117374573	RICTOR	chr5	38958795	38958920
ARID1B	chr6	157528077	157528155	DSCAML1	chr11	117374565	117374657	RICTOR	chr5	38958918	38958983
ARID1B	chr6	157528144	157528252	DSCAML1	chr11	117374646	117374750	RICTOR	chr5	38959165	38959275
ARID1B	chr6	157528241	157528327	DSCAML1	chr11	117375620	117375728	RICTOR	chr5	38959261	38959331
ARID1B	chr6	157528358	157528462	DSCAML1	chr11	117375717	117375803	RICTOR	chr5	38959377	38959492
ARID1B	chr6	157528430	157528555	DSCAML1	chr11	117376099	117376220	RICTOR	chr5	38959735	38959846
ARID1B	chr6	157528544	157528673	DSCAML1	chr11	117376180	117376314	RICTOR	chr5	38959872	38959951
ARID1B	chr6	157528662	157528748	DSCAML1	chr11	117376303	117376418	RICTOR	chr5	38959940	38960019
ARID1B	chr6	157528738	157528837	DSCAML1	chr11	117376406	117376537	RICTOR	chr5	38960008	38960094
ARID1B	chr6	157528864	157528967	DSCAML1	chr11	117387097	117387227	RICTOR	chr5	38960083	38960189
ARID1B	chr6	157528956	157529075	DSCAML1	chr11	117387200	117387295	RICTOR	chr5	38960397	38960515
ARID1B	chr6	157099035	157099138	DSCAML1	chr11	117387283	117387414	RICTOR	chr5	38960504	38960621
ARID1B	chr6	157099141	157099271	DSCAML1	chr11	117387403	117387537	RICTOR	chr5	38960610	38960712
ARID1B	chr6	157099271	157099381	DSCAML1	chr11	117389077	117389199	RICTOR	chr5	38962313	38962415
ARID1B	chr6	157099396	157099519	DSCAML1	chr11	117389185	117389305	RICTOR	chr5	38962433	38962543
ARID1B	chr6	157099827	157099954	DSCAML1	chr11	117389292	117389402	RICTOR	chr5	38962475	38962589
ARID1B	chr6	157100495	157100587	DSCAML1	chr11	117389361	117389449	RICTOR	chr5	38962658	38962762
ARID1B	chr6	157100501	157100596	DSCAML1	chr11	117389449	117389558	RICTOR	chr5	38962876	38962995
ARID1B	chr6	157150339	157150413	DSCAML1	chr11	117391757	117391892	RICTOR	chr5	38962984	38963103
ARID1B	chr6	157150409	157150501	DSCAML1	chr11	117391871	117391969	RICTOR	chr5	38963092	38963206
ARID1B	chr6	157150493	157150608	DSCAML1	chr11	117391956	117392073	RICTOR	chr5	38964796	38964908
ARID1B	chr6	157192763	157192882	DSCAML1	chr11	117392063	117392158	RICTOR	chr5	38964897	38964965
ARID1B	chr6	157222490	157222615	DSCAML1	chr11	117395412	117395545	RICTOR	chr5	38964954	38965044
ARID1B	chr6	157222584	157222688	DSCAML1	chr11	117395534	117395634	RICTOR	chr5	38966668	38966760
ARID1B	chr6	157256562	157256650	DSCAML1	chr11	117395621	117395756	RICTOR	chr5	38966749	38966817
ARID1B	chr6	157256635	157256707	DSCAML1	chr11	117395775	117395907	RICTOR	chr5	38967212	38967311
ARID1B	chr6	157256709	157256820	DSCAML1	chr11	117402958	117403087	RICTOR	chr5	38967307	38967422
ARID1B	chr6	157405699	157405827	DSCAML1	chr11	117403106	117403236	RICTOR	chr5	38967400	38967482
ARID1B	chr6	157405821	157405935	DSCAML1	chr11	117403205	117403299	RICTOR	chr5	38967471	38967579
ARID1B	chr6	157405927	157406014	DSCAML1	chr11	117647412	117647533	RICTOR	chr5	38967938	38968057
ARID1B	chr6	157406012	157406088	DSCAML1	chr11	117647522	117647634	RICTOR	chr5	38968046	38968130
ARID1B	chr6	157431587	157431712	DSCAML1	chr11	117647623	117647713	RICTOR	chr5	38968133	38968246
ARID1B	chr6	157454100	157454221	DSCAML1	chr11	117651157	117651285	RICTOR	chr5	38971902	38972022
ARID1B	chr6	157454221	157454346	DSCAML1	chr11	117651274	117651389	RICTOR	chr5	38975562	38975660
ARID1B	chr6	157469717	157469842	DSCAML1	chr11	117651378	117651492	RICTOR	chr5	38975649	38975764
ARID1B	chr6	157469842	157469936	DSCAML1	chr11	117651489	117651565	RICTOR	chr5	38978606	38978713
ARID1B	chr6	157469936	157470024	DSCAML1	chr11	117667771	117667900	RICTOR	chr5	38978702	38978802
ARID1B	chr6	157470024	157470113	EIF1AX	chrX	20146374	20146478	RICTOR	chr5	38981887	38982006
ARID1B	chr6	157488130	157488246	EIF1AX	chrX	20148593	20148706	RICTOR	chr5	38981995	38982091
ARID1B	chr6	157488246	157488378	EIF1AX	chrX	20148634	20148751	RICTOR	chr5	38982080	38982198

ARID1B	chr6	157495046	157495167	EIF1AX	chrX	20150246	20150364	RICTOR	chr5	38991051	38991123
ARID1B	chr6	157495167	157495294	EIF1AX	chrX	20150353	20150446	RICTOR	chr5	38991112	38991227
ARID1B	chr6	157502091	157502194	EIF1AX	chrX	20152043	20152162	RICTOR	chr5	38996904	38996969
ARID1B	chr6	157502194	157502322	EIF1AX	chrX	20153754	20153872	RICTOR	chr5	38996958	38997048
ARID1B	chr6	157505356	157505482	EIF1AX	chrX	20153861	20153969	RICTOR	chr5	39002585	39002687
ARID1B	chr6	157505476	157505586	EIF1AX	chrX	20156660	20156726	RICTOR	chr5	39002676	39002759
ARID1B	chr6	157510731	157510837	EIF1AX	chrX	20159681	20159787	RICTOR	chr5	39002748	39002831
ARID1B	chr6	157510833	157510920	KAT6A	chr8	41789664	41789770	RICTOR	chr5	39003588	39003674
ARID1B	chr6	157511101	157511208	KAT6A	chr8	41789752	41789833	RICTOR	chr5	39003663	39003743
ARID1B	chr6	157511208	157511324	KAT6A	chr8	41789818	41789931	RICTOR	chr5	39003732	39003849
ARID1B	chr6	157511322	157511402	KAT6A	chr8	41789927	41790041	RICTOR	chr5	39021018	39021138
ARID1B	chr6	157517243	157517369	KAT6A	chr8	41790038	41790154	RICTOR	chr5	39021095	39021207
ARID1B	chr6	157517369	157517474	KAT6A	chr8	41790118	41790248	RICTOR	chr5	39021196	39021302
ARID1B	chr6	157519854	157519965	KAT6A	chr8	41790247	41790376	RICTOR	chr5	39074501	39074634
ARID1B	chr6	157519965	157520080	KAT6A	chr8	41790376	41790492	SDHA	chr5	223451	223570
ARID1B	chr6	157521818	157521923	KAT6A	chr8	41790491	41790619	SDHA	chr5	223559	223649
ARID1B	chr6	157521909	157522005	KAT6A	chr8	41790803	41790941	SDHA	chr5	223638	223735
ARID1B	chr6	157522004	157522125	KAT6A	chr8	41790931	41791040	SDHA	chr5	224388	224494
ARID1B	chr6	157522123	157522223	KAT6A	chr8	41791036	41791127	SDHA	chr5	224483	224608
ARID1B	chr6	157522209	157522310	KAT6A	chr8	41791124	41791210	SDHA	chr5	224612	224739
ARID1B	chr6	157522310	157522431	KAT6A	chr8	41791206	41791328	SDHA	chr5	225434	225524
ARID1B	chr6	157522431	157522550	KAT6A	chr8	41791328	41791443	SDHA	chr5	225513	225630
ARID1B	chr6	157522550	157522642	KAT6A	chr8	41791443	41791570	SDHA	chr5	225604	225709
ARID1B	chr6	157524964	157525040	KAT6A	chr8	41791564	41791683	SDHA	chr5	225871	225983
ARID1B	chr6	157525040	157525148	KAT6A	chr8	41791679	41791806	SDHA	chr5	225972	226056
ARID1B	chr6	157527243	157527363	KAT6A	chr8	41791806	41791939	SDHA	chr5	226054	226183
ARID1B	chr6	157527355	157527469	KAT6A	chr8	41791820	41791954	SDHA	chr5	226159	226264
ARID1B	chr6	157527468	157527592	KAT6A	chr8	41791947	41792072	SDHA	chr5	228199	228320
ARID1B	chr6	157527571	157527660	KAT6A	chr8	41792010	41792139	SDHA	chr5	228309	228410
ARID1B	chr6	157527655	157527772	KAT6A	chr8	41792139	41792229	SDHA	chr5	228398	228516
ARID1B	chr6	157527772	157527900	KAT6A	chr8	41792229	41792326	SDHA	chr5	230895	231002
ARID1B	chr6	157527900	157528016	KAT6A	chr8	41792326	41792434	SDHA	chr5	230953	231072
ARID1B	chr6	157528011	157528141	KAT6A	chr8	41794759	41794847	SDHA	chr5	231107	231177
ARID1B	chr6	157528141	157528262	KAT6A	chr8	41794847	41794929	SDHA	chr5	233502	233615
ARID1B	chr6	157528262	157528393	KAT6A	chr8	41794929	41795013	SDHA	chr5	233605	233709
ARID1B	chr6	157528393	157528518	KAT6A	chr8	41795009	41795102	SDHA	chr5	233686	233809
ARID1B	chr6	157528518	157528636	KAT6A	chr8	41798300	41798377	SDHA	chr5	233791	233896
ARID1B	chr6	157528635	157528746	KAT6A	chr8	41798370	41798492	SDHA	chr5	235206	235334
ARID1B	chr6	157528746	157528872	KAT6A	chr8	41798486	41798585	SDHA	chr5	235295	235405
ARID1B	chr6	157528865	157528990	KAT6A	chr8	41798585	41798711	SDHA	chr5	235394	235510

ARID1B	chr6	157528990	157529082	KAT6A	chr8	41798709	41798826	SDHA	chr5	236456	236563
ARID2	chr12	46123587	46123706	KAT6A	chr8	41798826	41798941	SDHA	chr5	236561	236686
ARID2	chr12	46123685	46123820	KAT6A	chr8	41798910	41799020	SDHA	chr5	236675	236781
ARID2	chr12	46123829	46123914	KAT6A	chr8	41800315	41800441	SDHA	chr5	240397	240506
ARID2	chr12	46123903	46124025	KAT6A	chr8	41800441	41800565	SDHA	chr5	240495	240575
ARID2	chr12	46124871	46124991	KAT6A	chr8	41801269	41801346	SDHA	chr5	240564	240687
ARID2	chr12	46124980	46125057	KAT6A	chr8	41801346	41801436	SDHA	chr5	251056	251179
ARID2	chr12	46125046	46125152	KAT6A	chr8	41801434	41801516	SDHA	chr5	251168	251289
ARID2	chr12	46205144	46205238	KAT6A	chr8	41804077	41804202	SDHA	chr5	251359	251490
ARID2	chr12	46205227	46205298	KAT6A	chr8	41805201	41805326	SDHA	chr5	251449	251562
ARID2	chr12	46205287	46205385	KAT6A	chr8	41805325	41805446	SDHA	chr5	251538	251649
ARID2	chr12	46211393	46211476	KAT6A	chr8	41806708	41806791	SDHA	chr5	254403	254511
ARID2	chr12	46211475	46211563	KAT6A	chr8	41806789	41806872	SDHA	chr5	254500	254596
ARID2	chr12	46211552	46211630	KAT6A	chr8	41812803	41812885	SDHA	chr5	254585	254712
ARID2	chr12	46211619	46211724	KAT6A	chr8	41812863	41812960	SDHA	chr5	256360	256479
ARID2	chr12	46215132	46215235	KAT6A	chr8	41832163	41832258	SDHA	chr5	256468	256587
ARID2	chr12	46215224	46215291	KAT6A	chr8	41832253	41832353	SMAD4	chr18	48575099	48575213
ARID2	chr12	46230307	46230409	KAT6A	chr8	41834488	41834570	SMAD4	chr18	48575556	48575677
ARID2	chr12	46230398	46230513	KAT6A	chr8	41834570	41834670	SMAD4	chr18	48581190	48581302
ARID2	chr12	46230544	46230612	KAT6A	chr8	41834669	41834751	SMAD4	chr18	48584551	48584678
ARID2	chr12	46230601	46230724	KAT6A	chr8	41834751	41834824	SMAD4	chr18	48586251	48586361
ARID2	chr12	46230713	46230830	KAT6A	chr8	41836141	41836262	SMAD4	chr18	48591814	48591931
ARID2	chr12	46231022	46231096	KAT6A	chr8	41836246	41836324	SMAD4	chr18	48593399	48593519
ARID2	chr12	46231085	46231191	KAT6A	chr8	41838327	41838403	SMAD4	chr18	48603028	48603119
ARID2	chr12	46231180	46231264	KAT6A	chr8	41838403	41838483	SMAD4	chr18	48604658	48604774
ARID2	chr12	46231318	46231394	KAT6A	chr8	41839349	41839467	SRC	chr20	36012464	36012574
ARID2	chr12	46231383	46231476	KAT6A	chr8	41844947	41845075	SRC	chr20	36014396	36014525
ARID2	chr12	46231465	46231587	KAT6A	chr8	41905883	41906008	SRC	chr20	36014513	36014641
ARID2	chr12	46233052	46233158	KAT6A	chr8	41906008	41906124	SRC	chr20	36022231	36022331
ARID2	chr12	46233147	46233212	KAT6A	chr8	41906124	41906247	SRC	chr20	36022272	36022401
ARID2	chr12	46233201	46233282	KAT6A	chr8	41906247	41906349	SRC	chr20	36022400	36022498
ARID2	chr12	46233271	46233376	KAT6A	chr8	41906349	41906441	SRC	chr20	36022523	36022615
ARID2	chr12	46240555	46240665	KAT6A	chr8	41906441	41906515	SRC	chr20	36022601	36022700
ARID2	chr12	46240654	46240740	KAT6B	chr10	76602521	76602622	SRC	chr20	36022684	36022796
ARID2	chr12	46240729	46240848	KAT6B	chr10	76602622	76602735	SRC	chr20	36024482	36024608
ARID2	chr12	46242554	46242674	KAT6B	chr10	76602735	76602864	SRC	chr20	36024597	36024703
ARID2	chr12	46242663	46242777	KAT6B	chr10	76602864	76602984	SRC	chr20	36024685	36024779
ARID2	chr12	46242766	46242851	KAT6B	chr10	76602983	76603111	SRC	chr20	36026038	36026174
ARID2	chr12	46243297	46243418	KAT6B	chr10	76603111	76603237	SRC	chr20	36026112	36026234
ARID2	chr12	46243406	46243532	KAT6B	chr10	76719737	76719819	SRC	chr20	36026208	36026337

ARID2	chr12	46243521	46243639	KAT6B	chr10	76719765	76719868	SRC	chr20	36028403	36028512
ARID2	chr12	46243681	46243798	KAT6B	chr10	76729394	76729485	SRC	chr20	36028478	36028603
ARID2	chr12	46243787	46243862	KAT6B	chr10	76729450	76729568	SRC	chr20	36028560	36028666
ARID2	chr12	46243851	46243974	KAT6B	chr10	76729740	76729838	SRC	chr20	36028655	36028753
ARID2	chr12	46243963	46244079	KAT6B	chr10	76729837	76729923	SRC	chr20	36029906	36030033
ARID2	chr12	46244068	46244164	KAT6B	chr10	76732177	76732300	SRC	chr20	36030022	36030134
ARID2	chr12	46244153	46244272	KAT6B	chr10	76732286	76732384	SRC	chr20	36030732	36030865
ARID2	chr12	46244261	46244385	KAT6B	chr10	76732380	76732455	SRC	chr20	36030852	36030953
ARID2	chr12	46244374	46244492	KAT6B	chr10	76735158	76735231	SRC	chr20	36030935	36031059
ARID2	chr12	46244481	46244600	KAT6B	chr10	76735187	76735285	SRC	chr20	36031082	36031178
ARID2	chr12	46244589	46244701	KAT6B	chr10	76735285	76735374	SRC	chr20	36031162	36031276
ARID2	chr12	46244690	46244807	KAT6B	chr10	76735374	76735461	SRC	chr20	36031264	36031376
ARID2	chr12	46244796	46244924	KAT6B	chr10	76735461	76735542	SRC	chr20	36031425	36031558
ARID2	chr12	46244922	46245004	KAT6B	chr10	76735542	76735652	SRC	chr20	36031543	36031643
ARID2	chr12	46244993	46245078	KAT6B	chr10	76735630	76735743	SRC	chr20	36031676	36031795
ARID2	chr12	46245053	46245168	KAT6B	chr10	76735743	76735845	SRC	chr20	36031788	36031912
ARID2	chr12	46245157	46245278	KAT6B	chr10	76735845	76735961	TERT	chr5	1253784	1253893
ARID2	chr12	46245267	46245352	KAT6B	chr10	76735961	76736088	TERT	chr5	1253869	1254000
ARID2	chr12	46245341	46245459	KAT6B	chr10	76737009	76737123	TERT	chr5	1254400	1254519
ARID2	chr12	46245448	46245571	KAT6B	chr10	76737123	76737203	TERT	chr5	1254519	1254629
ARID2	chr12	46245560	46245684	KAT6B	chr10	76738934	76739045	TERT	chr5	1255346	1255472
ARID2	chr12	46245673	46245786	KAT6B	chr10	76739022	76739108	TERT	chr5	1255471	1255555
ARID2	chr12	46245775	46245899	KAT6B	chr10	76741566	76741666	TERT	chr5	1258616	1258741
ARID2	chr12	46245888	46246001	KAT6B	chr10	76741666	76741744	TERT	chr5	1258741	1258848
ARID2	chr12	46245990	46246083	KAT6B	chr10	76744795	76744879	TERT	chr5	1260476	1260599
ARID2	chr12	46246072	46246193	KAT6B	chr10	76744861	76744963	TERT	chr5	1260599	1260683
ARID2	chr12	46246182	46246306	KAT6B	chr10	76744941	76745051	TERT	chr5	1260681	1260771
ARID2	chr12	46246295	46246399	KAT6B	chr10	76748765	76748886	TERT	chr5	1264409	1264538
ARID2	chr12	46246388	46246506	KAT6B	chr10	76780306	76780424	TERT	chr5	1264538	1264653
ARID2	chr12	46246495	46246625	KAT6B	chr10	76780424	76780539	TERT	chr5	1264653	1264761
ARID2	chr12	46246614	46246729	KAT6B	chr10	76780535	76780653	TERT	chr5	1266445	1266553
ARID2	chr12	46254563	46254644	KAT6B	chr10	76780826	76780940	TERT	chr5	1266539	1266624
ARID2	chr12	46254619	46254700	KAT6B	chr10	76780940	76781028	TERT	chr5	1266624	1266707
ARID2	chr12	46254689	46254810	KAT6B	chr10	76781576	76781698	TERT	chr5	1268546	1268670
ARID2	chr12	46285475	46285598	KAT6B	chr10	76781698	76781803	TERT	chr5	1268670	1268801
ARID2	chr12	46285587	46285677	KAT6B	chr10	76781817	76781946	TERT	chr5	1271229	1271323
ARID2	chr12	46285666	46285751	KAT6B	chr10	76781831	76781956	TERT	chr5	1271321	1271403
ARID2	chr12	46285811	46285929	KAT6B	chr10	76784704	76784822	TERT	chr5	1272239	1272371
ARID2	chr12	46287054	46287178	KAT6B	chr10	76784822	76784925	TERT	chr5	1272371	1272455
ARID2	chr12	46287167	46287290	KAT6B	chr10	76784925	76785037	TERT	chr5	1278699	1278828

ARID2	chr12	46287279	46287400	KAT6B	chr10	76788209	76788337	TERT	chr5	1278827	1278962
ARID2	chr12	46287360	46287484	KAT6B	chr10	76788337	76788463	TERT	chr5	1279338	1279444
ARID2	chr12	46287473	46287585	KAT6B	chr10	76788463	76788590	TERT	chr5	1279424	1279549
ARID2	chr12	46298628	46298725	KAT6B	chr10	76788590	76788716	TERT	chr5	1279545	1279643
ARID2	chr12	46298708	46298813	KAT6B	chr10	76788640	76788756	TERT	chr5	1280210	1280312
ARID2	chr12	46298802	46298911	KAT6B	chr10	76788755	76788870	TERT	chr5	1280311	1280446
ARID2	chr12	46123588	46123691	KAT6B	chr10	76788866	76788994	TERT	chr5	1280446	1280536
ARID2	chr12	46123684	46123795	KAT6B	chr10	76788987	76789116	TERT	chr5	1282486	1282591
ARID2	chr12	46123803	46123889	KAT6B	chr10	76789096	76789214	TERT	chr5	1282591	1282683
ARID2	chr12	46123878	46123963	KAT6B	chr10	76789207	76789340	TERT	chr5	1282683	1282807
ARID2	chr12	46124981	46125072	KAT6B	chr10	76789338	76789465	TERT	chr5	1293371	1293486
ARID2	chr12	46125072	46125175	KAT6B	chr10	76789460	76789582	TERT	chr5	1293485	1293615
ARID2	chr12	46205142	46205267	KAT6B	chr10	76789582	76789711	TERT	chr5	1293626	1293722
ARID2	chr12	46205267	46205343	KAT6B	chr10	76789708	76789842	TERT	chr5	1293686	1293820
ARID2	chr12	46211392	46211510	KAT6B	chr10	76789839	76789964	TERT	chr5	1293814	1293912
ARID2	chr12	46211506	46211596	KAT6B	chr10	76789964	76790059	TERT	chr5	1293912	1294013
ARID2	chr12	46211594	46211663	KAT6B	chr10	76790059	76790160	TERT	chr5	1294010	1294143
ARID2	chr12	46215129	46215225	KAT6B	chr10	76790159	76790283	TERT	chr5	1294062	1294187
ARID2	chr12	46215229	46215305	KAT6B	chr10	76790283	76790408	TERT	chr5	1294186	1294306
ARID2	chr12	46230407	46230516	KAT6B	chr10	76790408	76790541	TERT	chr5	1294387	1294489
ARID2	chr12	46230423	46230545	KAT6B	chr10	76790541	76790637	TERT	chr5	1294413	1294550
ARID2	chr12	46230633	46230756	KAT6B	chr10	76790637	76790758	TERT	chr5	1294628	1294735
ARID2	chr12	46230756	46230832	KAT6B	chr10	76790758	76790844	TERT	chr5	1294629	1294767
ARID2	chr12	46231077	46231189	KMT2A	chr11	118307486	118307621	TP53	chr17	7572831	7572936
ARID2	chr12	46231127	46231197	KMT2A	chr11	118307530	118307660	TP53	chr17	7572937	7573040
ARID2	chr12	46231329	46231402	KMT2A	chr11	118339430	118339560	TP53	chr17	7573863	7573967
ARID2	chr12	46231399	46231473	KMT2A	chr11	118342300	118342403	TP53	chr17	7573944	7574041
ARID2	chr12	46231469	46231589	KMT2A	chr11	118342399	118342477	TP53	chr17	7576605	7576690
ARID2	chr12	46233062	46233176	KMT2A	chr11	118342475	118342578	TP53	chr17	7576837	7576950
ARID2	chr12	46233176	46233296	KMT2A	chr11	118342578	118342695	TP53	chr17	7576958	7577064
ARID2	chr12	46240568	46240665	KMT2A	chr11	118342684	118342790	TP53	chr17	7577065	7577165
ARID2	chr12	46240656	46240746	KMT2A	chr11	118342788	118342916	TP53	chr17	7577487	7577618
ARID2	chr12	46242555	46242681	KMT2A	chr11	118342907	118342991	TP53	chr17	7578142	7578234
ARID2	chr12	46242681	46242780	KMT2A	chr11	118342991	118343087	TP53	chr17	7578235	7578362
ARID2	chr12	46243279	46243399	KMT2A	chr11	118343087	118343170	TP53	chr17	7578305	7578432
ARID2	chr12	46243398	46243520	KMT2A	chr11	118343170	118343291	TP53	chr17	7578433	7578564
ARID2	chr12	46243520	46243641	KMT2A	chr11	118343291	118343401	TP53	chr17	7579279	7579385
ARID2	chr12	46243786	46243899	KMT2A	chr11	118343401	118343536	TP53	chr17	7579386	7579502
ARID2	chr12	46243899	46244018	KMT2A	chr11	118343507	118343619	TP53	chr17	7579503	7579588
ARID2	chr12	46244018	46244144	KMT2A	chr11	118343619	118343739	TP53	chr17	7579570	7579698

ARID2	chr12	46244144	46244226
ARID2	chr12	46244226	46244350
ARID2	chr12	46244350	46244467
ARID2	chr12	46244467	46244562
ARID2	chr12	46244562	46244690
ARID2	chr12	46244690	46244793
ARID2	chr12	46244793	46244898
ARID2	chr12	46244898	46245025
ARID2	chr12	46245025	46245139
ARID2	chr12	46245139	46245266
ARID2	chr12	46245266	46245389
ARID2	chr12	46245389	46245508
ARID2	chr12	46245508	46245626
ARID2	chr12	46245626	46245720
ARID2	chr12	46245718	46245814
ARID2	chr12	46245814	46245923
ARID2	chr12	46245922	46246044
ARID2	chr12	46246032	46246117
ARID2	chr12	46246117	46246197
ARID2	chr12	46246197	46246292
ARID2	chr12	46246292	46246403
ARID2	chr12	46246403	46246509
ARID2	chr12	46246508	46246611
ARID2	chr12	46246611	46246687
ARID2	chr12	46254567	46254645
ARID2	chr12	46254642	46254717
ARID2	chr12	46254707	46254774
ARID2	chr12	46285552	46285621
ARID2	chr12	46285621	46285733
ARID2	chr12	46285673	46285805
ARID2	chr12	46285805	46285922
ARID2	chr12	46287142	46287245
ARID2	chr12	46287245	46287332
ARID2	chr12	46287352	46287475
ARID2	chr12	46287467	46287578
ARID2	chr12	46298693	46298780
ARID2	chr12	46298772	46298888
ATRX	chrX	76763770	76763879
ATRX	chrX	76763876	76763960
ATRX	chrX	76763960	76764047
KMT2A	chr11	118343739	118343839
KMT2A	chr11	118343839	118343922
KMT2A	chr11	118343922	118344038
KMT2A	chr11	118344038	118344134
KMT2A	chr11	118344134	118344225
KMT2A	chr11	118344225	118344344
KMT2A	chr11	118344340	118344455
KMT2A	chr11	118344455	118344568
KMT2A	chr11	118344477	118344607
KMT2A	chr11	118344607	118344705
KMT2A	chr11	118344705	118344815
KMT2A	chr11	118344810	118344930
KMT2A	chr11	118344929	118345037
KMT2A	chr11	118347512	118347617
KMT2A	chr11	118347616	118347717
KMT2A	chr11	118348657	118348788
KMT2A	chr11	118348788	118348917
KMT2A	chr11	118350831	118350951
KMT2A	chr11	118350951	118351047
KMT2A	chr11	118352418	118352531
KMT2A	chr11	118352528	118352636
KMT2A	chr11	118352624	118352738
KMT2A	chr11	118352735	118352857
KMT2A	chr11	118353098	118353218
KMT2A	chr11	118354859	118354957
KMT2A	chr11	118354957	118355080
KMT2A	chr11	118355572	118355690
KMT2A	chr11	118359246	118359367
KMT2A	chr11	118359367	118359478
KMT2A	chr11	118360420	118360531
KMT2A	chr11	118360529	118360635
KMT2A	chr11	118360747	118360860
KMT2A	chr11	118360860	118360978
KMT2A	chr11	118361860	118361950
KMT2A	chr11	118361948	118362026
KMT2A	chr11	118362450	118362572
KMT2A	chr11	118362572	118362664
KMT2A	chr11	118363738	118363856
KMT2A	chr11	118363856	118363945
KMT2A	chr11	118364967	118365094
TP53	chr17	7579699	7579822
TP53	chr17	7579835	7579949

ATRX	chrX	76764047	76764131	KMT2A	chr11	118365110	118365200
ATRX	chrX	76776257	76776348	KMT2A	chr11	118365310	118365423
ATRX	chrX	76776348	76776416	KMT2A	chr11	118365423	118365509
ATRX	chrX	76776849	76776937	KMT2A	chr11	118366319	118366425
ATRX	chrX	76776937	76777027	KMT2A	chr11	118366425	118366517
ATRX	chrX	76777678	76777798	KMT2A	chr11	118366517	118366608
ATRX	chrX	76777798	76777883	KMT2A	chr11	118366958	118367083
ATRX	chrX	76778663	76778759	KMT2A	chr11	118368578	118368694
ATRX	chrX	76778758	76778870	KMT2A	chr11	118368694	118368809
ATRX	chrX	76778869	76778938	KMT2A	chr11	118369027	118369154
ATRX	chrX	76812899	76813013	KMT2A	chr11	118369153	118369235
ATRX	chrX	76812996	76813101	KMT2A	chr11	118369924	118370019
ATRX	chrX	76813100	76813169	KMT2A	chr11	118370019	118370136
ATRX	chrX	76814058	76814157	KMT2A	chr11	118370530	118370630
ATRX	chrX	76814145	76814211	KMT2A	chr11	118371625	118371750
ATRX	chrX	76814241	76814311	KMT2A	chr11	118371750	118371862
ATRX	chrX	76829629	76829754	KMT2A	chr11	118372289	118372391
ATRX	chrX	76829754	76829859	KMT2A	chr11	118372391	118372462
ATRX	chrX	76845217	76845330	KMT2A	chr11	118372462	118372588
ATRX	chrX	76845307	76845386	KMT2A	chr11	118373077	118373197
ATRX	chrX	76849104	76849218	KMT2A	chr11	118373194	118373317
ATRX	chrX	76849187	76849260	KMT2A	chr11	118373305	118373404
ATRX	chrX	76849260	76849354	KMT2A	chr11	118373403	118373524
ATRX	chrX	76854869	76854968	KMT2A	chr11	118373524	118373641
ATRX	chrX	76854964	76855084	KMT2A	chr11	118373641	118373742
ATRX	chrX	76855186	76855260	KMT2A	chr11	118373742	118373866
ATRX	chrX	76855258	76855360	KMT2A	chr11	118373866	118373976
ATRX	chrX	76855876	76855973	KMT2A	chr11	118373976	118374062
ATRX	chrX	76855958	76856041	KMT2A	chr11	118374062	118374184
ATRX	chrX	76872040	76872126	KMT2A	chr11	118374183	118374304
ATRX	chrX	76872126	76872241	KMT2A	chr11	118374304	118374404
ATRX	chrX	76874226	76874317	KMT2A	chr11	118374404	118374512
ATRX	chrX	76874310	76874384	KMT2A	chr11	118374502	118374610
ATRX	chrX	76874380	76874458	KMT2A	chr11	118374607	118374699
ATRX	chrX	76875749	76875867	KMT2A	chr11	118374699	118374775
ATRX	chrX	76875867	76875962	KMT2A	chr11	118374775	118374873
ATRX	chrX	76875961	76876078	KMT2A	chr11	118374856	118374974
ATRX	chrX	76888681	76888789	KMT2A	chr11	118374974	118375073
ATRX	chrX	76888765	76888842	KMT2A	chr11	118375073	118375171
ATRX	chrX	76888842	76888931	KMT2A	chr11	118375171	118375265

ATRX	chrX	76889063	76889138	KMT2A	chr11	118375265	118375383
ATRX	chrX	76889112	76889195	KMT2A	chr11	118375383	118375510
ATRX	chrX	76889179	76889285	KMT2A	chr11	118375510	118375635
ATRX	chrX	76890081	76890157	KMT2A	chr11	118375632	118375762
ATRX	chrX	76890153	76890241	KMT2A	chr11	118375762	118375892
ATRX	chrX	76891357	76891459	KMT2A	chr11	118375891	118376020
ATRX	chrX	76891420	76891494	KMT2A	chr11	118376020	118376127
ATRX	chrX	76907625	76907710	KMT2A	chr11	118376127	118376252
ATRX	chrX	76907643	76907731	KMT2A	chr11	118376252	118376369
ATRX	chrX	76909521	76909637	KMT2A	chr11	118376369	118376483
ATRX	chrX	76909637	76909756	KMT2A	chr11	118376481	118376582
ATRX	chrX	76912003	76912084	KMT2A	chr11	118376582	118376677
ATRX	chrX	76912084	76912204	KMT2A	chr11	118376677	118376752
ATRX	chrX	76918896	76918971	KMT2A	chr11	118376752	118376835
ATRX	chrX	76918971	76919069	KMT2A	chr11	118376835	118376946
ATRX	chrX	76920080	76920185	KMT2A	chr11	118376943	118377068
ATRX	chrX	76920162	76920242	KMT2A	chr11	118377067	118377155
ATRX	chrX	76931631	76931760	KMT2A	chr11	118377134	118377264
ATRX	chrX	76931760	76931885	KMT2A	chr11	118377264	118377362
ATRX	chrX	76936934	76937037	KMT2A	chr11	118378227	118378351
ATRX	chrX	76937037	76937127	KMT2A	chr11	118379793	118379914
ATRX	chrX	76937116	76937199	KMT2A	chr11	118380645	118380729
ATRX	chrX	76937197	76937283	KMT2A	chr11	118380729	118380833
ATRX	chrX	76937283	76937374	KMT2A	chr11	118382619	118382740
ATRX	chrX	76937366	76937461	KMT2A	chr11	118390293	118390421
ATRX	chrX	76937458	76937546	KMT2A	chr11	118390421	118390512
ATRX	chrX	76937542	76937613	KMT2A	chr11	118390585	118390673
ATRX	chrX	76937689	76937804	KMT2A	chr11	118390673	118390779
ATRX	chrX	76937804	76937884	KMT2A	chr11	118391419	118391525
ATRX	chrX	76937878	76937993	KMT2A	chr11	118391525	118391646
ATRX	chrX	76937992	76938118	KMT2A	chr11	118391932	118392054
ATRX	chrX	76938118	76938187	KMT2A	chr11	118392054	118392142
ATRX	chrX	76938368	76938491	KMT2A	chr11	118392607	118392732
ATRX	chrX	76938462	76938534	KMT2A	chr11	118392732	118392816
ATRX	chrX	76938534	76938608	KMT2A	chr11	118392812	118392931
ATRX	chrX	76938593	76938663	KMT2D	chr12	49415521	49415641
ATRX	chrX	76938657	76938770	KMT2D	chr12	49415630	49415739
ATRX	chrX	76938770	76938860	KMT2D	chr12	49415756	49415878
ATRX	chrX	76938860	76938953	KMT2D	chr12	49415868	49415957
ATRX	chrX	76938953	76939063	KMT2D	chr12	49416037	49416164

ATRX	chrX	76939038	76939133	KMT2D	chr12	49416340	49416444
ATRX	chrX	76939130	76939203	KMT2D	chr12	49416433	49416546
ATRX	chrX	76939194	76939301	KMT2D	chr12	49416534	49416631
ATRX	chrX	76939301	76939375	KMT2D	chr12	49416622	49416703
ATRX	chrX	76939372	76939467	KMT2D	chr12	49418271	49418378
ATRX	chrX	76939459	76939528	KMT2D	chr12	49418373	49418501
ATRX	chrX	76939522	76939594	KMT2D	chr12	49418555	49418674
ATRX	chrX	76939594	76939675	KMT2D	chr12	49418664	49418762
ATRX	chrX	76939671	76939741	KMT2D	chr12	49419919	49420039
ATRX	chrX	76939740	76939855	KMT2D	chr12	49420028	49420141
ATRX	chrX	76939822	76939896	KMT2D	chr12	49420108	49420214
ATRX	chrX	76939995	76940106	KMT2D	chr12	49420265	49420392
ATRX	chrX	76940407	76940492	KMT2D	chr12	49420381	49420502
ATRX	chrX	76944341	76944419	KMT2D	chr12	49420522	49420637
ATRX	chrX	76944419	76944496	KMT2D	chr12	49420568	49420700
ATRX	chrX	76949261	76949373	KMT2D	chr12	49420684	49420807
ATRX	chrX	76949373	76949463	KMT2D	chr12	49420806	49420926
ATRX	chrX	76951959	76952068	KMT2D	chr12	49420882	49421005
ATRX	chrX	76952138	76952223	KMT2D	chr12	49420962	49421064
ATRX	chrX	76952994	76953089	KMT2D	chr12	49421059	49421139
ATRX	chrX	76953078	76953152	KMT2D	chr12	49421489	49421606
ATRX	chrX	76954001	76954079	KMT2D	chr12	49421595	49421723
ATRX	chrX	76954079	76954172	KMT2D	chr12	49421750	49421874
ATRX	chrX	76972599	76972678	KMT2D	chr12	49421864	49421943
ATRX	chrX	76972678	76972781	KMT2D	chr12	49422530	49422657
ATRX	chrX	77041367	77041475	KMT2D	chr12	49422646	49422751
ATRX	chrX	77041475	77041555	KMT2D	chr12	49422738	49422835
CSMD3	chr8	113236965	113237070	KMT2D	chr12	49422849	49422963
CSMD3	chr8	113237070	113237195	KMT2D	chr12	49422935	49423029
CSMD3	chr8	113240929	113241038	KMT2D	chr12	49423118	49423231
CSMD3	chr8	113241066	113241169	KMT2D	chr12	49423208	49423306
CSMD3	chr8	113243750	113243822	KMT2D	chr12	49424024	49424151
CSMD3	chr8	113243815	113243893	KMT2D	chr12	49424150	49424241
CSMD3	chr8	113246544	113246633	KMT2D	chr12	49424274	49424400
CSMD3	chr8	113246633	113246714	KMT2D	chr12	49424389	49424493
CSMD3	chr8	113249405	113249518	KMT2D	chr12	49424473	49424561
CSMD3	chr8	113249518	113249590	KMT2D	chr12	49424607	49424732
CSMD3	chr8	113253972	113254102	KMT2D	chr12	49424697	49424813
CSMD3	chr8	113256585	113256661	KMT2D	chr12	49424802	49424913
CSMD3	chr8	113256640	113256723	KMT2D	chr12	49424943	49425059

CSMD3	chr8	113256722	113256794	KMT2D	chr12	49424963	49425089
CSMD3	chr8	113256792	113256868	KMT2D	chr12	49425108	49425240
CSMD3	chr8	113259192	113259277	KMT2D	chr12	49425235	49425358
CSMD3	chr8	113259276	113259397	KMT2D	chr12	49425384	49425511
CSMD3	chr8	113266436	113266541	KMT2D	chr12	49425406	49425534
CSMD3	chr8	113266541	113266638	KMT2D	chr12	49425598	49425696
CSMD3	chr8	113267438	113267531	KMT2D	chr12	49425790	49425895
CSMD3	chr8	113267531	113267613	KMT2D	chr12	49425884	49425980
CSMD3	chr8	113267613	113267710	KMT2D	chr12	49425970	49426062
CSMD3	chr8	113275805	113275931	KMT2D	chr12	49426046	49426178
CSMD3	chr8	113275926	113276049	KMT2D	chr12	49426177	49426304
CSMD3	chr8	113277648	113277746	KMT2D	chr12	49426295	49426406
CSMD3	chr8	113277746	113277829	KMT2D	chr12	49426391	49426514
CSMD3	chr8	113293304	113293431	KMT2D	chr12	49426536	49426622
CSMD3	chr8	113293431	113293507	KMT2D	chr12	49426610	49426693
CSMD3	chr8	113293506	113293573	KMT2D	chr12	49426676	49426781
CSMD3	chr8	113299258	113299347	KMT2D	chr12	49426808	49426936
CSMD3	chr8	113299346	113299469	KMT2D	chr12	49426870	49427001
CSMD3	chr8	113299469	113299555	KMT2D	chr12	49427071	49427192
CSMD3	chr8	113301478	113301591	KMT2D	chr12	49427196	49427286
CSMD3	chr8	113301591	113301707	KMT2D	chr12	49427249	49427364
CSMD3	chr8	113301707	113301807	KMT2D	chr12	49427353	49427480
CSMD3	chr8	113303701	113303812	KMT2D	chr12	49427469	49427585
CSMD3	chr8	113303768	113303840	KMT2D	chr12	49427604	49427734
CSMD3	chr8	113304720	113304839	KMT2D	chr12	49427723	49427847
CSMD3	chr8	113304839	113304958	KMT2D	chr12	49427871	49427984
CSMD3	chr8	113308019	113308095	KMT2D	chr12	49427916	49428039
CSMD3	chr8	113308095	113308218	KMT2D	chr12	49428157	49428287
CSMD3	chr8	113308218	113308299	KMT2D	chr12	49428281	49428382
CSMD3	chr8	113313978	113314065	KMT2D	chr12	49428364	49428460
CSMD3	chr8	113314065	113314144	KMT2D	chr12	49428565	49428670
CSMD3	chr8	113314144	113314229	KMT2D	chr12	49428659	49428756
CSMD3	chr8	113316911	113317034	KMT2D	chr12	49430869	49430993
CSMD3	chr8	113317032	113317149	KMT2D	chr12	49430981	49431087
CSMD3	chr8	113318150	113318272	KMT2D	chr12	49431076	49431197
CSMD3	chr8	113318272	113318364	KMT2D	chr12	49431192	49431324
CSMD3	chr8	113318364	113318477	KMT2D	chr12	49431292	49431415
CSMD3	chr8	113323125	113323250	KMT2D	chr12	49431397	49431494
CSMD3	chr8	113323250	113323357	KMT2D	chr12	49431483	49431612
CSMD3	chr8	113323357	113323429	KMT2D	chr12	49431601	49431723

CSMD3	chr8	113326119	113326243	KMT2D	chr12	49431681	49431813
CSMD3	chr8	113326253	113326378	KMT2D	chr12	49431804	49431932
CSMD3	chr8	113326579	113326671	KMT2D	chr12	49431921	49432041
CSMD3	chr8	113326671	113326763	KMT2D	chr12	49432030	49432136
CSMD3	chr8	113326763	113326868	KMT2D	chr12	49432125	49432244
CSMD3	chr8	113330969	113331084	KMT2D	chr12	49432187	49432297
CSMD3	chr8	113331079	113331185	KMT2D	chr12	49432286	49432401
CSMD3	chr8	113332109	113332229	KMT2D	chr12	49432368	49432504
CSMD3	chr8	113347547	113347662	KMT2D	chr12	49432493	49432623
CSMD3	chr8	113347657	113347738	KMT2D	chr12	49432622	49432740
CSMD3	chr8	113348808	113348935	KMT2D	chr12	49432729	49432807
CSMD3	chr8	113348933	113349024	KMT2D	chr12	49432943	49433061
CSMD3	chr8	113348980	113349099	KMT2D	chr12	49433029	49433151
CSMD3	chr8	113349755	113349847	KMT2D	chr12	49433144	49433243
CSMD3	chr8	113349847	113349954	KMT2D	chr12	49433232	49433359
CSMD3	chr8	113349954	113350043	KMT2D	chr12	49433378	49433482
CSMD3	chr8	113353729	113353810	KMT2D	chr12	49433437	49433516
CSMD3	chr8	113353806	113353893	KMT2D	chr12	49433601	49433705
CSMD3	chr8	113353872	113353948	KMT2D	chr12	49433664	49433797
CSMD3	chr8	113358281	113358383	KMT2D	chr12	49433797	49433906
CSMD3	chr8	113358379	113358452	KMT2D	chr12	49433874	49433967
CSMD3	chr8	113363333	113363434	KMT2D	chr12	49433957	49434088
CSMD3	chr8	113363451	113363554	KMT2D	chr12	49434007	49434109
CSMD3	chr8	113364629	113364729	KMT2D	chr12	49434212	49434326
CSMD3	chr8	113364729	113364794	KMT2D	chr12	49434323	49434457
CSMD3	chr8	113392565	113392659	KMT2D	chr12	49434454	49434586
CSMD3	chr8	113392626	113392702	KMT2D	chr12	49434586	49434682
CSMD3	chr8	113395735	113395851	KMT2D	chr12	49434671	49434782
CSMD3	chr8	113395851	113395936	KMT2D	chr12	49435045	49435168
CSMD3	chr8	113402807	113402927	KMT2D	chr12	49435150	49435282
CSMD3	chr8	113402927	113403023	KMT2D	chr12	49435276	49435398
CSMD3	chr8	113418754	113418856	KMT2D	chr12	49435405	49435494
CSMD3	chr8	113418810	113418885	KMT2D	chr12	49435488	49435569
CSMD3	chr8	113420501	113420568	KMT2D	chr12	49435679	49435788
CSMD3	chr8	113420549	113420641	KMT2D	chr12	49435768	49435893
CSMD3	chr8	113420641	113420718	KMT2D	chr12	49435882	49435983
CSMD3	chr8	113421136	113421267	KMT2D	chr12	49436225	49436339
CSMD3	chr8	113484776	113484875	KMT2D	chr12	49436338	49436439
CSMD3	chr8	113484833	113484924	KMT2D	chr12	49436494	49436611
CSMD3	chr8	113504613	113504741	KMT2D	chr12	49436604	49436683

CSMD3	chr8	113504741	113504839	KMT2D	chr12	49436781	49436883
CSMD3	chr8	113504826	113504905	KMT2D	chr12	49436872	49436980
CSMD3	chr8	113515966	113516088	KMT2D	chr12	49437076	49437176
CSMD3	chr8	113516088	113516174	KMT2D	chr12	49437162	49437245
CSMD3	chr8	113516169	113516242	KMT2D	chr12	49437378	49437507
CSMD3	chr8	113518879	113519001	KMT2D	chr12	49437495	49437578
CSMD3	chr8	113518999	113519123	KMT2D	chr12	49437565	49437667
CSMD3	chr8	113529228	113529311	KMT2D	chr12	49437661	49437768
CSMD3	chr8	113529310	113529396	KMT2D	chr12	49437726	49437830
CSMD3	chr8	113529391	113529464	KMT2D	chr12	49437938	49438068
CSMD3	chr8	113562879	113562964	KMT2D	chr12	49438057	49438190
CSMD3	chr8	113562964	113563047	KMT2D	chr12	49438105	49438232
CSMD3	chr8	113564785	113564873	KMT2D	chr12	49438432	49438562
CSMD3	chr8	113564873	113564972	KMT2D	chr12	49438547	49438679
CSMD3	chr8	113568913	113568993	KMT2D	chr12	49438678	49438758
CSMD3	chr8	113568993	113569065	KMT2D	chr12	49439612	49439742
CSMD3	chr8	113569046	113569169	KMT2D	chr12	49439722	49439840
CSMD3	chr8	113585711	113585834	KMT2D	chr12	49439861	49439970
CSMD3	chr8	113585833	113585917	KMT2D	chr12	49440023	49440131
CSMD3	chr8	113599288	113599396	KMT2D	chr12	49440104	49440217
CSMD3	chr8	113599313	113599413	KMT2D	chr12	49440333	49440462
CSMD3	chr8	113648987	113649107	KMT2D	chr12	49440457	49440583
CSMD3	chr8	113649107	113649195	KMT2D	chr12	49441642	49441768
CSMD3	chr8	113649195	113649266	KMT2D	chr12	49441758	49441862
CSMD3	chr8	113650878	113650968	KMT2D	chr12	49442345	49442451
CSMD3	chr8	113650993	113651083	KMT2D	chr12	49442440	49442562
CSMD3	chr8	113651083	113651168	KMT2D	chr12	49442777	49442903
CSMD3	chr8	113657287	113657381	KMT2D	chr12	49442892	49443017
CSMD3	chr8	113657381	113657461	KMT2D	chr12	49443422	49443549
CSMD3	chr8	113662362	113662453	KMT2D	chr12	49443538	49443620
CSMD3	chr8	113662429	113662536	KMT2D	chr12	49443596	49443725
CSMD3	chr8	113662533	113662616	KMT2D	chr12	49443716	49443803
CSMD3	chr8	113668357	113668453	KMT2D	chr12	49443789	49443917
CSMD3	chr8	113668438	113668538	KMT2D	chr12	49443943	49444022
CSMD3	chr8	113668538	113668610	KMT2D	chr12	49443999	49444127
CSMD3	chr8	113678417	113678536	KMT2D	chr12	49444126	49444217
CSMD3	chr8	113678536	113678624	KMT2D	chr12	49444207	49444317
CSMD3	chr8	113678607	113678710	KMT2D	chr12	49444303	49444395
CSMD3	chr8	113694604	113694689	KMT2D	chr12	49444470	49444588
CSMD3	chr8	113694689	113694809	KMT2D	chr12	49444650	49444775

CSMD3	chr8	113694828	113694905	KMT2D	chr12	49444693	49444828
CSMD3	chr8	113697531	113697655	KMT2D	chr12	49444928	49445045
CSMD3	chr8	113697659	113697755	KMT2D	chr12	49445026	49445113
CSMD3	chr8	113697748	113697832	KMT2D	chr12	49445090	49445176
CSMD3	chr8	113697831	113697904	KMT2D	chr12	49445145	49445263
CSMD3	chr8	113697882	113697963	KMT2D	chr12	49445293	49445402
CSMD3	chr8	113702090	113702194	KMT2D	chr12	49445334	49445452
CSMD3	chr8	113702194	113702310	KMT2D	chr12	49445468	49445561
CSMD3	chr8	113812296	113812417	KMT2D	chr12	49445552	49445669
CSMD3	chr8	113812417	113812508	KMT2D	chr12	49445660	49445754
CSMD3	chr8	113841846	113841967	KMT2D	chr12	49445714	49445803
CSMD3	chr8	113841967	113842081	KMT2D	chr12	49445793	49445921
CSMD3	chr8	113871327	113871449	KMT2D	chr12	49445910	49446044
CSMD3	chr8	113871449	113871522	KMT2D	chr12	49446033	49446145
CSMD3	chr8	113933767	113933870	KMT2D	chr12	49446111	49446217
CSMD3	chr8	113933853	113933945	KMT2D	chr12	49446249	49446377
CSMD3	chr8	113933945	113934029	KMT2D	chr12	49446366	49446483
CSMD3	chr8	113959937	113960063	KMT2D	chr12	49446659	49446789
CSMD3	chr8	113966943	113967066	KMT2D	chr12	49446782	49446872
CSMD3	chr8	113988046	113988127	KMT2D	chr12	49446895	49447014
CSMD3	chr8	113988127	113988239	KMT2D	chr12	49447003	49447114
CSMD3	chr8	113988239	113988351	KMT2D	chr12	49447240	49447368
CSMD3	chr8	113988351	113988476	KMT2D	chr12	49447357	49447441
CSMD3	chr8	114031168	114031291	KMT2D	chr12	49447722	49447850
CSMD3	chr8	114031291	114031364	KMT2D	chr12	49447839	49447944
CSMD3	chr8	114031364	114031449	KMT2D	chr12	49447988	49448114
CSMD3	chr8	114110962	114111084	KMT2D	chr12	49448107	49448227
CSMD3	chr8	114111083	114111165	KMT2D	chr12	49448216	49448345
CSMD3	chr8	114111164	114111285	KMT2D	chr12	49448326	49448438
CSMD3	chr8	114185925	114186041	KMT2D	chr12	49448459	49448540
CSMD3	chr8	114186039	114186121	KMT2D	chr12	49448517	49448612
CSMD3	chr8	114186121	114186237	KMT2D	chr12	49448633	49448719
CSMD3	chr8	114290797	114290867	KMT2D	chr12	49448707	49448822
CSMD3	chr8	114290864	114290932	KMT2D	chr12	49449023	49449145
CSMD3	chr8	114326780	114326900	LRP1B	chr2	140990697	140990818
CSMD3	chr8	114326833	114326954	LRP1B	chr2	140990811	140990928
CSMD3	chr8	114326943	114327019	LRP1B	chr2	140992318	140992439
CSMD3	chr8	114388873	114388991	LRP1B	chr2	140992428	140992539
CSMD3	chr8	114388988	114389083	LRP1B	chr2	140995689	140995806
CSMD3	chr8	114448856	114448984	LRP1B	chr2	140996978	140997092

CSMD3	chr8	114448983	114449098	LRP1B	chr2	140997092	140997205
DAXX	chr6	33286486	33286609	LRP1B	chr2	141004578	141004696
DAXX	chr6	33286760	33286892	LRP1B	chr2	141004685	141004761
DAXX	chr6	33286887	33287005	LRP1B	chr2	141027774	141027878
DAXX	chr6	33287116	33287242	LRP1B	chr2	141027867	141027942
DAXX	chr6	33287240	33287337	LRP1B	chr2	141031885	141032009
DAXX	chr6	33287336	33287434	LRP1B	chr2	141031998	141032121
DAXX	chr6	33287434	33287552	LRP1B	chr2	141032114	141032221
DAXX	chr6	33287549	33287650	LRP1B	chr2	141055357	141055481
DAXX	chr6	33287792	33287921	LRP1B	chr2	141055479	141055596
DAXX	chr6	33287943	33288039	LRP1B	chr2	141072485	141072569
DAXX	chr6	33288143	33288271	LRP1B	chr2	141072558	141072680
DAXX	chr6	33288268	33288373	LRP1B	chr2	141079444	141079562
DAXX	chr6	33288475	33288591	LRP1B	chr2	141079551	141079633
DAXX	chr6	33288591	33288721	LRP1B	chr2	141079622	141079729
DAXX	chr6	33288713	33288828	LRP1B	chr2	141081373	141081478
DAXX	chr6	33288828	33288918	LRP1B	chr2	141081492	141081576
DAXX	chr6	33288918	33289040	LRP1B	chr2	141081565	141081679
DAXX	chr6	33289038	33289137	LRP1B	chr2	141083235	141083345
DAXX	chr6	33289135	33289250	LRP1B	chr2	141083334	141083457
DAXX	chr6	33289242	33289317	LRP1B	chr2	141091970	141092046
DAXX	chr6	33289326	33289458	LRP1B	chr2	141092035	141092142
DAXX	chr6	33289488	33289576	LRP1B	chr2	141093096	141093208
DAXX	chr6	33289563	33289676	LRP1B	chr2	141093197	141093317
DAXX	chr6	33289671	33289798	LRP1B	chr2	141093306	141093417
DAXX	chr6	33290581	33290707	LRP1B	chr2	141108350	141108425
KMT2C	chr7	151833860	151833980	LRP1B	chr2	141108414	141108532
KMT2C	chr7	151833969	151834062	LRP1B	chr2	141108504	141108592
KMT2C	chr7	151835759	151835866	LRP1B	chr2	141108583	141108700
KMT2C	chr7	151835855	151835975	LRP1B	chr2	141110439	141110540
KMT2C	chr7	151835964	151836082	LRP1B	chr2	141110529	141110636
KMT2C	chr7	151836208	151836326	LRP1B	chr2	141110624	141110747
KMT2C	chr7	151836315	151836396	LRP1B	chr2	141113853	141113923
KMT2C	chr7	151836657	151836749	LRP1B	chr2	141113912	141114021
KMT2C	chr7	151836738	151836815	LRP1B	chr2	141114010	141114120
KMT2C	chr7	151836804	151836926	LRP1B	chr2	141115485	141115603
KMT2C	chr7	151841705	151841814	LRP1B	chr2	141115592	141115703
KMT2C	chr7	151841803	151841913	LRP1B	chr2	141116347	141116452
KMT2C	chr7	151841902	151842016	LRP1B	chr2	141116441	141116531
KMT2C	chr7	151842116	151842227	LRP1B	chr2	141122152	141122275

KMT2C	chr7	151842216	151842338	LRP1B	chr2	141122264	141122370
KMT2C	chr7	151842327	151842430	LRP1B	chr2	141128204	141128311
KMT2C	chr7	151843534	151843656	LRP1B	chr2	141128300	141128421
KMT2C	chr7	151843645	151843775	LRP1B	chr2	141128704	141128783
KMT2C	chr7	151843763	151843871	LRP1B	chr2	141128767	141128838
KMT2C	chr7	151845051	151845133	LRP1B	chr2	141130496	141130601
KMT2C	chr7	151845122	151845230	LRP1B	chr2	141130590	141130668
KMT2C	chr7	151845220	151845329	LRP1B	chr2	141130657	141130745
KMT2C	chr7	151845318	151845434	LRP1B	chr2	141135724	141135837
KMT2C	chr7	151845423	151845533	LRP1B	chr2	141135833	141135949
KMT2C	chr7	151845522	151845648	LRP1B	chr2	141143370	141143486
KMT2C	chr7	151845637	151845759	LRP1B	chr2	141143475	141143588
KMT2C	chr7	151845748	151845827	LRP1B	chr2	141199994	141200118
KMT2C	chr7	151845816	151845908	LRP1B	chr2	141200107	141200202
KMT2C	chr7	151845897	151846015	LRP1B	chr2	141201881	141201956
KMT2C	chr7	151846001	151846110	LRP1B	chr2	141201945	141202050
KMT2C	chr7	151846164	151846287	LRP1B	chr2	141202038	141202158
KMT2C	chr7	151847909	151848030	LRP1B	chr2	141202147	141202264
KMT2C	chr7	151848019	151848094	LRP1B	chr2	141208052	141208151
KMT2C	chr7	151848083	151848189	LRP1B	chr2	141208140	141208256
KMT2C	chr7	151848421	151848551	LRP1B	chr2	141213992	141214118
KMT2C	chr7	151848540	151848651	LRP1B	chr2	141214107	141214228
KMT2C	chr7	151848640	151848720	LRP1B	chr2	141215038	141215133
KMT2C	chr7	151849681	151849803	LRP1B	chr2	141215122	141215233
KMT2C	chr7	151849792	151849915	LRP1B	chr2	141232712	141232829
KMT2C	chr7	151849904	151849997	LRP1B	chr2	141232819	141232923
KMT2C	chr7	151849986	151850103	LRP1B	chr2	141242838	141242955
KMT2C	chr7	151850995	151851118	LRP1B	chr2	141242945	141243049
KMT2C	chr7	151851107	151851196	LRP1B	chr2	141243038	141243118
KMT2C	chr7	151851185	151851293	LRP1B	chr2	141245102	141245224
KMT2C	chr7	151851334	151851457	LRP1B	chr2	141245213	141245320
KMT2C	chr7	151851447	151851534	LRP1B	chr2	141250107	141250216
KMT2C	chr7	151851523	151851601	LRP1B	chr2	141250205	141250317
KMT2C	chr7	151852927	151853011	LRP1B	chr2	141253072	141253194
KMT2C	chr7	151853009	151853126	LRP1B	chr2	141253183	141253306
KMT2C	chr7	151853123	151853210	LRP1B	chr2	141253295	141253412
KMT2C	chr7	151853268	151853369	LRP1B	chr2	141259137	141259259
KMT2C	chr7	151853358	151853481	LRP1B	chr2	141259253	141259323
KMT2C	chr7	151855827	151855932	LRP1B	chr2	141259312	141259418
KMT2C	chr7	151855921	151856000	LRP1B	chr2	141259375	141259453

KMT2C	chr7	151855989	151856091	LRP1B	chr2	141260491	141260603
KMT2C	chr7	151856080	151856207	LRP1B	chr2	141260592	141260715
KMT2C	chr7	151859130	151859245	LRP1B	chr2	141264337	141264420
KMT2C	chr7	151859234	151859317	LRP1B	chr2	141264409	141264511
KMT2C	chr7	151859306	151859423	LRP1B	chr2	141267416	141267542
KMT2C	chr7	151859418	151859529	LRP1B	chr2	141267531	141267638
KMT2C	chr7	151859518	151859623	LRP1B	chr2	141272119	141272226
KMT2C	chr7	151859614	151859742	LRP1B	chr2	141272215	141272294
KMT2C	chr7	151859742	151859851	LRP1B	chr2	141272283	141272366
KMT2C	chr7	151859840	151859959	LRP1B	chr2	141274388	141274486
KMT2C	chr7	151859948	151860062	LRP1B	chr2	141274475	141274593
KMT2C	chr7	151860051	151860163	LRP1B	chr2	141283382	141283508
KMT2C	chr7	151860152	151860268	LRP1B	chr2	141283497	141283610
KMT2C	chr7	151860257	151860384	LRP1B	chr2	141283740	141283822
KMT2C	chr7	151860373	151860486	LRP1B	chr2	141283811	141283929
KMT2C	chr7	151860475	151860604	LRP1B	chr2	141291524	141291641
KMT2C	chr7	151860593	151860709	LRP1B	chr2	141291630	141291718
KMT2C	chr7	151860703	151860836	LRP1B	chr2	141291708	141291801
KMT2C	chr7	151860829	151860936	LRP1B	chr2	141294098	141294211
KMT2C	chr7	151860925	151861017	LRP1B	chr2	141294200	141294293
KMT2C	chr7	151864171	151864263	LRP1B	chr2	141298529	141298647
KMT2C	chr7	151864252	151864334	LRP1B	chr2	141298641	141298763
KMT2C	chr7	151864323	151864432	LRP1B	chr2	141299277	141299386
KMT2C	chr7	151864413	151864532	LRP1B	chr2	141299375	141299501
KMT2C	chr7	151866186	151866299	LRP1B	chr2	141299491	141299581
KMT2C	chr7	151866285	151866353	LRP1B	chr2	141356103	141356223
KMT2C	chr7	151868296	151868398	LRP1B	chr2	141356212	141356281
KMT2C	chr7	151868387	151868492	LRP1B	chr2	141356270	141356347
KMT2C	chr7	151871137	151871257	LRP1B	chr2	141356336	141356459
KMT2C	chr7	151871246	151871355	LRP1B	chr2	141359025	141359143
KMT2C	chr7	151871322	151871426	LRP1B	chr2	141359135	141359252
KMT2C	chr7	151873159	151873245	LRP1B	chr2	141457700	141457819
KMT2C	chr7	151873234	151873337	LRP1B	chr2	141457811	141457878
KMT2C	chr7	151873326	151873456	LRP1B	chr2	141457949	141458074
KMT2C	chr7	151873445	151873568	LRP1B	chr2	141458063	141458145
KMT2C	chr7	151873557	151873662	LRP1B	chr2	141458134	141458207
KMT2C	chr7	151873651	151873763	LRP1B	chr2	141459243	141459314
KMT2C	chr7	151873752	151873876	LRP1B	chr2	141459303	141459424
KMT2C	chr7	151873851	151873944	LRP1B	chr2	141459667	141459789
KMT2C	chr7	151873933	151874055	LRP1B	chr2	141459778	141459873

KMT2C	chr7	151874044	151874154	LRP1B	chr2	141459958	141460053
KMT2C	chr7	151874143	151874227	LRP1B	chr2	141460042	141460156
KMT2C	chr7	151874216	151874329	LRP1B	chr2	141473461	141473575
KMT2C	chr7	151874318	151874392	LRP1B	chr2	141473564	141473651
KMT2C	chr7	151874381	151874459	LRP1B	chr2	141473640	141473742
KMT2C	chr7	151874448	151874562	LRP1B	chr2	141474228	141474307
KMT2C	chr7	151874551	151874677	LRP1B	chr2	141474296	141474416
KMT2C	chr7	151874666	151874779	LRP1B	chr2	141526706	141526822
KMT2C	chr7	151874777	151874874	LRP1B	chr2	141526811	141526940
KMT2C	chr7	151874863	151874957	LRP1B	chr2	141528432	141528553
KMT2C	chr7	151874946	151875066	LRP1B	chr2	141528543	141528659
KMT2C	chr7	151875055	151875172	LRP1B	chr2	141533639	141533712
KMT2C	chr7	151876880	151876975	LRP1B	chr2	141533701	141533793
KMT2C	chr7	151876964	151877058	LRP1B	chr2	141533785	141533907
KMT2C	chr7	151877047	151877173	LRP1B	chr2	141571148	141571268
KMT2C	chr7	151877162	151877255	LRP1B	chr2	141571257	141571330
KMT2C	chr7	151877739	151877851	LRP1B	chr2	141571319	141571408
KMT2C	chr7	151877841	151877965	LRP1B	chr2	141597524	141597594
KMT2C	chr7	151877948	151878072	LRP1B	chr2	141597583	141597690
KMT2C	chr7	151878057	151878160	LRP1B	chr2	141598460	141598578
KMT2C	chr7	151878148	151878251	LRP1B	chr2	141598567	141598689
KMT2C	chr7	151878239	151878363	LRP1B	chr2	141607584	141607686
KMT2C	chr7	151878353	151878480	LRP1B	chr2	141607675	141607775
KMT2C	chr7	151878469	151878593	LRP1B	chr2	141607764	141607834
KMT2C	chr7	151878582	151878692	LRP1B	chr2	141607823	141607913
KMT2C	chr7	151878681	151878806	LRP1B	chr2	141609114	141609232
KMT2C	chr7	151878795	151878924	LRP1B	chr2	141609232	141609319
KMT2C	chr7	151878915	151879033	LRP1B	chr2	141609308	141609373
KMT2C	chr7	151879022	151879142	LRP1B	chr2	141625139	141625208
KMT2C	chr7	151879131	151879233	LRP1B	chr2	141625197	141625317
KMT2C	chr7	151879213	151879319	LRP1B	chr2	141625306	141625425
KMT2C	chr7	151879307	151879435	LRP1B	chr2	141625606	141625703
KMT2C	chr7	151879424	151879539	LRP1B	chr2	141625692	141625789
KMT2C	chr7	151879528	151879640	LRP1B	chr2	141625778	141625886
KMT2C	chr7	151879629	151879749	LRP1B	chr2	141641292	141641418
KMT2C	chr7	151879949	151880040	LRP1B	chr2	141641407	141641487
KMT2C	chr7	151880029	151880147	LRP1B	chr2	141641476	141641602
KMT2C	chr7	151880136	151880233	LRP1B	chr2	141643621	141643737
KMT2C	chr7	151880223	151880339	LRP1B	chr2	141643726	141643831
KMT2C	chr7	151882542	151882666	LRP1B	chr2	141643820	141643937

KMT2C	chr7	151882655	151882766	LRP1B	chr2	141660435	141660552
KMT2C	chr7	151884253	151884369	LRP1B	chr2	141660541	141660663
KMT2C	chr7	151884358	151884459	LRP1B	chr2	141660652	141660759
KMT2C	chr7	151884446	151884545	LRP1B	chr2	141665332	141665451
KMT2C	chr7	151884547	151884661	LRP1B	chr2	141665440	141665554
KMT2C	chr7	151884696	151884817	LRP1B	chr2	141665543	141665663
KMT2C	chr7	151884806	151884885	LRP1B	chr2	141680486	141680608
KMT2C	chr7	151884874	151884982	LRP1B	chr2	141680597	141680720
KMT2C	chr7	151891012	151891139	LRP1B	chr2	141680709	141680813
KMT2C	chr7	151891119	151891200	LRP1B	chr2	141707688	141707807
KMT2C	chr7	151891189	151891271	LRP1B	chr2	141707828	141707919
KMT2C	chr7	151891505	151891599	LRP1B	chr2	141707908	141707986
KMT2C	chr7	151891588	151891703	LRP1B	chr2	141709363	141709468
KMT2C	chr7	151892875	151892989	LRP1B	chr2	141709457	141709531
KMT2C	chr7	151892978	151893075	LRP1B	chr2	141739680	141739806
KMT2C	chr7	151893062	151893146	LRP1B	chr2	141739757	141739872
KMT2C	chr7	151896287	151896408	LRP1B	chr2	141747090	141747215
KMT2C	chr7	151896397	151896515	LRP1B	chr2	141747215	141747331
KMT2C	chr7	151896494	151896597	LRP1B	chr2	141751528	141751653
KMT2C	chr7	151899895	151900005	LRP1B	chr2	141751640	141751717
KMT2C	chr7	151899995	151900081	LRP1B	chr2	141762863	141762970
KMT2C	chr7	151900109	151900200	LRP1B	chr2	141762957	141763036
KMT2C	chr7	151902097	151902214	LRP1B	chr2	141771052	141771167
KMT2C	chr7	151902203	151902278	LRP1B	chr2	141771155	141771222
KMT2C	chr7	151902267	151902360	LRP1B	chr2	141771211	141771324
KMT2C	chr7	151904284	151904385	LRP1B	chr2	141773217	141773337
KMT2C	chr7	151904374	151904467	LRP1B	chr2	141773326	141773440
KMT2C	chr7	151904456	151904579	LRP1B	chr2	141773429	141773524
KMT2C	chr7	151917489	151917617	LRP1B	chr2	141777384	141777511
KMT2C	chr7	151917590	151917705	LRP1B	chr2	141777500	141777581
KMT2C	chr7	151917694	151917772	LRP1B	chr2	141777570	141777677
KMT2C	chr7	151917761	151917870	LRP1B	chr2	141806479	141806601
KMT2C	chr7	151918992	151919104	LRP1B	chr2	141806590	141806700
KMT2C	chr7	151919093	151919170	LRP1B	chr2	141806689	141806801
KMT2C	chr7	151919161	151919257	LRP1B	chr2	141812611	141812733
KMT2C	chr7	151919592	151919708	LRP1B	chr2	141812722	141812840
KMT2C	chr7	151919697	151919817	LRP1B	chr2	141816391	141816510
KMT2C	chr7	151921010	151921125	LRP1B	chr2	141816471	141816570
KMT2C	chr7	151921114	151921217	LRP1B	chr2	141819533	141819653
KMT2C	chr7	151921206	151921314	LRP1B	chr2	141819642	141819771

KMT2C	chr7	151921464	151921572	LRP1B	chr2	141819760	141819852
KMT2C	chr7	151921561	151921677	LRP1B	chr2	141945935	141946054
KMT2C	chr7	151921666	151921766	LRP1B	chr2	141946043	141946162
KMT2C	chr7	151926944	151927044	LRP1B	chr2	141986656	141986779
KMT2C	chr7	151927033	151927143	LRP1B	chr2	141986768	141986885
KMT2C	chr7	151927132	151927201	LRP1B	chr2	141986873	141986988
KMT2C	chr7	151927242	151927350	LRP1B	chr2	141986977	141987039
KMT2C	chr7	151927339	151927457	LRP1B	chr2	142004702	142004827
KMT2C	chr7	151932843	151932929	LRP1B	chr2	142004816	142004933
KMT2C	chr7	151932926	151933017	LRP1B	chr2	142012075	142012163
KMT2C	chr7	151933005	151933096	LRP1B	chr2	142012152	142012255
KMT2C	chr7	151935708	151935828	LRP1B	chr2	142237849	142237969
KMT2C	chr7	151935815	151935911	LRP1B	chr2	142237958	142238048
KMT2C	chr7	151935900	151935972	LRP1B	chr2	142238037	142238156
KMT2C	chr7	151944907	151945027	LRP1B	chr2	142567837	142567956
KMT2C	chr7	151945016	151945145	LRP1B	chr2	142567945	142568022
KMT2C	chr7	151945134	151945259	LRP1B	chr2	142888142	142888269
KMT2C	chr7	151945249	151945363	LRP1B	chr2	142888258	142888381
KMT2C	chr7	151945352	151945469	MEN1	chr11	64571750	64571860
KMT2C	chr7	151945458	151945565	MEN1	chr11	64571849	64571980
KMT2C	chr7	151945554	151945676	MEN1	chr11	64572152	64572267
KMT2C	chr7	151945665	151945774	MEN1	chr11	64572445	64572533
KMT2C	chr7	151946891	151946999	MEN1	chr11	64572520	64572605
KMT2C	chr7	151946988	151947088	MEN1	chr11	64572598	64572692
KMT2C	chr7	151947845	151947934	MEN1	chr11	64573114	64573235
KMT2C	chr7	151947923	151948014	MEN1	chr11	64573224	64573351
KMT2C	chr7	151948003	151948120	MEN1	chr11	64573650	64573750
KMT2C	chr7	151948914	151949005	MEN1	chr11	64573717	64573817
KMT2C	chr7	151948991	151949110	MEN1	chr11	64573815	64573932
KMT2C	chr7	151949100	151949225	MEN1	chr11	64574459	64574580
KMT2C	chr7	151949571	151949666	MEN1	chr11	64574592	64574708
KMT2C	chr7	151949655	151949753	MEN1	chr11	64574984	64575090
KMT2C	chr7	151949742	151949855	MEN1	chr11	64575080	64575162
KMT2C	chr7	151960049	151960173	MEN1	chr11	64575329	64575457
KMT2C	chr7	151960163	151960274	MEN1	chr11	64575446	64575546
KMT2C	chr7	151962065	151962152	MEN1	chr11	64577079	64577199
KMT2C	chr7	151962141	151962267	MEN1	chr11	64577188	64577322
KMT2C	chr7	151962254	151962352	MEN1	chr11	64577280	64577416
KMT2C	chr7	151970720	151970822	MEN1	chr11	64577410	64577548
KMT2C	chr7	151970811	151970935	MEN1	chr11	64577540	64577659

KMT2C	chr7	151970924	151971036	NCAM2	chr21	22370845	22370949
KMT2C	chr7	152006965	152007081	NCAM2	chr21	22652883	22652989
KMT2C	chr7	152007070	152007150	NCAM2	chr21	22656402	22656514
KMT2C	chr7	152007139	152007261	NCAM2	chr21	22656510	22656588
KMT2C	chr7	152008743	152008865	NCAM2	chr21	22656577	22656647
KMT2C	chr7	152008859	152008935	NCAM2	chr21	22656636	22656747
KMT2C	chr7	152008924	152009007	NCAM2	chr21	22658551	22658645
KMT2C	chr7	152008996	152009086	NCAM2	chr21	22658634	22658747
KMT2C	chr7	152012123	152012248	NCAM2	chr21	22664376	22664481
KMT2C	chr7	152012237	152012348	NCAM2	chr21	22664470	22664582
KMT2C	chr7	152012337	152012442	NCAM2	chr21	22696620	22696743
KMT2C	chr7	152012431	152012512	NCAM2	chr21	22696732	22696847
KMT2C	chr7	152027600	152027727	NCAM2	chr21	22707810	22707895
KMT2C	chr7	152027716	152027838	NCAM2	chr21	22707884	22707996
KMT2C	chr7	152027827	152027939	NCAM2	chr21	22710652	22710738
KMT2C	chr7	152055619	152055710	NCAM2	chr21	22710727	22710797
KMT2C	chr7	152055699	152055812	NCAM2	chr21	22710786	22710879
KMT2C	chr7	152132719	152132848	NCAM2	chr21	22746140	22746267
KMT2C	chr7	152132753	152132885	NCAM2	chr21	22746256	22746371
PTEN	chr10	89624204	89624323	NCAM2	chr21	22782524	22782634
PTEN	chr10	89653752	89653818	NCAM2	chr21	22782641	22782703
PTEN	chr10	89653817	89653930	NCAM2	chr21	22782692	22782763
PTEN	chr10	89685245	89685357	NCAM2	chr21	22790771	22790869
PTEN	chr10	89690820	89690917	NCAM2	chr21	22790858	22790952
PTEN	chr10	89692714	89692819	NCAM2	chr21	22804394	22804515
PTEN	chr10	89692820	89692920	NCAM2	chr21	22804503	22804613
PTEN	chr10	89692921	89693032	NCAM2	chr21	22838869	22838963
PTEN	chr10	89711803	89711928	NCAM2	chr21	22838952	22839069
PTEN	chr10	89711917	89712027	NCAM2	chr21	22840961	22841032
PTEN	chr10	89717580	89717695	NCAM2	chr21	22841021	22841139
PTEN	chr10	89717695	89717792	NCAM2	chr21	22849494	22849618
PTEN	chr10	89720564	89720686	NCAM2	chr21	22849612	22849698
PTEN	chr10	89720693	89720769	NCAM2	chr21	22849687	22849764
PTEN	chr10	89720770	89720842	NCAM2	chr21	22849753	22849833
PTEN	chr10	89724949	89725061	NCAM2	chr21	22881048	22881174
PTEN	chr10	89725059	89725147	NCAM2	chr21	22881187	22881286
PTEN	chr10	89725120	89725240	NCAM2	chr21	22881275	22881387
PTPRZ1	chr7	121513512	121513648	NCAM2	chr21	22906777	22906891
PTPRZ1	chr7	121568117	121568234	NCAM2	chr21	22906880	22906990
PTPRZ1	chr7	121568234	121568341	NCAM2	chr21	22910090	22910190

PTPRZ1	chr7	121608011	121608124	NCAM2	chr21	22910188	22910294
PTPRZ1	chr7	121608066	121608153	NOTCH2	chr1	120457827	120457943
PTPRZ1	chr7	121612580	121612652	NOTCH2	chr1	120457929	120458056
PTPRZ1	chr7	121612652	121612746	NOTCH2	chr1	120458045	120458148
PTPRZ1	chr7	121616196	121616276	NOTCH2	chr1	120458115	120458236
PTPRZ1	chr7	121616274	121616394	NOTCH2	chr1	120458228	120458308
PTPRZ1	chr7	121616860	121616956	NOTCH2	chr1	120458327	120458457
PTPRZ1	chr7	121623655	121623743	NOTCH2	chr1	120458437	120458553
PTPRZ1	chr7	121623743	121623846	NOTCH2	chr1	120458543	120458649
PTPRZ1	chr7	121623846	121623967	NOTCH2	chr1	120458629	120458738
PTPRZ1	chr7	121623992	121624085	NOTCH2	chr1	120458734	120458862
PTPRZ1	chr7	121624085	121624198	NOTCH2	chr1	120458852	120458934
PTPRZ1	chr7	121636409	121636485	NOTCH2	chr1	120458923	120459031
PTPRZ1	chr7	121636485	121636599	NOTCH2	chr1	120459020	120459141
PTPRZ1	chr7	121636599	121636714	NOTCH2	chr1	120459122	120459240
PTPRZ1	chr7	121637889	121637970	NOTCH2	chr1	120459229	120459333
PTPRZ1	chr7	121637961	121638033	NOTCH2	chr1	120460236	120460366
PTPRZ1	chr7	121638033	121638129	NOTCH2	chr1	120460355	120460435
PTPRZ1	chr7	121644631	121644753	NOTCH2	chr1	120460945	120461060
PTPRZ1	chr7	121650302	121650428	NOTCH2	chr1	120461049	120461152
PTPRZ1	chr7	121650428	121650554	NOTCH2	chr1	120461141	120461211
PTPRZ1	chr7	121650554	121650683	NOTCH2	chr1	120461919	120462003
PTPRZ1	chr7	121650681	121650800	NOTCH2	chr1	120461949	120462078
PTPRZ1	chr7	121650790	121650886	NOTCH2	chr1	120462044	120462151
PTPRZ1	chr7	121650886	121651008	NOTCH2	chr1	120462140	120462250
PTPRZ1	chr7	121651008	121651112	NOTCH2	chr1	120462799	120462929
PTPRZ1	chr7	121651112	121651221	NOTCH2	chr1	120462884	120462984
PTPRZ1	chr7	121651219	121651344	NOTCH2	chr1	120464234	120464357
PTPRZ1	chr7	121651344	121651422	NOTCH2	chr1	120464346	120464458
PTPRZ1	chr7	121651422	121651503	NOTCH2	chr1	120464818	120464920
PTPRZ1	chr7	121651503	121651628	NOTCH2	chr1	120464916	120464994
PTPRZ1	chr7	121651628	121651754	NOTCH2	chr1	120464983	120465081
PTPRZ1	chr7	121651752	121651835	NOTCH2	chr1	120465150	120465267
PTPRZ1	chr7	121651834	121651956	NOTCH2	chr1	120465259	120465372
PTPRZ1	chr7	121651956	121652044	NOTCH2	chr1	120465361	120465440
PTPRZ1	chr7	121652044	121652156	NOTCH2	chr1	120466205	120466320
PTPRZ1	chr7	121652156	121652279	NOTCH2	chr1	120466316	120466444
PTPRZ1	chr7	121652279	121652369	NOTCH2	chr1	120466466	120466564
PTPRZ1	chr7	121652369	121652496	NOTCH2	chr1	120466553	120466632
PTPRZ1	chr7	121652496	121652572	NOTCH2	chr1	120467885	120468012

PTPRZ1	chr7	121652572	121652702	NOTCH2	chr1	120468003	120468136
PTPRZ1	chr7	121652700	121652818	NOTCH2	chr1	120468125	120468231
PTPRZ1	chr7	121652818	121652920	NOTCH2	chr1	120468210	120468296
PTPRZ1	chr7	121652920	121653019	NOTCH2	chr1	120468322	120468443
PTPRZ1	chr7	121653015	121653135	NOTCH2	chr1	120469009	120469132
PTPRZ1	chr7	121653135	121653250	NOTCH2	chr1	120469121	120469202
PTPRZ1	chr7	121653241	121653319	NOTCH2	chr1	120471564	120471688
PTPRZ1	chr7	121653319	121653429	NOTCH2	chr1	120471655	120471771
PTPRZ1	chr7	121653459	121653580	NOTCH2	chr1	120471759	120471861
PTPRZ1	chr7	121653580	121653652	NOTCH2	chr1	120477990	120478111
PTPRZ1	chr7	121653652	121653762	NOTCH2	chr1	120478096	120478169
PTPRZ1	chr7	121653762	121653887	NOTCH2	chr1	120478158	120478244
PTPRZ1	chr7	121653887	121653965	NOTCH2	chr1	120479848	120479922
PTPRZ1	chr7	121659105	121659187	NOTCH2	chr1	120479917	120480016
PTPRZ1	chr7	121659187	121659280	NOTCH2	chr1	120480005	120480102
PTPRZ1	chr7	121659280	121659407	NOTCH2	chr1	120480435	120480539
PTPRZ1	chr7	121668565	121668657	NOTCH2	chr1	120480528	120480622
PTPRZ1	chr7	121668657	121668737	NOTCH2	chr1	120480622	120480723
PTPRZ1	chr7	121671478	121671545	NOTCH2	chr1	120483071	120483182
PTPRZ1	chr7	121671558	121671681	NOTCH2	chr1	120483172	120483277
PTPRZ1	chr7	121674152	121674274	NOTCH2	chr1	120483266	120483389
PTPRZ1	chr7	121674284	121674356	NOTCH2	chr1	120484069	120484176
PTPRZ1	chr7	121674356	121674446	NOTCH2	chr1	120484165	120484274
PTPRZ1	chr7	121676624	121676696	NOTCH2	chr1	120484263	120484387
PTPRZ1	chr7	121676696	121676798	NOTCH2	chr1	120490931	120491044
PTPRZ1	chr7	121678749	121678827	NOTCH2	chr1	120491033	120491116
PTPRZ1	chr7	121678826	121678905	NOTCH2	chr1	120491105	120491202
PTPRZ1	chr7	121678903	121679006	NOTCH2	chr1	120491561	120491687
PTPRZ1	chr7	121679469	121679565	NOTCH2	chr1	120491676	120491789
PTPRZ1	chr7	121679565	121679651	NOTCH2	chr1	120493297	120493400
PTPRZ1	chr7	121680781	121680910	NOTCH2	chr1	120493389	120493477
PTPRZ1	chr7	121680910	121681040	NOTCH2	chr1	120496152	120496247
PTPRZ1	chr7	121682562	121682686	NOTCH2	chr1	120496236	120496354
PTPRZ1	chr7	121682672	121682749	NOTCH2	chr1	120497601	120497699
PTPRZ1	chr7	121684394	121684516	NOTCH2	chr1	120497688	120497813
PTPRZ1	chr7	121684516	121684630	NOTCH2	chr1	120497801	120497878
PTPRZ1	chr7	121691486	121691561	NOTCH2	chr1	120501905	120502029
PTPRZ1	chr7	121691561	121691636	NOTCH2	chr1	120502018	120502135
PTPRZ1	chr7	121691833	121691938	NOTCH2	chr1	120506097	120506225
PTPRZ1	chr7	121691938	121692026	NOTCH2	chr1	120506200	120506322

PTPRZ1	chr7	121693933	121694016	NOTCH2	chr1	120506306	120506419
PTPRZ1	chr7	121694016	121694101	NOTCH2	chr1	120506408	120506484
PTPRZ1	chr7	121694920	121695042	NOTCH2	chr1	120508019	120508126
PTPRZ1	chr7	121695023	121695114	NOTCH2	chr1	120508115	120508200
PTPRZ1	chr7	121695089	121695195	NOTCH2	chr1	120508939	120509045
PTPRZ1	chr7	121698807	121698891	NOTCH2	chr1	120509034	120509123
PTPRZ1	chr7	121698891	121699008	NOTCH2	chr1	120510029	120510158
PTPRZ1	chr7	121699725	121699835	NOTCH2	chr1	120510147	120510254
PTPRZ1	chr7	121699835	121699947	NOTCH2	chr1	120510581	120510708
PTPRZ1	chr7	121701048	121701175	NOTCH2	chr1	120510697	120510821
PTPRZ1	chr7	121701175	121701292	NOTCH2	chr1	120510782	120510871
RAI1	chr17	17696149	17696271	NOTCH2	chr1	120512086	120512210
RAI1	chr17	17696268	17696373	NOTCH2	chr1	120512133	120512262
RAI1	chr17	17696354	17696435	NOTCH2	chr1	120512251	120512375
RAI1	chr17	17696429	17696558	NOTCH2	chr1	120512363	120512441
RAI1	chr17	17696495	17696599	NOTCH2	chr1	120529543	120529672
RAI1	chr17	17696598	17696720	NOTCH2	chr1	120529661	120529762
RAI1	chr17	17696714	17696833	NOTCH2	chr1	120539521	120539630
RAI1	chr17	17696832	17696961	NOTCH2	chr1	120539619	120539701
RAI1	chr17	17696946	17697059	NOTCH2	chr1	120539689	120539817
RAI1	chr17	17697059	17697193	NOTCH2	chr1	120539816	120539937
RAI1	chr17	17697193	17697329	NOTCH2	chr1	120539925	120540008
RAI1	chr17	17697329	17697422	NOTCH2	chr1	120547928	120548049
RAI1	chr17	17697422	17697532	NOTCH2	chr1	120548038	120548141
RAI1	chr17	17697531	17697649	NOTCH2	chr1	120548190	120548268
RAI1	chr17	17697649	17697740	NOTCH2	chr1	120572504	120572620
RAI1	chr17	17697729	17697849	PCLO	chr7	82387785	82387910
RAI1	chr17	17697848	17697978	PCLO	chr7	82387899	82387992
RAI1	chr17	17697977	17698105	PCLO	chr7	82387981	82388104
RAI1	chr17	17698097	17698190	PCLO	chr7	82389855	82389970
RAI1	chr17	17698190	17698318	PCLO	chr7	82389978	82390098
RAI1	chr17	17698310	17698441	PCLO	chr7	82390635	82390720
RAI1	chr17	17698440	17698559	PCLO	chr7	82390704	82390792
RAI1	chr17	17698559	17698686	PCLO	chr7	82390792	82390905
RAI1	chr17	17698679	17698815	PCLO	chr7	82430818	82430935
RAI1	chr17	17698811	17698947	PCLO	chr7	82434951	82435049
RAI1	chr17	17698943	17699066	PCLO	chr7	82435031	82435149
RAI1	chr17	17699066	17699182	PCLO	chr7	82451773	82451893
RAI1	chr17	17699177	17699313	PCLO	chr7	82451828	82451947
RAI1	chr17	17699309	17699438	PCLO	chr7	82451936	82452044

RAI1	chr17	17699437	17699546	PCLO	chr7	82453509	82453580
RAI1	chr17	17699543	17699646	PCLO	chr7	82453569	82453684
RAI1	chr17	17699645	17699777	PCLO	chr7	82453699	82453816
RAI1	chr17	17699735	17699817	PCLO	chr7	82455837	82455947
RAI1	chr17	17699911	17700028	PCLO	chr7	82455945	82456060
RAI1	chr17	17700025	17700154	PCLO	chr7	82457113	82457214
RAI1	chr17	17700141	17700262	PCLO	chr7	82457202	82457276
RAI1	chr17	17700261	17700349	PCLO	chr7	82464929	82465045
RAI1	chr17	17700340	17700467	PCLO	chr7	82467434	82467553
RAI1	chr17	17700467	17700591	PCLO	chr7	82467542	82467652
RAI1	chr17	17700551	17700651	PCLO	chr7	82467667	82467755
RAI1	chr17	17700641	17700754	PCLO	chr7	82470717	82470807
RAI1	chr17	17700754	17700871	PCLO	chr7	82470796	82470868
RAI1	chr17	17700869	17700984	PCLO	chr7	82474574	82474682
RAI1	chr17	17700984	17701112	PCLO	chr7	82474671	82474788
RAI1	chr17	17701106	17701219	PCLO	chr7	82474777	82474850
RAI1	chr17	17701219	17701344	PCLO	chr7	82475827	82475903
RAI1	chr17	17701322	17701398	PCLO	chr7	82475892	82475976
RAI1	chr17	17701397	17701481	PCLO	chr7	82476374	82476481
RAI1	chr17	17701479	17701616	PCLO	chr7	82476470	82476584
RAI1	chr17	17701615	17701720	PCLO	chr7	82508584	82508708
RAI1	chr17	17701691	17701818	PCLO	chr7	82508707	82508816
RAI1	chr17	17701815	17701900	PCLO	chr7	82531979	82532081
RAI1	chr17	17707047	17707179	PCLO	chr7	82538138	82538233
RAI1	chr17	17712680	17712784	PCLO	chr7	82538222	82538339
RAI1	chr17	17713243	17713348	PCLO	chr7	82543902	82544021
SETD2	chr3	47058484	47058600	PCLO	chr7	82544010	82544139
SETD2	chr3	47058592	47058693	PCLO	chr7	82544128	82544238
SETD2	chr3	47058682	47058775	PCLO	chr7	82544227	82544307
SETD2	chr3	47058748	47058844	PCLO	chr7	82544296	82544385
SETD2	chr3	47059029	47059158	PCLO	chr7	82544374	82544482
SETD2	chr3	47059147	47059253	PCLO	chr7	82544471	82544559
SETD2	chr3	47059247	47059328	PCLO	chr7	82544548	82544653
SETD2	chr3	47061145	47061275	PCLO	chr7	82544642	82544763
SETD2	chr3	47061264	47061389	PCLO	chr7	82544748	82544822
SETD2	chr3	47079043	47079163	PCLO	chr7	82544811	82544932
SETD2	chr3	47079152	47079274	PCLO	chr7	82544921	82545046
SETD2	chr3	47079263	47079365	PCLO	chr7	82545035	82545132
SETD2	chr3	47083949	47084063	PCLO	chr7	82545121	82545242
SETD2	chr3	47084052	47084154	PCLO	chr7	82545231	82545350

SETD2	chr3	47084140	47084240	PCLO	chr7	82545339	82545443
SETD2	chr3	47087906	47088000	PCLO	chr7	82545432	82545545
SETD2	chr3	47087985	47088078	PCLO	chr7	82545534	82545661
SETD2	chr3	47088067	47088161	PCLO	chr7	82545650	82545771
SETD2	chr3	47098236	47098339	PCLO	chr7	82545760	82545861
SETD2	chr3	47098328	47098444	PCLO	chr7	82545850	82545962
SETD2	chr3	47098433	47098550	PCLO	chr7	82545951	82546072
SETD2	chr3	47098539	47098660	PCLO	chr7	82546061	82546168
SETD2	chr3	47098649	47098773	PCLO	chr7	82546166	82546283
SETD2	chr3	47098764	47098887	PCLO	chr7	82578717	82578803
SETD2	chr3	47098873	47098982	PCLO	chr7	82578792	82578911
SETD2	chr3	47098957	47099049	PCLO	chr7	82578900	82579013
SETD2	chr3	47103562	47103645	PCLO	chr7	82579002	82579128
SETD2	chr3	47103619	47103731	PCLO	chr7	82579117	82579218
SETD2	chr3	47103720	47103825	PCLO	chr7	82579207	82579328
SETD2	chr3	47103814	47103888	PCLO	chr7	82579317	82579443
SETD2	chr3	47108507	47108587	PCLO	chr7	82579432	82579558
SETD2	chr3	47108576	47108658	PCLO	chr7	82579547	82579666
SETD2	chr3	47125057	47125181	PCLO	chr7	82579655	82579779
SETD2	chr3	47125170	47125249	PCLO	chr7	82579768	82579878
SETD2	chr3	47125238	47125344	PCLO	chr7	82579867	82579995
SETD2	chr3	47125333	47125445	PCLO	chr7	82579981	82580081
SETD2	chr3	47125434	47125561	PCLO	chr7	82580070	82580193
SETD2	chr3	47125550	47125667	PCLO	chr7	82580182	82580291
SETD2	chr3	47125656	47125781	PCLO	chr7	82580280	82580396
SETD2	chr3	47125769	47125890	PCLO	chr7	82580385	82580511
SETD2	chr3	47127537	47127654	PCLO	chr7	82580500	82580613
SETD2	chr3	47127643	47127762	PCLO	chr7	82580602	82580726
SETD2	chr3	47127751	47127854	PCLO	chr7	82580715	82580818
SETD2	chr3	47129568	47129664	PCLO	chr7	82581134	82581252
SETD2	chr3	47129653	47129731	PCLO	chr7	82581238	82581346
SETD2	chr3	47129720	47129794	PCLO	chr7	82581335	82581441
SETD2	chr3	47139351	47139471	PCLO	chr7	82581430	82581545
SETD2	chr3	47139460	47139570	PCLO	chr7	82581534	82581655
SETD2	chr3	47139559	47139635	PCLO	chr7	82581644	82581770
SETD2	chr3	47142824	47142936	PCLO	chr7	82581759	82581867
SETD2	chr3	47142932	47143041	PCLO	chr7	82581856	82581982
SETD2	chr3	47143031	47143134	PCLO	chr7	82581971	82582092
SETD2	chr3	47144769	47144882	PCLO	chr7	82582081	82582206
SETD2	chr3	47144871	47144963	PCLO	chr7	82582195	82582316

SETD2	chr3	47147438	47147521	PCLO	chr7	82582302	82582416
SETD2	chr3	47147510	47147629	PCLO	chr7	82582405	82582531
SETD2	chr3	47147618	47147741	PCLO	chr7	82582520	82582637
SETD2	chr3	47155378	47155473	PCLO	chr7	82582626	82582739
SETD2	chr3	47155462	47155531	PCLO	chr7	82582728	82582847
SETD2	chr3	47158019	47158110	PCLO	chr7	82582836	82582950
SETD2	chr3	47158099	47158203	PCLO	chr7	82582939	82583053
SETD2	chr3	47158198	47158314	PCLO	chr7	82583084	82583179
SETD2	chr3	47161615	47161736	PCLO	chr7	82583168	82583288
SETD2	chr3	47161728	47161812	PCLO	chr7	82583277	82583389
SETD2	chr3	47161784	47161885	PCLO	chr7	82583378	82583471
SETD2	chr3	47161873	47162002	PCLO	chr7	82583450	82583548
SETD2	chr3	47161993	47162097	PCLO	chr7	82583537	82583656
SETD2	chr3	47162086	47162210	PCLO	chr7	82583645	82583769
SETD2	chr3	47162199	47162309	PCLO	chr7	82583758	82583881
SETD2	chr3	47162298	47162425	PCLO	chr7	82583870	82583950
SETD2	chr3	47162414	47162538	PCLO	chr7	82583939	82584057
SETD2	chr3	47162527	47162626	PCLO	chr7	82584046	82584166
SETD2	chr3	47162615	47162723	PCLO	chr7	82584155	82584272
SETD2	chr3	47162712	47162826	PCLO	chr7	82584261	82584361
SETD2	chr3	47162815	47162933	PCLO	chr7	82584350	82584469
SETD2	chr3	47162922	47163046	PCLO	chr7	82584458	82584574
SETD2	chr3	47163035	47163133	PCLO	chr7	82584563	82584648
SETD2	chr3	47163122	47163204	PCLO	chr7	82584632	82584711
SETD2	chr3	47163193	47163311	PCLO	chr7	82584700	82584813
SETD2	chr3	47163300	47163425	PCLO	chr7	82584802	82584923
SETD2	chr3	47163414	47163522	PCLO	chr7	82584912	82585037
SETD2	chr3	47163511	47163615	PCLO	chr7	82585026	82585132
SETD2	chr3	47163582	47163660	PCLO	chr7	82585121	82585209
SETD2	chr3	47163729	47163849	PCLO	chr7	82585198	82585289
SETD2	chr3	47163838	47163957	PCLO	chr7	82585278	82585386
SETD2	chr3	47163946	47164071	PCLO	chr7	82585375	82585476
SETD2	chr3	47164070	47164184	PCLO	chr7	82585465	82585557
SETD2	chr3	47164155	47164250	PCLO	chr7	82585546	82585668
SETD2	chr3	47164239	47164322	PCLO	chr7	82585657	82585780
SETD2	chr3	47164311	47164422	PCLO	chr7	82585769	82585885
SETD2	chr3	47164377	47164480	PCLO	chr7	82585874	82585993
SETD2	chr3	47164574	47164658	PCLO	chr7	82585982	82586091
SETD2	chr3	47164647	47164731	PCLO	chr7	82586080	82586169
SETD2	chr3	47164720	47164847	PCLO	chr7	82586158	82586279

SETD2	chr3	47164836	47164932	PCLO	chr7	82595068	82595170
SETD2	chr3	47164919	47165042	PCLO	chr7	82595159	82595241
SETD2	chr3	47165031	47165113	PCLO	chr7	82595230	82595355
SETD2	chr3	47165102	47165219	PCLO	chr7	82595354	82595482
SETD2	chr3	47165208	47165297	PCLO	chr7	82595472	82595564
SETD2	chr3	47165286	47165395	PCLO	chr7	82595547	82595619
SETD2	chr3	47165384	47165495	PCLO	chr7	82595675	82595779
SETD2	chr3	47165482	47165603	PCLO	chr7	82595768	82595886
SETD2	chr3	47165592	47165690	PCLO	chr7	82763512	82763631
SETD2	chr3	47165679	47165769	PCLO	chr7	82763620	82763738
SETD2	chr3	47165758	47165869	PCLO	chr7	82763727	82763837
SETD2	chr3	47165858	47165984	PCLO	chr7	82763825	82763933
SETD2	chr3	47165973	47166088	PCLO	chr7	82763899	82764018
SETD2	chr3	47168064	47168163	PCLO	chr7	82764007	82764125
SETD2	chr3	47168162	47168237	PCLO	chr7	82764114	82764244
SMARCA1	chrX	128581070	128581171	PCLO	chr7	82764257	82764384
SMARCA1	chrX	128581160	128581255	PCLO	chr7	82764374	82764490
SMARCA1	chrX	128581244	128581340	PCLO	chr7	82764479	82764557
SMARCA1	chrX	128582181	128582304	PCLO	chr7	82764546	82764672
SMARCA1	chrX	128582293	128582377	PCLO	chr7	82764661	82764759
SMARCA1	chrX	128582366	128582447	PCLO	chr7	82764748	82764867
SMARCA1	chrX	128582435	128582512	PCLO	chr7	82764859	82764983
SMARCA1	chrX	128599405	128599502	PCLO	chr7	82783989	82784111
SMARCA1	chrX	128599491	128599574	PCLO	chr7	82784100	82784226
SMARCA1	chrX	128599563	128599668	PCLO	chr7	82784215	82784331
SMARCA1	chrX	128599657	128599759	PCLO	chr7	82784555	82784660
SMARCA1	chrX	128599758	128599842	PCLO	chr7	82784649	82784745
SMARCA1	chrX	128599831	128599910	PCLO	chr7	82784696	82784808
SMARCA1	chrX	128599899	128600016	PCLO	chr7	82784794	82784904
SMARCA1	chrX	128602695	128602804	PCLO	chr7	82784925	82785044
SMARCA1	chrX	128602793	128602911	PCLO	chr7	82785034	82785149
SMARCA1	chrX	128602900	128603011	PCLO	chr7	82785138	82785221
SMARCA1	chrX	128605094	128605219	PCLO	chr7	82785210	82785314
SMARCA1	chrX	128605208	128605286	PCLO	chr7	82785303	82785388
SMARCA1	chrX	128605282	128605405	PCLO	chr7	82785377	82785494
SMARCA1	chrX	128614586	128614709	PCLO	chr7	82785483	82785583
SMARCA1	chrX	128614698	128614785	PCLO	chr7	82785572	82785693
SMARCA1	chrX	128614774	128614886	PCLO	chr7	82785703	82785818
SMARCA1	chrX	128614921	128615017	PCLO	chr7	82791623	82791757
SMARCA1	chrX	128615006	128615107	PCLO	chr7	82791757	82791885

SMARCA1	chrX	128615096	128615212	PSIP1	chr9	15465496	15465599
SMARCA1	chrX	128620883	128620973	PSIP1	chr9	15466690	15466796
SMARCA1	chrX	128620969	128621084	PSIP1	chr9	15466785	15466906
SMARCA1	chrX	128621107	128621191	PSIP1	chr9	15468592	15468718
SMARCA1	chrX	128622822	128622936	PSIP1	chr9	15468707	15468830
SMARCA1	chrX	128622924	128623041	PSIP1	chr9	15468819	15468913
SMARCA1	chrX	128623036	128623148	PSIP1	chr9	15468943	15469030
SMARCA1	chrX	128623926	128624034	PSIP1	chr9	15469019	15469136
SMARCA1	chrX	128623995	128624090	PSIP1	chr9	15469173	15469295
SMARCA1	chrX	128624143	128624264	PSIP1	chr9	15469288	15469353
SMARCA1	chrX	128625793	128625913	PSIP1	chr9	15469822	15469942
SMARCA1	chrX	128625902	128626008	PSIP1	chr9	15469939	15470010
SMARCA1	chrX	128625997	128626120	PSIP1	chr9	15471154	15471277
SMARCA1	chrX	128626930	128627055	PSIP1	chr9	15472601	15472711
SMARCA1	chrX	128627017	128627131	PSIP1	chr9	15472672	15472778
SMARCA1	chrX	128630632	128630753	PSIP1	chr9	15474022	15474137
SMARCA1	chrX	128630742	128630821	PSIP1	chr9	15474136	15474244
SMARCA1	chrX	128630810	128630917	PSIP1	chr9	15474238	15474318
SMARCA1	chrX	128631730	128631850	PSIP1	chr9	15478424	15478540
SMARCA1	chrX	128631839	128631915	PSIP1	chr9	15479538	15479662
SMARCA1	chrX	128631904	128631984	PSIP1	chr9	15479596	15479706
SMARCA1	chrX	128631973	128632098	PSIP1	chr9	15485945	15486040
SMARCA1	chrX	128633595	128633714	PSIP1	chr9	15486017	15486104
SMARCA1	chrX	128633713	128633783	PSIP1	chr9	15486780	15486872
SMARCA1	chrX	128633772	128633886	PSIP1	chr9	15486846	15486927
SMARCA1	chrX	128638618	128638733	PSIP1	chr9	15486935	15487057
SMARCA1	chrX	128638722	128638830	PSIP1	chr9	15489938	15490039
SMARCA1	chrX	128639945	128640034	PSIP1	chr9	15490028	15490136
SMARCA1	chrX	128640023	128640121	PSIP1	chr9	15506517	15506605
SMARCA1	chrX	128640110	128640230	PSIP1	chr9	15506577	15506695
SMARCA1	chrX	128640198	128640300	PSIP1	chr9	15510056	15510176
SMARCA1	chrX	128641840	128641963	PSIP1	chr9	15510192	15510324
SMARCA1	chrX	128641958	128642052	PTPRZ1	chr7	121513512	121513635
SMARCA1	chrX	128642041	128642161	PTPRZ1	chr7	121568122	121568245
SMARCA1	chrX	128645711	128645803	PTPRZ1	chr7	121568234	121568339
SMARCA1	chrX	128645792	128645916	PTPRZ1	chr7	121607881	121607998
SMARCA1	chrX	128645905	128646022	PTPRZ1	chr7	121608002	121608104
SMARCA1	chrX	128649579	128649665	PTPRZ1	chr7	121608082	121608182
SMARCA1	chrX	128649654	128649771	PTPRZ1	chr7	121608179	121608290
SMARCA1	chrX	128649749	128649833	PTPRZ1	chr7	121612577	121612649

SMARCA1	chrX	128649882	128649990	PTPRZ1	chr7	121612638	121612751
SMARCA1	chrX	128649979	128650055	PTPRZ1	chr7	121616180	121616288
SMARCA1	chrX	128650214	128650322	PTPRZ1	chr7	121616277	121616389
SMARCA1	chrX	128650311	128650433	PTPRZ1	chr7	121616853	121616927
SMARCA1	chrX	128650422	128650540	PTPRZ1	chr7	121623651	121623746
SMARCA1	chrX	128652324	128652390	PTPRZ1	chr7	121623735	121623850
SMARCA1	chrX	128652379	128652474	PTPRZ1	chr7	121623839	121623953
SMARCA1	chrX	128657146	128657270	PTPRZ1	chr7	121623983	121624081
SMARCA1	chrX	128657157	128657285	PTPRZ1	chr7	121624070	121624187
SMARCA2	chr9	2028866	2028994	PTPRZ1	chr7	121636409	121636491
SMARCA2	chr9	2028994	2029094	PTPRZ1	chr7	121636480	121636561
SMARCA2	chr9	2029051	2029165	PTPRZ1	chr7	121636550	121636669
SMARCA2	chr9	2029154	2029251	PTPRZ1	chr7	121637888	121638008
SMARCA2	chr9	2029240	2029327	PTPRZ1	chr7	121637997	121638113
SMARCA2	chr9	2032876	2032976	PTPRZ1	chr7	121644631	121644751
SMARCA2	chr9	2032965	2033054	PTPRZ1	chr7	121650287	121650407
SMARCA2	chr9	2033023	2033115	PTPRZ1	chr7	121650396	121650519
SMARCA2	chr9	2039375	2039460	PTPRZ1	chr7	121650508	121650626
SMARCA2	chr9	2039449	2039538	PTPRZ1	chr7	121650615	121650729
SMARCA2	chr9	2039527	2039647	PTPRZ1	chr7	121650718	121650836
SMARCA2	chr9	2039636	2039733	PTPRZ1	chr7	121650825	121650932
SMARCA2	chr9	2039723	2039843	PTPRZ1	chr7	121650921	121651039
SMARCA2	chr9	2039766	2039896	PTPRZ1	chr7	121651028	121651148
SMARCA2	chr9	2039894	2039994	PTPRZ1	chr7	121651137	121651259
SMARCA2	chr9	2047087	2047207	PTPRZ1	chr7	121651248	121651370
SMARCA2	chr9	2047449	2047559	PTPRZ1	chr7	121651359	121651487
SMARCA2	chr9	2054524	2054647	PTPRZ1	chr7	121651476	121651578
SMARCA2	chr9	2054636	2054706	PTPRZ1	chr7	121651567	121651692
SMARCA2	chr9	2054695	2054773	PTPRZ1	chr7	121651671	121651776
SMARCA2	chr9	2056601	2056723	PTPRZ1	chr7	121651765	121651876
SMARCA2	chr9	2056679	2056803	PTPRZ1	chr7	121651865	121651967
SMARCA2	chr9	2056826	2056912	PTPRZ1	chr7	121651956	121652082
SMARCA2	chr9	2058206	2058324	PTPRZ1	chr7	121652065	121652181
SMARCA2	chr9	2058313	2058431	PTPRZ1	chr7	121652170	121652281
SMARCA2	chr9	2058420	2058514	PTPRZ1	chr7	121652270	121652393
SMARCA2	chr9	2060715	2060840	PTPRZ1	chr7	121652382	121652492
SMARCA2	chr9	2060829	2060949	PTPRZ1	chr7	121652481	121652585
SMARCA2	chr9	2060937	2061037	PTPRZ1	chr7	121652574	121652685
SMARCA2	chr9	2070354	2070463	PTPRZ1	chr7	121652674	121652802
SMARCA2	chr9	2070432	2070522	PTPRZ1	chr7	121652791	121652907

SMARCA2	chr9	2073083	2073212	PTPRZ1	chr7	121652896	121653017
SMARCA2	chr9	2073201	2073325	PTPRZ1	chr7	121653006	121653132
SMARCA2	chr9	2073280	2073397	PTPRZ1	chr7	121653122	121653196
SMARCA2	chr9	2073453	2073578	PTPRZ1	chr7	121653280	121653399
SMARCA2	chr9	2073567	2073673	PTPRZ1	chr7	121653355	121653473
SMARCA2	chr9	2076162	2076275	PTPRZ1	chr7	121653462	121653585
SMARCA2	chr9	2076264	2076387	PTPRZ1	chr7	121653580	121653645
SMARCA2	chr9	2077549	2077647	PTPRZ1	chr7	121653634	121653756
SMARCA2	chr9	2077636	2077748	PTPRZ1	chr7	121653745	121653853
SMARCA2	chr9	2077737	2077826	PTPRZ1	chr7	121653842	121653953
SMARCA2	chr9	2081726	2081845	PTPRZ1	chr7	121659136	121659260
SMARCA2	chr9	2081834	2081933	PTPRZ1	chr7	121659249	121659358
SMARCA2	chr9	2081922	2082029	PTPRZ1	chr7	121668545	121668651
SMARCA2	chr9	2082018	2082090	PTPRZ1	chr7	121668640	121668715
SMARCA2	chr9	2083269	2083378	PTPRZ1	chr7	121671459	121671553
SMARCA2	chr9	2083367	2083465	PTPRZ1	chr7	121671542	121671663
SMARCA2	chr9	2083957	2084073	PTPRZ1	chr7	121674109	121674187
SMARCA2	chr9	2084062	2084167	PTPRZ1	chr7	121674303	121674390
SMARCA2	chr9	2084156	2084246	PTPRZ1	chr7	121674378	121674451
SMARCA2	chr9	2086741	2086864	PTPRZ1	chr7	121676624	121676696
SMARCA2	chr9	2086853	2086954	PTPRZ1	chr7	121676685	121676796
SMARCA2	chr9	2086943	2087020	PTPRZ1	chr7	121678749	121678848
SMARCA2	chr9	2087009	2087121	PTPRZ1	chr7	121678837	121678906
SMARCA2	chr9	2088428	2088520	PTPRZ1	chr7	121679453	121679576
SMARCA2	chr9	2088509	2088590	PTPRZ1	chr7	121679565	121679652
SMARCA2	chr9	2088579	2088663	PTPRZ1	chr7	121680852	121680931
SMARCA2	chr9	2096600	2096727	PTPRZ1	chr7	121680920	121681044
SMARCA2	chr9	2096716	2096817	PTPRZ1	chr7	121682566	121682682
SMARCA2	chr9	2097302	2097417	PTPRZ1	chr7	121682671	121682747
SMARCA2	chr9	2097406	2097486	PTPRZ1	chr7	121682736	121682831
SMARCA2	chr9	2097475	2097586	PTPRZ1	chr7	121684364	121684485
SMARCA2	chr9	2101510	2101603	PTPRZ1	chr7	121684474	121684544
SMARCA2	chr9	2101591	2101666	PTPRZ1	chr7	121684533	121684632
SMARCA2	chr9	2103932	2104027	PTPRZ1	chr7	121691396	121691511
SMARCA2	chr9	2104016	2104104	PTPRZ1	chr7	121691500	121691619
SMARCA2	chr9	2104093	2104219	PTPRZ1	chr7	121691855	121691937
SMARCA2	chr9	2110162	2110285	PTPRZ1	chr7	121691926	121692024
SMARCA2	chr9	2110274	2110380	PTPRZ1	chr7	121693943	121694031
SMARCA2	chr9	2110369	2110477	PTPRZ1	chr7	121694020	121694102
SMARCA2	chr9	2115667	2115788	PTPRZ1	chr7	121694903	121695004

SMARCA2	chr9	2115777	2115903	PTPRZ1	chr7	121694993	121695111
SMARCA2	chr9	2115898	2115979	PTPRZ1	chr7	121695099	121695187
SMARCA2	chr9	2115964	2116074	PTPRZ1	chr7	121698763	121698893
SMARCA2	chr9	2119393	2119507	PTPRZ1	chr7	121698884	121699006
SMARCA2	chr9	2119496	2119623	PTPRZ1	chr7	121699744	121699864
SMARCA2	chr9	2123581	2123701	PTPRZ1	chr7	121699853	121699973
SMARCA2	chr9	2123690	2123803	PTPRZ1	chr7	121701056	121701185
SMARCA2	chr9	2123792	2123920	PTPRZ1	chr7	121701174	121701291
SMARCA2	chr9	2123873	2123961	RIN3	chr14	92980289	92980382
SMARCA2	chr9	2157748	2157868	RIN3	chr14	93022079	93022204
SMARCA2	chr9	2157835	2157951	RIN3	chr14	93022189	93022313
SMARCA2	chr9	2158883	2158968	RIN3	chr14	93043668	93043789
SMARCA2	chr9	2158957	2159069	RIN3	chr14	93043778	93043883
SMARCA2	chr9	2159715	2159837	RIN3	chr14	93081717	93081844
SMARCA2	chr9	2159826	2159931	RIN3	chr14	93107553	93107673
SMARCA2	chr9	2159920	2159995	RIN3	chr14	93107683	93107812
SMARCA2	chr9	2161551	2161666	RIN3	chr14	93117832	93117942
SMARCA2	chr9	2161658	2161772	RIN3	chr14	93117931	93118060
SMARCA2	chr9	2161761	2161880	RIN3	chr14	93118058	93118154
SMARCA2	chr9	2161868	2161970	RIN3	chr14	93118421	93118518
SMARCA2	chr9	2170358	2170458	RIN3	chr14	93118474	93118580
SMARCA2	chr9	2170447	2170526	RIN3	chr14	93118580	93118716
SMARCA2	chr9	2181426	2181549	RIN3	chr14	93118721	93118824
SMARCA2	chr9	2181538	2181639	RIN3	chr14	93118808	93118896
SMARCA2	chr9	2181628	2181726	RIN3	chr14	93118872	93118990
SMARCA2	chr9	2182048	2182153	RIN3	chr14	93118979	93119092
SMARCA2	chr9	2182142	2182240	RIN3	chr14	93119048	93119179
SMARCA2	chr9	2182219	2182313	RIN3	chr14	93119167	93119255
SMARCA2	chr9	2185961	2186080	RIN3	chr14	93119217	93119342
SMARCA2	chr9	2186069	2186172	RIN3	chr14	93119331	93119464
SMARCA2	chr9	2186161	2186278	RIN3	chr14	93125382	93125507
SMARCA2	chr9	2191128	2191250	RIN3	chr14	93125498	93125612
SMARCA2	chr9	2191239	2191318	RIN3	chr14	93125601	93125729
SMARCA2	chr9	2191386	2191483	RIN3	chr14	93125718	93125847
SMARCA2	chr9	2192608	2192733	RIN3	chr14	93142789	93142888
SMARCA2	chr9	2192722	2192793	RIN3	chr14	93142870	93142970
SMARCA2	chr9	2029010	2029139	RIN3	chr14	93151312	93151412
SMARCA2	chr9	2029138	2029254	RIN3	chr14	93151395	93151490
SMARCA2	chr9	2032876	2033001	RIN3	chr14	93151469	93151563
SMARCA2	chr9	2033000	2033116	RIN3	chr14	93154166	93154279

SMARCA2	chr9	2039379	2039506	RIN3	chr14	93154277	93154369
SMARCA2	chr9	2039497	2039627	RIN3	chr14	93154476	93154578
SMARCA2	chr9	2039627	2039736	RIN3	chr14	93154598	93154690
SMARCA2	chr9	2039892	2040002	SPHKAP	chr2	228846386	228846507
SMARCA2	chr9	2047433	2047566	SPHKAP	chr2	228846496	228846618
SMARCA2	chr9	2054507	2054619	SPHKAP	chr2	228855613	228855729
SMARCA2	chr9	2054619	2054702	SPHKAP	chr2	228855718	228855830
SMARCA2	chr9	2054702	2054780	SPHKAP	chr2	228855789	228855910
SMARCA2	chr9	2056663	2056791	SPHKAP	chr2	228855945	228856065
SMARCA2	chr9	2056791	2056900	SPHKAP	chr2	228858229	228858356
SMARCA2	chr9	2058251	2058370	SPHKAP	chr2	228860146	228860271
SMARCA2	chr9	2058367	2058480	SPHKAP	chr2	228860265	228860393
SMARCA2	chr9	2060767	2060880	SPHKAP	chr2	228860393	228860476
SMARCA2	chr9	2060880	2060991	SPHKAP	chr2	228881079	228881191
SMARCA2	chr9	2070370	2070500	SPHKAP	chr2	228881180	228881292
SMARCA2	chr9	2073192	2073292	SPHKAP	chr2	228881281	228881400
SMARCA2	chr9	2073286	2073388	SPHKAP	chr2	228881389	228881466
SMARCA2	chr9	2073553	2073687	SPHKAP	chr2	228881455	228881557
SMARCA2	chr9	2076173	2076246	SPHKAP	chr2	228881543	228881648
SMARCA2	chr9	2076246	2076349	SPHKAP	chr2	228881637	228881751
SMARCA2	chr9	2077606	2077733	SPHKAP	chr2	228881740	228881838
SMARCA2	chr9	2077733	2077820	SPHKAP	chr2	228881824	228881954
SMARCA2	chr9	2081775	2081896	SPHKAP	chr2	228881950	228882074
SMARCA2	chr9	2081896	2082002	SPHKAP	chr2	228882063	228882183
SMARCA2	chr9	2083257	2083364	SPHKAP	chr2	228882172	228882298
SMARCA2	chr9	2083364	2083467	SPHKAP	chr2	228882285	228882402
SMARCA2	chr9	2083996	2084125	SPHKAP	chr2	228882432	228882549
SMARCA2	chr9	2084125	2084235	SPHKAP	chr2	228882538	228882647
SMARCA2	chr9	2086773	2086892	SPHKAP	chr2	228882636	228882720
SMARCA2	chr9	2086892	2087007	SPHKAP	chr2	228882709	228882808
SMARCA2	chr9	2087007	2087103	SPHKAP	chr2	228882797	228882924
SMARCA2	chr9	2088423	2088523	SPHKAP	chr2	228882913	228883033
SMARCA2	chr9	2088514	2088598	SPHKAP	chr2	228883022	228883110
SMARCA2	chr9	2088598	2088672	SPHKAP	chr2	228883099	228883201
SMARCA2	chr9	2096647	2096774	SPHKAP	chr2	228883190	228883271
SMARCA2	chr9	2097303	2097423	SPHKAP	chr2	228883256	228883357
SMARCA2	chr9	2097414	2097486	SPHKAP	chr2	228883346	228883429
SMARCA2	chr9	2101507	2101582	SPHKAP	chr2	228883418	228883533
SMARCA2	chr9	2101581	2101652	SPHKAP	chr2	228883522	228883628
SMARCA2	chr9	2103946	2104050	SPHKAP	chr2	228883617	228883739

SMARCA2	chr9	2104050	2104176	SPHKAP	chr2	228883701	228883823
SMARCA2	chr9	2110211	2110323	SPHKAP	chr2	228883847	228883926
SMARCA2	chr9	2110323	2110447	SPHKAP	chr2	228883912	228884030
SMARCA2	chr9	2115779	2115885	SPHKAP	chr2	228884019	228884127
SMARCA2	chr9	2115880	2115968	SPHKAP	chr2	228884116	228884229
SMARCA2	chr9	2115961	2116065	SPHKAP	chr2	228884218	228884302
SMARCA2	chr9	2119436	2119555	SPHKAP	chr2	228884291	228884405
SMARCA2	chr9	2123691	2123813	SPHKAP	chr2	228884394	228884471
SMARCA2	chr9	2123813	2123944	SPHKAP	chr2	228884460	228884560
SMARCA2	chr9	2161672	2161794	SPHKAP	chr2	228884549	228884671
SMARCA2	chr9	2161792	2161908	SPHKAP	chr2	228884660	228884789
SMARCA2	chr9	2170306	2170431	SPHKAP	chr2	228884778	228884893
SMARCA2	chr9	2170443	2170552	SPHKAP	chr2	228886377	228886498
SMARCA2	chr9	2181516	2181584	SPHKAP	chr2	228886487	228886600
SMARCA2	chr9	2181586	2181715	SPHKAP	chr2	228886589	228886692
SMARCA2	chr9	2182108	2182236	SPHKAP	chr2	228890061	228890161
SMARCA2	chr9	2182236	2182356	SPHKAP	chr2	228890150	228890271
SMARCA2	chr9	2186029	2186128	SPHKAP	chr2	228892172	228892284
SMARCA2	chr9	2186128	2186238	SPHKAP	chr2	228973490	228973558
SMARCA2	chr9	2191245	2191372	SPHKAP	chr2	228973547	228973665
SMARCA2	chr9	2191372	2191483	SPHKAP	chr2	228996569	228996699
SMARCA2	chr9	2192636	2192760	SPHKAP	chr2	228996695	228996812
SMARCA3	chr3	148749925	148750031	SPHKAP	chr2	229046228	229046359
SMARCA3	chr3	148750007	148750120	TDRD7	chr9	100190710	100190782
SMARCA3	chr3	148750109	148750228	TDRD7	chr9	100190771	100190866
SMARCA3	chr3	148752607	148752727	TDRD7	chr9	100190855	100190975
SMARCA3	chr3	148752726	148752793	TDRD7	chr9	100193151	100193270
SMARCA3	chr3	148752782	148752869	TDRD7	chr9	100193259	100193367
SMARCA3	chr3	148756843	148756915	TDRD7	chr9	100194294	100194365
SMARCA3	chr3	148756904	148756986	TDRD7	chr9	100194354	100194443
SMARCA3	chr3	148756975	148757086	TDRD7	chr9	100194432	100194530
SMARCA3	chr3	148757283	148757384	TDRD7	chr9	100201461	100201578
SMARCA3	chr3	148757373	148757457	TDRD7	chr9	100201574	100201666
SMARCA3	chr3	148757443	148757558	TDRD7	chr9	100203912	100203992
SMARCA3	chr3	148757711	148757829	TDRD7	chr9	100203977	100204047
SMARCA3	chr3	148757818	148757901	TDRD7	chr9	100204141	100204245
SMARCA3	chr3	148757946	148758069	TDRD7	chr9	100222412	100222533
SMARCA3	chr3	148759199	148759267	TDRD7	chr9	100222522	100222648
SMARCA3	chr3	148759256	148759326	TDRD7	chr9	100222637	100222749
SMARCA3	chr3	148759315	148759420	TDRD7	chr9	100222738	100222860

SMARCA3	chr3	148759409	148759512	TDRD7	chr9	100222850	100222979
SMARCA3	chr3	148759849	148759966	TDRD7	chr9	100222969	100223083
SMARCA3	chr3	148759955	148760023	TDRD7	chr9	100227071	100227183
SMARCA3	chr3	148763800	148763912	TDRD7	chr9	100227172	100227276
SMARCA3	chr3	148763901	148764023	TDRD7	chr9	100227241	100227318
SMARCA3	chr3	148764006	148764096	TDRD7	chr9	100232755	100232879
SMARCA3	chr3	148765672	148765791	TDRD7	chr9	100232868	100232970
SMARCA3	chr3	148765780	148765900	TDRD7	chr9	100234515	100234598
SMARCA3	chr3	148765889	148766000	TDRD7	chr9	100234587	100234679
SMARCA3	chr3	148766569	148766673	TDRD7	chr9	100234668	100234787
SMARCA3	chr3	148766662	148766760	TDRD7	chr9	100235698	100235807
SMARCA3	chr3	148766759	148766880	TDRD7	chr9	100235796	100235918
SMARCA3	chr3	148767952	148768052	TDRD7	chr9	100237600	100237725
SMARCA3	chr3	148768027	148768131	TDRD7	chr9	100237714	100237830
SMARCA3	chr3	148768111	148768226	TDRD7	chr9	100240660	100240774
SMARCA3	chr3	148772992	148773109	TDRD7	chr9	100240763	100240872
SMARCA3	chr3	148773098	148773201	TDRD7	chr9	100243002	100243125
SMARCA3	chr3	148773192	148773285	TDRD7	chr9	100243114	100243230
SMARCA3	chr3	148777377	148777489	TDRD7	chr9	100245098	100245182
SMARCA3	chr3	148777505	148777573	TDRD7	chr9	100245171	100245250
SMARCA3	chr3	148777562	148777680	TDRD7	chr9	100245239	100245359
SMARCA3	chr3	148778374	148778492	TDRD7	chr9	100245348	100245465
SMARCA3	chr3	148778476	148778591	TDRD7	chr9	100245448	100245557
SMARCA3	chr3	148778580	148778679	TDRD7	chr9	100245546	100245662
SMARCA3	chr3	148778684	148778767	TDRD7	chr9	100249405	100249511
SMARCA3	chr3	148781160	148781258	TDRD7	chr9	100249500	100249624
SMARCA3	chr3	148781247	148781361	TDRD7	chr9	100257908	100258027
SMARCA3	chr3	148782493	148782606	TDRD7	chr9	100258015	100258127
SMARCA3	chr3	148782595	148782711	TDRD7	chr9	100258118	100258233
SMARCA3	chr3	148785923	148786043	THSD7B	chr2	137748419	137748544
SMARCA3	chr3	148786032	148786136	THSD7B	chr2	137813954	137814075
SMARCA3	chr3	148788973	148789076	THSD7B	chr2	137814064	137814150
SMARCA3	chr3	148789065	148789175	THSD7B	chr2	137814135	137814216
SMARCA3	chr3	148789164	148789280	THSD7B	chr2	137814206	137814315
SMARCA3	chr3	148789314	148789425	THSD7B	chr2	137814304	137814416
SMARCA3	chr3	148789407	148789521	THSD7B	chr2	137814405	137814525
SMARCA3	chr3	148790930	148791007	THSD7B	chr2	137814515	137814594
SMARCA3	chr3	148790969	148791082	THSD7B	chr2	137814583	137814658
SMARCA3	chr3	148791072	148791171	THSD7B	chr2	137814647	137814762
SMARCA3	chr3	148791912	148792032	THSD7B	chr2	137814751	137814867

SMARCA3	chr3	148792021	148792141	THSD7B	chr2	137852396	137852484
SMARCA3	chr3	148792130	148792237	THSD7B	chr2	137852473	137852589
SMARCA3	chr3	148793570	148793692	THSD7B	chr2	137852578	137852701
SMARCA3	chr3	148793681	148793791	THSD7B	chr2	137872594	137872715
SMARCA3	chr3	148793780	148793889	THSD7B	chr2	137872704	137872811
SMARCA3	chr3	148802407	148802523	THSD7B	chr2	137917732	137917858
SMARCA3	chr3	148802512	148802623	THSD7B	chr2	137917850	137917960
SMARCA3	chr3	148802612	148802727	THSD7B	chr2	137928268	137928369
SMARCA3	chr3	148804051	148804183	THSD7B	chr2	137928352	137928469
SMARCA4	chr19	11094701	11094824	THSD7B	chr2	137928458	137928537
SMARCA4	chr19	11094877	11095009	THSD7B	chr2	137988600	137988722
SMARCA4	chr19	11095028	11095119	THSD7B	chr2	137988689	137988771
SMARCA4	chr19	11095870	11095990	THSD7B	chr2	137990390	137990520
SMARCA4	chr19	11095972	11096098	THSD7B	chr2	137990509	137990632
SMARCA4	chr19	11096090	11096167	THSD7B	chr2	137990621	137990714
SMARCA4	chr19	11096767	11096901	THSD7B	chr2	137999974	138000094
SMARCA4	chr19	11096903	11097018	THSD7B	chr2	138000083	138000157
SMARCA4	chr19	11097039	11097167	THSD7B	chr2	138030072	138030197
SMARCA4	chr19	11097169	11097291	THSD7B	chr2	138030186	138030285
SMARCA4	chr19	11097274	11097389	THSD7B	chr2	138033456	138033533
SMARCA4	chr19	11097486	11097571	THSD7B	chr2	138033522	138033634
SMARCA4	chr19	11097561	11097676	THSD7B	chr2	138163088	138163211
SMARCA4	chr19	11097701	11097786	THSD7B	chr2	138163200	138163287
SMARCA4	chr19	11098170	11098306	THSD7B	chr2	138163276	138163392
SMARCA4	chr19	11098528	11098657	THSD7B	chr2	138169139	138169257
SMARCA4	chr19	11099892	11100010	THSD7B	chr2	138169246	138169374
SMARCA4	chr19	11099971	11100101	THSD7B	chr2	138169362	138169453
SMARCA4	chr19	11100089	11100177	THSD7B	chr2	138208358	138208449
SMARCA4	chr19	11101749	11101880	THSD7B	chr2	138208438	138208562
SMARCA4	chr19	11101824	11101957	THSD7B	chr2	138208551	138208659
SMARCA4	chr19	11101951	11102071	THSD7B	chr2	138320702	138320823
SMARCA4	chr19	11105426	11105507	THSD7B	chr2	138320812	138320924
SMARCA4	chr19	11105496	11105626	THSD7B	chr2	138320913	138320988
SMARCA4	chr19	11105589	11105705	THSD7B	chr2	138329871	138329982
SMARCA4	chr19	11105694	11105780	THSD7B	chr2	138329974	138330078
SMARCA4	chr19	11106750	11106874	THSD7B	chr2	138330067	138330140
SMARCA4	chr19	11106854	11106947	THSD7B	chr2	138373722	138373844
SMARCA4	chr19	11106935	11107041	THSD7B	chr2	138373830	138373913
SMARCA4	chr19	11107117	11107251	THSD7B	chr2	138375933	138376059
SMARCA4	chr19	11107242	11107319	THSD7B	chr2	138376045	138376119

SMARCA4	chr19	11113608	11113740	THSD7B	chr2	138378088	138378199
SMARCA4	chr19	11113729	11113836	THSD7B	chr2	138378189	138378307
SMARCA4	chr19	11113788	11113911	THSD7B	chr2	138399994	138400070
SMARCA4	chr19	11113928	11114007	THSD7B	chr2	138400059	138400179
SMARCA4	chr19	11113996	11114123	THSD7B	chr2	138400145	138400232
SMARCA4	chr19	11118506	11118629	THSD7B	chr2	138412961	138413085
SMARCA4	chr19	11118565	11118661	THSD7B	chr2	138413073	138413196
SMARCA4	chr19	11120966	11121071	THSD7B	chr2	138413182	138413283
SMARCA4	chr19	11121060	11121174	THSD7B	chr2	138414354	138414480
SMARCA4	chr19	11121163	11121291	THSD7B	chr2	138414469	138414549
SMARCA4	chr19	11123560	11123651	THSD7B	chr2	138414538	138414663
SMARCA4	chr19	11123640	11123764	THSD7B	chr2	138414652	138414740
SMARCA4	chr19	11123751	11123838	THSD7B	chr2	138417220	138417329
SMARCA4	chr19	11129509	11129603	THSD7B	chr2	138420935	138421053
SMARCA4	chr19	11129592	11129712	THSD7B	chr2	138421045	138421166
SMARCA4	chr19	11129701	11129785	THSD7B	chr2	138425307	138425410
SMARCA4	chr19	11130103	11130232	THSD7B	chr2	138425401	138425487
SMARCA4	chr19	11130250	11130339	THSD7B	chr2	138434073	138434195
SMARCA4	chr19	11130328	11130457	TSC1	chr9	135771531	135771657
SMARCA4	chr19	11132339	11132428	TSC1	chr9	135771646	135771773
SMARCA4	chr19	11132417	11132526	TSC1	chr9	135771764	135771861
SMARCA4	chr19	11132490	11132598	TSC1	chr9	135771851	135771977
SMARCA4	chr19	11132586	11132693	TSC1	chr9	135771943	135772072
SMARCA4	chr19	11134136	11134267	TSC1	chr9	135772032	135772121
SMARCA4	chr19	11134230	11134333	TSC1	chr9	135772110	135772188
SMARCA4	chr19	11134322	11134449	TSC1	chr9	135772531	135772661
SMARCA4	chr19	11134912	11135009	TSC1	chr9	135772650	135772766
SMARCA4	chr19	11134998	11135101	TSC1	chr9	135772742	135772837
SMARCA4	chr19	11135093	11135192	TSC1	chr9	135772826	135772954
SMARCA4	chr19	11135975	11136108	TSC1	chr9	135772943	135773044
SMARCA4	chr19	11136089	11136220	TSC1	chr9	135776005	135776123
SMARCA4	chr19	11136183	11136276	TSC1	chr9	135776112	135776234
SMARCA4	chr19	11136900	11137010	TSC1	chr9	135776952	135777055
SMARCA4	chr19	11136995	11137078	TSC1	chr9	135777044	135777162
SMARCA4	chr19	11138396	11138501	TSC1	chr9	135777880	135778005
SMARCA4	chr19	11138490	11138574	TSC1	chr9	135777994	135778091
SMARCA4	chr19	11138563	11138681	TSC1	chr9	135778080	135778184
SMARCA4	chr19	11141344	11141463	TSC1	chr9	135778995	135779116
SMARCA4	chr19	11141452	11141534	TSC1	chr9	135779051	135779140
SMARCA4	chr19	11141523	11141619	TSC1	chr9	135779738	135779858

SMARCA4	chr19	11143907	11144039	TSC1	chr9	135780946	135781054
SMARCA4	chr19	11144021	11144112	TSC1	chr9	135781031	135781122
SMARCA4	chr19	11144101	11144223	TSC1	chr9	135781111	135781235
SMARCA4	chr19	11144197	11144290	TSC1	chr9	135781224	135781351
SMARCA4	chr19	11144315	11144414	TSC1	chr9	135781328	135781429
SMARCA4	chr19	11144398	11144518	TSC1	chr9	135781462	135781564
SMARCA4	chr19	11144573	11144703	TSC1	chr9	135782009	135782129
SMARCA4	chr19	11144711	11144817	TSC1	chr9	135782118	135782232
SMARCA4	chr19	11144806	11144932	TSC1	chr9	135782661	135782783
SMARCA4	chr19	11145535	11145668	TSC1	chr9	135785863	135785987
SMARCA4	chr19	11145687	11145802	TSC1	chr9	135785952	135786078
SMARCA4	chr19	11145788	11145898	TSC1	chr9	135786072	135786167
SMARCA4	chr19	11150062	11150135	TSC1	chr9	135786278	135786403
SMARCA4	chr19	11150121	11150206	TSC1	chr9	135786392	135786510
SMARCA4	chr19	11150195	11150295	TSC1	chr9	135786782	135786906
SMARCA4	chr19	11151839	11151960	TSC1	chr9	135786895	135786981
SMARCA4	chr19	11151949	11152044	TSC1	chr9	135787643	135787765
SMARCA4	chr19	11152038	11152170	TSC1	chr9	135787754	135787857
SMARCA4	chr19	11152166	11152295	TSC1	chr9	135796714	135796833
SMARCA4	chr19	11168855	11168979	TSC1	chr9	135797140	135797267
SMARCA4	chr19	11168968	11169102	TSC1	chr9	135797256	135797375
SMARCA4	chr19	11169361	11169468	TSC1	chr9	135798657	135798772
SMARCA4	chr19	11169447	11169530	TSC1	chr9	135798761	135798889
SMARCA4	chr19	11169513	11169634	TSC1	chr9	135800912	135801028
SMARCA4	chr19	11170321	11170443	TSC1	chr9	135801017	135801137
SMARCA4	chr19	11170432	11170554	TSC1	chr9	135802545	135802665
SMARCA4	chr19	11170556	11170686	TSC1	chr9	135802654	135802732
SMARCA4	chr19	11170702	11170818	TSC1	chr9	135804114	135804238
SMARCA4	chr19	11170738	11170847	TSC1	chr9	135804172	135804295
SMARCA4	chr19	11172369	11172477	TSC2	chr16	2098563	2098652
SMARCA4	chr19	11172474	11172557	TSC2	chr16	2098641	2098764
SMARCA4	chr19	11094812	11094945	TSC2	chr16	2100286	2100410
SMARCA4	chr19	11094907	11095037	TSC2	chr16	2100399	2100498
SMARCA4	chr19	11095033	11095141	TSC2	chr16	2103239	2103374
SMARCA4	chr19	11095876	11096008	TSC2	chr16	2103363	2103496
SMARCA4	chr19	11096008	11096102	TSC2	chr16	2104240	2104344
SMARCA4	chr19	11096820	11096939	TSC2	chr16	2104333	2104458
SMARCA4	chr19	11096931	11097067	TSC2	chr16	2105365	2105464
SMARCA4	chr19	11097063	11097178	TSC2	chr16	2105453	2105530
SMARCA4	chr19	11097172	11097291	TSC2	chr16	2106110	2106230

SMARCA4	chr19	11097561	11097690	TSC2	chr16	2106214	2106310
SMARCA4	chr19	11098335	11098455	TSC2	chr16	2106577	2106696
SMARCA4	chr19	11098390	11098524	TSC2	chr16	2106685	2106780
SMARCA4	chr19	11098579	11098679	TSC2	chr16	2107016	2107145
SMARCA4	chr19	11099892	11100023	TSC2	chr16	2107123	2107252
SMARCA4	chr19	11100023	11100155	TSC2	chr16	2108732	2108862
SMARCA4	chr19	11101814	11101952	TSC2	chr16	2108853	2108973
SMARCA4	chr19	11101952	11102078	TSC2	chr16	2110567	2110698
SMARCA4	chr19	11105487	11105568	TSC2	chr16	2110687	2110769
SMARCA4	chr19	11105568	11105692	TSC2	chr16	2110750	2110872
SMARCA4	chr19	11106872	11106966	TSC2	chr16	2111854	2111964
SMARCA4	chr19	11106966	11107097	TSC2	chr16	2111952	2112079
SMARCA4	chr19	11107119	11107255	TSC2	chr16	2112369	2112503
SMARCA4	chr19	11113608	11113710	TSC2	chr16	2112492	2112612
SMARCA4	chr19	11113709	11113836	TSC2	chr16	2113055	2113185
SMARCA4	chr19	11113831	11113917	TSC2	chr16	2114211	2114321
SMARCA4	chr19	11113991	11114121	TSC2	chr16	2114301	2114415
SMARCA4	chr19	11118569	11118658	TSC2	chr16	2114404	2114507
SMARCA4	chr19	11118658	11118764	TSC2	chr16	2115461	2115588
SMARCA4	chr19	11121015	11121142	TSC2	chr16	2115569	2115694
SMARCA4	chr19	11121141	11121239	TSC2	chr16	2120424	2120554
SMARCA4	chr19	11123577	11123701	TSC2	chr16	2121498	2121595
SMARCA4	chr19	11123694	11123789	TSC2	chr16	2121584	2121681
SMARCA4	chr19	11129592	11129725	TSC2	chr16	2121704	2121799
SMARCA4	chr19	11130251	11130338	TSC2	chr16	2121797	2121922
SMARCA4	chr19	11130336	11130464	TSC2	chr16	2122181	2122293
SMARCA4	chr19	11132349	11132439	TSC2	chr16	2122264	2122351
SMARCA4	chr19	11132433	11132557	TSC2	chr16	2122729	2122859
SMARCA4	chr19	11132548	11132665	TSC2	chr16	2122848	2122959
SMARCA4	chr19	11134182	11134317	TSC2	chr16	2122948	2123053
SMARCA4	chr19	11134996	11135096	TSC2	chr16	2124094	2124209
SMARCA4	chr19	11135094	11135192	TSC2	chr16	2124214	2124318
SMARCA4	chr19	11136088	11136220	TSC2	chr16	2124307	2124412
SMARCA4	chr19	11136951	11137059	TSC2	chr16	2125863	2125997
SMARCA4	chr19	11138421	11138505	TSC2	chr16	2126001	2126096
SMARCA4	chr19	11138505	11138631	TSC2	chr16	2126085	2126187
SMARCA4	chr19	11141347	11141469	TSC2	chr16	2126404	2126532
SMARCA4	chr19	11141469	11141588	TSC2	chr16	2126521	2126653
SMARCA4	chr19	11143958	11144091	TSC2	chr16	2127494	2127618
SMARCA4	chr19	11144088	11144224	TSC2	chr16	2127607	2127714

SMARCA4	chr19	11144781	11144902
SMARCA4	chr19	11145515	11145649
SMARCA4	chr19	11145604	11145730
SMARCA4	chr19	11145729	11145864
SMARCA4	chr19	11151960	11152050
SMARCA4	chr19	11152047	11152169
SMARCA4	chr19	11152165	11152291
SMARCA4	chr19	11168921	11169047
SMARCA4	chr19	11169446	11169537
SMARCA4	chr19	11169535	11169634
SMARCA4	chr19	11170419	11170558
SMARCA4	chr19	11170665	11170758
SMARCA4	chr19	11170758	11170853
SMARCA4	chr19	11172408	11172544
TSC2	chr16	2098562	2098648
TSC2	chr16	2098648	2098764
TSC2	chr16	2100380	2100507
TSC2	chr16	2103212	2103343
TSC2	chr16	2103341	2103468
TSC2	chr16	2104243	2104346
TSC2	chr16	2104346	2104464
TSC2	chr16	2105325	2105456
TSC2	chr16	2105456	2105532
TSC2	chr16	2106130	2106232
TSC2	chr16	2106595	2106723
TSC2	chr16	2106722	2106829
TSC2	chr16	2107094	2107206
TSC2	chr16	2108734	2108865
TSC2	chr16	2108865	2108973
TSC2	chr16	2110606	2110745
TSC2	chr16	2110745	2110871
TSC2	chr16	2111854	2111972
TSC2	chr16	2111972	2112070
TSC2	chr16	2112487	2112614
TSC2	chr16	2112921	2113018
TSC2	chr16	2114213	2114327
TSC2	chr16	2114327	2114454
TSC2	chr16	2115463	2115587
TSC2	chr16	2115578	2115696
TSC2	chr16	2120424	2120528
TSC2	chr16	2127713	2127805
TSC2	chr16	2128962	2129069
TSC2	chr16	2129042	2129166
TSC2	chr16	2129155	2129253
TSC2	chr16	2129259	2129378
TSC2	chr16	2129347	2129430
TSC2	chr16	2129547	2129658
TSC2	chr16	2129588	2129714
TSC2	chr16	2130122	2130227
TSC2	chr16	2130188	2130292
TSC2	chr16	2130281	2130401
TSC2	chr16	2131576	2131673
TSC2	chr16	2131662	2131787
TSC2	chr16	2131742	2131864
TSC2	chr16	2132387	2132489
TSC2	chr16	2132467	2132591
TSC2	chr16	2133629	2133766
TSC2	chr16	2133772	2133904
TSC2	chr16	2134145	2134250
TSC2	chr16	2134242	2134373
TSC2	chr16	2134467	2134604
TSC2	chr16	2134662	2134761
TSC2	chr16	2134956	2135050
TSC2	chr16	2135242	2135377
TSC2	chr16	2136098	2136231
TSC2	chr16	2136222	2136344
TSC2	chr16	2136368	2136503
TSC2	chr16	2136664	2136760
TSC2	chr16	2136746	2136829
TSC2	chr16	2136818	2136947
TSC2	chr16	2137783	2137903
TSC2	chr16	2137892	2138025
TSC2	chr16	2137946	2138080
TSC2	chr16	2138076	2138183
TSC2	chr16	2138201	2138318
TSC2	chr16	2138295	2138401
TSC2	chr16	2138459	2138585
TSC2	chr16	2138554	2138639

TSC2	chr16	2120524	2120652
TSC2	chr16	2121502	2121596
TSC2	chr16	2121593	2121681
TSC2	chr16	2121704	2121792
TSC2	chr16	2121791	2121929
TSC2	chr16	2121928	2122044
TSC2	chr16	2122197	2122322
TSC2	chr16	2122320	2122414
TSC2	chr16	2122843	2122952
TSC2	chr16	2122952	2123055
TSC2	chr16	2124193	2124301
TSC2	chr16	2124301	2124422
TSC2	chr16	2125749	2125850
TSC2	chr16	2125849	2125939
TSC2	chr16	2126041	2126176
TSC2	chr16	2126481	2126573
TSC2	chr16	2126572	2126666
TSC2	chr16	2127522	2127652
TSC2	chr16	2127652	2127752
TSC2	chr16	2128975	2129063
TSC2	chr16	2129052	2129165
TSC2	chr16	2129165	2129254
TSC2	chr16	2129267	2129379
TSC2	chr16	2129367	2129505
TSC2	chr16	2129547	2129685
TSC2	chr16	2130130	2130259
TSC2	chr16	2130243	2130354
TSC2	chr16	2130340	2130448
TSC2	chr16	2131554	2131636
TSC2	chr16	2131635	2131735
TSC2	chr16	2131734	2131855
TSC2	chr16	2132412	2132545
TSC2	chr16	2133688	2133818
TSC2	chr16	2133816	2133918
TSC2	chr16	2134144	2134246
TSC2	chr16	2134241	2134367
TSC2	chr16	2134353	2134489
TSC2	chr16	2134487	2134625
TSC2	chr16	2134619	2134745
TSC2	chr16	2134882	2134996

TSC2	chr16	2134996	2135133
TSC2	chr16	2135221	2135343
TSC2	chr16	2136169	2136272
TSC2	chr16	2136252	2136371
TSC2	chr16	2136371	2136504
TSC2	chr16	2136698	2136826
TSC2	chr16	2136826	2136955
TSC2	chr16	2137783	2137899
TSC2	chr16	2137896	2137998
TSC2	chr16	2138013	2138154
TSC2	chr16	2138197	2138334
TSC2	chr16	2138421	2138519
TSC2	chr16	2138507	2138639
