# Supplementary material for: Lung neuroendocrine tumours: deep sequencing of the four World Health Organization histotypes reveals chromatin‐remodelling genes as major players and a prognostic role for TERT, RB1, MEN1 and KMT2D
Source: J Pathol. 2016 Dec 29;241(4):488–500. doi: 10.1002/path.4853 (PMC5324596; doi:10.1002/path.4853)
Supplement: Supplementary file 7 — Table S2. Clinicopathological features of 148 lung neuroendocrine tumours and analyses executed. [file PATH-241-488-s009.docx]

**Supplementary Table S2.** Clinicopathological features of 148 lung neuroendocrine tumours and analyses executed.

| **ID** | **S** | **A** | **His** | **Size** | **T** | **N** | **M** | **Stage** | **Ncr** | **V. inv** | **P. inv** | **WES** | **HCTS** | **CNA** | **TS** |
| --- | --- | --- | --- | --- | --- | --- | --- | --- | --- | --- | --- | --- | --- | --- | --- |
| 209 | F | 30 | TC | 3 | 1 | 0 | 0 | 1a | 0 | 0 | 0 | X | X | X | X |
| 349 | F | 45 | TC | 2.5 | 1 | 0 | 0 | 1a | 0 | 0 | 0 | X | X | X | X |
| 351 | F | 76 | TC | 2.5 | 1 | 0 | 0 | 1a | 0 | 1 | 0 | X | X | X | X |
| 243 | F | 34 | TC | 3 | 1 | 0 | 0 | 1a | 0 | 0 | 0 | X | X | X | X |
| 245 | M | 35 | TC | 3.2 | 2 | 0 | 0 | 1b | 0 | 0 | 0 | X | X | X | X |
| 304 | M | 32 | TC | 2.1 | 1 | 0 | 0 | 1a | 0 | 0 | 0 | X | X | X | X |
| 384 | F | 52 | TC | 0.7 | 1 | 0 | 0 | 1a | 0 | 0 | 0 | X | X | X | X |
| 402 | M | 39 | TC | 3.2 | 2 | 1 | 0 | 2a | 0 | 0 | 0 | X | X | X | X |
| 497 | F | 52 | TC | 2.8 | 1 | 0 | 0 | 1a | 0 | 0 | 0 | X | X | X | X |
| 467 | M | 72 | TC | 2.5 | 1 | 0 | 0 | 1a | 0 | 0 | 0 | X | X | X | X |
| 2 | M | 59 | TC | 1.5 | 1 | 0 | 0 | 1 | 0 | 0 | 0 |  |  | X | X |
| 4 | M | 53 | TC | 1.5 | 1 | 0 | 0 | 1 | 0 | 0 | 0 |  |  | X | X |
| 8 | F | 47 | TC | 2.8 | 1 | 0 | 0 | 1 | 0 | 0 | 0 |  |  | X | X |
| 11 | M | 54 | TC | 1.5 | 1 | 0 | 0 | 1 | 0 | 1 | 0 |  |  | X | X |
| 14 | F | 37 | TC | 7 | 2 | 0 | 0 | 2 | 0 | 1 | 0 |  |  | X | X |
| 15 | F | 17 | TC | 5 | 2 | 0 | 0 | 1 | 0 | 0 | 0 |  |  | X | X |
| 17 | M | 61 | TC | 1 | 1 | 1 | 0 | 1a | 0 | 0 | 0 |  |  | X | X |
| 18 | M | 43 | TC | 3 | 1 | 0 | 0 | 1 | 0 | 0 | 0 |  |  | X | X |
| 19 | F | 71 | TC | 1 | 1 | 0 | 0 | 1 | 0 | 0 | 0 |  |  | X | X |
| 21 | F | 45 | TC | 2.4 | 1 | 0 | 0 | 1 | 0 | 0 | 0 |  |  | X | X |
| 22 | F | 35 | TC | 1.7 | 1 | 0 | 0 | 1 | 0 | 0 | 0 |  |  | X | X |
| 26 | F | 55 | TC | 5 | 2 | 0 | 0 | 1 | 0 | 0 | 0 |  |  | X | X |
| 28 | M | 65 | TC | 3.3 | 2 | 0 | 0 | 1 | 0 | 0 | 0 |  |  | X | X |
| 29 | F | 23 | TC | 4 | 2 | 0 | 0 | 1 | 0 | 0 | 0 |  |  | X | X |
| 31 | M | 72 | TC | 1.3 | 1 | 0 | 0 | 1 | 0 | 0 | 0 |  |  | X | X |
| 32 | M | 49 | TC | 6.2 | 2 | 0 | 0 | 1 | 0 | 0 | 0 |  |  | X | X |
| 33 | M | 57 | TC | 3 | 1 | 0 | 0 | 1 | 0 | 0 | 0 |  |  | X | X |
| 34 | F | 55 | TC | 4 | 2 | 0 | 0 | 1 | 0 | 0 | 0 |  |  | X | X |
| 39 | M | 56 | TC | 2.5 | 1 | 0 | 0 | 1b | 0 | 0 | 0 |  |  | X | X |
| 41 | F | 63 | TC | 3 | 1 | 0 | 0 | 1a | 0 | 0 | 0 |  |  | X | X |
| 42 | F | 61 | TC | 5 | 2 | 0 | 0 | 1b | 0 | 0 | 0 |  |  | X | X |
| 66 | F | 77 | TC | 1 | 2 | 0 | 0 | 1b | 0 | 0 | 0 |  |  | X | X |
| 67 | M | 71 | TC | 2.7 | 1 | 0 | 0 | 1a | 0 | 0 | 0 |  |  | X | X |
| 68 | M | 65 | TC | 0.7 | 1 | 0 | 0 | 1a | 0 | 0 | 0 |  |  | X | X |
| 70 | F | 73 | TC | 2.2 | 1 | 0 | 0 | 1a | 0 | 0 | 0 |  |  | X | X |
| 71 | F | 35 | TC | 2.8 | 1 | 0 | 0 | 1a | 0 | 0 | 0 |  |  | X | X |
| 72 | F | 66 | TC | 3.8 | 2 | 0 | 0 | 1b | 0 | 0 | 0 |  |  | X | X |
| 73 | F | 77 | TC | 2.7 | 1 | 0 | 0 | 1a | 0 | 0 | 0 |  |  | X | X |
| 74 | M | 64 | TC | 1.8 | 1 | 0 | 0 | 1a | 0 | 0 | 0 |  |  | X | X |
| 75 | F | 80 | TC | 2.2 | 1 | 0 | 0 | 1a | 0 | 0 | 0 |  |  | X | X |
| 609 | F | 72 | TC | 1.6 | 1 | 0 | 0 | 1 | NA | 0 | 0 |  | X | X | X |
| 018 | F | 53 | TC | 1.5 | 1 | 0 | 0 | 1a | 0 | 0 | 0 |  | X | X | X |
| 057 | F | 28 | TC | 5 | 2 | 1 | 0 | 2a | 0 | 0 | 0 |  | X | X | X |
| 261 | M | 62 | TC | 1.2 | 1 | 0 | 0 | 1a | 0 | 0 | 0 |  | X | X | X |
| 017 | F | 59 | TC | 2 | 1 | 0 | 0 | 1a | 0 | 0 | 0 |  | X | X | X |
| 154 | M | 31 | TC | 3.3 | 2 | 0 | 0 | 1b | 0 | 0 | 0 |  | X | X | X |
| 291 | F | 62 | TC | 1.8 | 3 | 1 | 0 | 3a | 0 | 1 | 1 |  | X | X | X |
| 419 | F | 50 | TC | 2.5 | 1 | 0 | 0 | 1a | 0 | 0 | 0 |  | X | X | X |
| 393 | M | 62 | TC | 4 | 2 | 0 | 0 | 1b | 0 | 0 | 0 |  | X | X | X |
| 549 | F | 64 | TC | 3.2 | 4 | 0 | 0 | 3a | 0 | 0 | 0 |  | X | X | X |
| 552 | F | 51 | TC | 2.3 | 1 | 0 | 0 | 1a | 0 | 0 | 0 |  | X | X | X |
| 608 | F | 57 | TC | 1.3 | 1 | 0 | 0 | 1a | 0 | 0 | 0 |  | X | X | X |
| 611 | F | 62 | TC | 1.7 | 3 | 1 | 0 | 3a | 0 | 0 | 0 |  | X | X | X |
| 034 | F | 75 | AC | 4 | 3 | 1 | 1 | 4 | 1 | 1 | 0 | X | X | X | X |
| 364 | F | 55 | AC | 3.2 | 2 | 0 | 0 | 1b | 1 | 0 | 0 | X | X | X | X |
| 389 | F | 83 | AC | 8.5 | 3 | 0 | 0 | 2b | 0 | 0 | 0 | X | X | X | X |
| 450 | F | 66 | AC | 2.2 | 1 | 0 | 0 | 1a | 0 | 0 | 0 | X | X | X | X |
| 13 | M | 67 | AC | 5.5 | 2 | 0 | 0 | 2 | NA | NA | 0 |  |  | X | X |
| 38 | F | 54 | AC | 2.5 | 2 | 1 | 0 | 2a | 0 | 0 | 0 |  |  | X | X |
| 77 | F | 47 | AC | 3.1 | 2 | 1 | 0 | 2a | NA | NA | NA |  |  | X | X |
| 78 | F | 70 | AC | 4.4 | 2 | 1 | 0 | 2a | NA | NA | NA |  |  | X | X |
| 79 | M | 79 | AC | 4.5 | 2 | 0 | 0 | 1b | NA | NA | NA |  |  | X | X |
| 80 | M | 79 | AC | 1.5 | 2 | 1 | 0 | 3a | NA | NA | NA |  |  | X | X |
| 81 | M | 53 | AC | 3.5 | 1 | 0 | 0 | 1a | NA | NA | NA |  |  | X | X |
| 82 | F | 66 | AC | 1.5 | 1 | 0 | 0 | 1a | NA | NA | NA |  |  | X | X |
| 83 | F | 78 | AC | 1.5 | 1 | 0 | 0 | 1a | NA | NA | NA |  |  | X | X |
| 84 | F | 72 | AC | 1.2 | 1 | 0 | 0 | 1a | NA | NA | NA |  |  | X | X |
| 85 | M | 20 | AC | 1.4 | 1 | 0 | 0 | 1a | NA | NA | NA |  |  | X | X |
| 86 | F | 56 | AC | 1.4 | 1 | 0 | 0 | 1a | NA | NA | NA |  |  | X | X |
| 87 | M | 65 | AC | 2.8 | 1 | 0 | 0 | 1a | NA | NA | NA |  |  | X | X |
| 88 | F | 51 | AC | 1.5 | 2 | 1 | 0 | 2a | NA | NA | NA |  |  | X | X |
| 89 | M | 51 | AC | 4.5 | 2 | 0 | 0 | 2b | NA | NA | NA |  |  | X | X |
| 90 | F | 63 | AC | 0.8 | 1 | 0 | 0 | 1a | NA | NA | NA |  |  | X | X |
| 91 | M | 65 | AC | 8.5 | 3 | 0 | 0 | 2b | NA | NA | NA |  |  | X | X |
| 92 | M | 75 | AC | 6.7 | 2 | 0 | 0 | 2a | NA | NA | NA |  |  | X | X |
| 93 | M | 64 | AC | 6.3 | 2 | 0 | 0 | 2a | NA | NA | NA |  |  | X | X |
| 94 | F | 26 | AC | 5 | 2 | 1 | 0 | 2b | NA | NA | NA |  |  | X | X |
| 95 | F | 42 | AC | 3.5 | 3 | 0 | 0 | 2b | NA | NA | NA |  |  | X | X |
| 502 | F | 66 | AC | 1.7 | 1 | 0 | 0 | 1 | 0 | 0 | 0 |  | X | X | X |
| 394 | M | 47 | AC | 1.5 | 1 | 0 | 0 | 1 | NA | NA | NA |  | X | X | X |
| 400 | M | 79 | AC | 2.4 | 1 | 0 | 0 | 1 | 1 | 0 | 0 |  | X | X | X |
| 166 | M | 59 | AC | 3.5 | 2 | 1 | 0 | 2a | 0 | 0 | 0 |  | X | X | X |
| 021 | M | 77 | AC | 2.4 | 3 | 1 | 0 | 3a | 1 | 1 | 1 |  | X | X | X |
| 288 | F | 55 | AC | 5 | 2 | 1 | 0 | 2a | 1 | 1 | 0 |  | X | X | X |
| 436 | F | 70 | AC | 4 | 2 | 0 | 0 | 1b | 0 | 0 | 0 |  | X | X | X |
| 563 | F | 35 | AC | 2 | 1 | 0 | 0 | 1a | 0 | 0 | 0 |  | X | X | X |
| 593 | M | 75 | AC | 1.8 | 1 | 0 | 0 | 1a | 0 | 0 | 0 |  | X | X | X |
| 607 | F | 56 | AC | 2.6 | 1 | 1 | 0 | 2a | 0 | 0 | 0 |  | X | X | X |
| 122 | M | 65 | LCNEC | 2 | 3 | 0 | 1 | 4 | 1 | 0 | 0 | X | X | X | X |
| 302 | M | 70 | LCNEC | 3.5 | 3 | 0 | 0 | 2b | 1 | 1 | 1 | X | X | X | X |
| 509 | M | 79 | LCNEC | 4 | 2 | 0 | 0 | 1b | 1 | 0 | 0 | X | X | X | X |
| 59 | F | 57 | LCNEC | NA | 2 | 1 | 1 | 4 | 1 | NA | NA |  |  | X | X |
| 65 | M | 78 | LCNEC | NA | 4 | 1 | 0 | 3b | 1 | NA | NA |  |  | X | X |
| 96 | M | 65 | LCNEC | 2.5 | 1 | 1 | 0 | 3a | 1 | NA | NA |  |  | X | X |
| 97 | F | 73 | LCNEC | 3 | 1 | 0 | 0 | 1a | 1 | NA | NA |  |  | X | X |
| 98 | M | 84 | LCNEC | 1.8 | 2 | 0 | 0 | 1b | 1 | NA | NA |  |  | X | X |
| 99 | M | 79 | LCNEC | 5.5 | 2 | 0 | 0 | 1b | 1 | NA | NA |  |  | X | X |
| 100 | M | 75 | LCNEC | 10 | 3 | 0 | 0 | 2a | 1 | NA | NA |  |  | X | X |
| 101 | M | 68 | LCNEC | 7 | 3 | 0 | 0 | 2b | 1 | NA | NA |  |  | X | X |
| 102 | M | 69 | LCNEC | 3.2 | 2 | 0 | 0 | 1b | 1 | NA | NA |  |  | X | X |
| 103 | F | 63 | LCNEC | 7.5 | 3 | 0 | 0 | 2b | 1 | NA | NA |  |  | X | X |
| 104 | M | 61 | LCNEC | 1.6 | 1 | 1 | 0 | 3a | 1 | NA | NA |  |  | X | X |
| 105 | M | 75 | LCNEC | 15 | 3 | 1 | 0 | 3a | 1 | NA | NA |  |  | X | X |
| 106 | M | 79 | LCNEC | 3.5 | 3 | 1 | 0 | 3a | 1 | NA | NA |  |  | X | X |
| 107 | F | 61 | LCNEC | 1.5 | 1 | 0 | 0 | 1a | 1 | NA | NA |  |  | X | X |
| 108 | M | 72 | LCNEC | 2.5 | 1 | 0 | 0 | 1a | 1 | NA | NA |  |  | X | X |
| 109 | M | 58 | LCNEC | 2.3 | 1 | 0 | 0 | 1a | 1 | NA | NA |  |  | X | X |
| 110 | M | 75 | LCNEC | 2.5 | 2 | 1 | 0 | 2a | 1 | NA | NA |  |  | X | X |
| 111 | F | 62 | LCNEC | 1.5 | 2 | 0 | 0 | 1b | 1 | NA | NA |  |  | X | X |
| 112 | M | 75 | LCNEC | 3.6 | 2 | 1 | 0 | 2a | 1 | NA | NA |  |  | X | X |
| 113 | M | 69 | LCNEC | 2.3 | 1 | 1 | 0 | 2a | 1 | NA | NA |  |  | X | X |
| 114 | M | 72 | LCNEC | 3.8 | 2 | 0 | 0 | 1b | 1 | NA | NA |  |  | X | X |
| 115 | M | 66 | LCNEC | 2.5 | 1 | 0 | 0 | 1a | 1 | NA | NA |  |  | X | X |
| 223 | M | 75 | LCNEC | 3.4 | 2 | 0 | 1 | 4 | 1 | 0 | 0 |  | X | X | X |
| 308 | M | 77 | LCNEC | 3.5 | 2 | 0 | 0 | 1b | 1 | 0 | 0 |  | X | X | X |
| 080 | M | 48 | SCLC | 4 | 3 | 1 | 0 | 3a | 1 | 1 | 1 | X | X | X | X |
| 348 | M | 72 | SCLC | 2.5 | 1 | 0 | 0 | 1a | 1 | 0 | 0 | X | X | X | X |
| 477 | F | 79 | SCLC | 2.8 | 1 | 1 | 0 | 3a | 1 | 1 | 0 | X | X | X | X |
| 205 | M | 66 | SCLC | 3.3 | 3 | 0 | 0 | 2b | 1 | NA | NA |  | X | X | X |
| 1 | M | 65 | SCLC | 3.5 | 2 | 1 | 0 | 2a | 1 | 1 | 0 |  |  | X | X |
| 3 | M | 53 | SCLC | 5.5 | 4 | 0 | 1 | 4 | 0 | 1 | 1 |  |  | X | X |
| 6 | M | 69 | SCLC | 2 | 1 | 1 | 0 | 3a | 1 | 1 | 0 |  |  | X | X |
| 7 | M | 60 | SCLC | 2.5 | 1 | 0 | 0 | 1a | 1 | NA | 0 |  |  | X | X |
| 9 | M | 67 | SCLC | 7.5 | 3 | 1 | 0 | 3a | 1 | NA | 0 |  |  | X | X |
| 12 | M | 68 | SCLC | 1.4 | 1 | 1 | 0 | 1 | 1 | 1 | 0 |  |  | X | X |
| 16 | F | 43 | SCLC | 5.5 | 2 | 1 | 0 | 2a | 1 | 1 | 0 |  |  | X | X |
| 20 | M | 81 | SCLC | 2.4 | 1 | 1 | 0 | 1a | 1 | NA | 1 |  |  | X | X |
| 43 | F | 64 | SCLC | 0.8 | 2 | 1 | 0 | 3a | 1 | NA | NA |  |  | X | X |
| 44 | F | 77 | SCLC | 1 | 2 | 1 | 0 | 3a | 1 | NA | NA |  |  | X | X |
| 45 | M | 65 | SCLC | NA | 2 | 1 | 0 | 3a | 1 | NA | NA |  |  | X | X |
| 46 | M | 64 | SCLC | NA | 4 | 1 | 0 | 3b | 1 | NA | NA |  |  | X | X |
| 47 | F | 55 | SCLC | NA | 1 | 1 | 0 | 3a | 1 | NA | NA |  |  | X | X |
| 48 | M | 63 | SCLC | NA | 4 | 1 | 0 | 3b | 1 | NA | NA |  |  | X | X |
| 49 | M | 71 | SCLC | NA | 1 | 1 | 1 | 4 | 1 | NA | NA |  |  | X | X |
| 50 | M | 52 | SCLC | NA | 4 | 1 | 1 | 4 | 1 | NA | NA |  |  | X | X |
| 51 | F | 66 | SCLC | NA | 2 | 0 | 1 | 4 | 1 | NA | NA |  |  | X | X |
| 52 | M | 62 | SCLC | NA | 2 | 1 | 0 | 3a | 1 | NA | NA |  |  | X | X |
| 53 | F | 73 | SCLC | NA | 4 | 1 | 0 | 3b | 1 | NA | NA |  |  | X | X |
| 54 | M | 79 | SCLC | NA | 4 | 1 | 0 | 3b | 1 | NA | NA |  |  | X | X |
| 55 | M | 71 | SCLC | NA | 2 | 1 | 0 | 3a | 1 | NA | NA |  |  | X | X |
| 56 | F | 70 | SCLC | NA | 4 | 1 | 1 | 4 | 1 | NA | NA |  |  | X | X |
| 57 | M | 84 | SCLC | NA | 4 | 1 | 1 | 4 | 1 | NA | NA |  |  | X | X |
| 58 | M | 71 | SCLC | NA | 4 | 1 | 1 | 4 | 1 | NA | NA |  |  | X | X |
| 60 | M | 63 | SCLC | NA | 4 | 1 | 1 | 4 | 1 | NA | NA |  |  | X | X |
| 61 | F | 80 | SCLC | NA | 4 | 1 | 0 | 3b | 1 | NA | NA |  |  | X | X |
| 62 | M | 71 | SCLC | NA | 4 | 1 | 1 | 4 | 1 | NA | NA |  |  | X | X |
| 63 | M | 72 | SCLC | NA | 2 | 1 | 1 | 4 | 1 | NA | NA |  |  | X | X |
| 64 | F | 63 | SCLC | NA | 2 | 1 | 1 | 4 | 1 | NA | NA |  |  | X | X |

**Note:** S, sex; A, age of diagnosis; His, histotype; Size, tumour size (cm); stage, tumour stage; Ncr, necrosis; V. inv, vascular invasion; P. inv, pleura invasion; WES, whole exome sequencing; HCTS, high-coverage target sequencing of 418 genes; CNA, copy number analysis; TS, targeted sequencing of 88 genes; TC, typical carcinoid; AC, atypical carcinoids; LCNEC, large cell neuroendocrine carcinoma; SCLC, small cell lung cancer.
