# Supplementary material for: Lung neuroendocrine tumours: deep sequencing of the four World Health Organization histotypes reveals chromatin‐remodelling genes as major players and a prognostic role for TERT, RB1, MEN1 and KMT2D
Source: J Pathol. 2016 Dec 29;241(4):488–500. doi: 10.1002/path.4853 (PMC5324596; doi:10.1002/path.4853)
Supplement: Supplementary file 8 — Table S3. Prevalence of mutations in 88 genes for 148 lung neuroendocrine tumours. Related to Figure S2A and Table 2. [file PATH-241-488-s001.docx]

**Supplementary Table S3.** Prevalence of mutations in 88 genes for 148 lung neuroendocrine tumours. Related to Figure 1A and Table 2.

| **Mutation distribution among histological subtypes** | | | | | | | | | | | **Type of mutation** | | | | | **p-value*** |
| --- | --- | --- | --- | --- | --- | --- | --- | --- | --- | --- | --- | --- | --- | --- | --- | --- |
|  | TC | (n=53) | AC | (n=35) | LCNEC | (n=27) | SCLC | (n=33) | Total | (n=148) | M | N | D | S | F |  |
| **Genes^#^** | n | [%] | n | [%] | n | [%] | n | [%] | n | [%] |  |  |  |  |  |  |
| *APC* | 1 | [1.9] | 1 | [2.9] |  |  | 1 | [3.0] | 3 | [2.0] | 3 |  |  |  |  | - |
| *ARID1A* | 3 | [5.7] | 2 | [5.7] |  |  | 1 | [3.0] | 6 | [4.1] | 3 | 3 |  |  |  | - |
| *ARID1B* |  |  | 1 | [2.9] | 1 | [3.7] | 1 | [3.0] | 3 | [2.0] | 3 |  |  |  |  | - |
| *ARID2* |  |  | 2 | [5.7] | 1 | [3.7] | 2 | [6.1] | 5 | [3.4] | 5 |  |  |  |  | - |
| *ATM* |  |  |  |  | 1 | [3.7] | 1 | [3.0] | 2 | [1.4] | 2 |  |  |  |  | - |
| *ATRX* |  |  | 1 | [2.9] |  |  | 1 | [3.0] | 2 | [1.4] | 2 |  |  |  |  | - |
| *BAP1* | 1 | [1.9] |  |  |  |  |  |  | 1 | [0.7] | 1 |  |  |  |  | - |
| *CSF1R* |  |  |  |  |  |  | 1 | [3.0] | 1 | [0.7] | 1 |  |  |  |  | - |
| *CSMD3* | 2 | [3.8] |  |  | 7 | [25.9] | 8 | [24.2] | 16 | [10.8] | 15 | 1 |  |  |  | **0.0011** |
| *CTNNB1* | 1 | [1.9] |  |  |  |  | 1 | [3.0] | 2 | [1.4] | 2 |  |  |  |  | - |
| *DAXX* |  |  |  |  | 1 | [3.7] |  |  | 1 | [0.7] | 1 |  |  |  |  | - |
| *DSCAML1* | 2 | [3.8] | 2 | [5.7] | 1 | [3.7] | 2 | [6.1] | 7 | [4.7] | 5 |  |  | 2 |  | - |
| *EGFR* |  |  |  |  |  |  | 2 | [6.1] | 2 | [1.4] | 2 |  |  |  |  | - |
| *ERBB4* | 1 | [1.9] |  |  | 2 | [7.5] |  |  | 3 | [2.0] | 3 |  |  |  |  | - |
| *FGFR2* |  |  |  |  |  |  | 1 | [3.0] | 1 | [0.7] | 1 |  |  |  |  | - |
| *FGFR3* |  |  |  |  |  |  | 1 | [3.0] | 1 | [0.7] | 1 |  |  |  |  | - |
| *FLT3* | 1 | [1.9] |  |  |  |  |  |  | 1 | [0.7] | 1 |  |  |  |  | - |
| *GNAS* | 1 | [1.9] |  |  |  |  |  |  | 1 | [0.7] | 1 |  |  |  |  | - |
| *HRAS* |  |  |  |  |  |  | 1 | [3.0] | 1 | [0.7] | 1 |  |  |  |  | - |
| *IDH1* |  |  | 1 | [2.9] |  |  |  |  | 1 | [0.7] | 1 |  |  |  |  | - |
| *JAK2* | 1 | [1.9] |  |  |  |  |  |  | 1 | [0.7] |  | 1 |  |  |  | - |
| *KAT6A* |  |  |  |  |  |  | 1 | [3.0] | 1 | [0.7] |  | 1 |  |  |  | - |
| *KAT6B* | 1 | [1.9] |  |  | 1 | [3.7] |  |  | 2 | [1.4] | 2 |  |  |  |  | - |
| *KDM5C* |  |  | 1 | [2.9] |  |  |  |  | 1 | [0.7] | 1 |  |  |  |  | - |
| *KDR* |  |  | 1 | [2.9] |  |  | 3 | [9.1] | 4 | [2.7] | 3 |  |  | 1 |  | - |
| *KIT* |  |  |  |  |  |  | 1 | [3.0] | 1 | [0.7] | 1 |  |  |  |  | - |
| *KMT2A* |  |  | 1 | [2.9] | 1 | [3.7] |  |  | 2 | [1.4] | 2 |  |  |  |  | - |
| *KMT2C* | 3 | [5.7] | 4 | [11.4] | 2 | [7.5] |  |  | 9 | [6.1] | 6 | 3 |  |  |  | 0.27 |
| *KMT2D* | 1 | [1.9] | 3 | [8.6] | 5 | [18.5] | 8 | [24.2] | 17 | [11.5] | 12 | 4 | 1 |  |  | **0.0094** |
| *KRAS* | 2 | [3.8] | 1 | [2.9] | 2 | [7.5] |  |  | 5 | [3.4] | 5 |  |  |  |  | - |
| *LRP1B* | 2 | [3.8] | 1 | [2.9] | 5 | [18.5] | 11 | [33.3] | 19 | [12.8] | 14 | 3 |  | 2 |  | **0.00088** |
| *MEN1* | 3 | [5.7] | 7 | [20.0] | 1 | [3.7] |  |  | 11 | [7.4] | 6 | 3 | 1 |  | 1 | **0.022** |
| *MET* |  |  |  |  | 1 | [3.7] |  |  | 1 | [0.7] | 1 |  |  |  |  | - |
| *NCAM2* | 2 | [3.8] | 1 | [2.9] | 2 | [7.5] | 2 | [6.1] | 7 | [4.7] | 7 |  |  |  |  | - |
| *NOTCH2* | 2 | [3.8] | 2 | [5.7] | 4 | [14.8] | 1 | [3.0] | 9 | [6.1] | 7 |  |  | 2 |  | 0.28 |
| *PBRM1* | 1 | [1.9] |  |  | 1 | [3.7] |  |  | 2 | [1.4] | 2 |  |  |  |  | - |
| *PCLO* | 1 | [1.9] | 3 | [8.6] | 3 | [11.1] | 3 | [9.1] | 10 | [6.8] | 9 |  | 1 |  |  | 0.28 |
| *PDGFRA* |  |  | 2 | [5.7] |  |  |  |  | 2 | [1.4] | 2 |  |  |  |  | - |
| *PIK3CA* |  |  | 1 | [2.9] | 3 | [11.1] | 1 | [3.0] | 5 | [3.4] | 5 |  |  |  |  | 0.073 |
| *PTEN* | 1 | [1.9] |  |  |  |  | 1 | [3.0] | 2 | [1.4] |  | 2 |  |  |  | - |
| *PTPN11* |  |  |  |  |  |  | 1 | [3.0] | 1 | [0.7] | 1 |  |  |  |  | - |
| *PTPRZ1* |  |  | 3 | [8.6] | 1 | [3.7] | 2 | [6.1] | 6 | [4.1] | 6 |  |  |  |  | - |
| *RAI1* |  |  | 1 | [2.9] |  |  | 1 | [3.0] | 2 | [1.4] | 1 |  |  |  | 1 | - |
| *RB1* | 1 | [1.9] | 1 | [2.9] | 4 | [14.8] | 8 | [24.2] | 14 | [9.5] | 8 | 4 |  | 1 | 1 | **0.0047** |
| *RIN3* |  |  |  |  | 1 | [3.7] | 2 | [6.1] | 3 | [2.0] | 3 |  |  |  |  | - |
| *SETD2* | 1 | [1.9] | 2 | [5.7] | 1 | [3.7] |  |  | 4 | [2.7] | 4 |  |  |  |  | - |
| *SMARCA2* |  |  |  |  | 3 | [11.1] |  |  | 3 | [2.0] | 1 | 1 | 1 |  |  | **0.011** |
| *SMARCA3* |  |  |  |  |  |  | 1 | [3.0] | 1 | [0.7] | 1 |  |  |  |  | - |
| *SMARCA4* |  |  | 3 | [8.6] | 1 | [3.7] |  |  | 4 | [2.7] | 2 | 2 |  |  |  | - |
| *SPHKAP* | 1 | [1.9] | 2 | [5.7] | 2 | [7.5] | 1 | [3.0] | 6 | [4.1] | 6 |  |  |  |  | - |
| *STK11* |  |  |  |  | 1 | [3.7] |  |  | 1 | [0.7] | 1 |  |  |  |  | - |
| *TDRD7* | 1 | [1.9] | 1 | [2.9] |  |  |  |  | 2 | [1.4] | 2 |  |  |  |  | - |
| *THSD7B* |  |  |  |  | 2 | [7.5] | 3 | [9.1] | 5 | [3.4] | 5 |  |  |  |  | - |
| *TP53* | 5 | [9.4] | 4 | [11.4] | 18 | [66.7] | 21 | [63.6] | 48 | [32.4] | 40 | 5 |  | 1 | 2 | **5.4E-10** |
| *TSC1* |  |  |  |  |  |  | 1 | [3.0] | 1 | [0.7] | 1 |  |  |  |  | - |
| *TSC2* |  |  |  |  | 1 | [3.7] |  |  | 1 | [0.7] | 1 |  |  |  |  | - |

**Note:** TC, typical carcinoid; AC, atypical carcinoid; LCNEC, large-cell neuroendocrine carcinoma; SCLC, small-cell lung carcinoma; M, missense mutation; N, nonsense mutation; D, deletion; S, splice site alteration; F, frameshift mutation.

^#^ 88 genes were analysed; 56 of them were found mutated in at least one case and are here reported.

* Fisher’s exact test with correction for multiple comparisons according to Benjamini-Hochberg was performed if a gene was mutated in at least 10% of cases of one LNET subtype.
