# Supplementary material for: Lung neuroendocrine tumours: deep sequencing of the four World Health Organization histotypes reveals chromatin‐remodelling genes as major players and a prognostic role for TERT, RB1, MEN1 and KMT2D
Source: J Pathol. 2016 Dec 29;241(4):488–500. doi: 10.1002/path.4853 (PMC5324596; doi:10.1002/path.4853)
Supplement: Supplementary file 9 — Table S4A. Discovery screen, whole exome sequencing: list of mutations found in 20 lung neuroendocrine tumours. Related to Supplementary Figure S1A. Table S4B. Discovery screen, high coverage targeted sequencing of 418 genes: list of mutations found in 46 lung neuroendocrine tumours. Related to Supplementary Figure S1B. Table S4C. Discovery screen, integration of whole exome sequencing and high coverage targeted sequencing of 418 genes: list of 36 genes mutated in at least two cases of 46 lung neuroendocrine tumours. Related to Supplementary Figures S1A and S1B. [file PATH-241-488-s010.zip › PATH_4853_TableS4C.docx]

**Supplementary Table S4C.** Discovery screen, integration of whole exome sequencing and high coverage targeted sequencing of 418 genes: list of 36 genes mutated at least two cases of 46 lung neuroendocrine tumours. Related to Supplementary Figures S2A and S2B.

| **Genes** | **Mutation distribution among histological subtypes** | | | | | | |  |  |  | **Type of mutation** | | | | |
| --- | --- | --- | --- | --- | --- | --- | --- | --- | --- | --- | --- | --- | --- | --- | --- |
|  | TC | (n=23) | AC | (n=14) | LCNEC | (n=5) | SCLC | (n=4) | Total | (n=46) | M | N | D | S | F |
|  | n | [%] | n | [%] | n | [%] | n | [%] | n | [%] |  |  |  |  |  |
| *APC* | 1 | [4.3] | 1 | [7.1] | 0 | [0.0] | 0 | [0.0] | 2 | [4.3] | 2 |  |  |  |  |
| *ARID1A* | 1 | [4.3] | 0 | [0.0] | 0 | [0.0] | 0 | [0.0] | 1 | [2.2] |  | 1 |  |  |  |
| *ARID2* | 0 | [0.0] | 1 | [7.1] | 1 | [20.0] | 1 | [25.0] | 3 | [6.5] | 3 |  |  |  |  |
| *ATRX* | 0 | [0.0] | 1 | [7.1] | 0 | [0.0] | 0 | [0.0] | 1 | [2.2] | 1 |  |  |  |  |
| *BAP1* | 1 | [4.3] | 0 | [0.0] | 0 | [0.0] | 0 | [0.0] | 1 | [2.2] | 1 |  |  |  |  |
| *CSMD3* | 1 | [4.3] | 0 | [0.0] | 0 | [0.0] | 3 | [75.0] | 4 | [8.7] | 4 |  |  |  |  |
| *DAXX* | 0 | [0.0] | 0 | [0.0] | 1 | [20.0] | 0 | [0.0] | 1 | [2.2] | 1 |  |  |  |  |
| *DSCAML1* | 0 | [0.0] | 1 | [7.1] | 0 | [0.0] | 0 | [0.0] | 1 | [2.2] | 1 |  |  |  |  |
| *ERBB4* | 0 | [0.0] | 0 | [0.0] | 1 | [20.0] | 0 | [0.0] | 1 | [2.2] | 1 |  |  |  |  |
| *FLT3* | 1 | [4.3] | 0 | [0.0] | 0 | [0.0] | 0 | [0.0] | 1 | [2.2] | 1 |  |  |  |  |
| *JAK2* | 1 | [4.3] | 0 | [0.0] | 0 | [0.0] | 0 | [0.0] | 1 | [2.2] |  | 1 |  |  |  |
| *KAT6A* | 0 | [0.0] | 0 | [0.0] | 0 | [0.0] | 1 | [25.0] | 1 | [2.2] |  | 1 |  |  |  |
| *KAT6B* | 1 | [4.3] | 0 | [0.0] | 1 | [20.0] | 0 | [0.0] | 2 | [4.3] | 2 |  |  |  |  |
| *KDM5C* | 0 | [0.0] | 1 | [7.1] | 0 | [0.0] | 0 | [0.0] | 1 | [2.2] | 1 |  |  |  |  |
| *KDR* | 0 | [0.0] | 1 | [7.1] | 0 | [0.0] | 1 | [25.0] | 2 | [4.3] | 2 |  |  |  |  |
| *KMT2A* | 0 | [0.0] | 1 | [7.1] | 1 | [20.0] | 0 | [0.0] | 2 | [4.3] | 2 |  |  |  |  |
| *KMT2C* | 1 | [4.3] | 1 | [7.1] | 1 | [20.0] | 0 | [0.0] | 3 | [6.5] | 3 |  |  |  |  |
| *KMT2D* | 0 | [0.0] | 0 | [0.0] | 2 | [40.0] | 2 | [50.0] | 4 | [8.7] | 3 |  | 1 |  |  |
| *LRP1B* | 2 | [8.7] | 0 | [0.0] | 2 | [40.0] | 2 | [50.0] | 6 | [13.0] | 5 |  |  | 1 |  |
| *MEN1* | 0 | [0.0] | 1 | [7.1] | 1 | [20.0] | 0 | [0.0] | 2 | [4.3] | 1 | 1 |  |  |  |
| *MET* | 0 | [0.0] | 0 | [0.0] | 1 | [20.0] | 0 | [0.0] | 1 | [2.2] | 1 |  |  |  |  |
| *NCAM2* | 0 | [0.0] | 1 | [7.1] | 0 | [0.0] | 1 | [25.0] | 2 | [4.3] | 2 |  |  |  |  |
| *NOTCH2* | 1 | [4.3] | 2 | [14.3] | 2 | [40.0] | 0 | [0.0] | 5 | [10.9] | 5 |  |  |  |  |
| *PCLO* | 1 | [4.3] | 1 | [7.1] | 0 | [0.0] | 1 | [25.0] | 3 | [6.5] | 3 |  |  |  |  |
| *PDGFRA* | 0 | [0.0] | 2 | [14.3] | 0 | [0.0] | 0 | [0.0] | 2 | [4.3] | 2 |  |  |  |  |
| *PIK3CA* | 0 | [0.0] | 0 | [0.0] | 1 | [20.0] | 1 | [25.0] | 2 | [4.3] | 2 |  |  |  |  |
| *PTPRZ1* | 0 | [0.0] | 1 | [7.1] | 1 | [20.0] | 2 | [50.0] | 4 | [8.7] | 4 |  |  |  |  |
| *RAI1* | 0 | [0.0] | 1 | [7.1] | 0 | [0.0] | 0 | [0.0] | 1 | [2.2] |  |  |  |  | 1 |
| *RB1* | 0 | [0.0] | 1 | [7.1] | 1 | [20.0] | 2 | [50.0] | 4 | [8.7] | 1 | 1 |  | 1 | 1 |
| *RIN3* | 0 | [0.0] | 0 | [0.0] | 1 | [20.0] | 1 | [25.0] | 2 | [4.3] | 2 |  |  |  |  |
| *SETD2* | 0 | [0.0] | 1 | [7.1] | 0 | [0.0] | 0 | [0.0] | 1 | [2.2] | 1 |  |  |  |  |
| *SMARCA4* | 0 | [0.0] | 2 | [14.3] | 1 | [20.0] | 0 | [0.0] | 3 | [6.5] | 1 | 2 |  |  |  |
| *SPHKAP* | 1 | [4.3] | 2 | [14.3] | 0 | [0.0] | 0 | [0.0] | 3 | [6.5] | 3 |  |  |  |  |
| *TDRD7* | 1 | [4.3] | 1 | [7.1] | 0 | [0.0] | 0 | [0.0] | 2 | [4.3] | 2 |  |  |  |  |
| *THSD7B* | 0 | [0.0] | 0 | [0.0] | 1 | [20.0] | 1 | [25.0] | 2 | [4.3] | 2 |  |  |  |  |
| *TP53* | 1 | [4.3] | 1 | [7.1] | 3 | [60.0] | 4 | [100.0] | 9 | [19.6] | 8 | 1 |  |  |  |

**Note:** TC, typical carcinoid; AC, atypical carcinoid; LCNEC, large-cell neuroendocrine carcinoma; SCLC, small-cell lung carcinoma; M, missense mutation; N, nonsense mutation; D, deletion; S, splice site alteration; F, frameshift mutation.
