# Supplementary material for: Lung neuroendocrine tumours: deep sequencing of the four World Health Organization histotypes reveals chromatin‐remodelling genes as major players and a prognostic role for TERT, RB1, MEN1 and KMT2D
Source: J Pathol. 2016 Dec 29;241(4):488–500. doi: 10.1002/path.4853 (PMC5324596; doi:10.1002/path.4853)
Supplement: Supplementary file 9 — Table S4A. Discovery screen, whole exome sequencing: list of mutations found in 20 lung neuroendocrine tumours. Related to Supplementary Figure S1A. Table S4B. Discovery screen, high coverage targeted sequencing of 418 genes: list of mutations found in 46 lung neuroendocrine tumours. Related to Supplementary Figure S1B. Table S4C. Discovery screen, integration of whole exome sequencing and high coverage targeted sequencing of 418 genes: list of 36 genes mutated in at least two cases of 46 lung neuroendocrine tumours. Related to Supplementary Figures S1A and S1B. [file PATH-241-488-s010.zip › PATH_4853_TableS4A.docx]

**Supplementary Table S4A.** Discovery screen, whole exome sequencing: list of mutations found in 20 lung neuroendocrine tumours. Related to Supplementary Figure S2A.

| **ID** | **Histotype** | **Gene** | **Chr** | **Pos** | **Ref** | **Alt** | **Var.Freq** | **Effect** | **effect type** | **change type** | **validated** |
| --- | --- | --- | --- | --- | --- | --- | --- | --- | --- | --- | --- |
| 209 | TC | ABCD1 | X | 152990505 | C | T | 21 | ; | silent | SNV | no |
|  | TC | B3GAT1 | 11 | 134254024 | G | T | 13 | C/*;57 | nonsense | SNV | no |
|  | TC | BASP1 | 5 | 17275475 | G | T | 11 | E/D;50 | missense | SNV | no |
|  | TC | CSMD1 | 8 | 3019683 | G | T | 12 | Q/K;1948 | missense | SNV | no |
|  | TC | DIAPH2 | X | 95993734 | A | T | 14 | E/D;105 | missense | SNV | no |
|  | TC | DSCAML1 | 11 | 117651410 | G | T | 10 | S/R;114 | missense | SNV | yes |
|  | TC | GPR113 | 2 | 26538467 | G | T | 11 | P/Q;282 | missense | SNV | no |
|  | TC | HIC2 | 22 | 21799521 | G | A | 31 | E/K;113 | missense | SNV | no |
|  | TC | MIB2 | 1 | 1563873 | G | T | 12 | R/L;773 | missense | SNV | no |
|  | TC | MTHFD2L | 4 | 75067042 | A | G | 24 | I/V;223 | missense | SNV | no |
|  | TC | NCKAP5L | 12 | 50191040 | G | T | 11 | D/E;201 | missense | SNV | no |
|  | TC | RAPGEF2 | 4 | 160253650 | G | T | 13 | A/S;485 | missense | SNV | no |
|  | TC | S100A16 | 1 | 153580157 | G | T | 10 | N/K;55 | missense | SNV | no |
| 243 | TC | CDH11 | 16 | 65038691 | G | A | 12 | R/W;28 | missense | SNV | yes |
|  | TC | DSCAM | 21 | 41452216 | G | A | 24 | P/L;1428 | missense | SNV | no |
|  | TC | ERBB3 | 12 | 56495126 | G | T | 10 | M/I;1161 | missense | SNV | yes |
|  | TC | NFIB | 9 | 14307431 | T | A | 13 | K/M;40 | missense | SNV | no |
|  | TC | UGT2A3 | 4 | 69796366 | A | G | 14 | I/T;401 | missense | SNV | no |
|  | TC | VAX1 | 10 | 118897620 | A | G | 12 | ; | silent | SNV | no |
| 245 | TC | AKAP4 | X | 49958854 | G | T | 12 | N/K;170 | missense | SNV | no |
|  | TC | C11orf54 | 11 | 93494685 | T | G | 10 | F/C;210 | missense | SNV | no |
|  | TC | C11orf82 | 11 | 82643371 | G | A | 12 | E/K;331 | missense | SNV | no |
|  | TC | FOLH1 | 11 | 49192762 | * | -AGAACCA | 36 | FRAME_SHIFT;429 | frameshift | DEL | no |
|  | TC | ISOC1 | 5 | 128440899 | G | A | 46 | G/R;151 | missense | SNV | no |
|  | TC | LIPA | 10 | 90988130 | T | G | 56 | Q/H;85 | missense | SNV | no |
|  | TC | TTN | 2 | 179563630 | C | G | 47 | A/P;9915 | missense | SNV | no |
|  | TC | NAV1 | 1 | 201687726 | A | C | 54 | T/P;357 | missense | SNV | no |
| 304 | TC | ABCB1 | 7 | 87135326 | G | C | 18 | Q/E;1175 | missense | SNV | no |
|  | TC | ABCB11 | 2 | 169826634 | G | T | 15 | P/H;577 | missense | SNV | no |
|  | TC | ABHD12 | 20 | 25288678 | G | T | 11 | T/K;264 | missense | SNV | no |
|  | TC | ABHD12 | 20 | 25295585 | G | A | 24 | H/Y;199 | missense | SNV | no |
|  | TC | ACOT12 | 5 | 80643674 | G | T | 44 | P/Q;191 | missense | SNV | no |
|  | TC | ACTC1 | 15 | 35085490 | G | T | 24 | A/D;137 | missense | SNV | no |
|  | TC | ACTN2 | 1 | 236923040 | T | C | 44 | M/T;773 | missense | SNV | no |
|  | TC | ADAMTS16 | 5 | 5239380 | C | A | 32 | H/Q;757 | missense | SNV | yes |
|  | TC | ADAMTSL1 | 9 | 18574090 | G | T | 42 | K/N;100 | missense | SNV | no |
|  | TC | ADAMTSL4 | 1 | 150529198 | C | A | 23 | P/T;560 | missense | SNV | no |
|  | TC | ADGB | 6 | 147042606 | G | T | 42 | C/F;687 | missense | SNV | no |
|  | TC | AK5 | 1 | 77806158 | G | A | 52 | D/N;266 | missense | SNV | no |
|  | TC | AKAP17A | X | 1719897 | C | G | 100 | P/A;500 | missense | SNV | no |
|  | TC | AKAP6 | 14 | 33291713 | G | A | 45 | S/N;1565 | missense | SNV | no |
|  | TC | AKAP9 | 7 | 91652179 | * | +AAC | 43 | CODON_INSERTION;1335 | frameshift | INS | yes |
|  | TC | ALDOB | 9 | 104189771 | C | A | 11 | C/F;178 | missense | SNV | no |
|  | TC | ALOX5 | 10 | 45935899 | G | A | 26 | E/K;335 | missense | SNV | no |
|  | TC | ANXA6 | 5 | 150501734 | C | T | 63 | A/T;441 | missense | SNV | no |
|  | TC | APBB1 | 11 | 6423414 | T | A | 34 | E/V;427 | missense | SNV | no |
|  | TC | API5 | 11 | 43357529 | G | T | 31 | G/C;493 | missense | SNV | no |
|  | TC | AQP6 | 12 | 50367159 | T | C | 27 | M/T;68 | missense | SNV | no |
|  | TC | ARHGEF18 | 19 | 7532238 | C | T | 63 | R/W;862 | missense | SNV | no |
|  | TC | ARHGEF40 | 14 | 21542654 | C | A | 21 | S/R;255 | missense | SNV | no |
|  | TC | ARHGEF40 | 14 | 21543067 | G | A | 15 | R/Q;393 | missense | SNV | no |
|  | TC | ARID3B | 15 | 74836290 | * | +CAG | 50 | CODON_INSERTION;5 | frameshift | INS | no |
|  | TC | ARRDC4 | 15 | 98512353 | G | C | 25 | G/A;209 | missense | SNV | no |
|  | TC | ASMTL | X | 1522164 | * | -A | 95 | FRAME_SHIFT;622 | frameshift | DEL | no |
|  | TC | ATF1 | 12 | 51189708 | A | G | 54 | E/G;37 | missense | SNV | no |
|  | TC | ATG12 | 5 | 115173333 | C | T | 63 | E/K;98 | missense | SNV | no |
|  | TC | ATP11A | 13 | 113510262 | G | A | 38 | D/N;761 | missense | SNV | no |
|  | TC | ATP2C2 | 16 | 84492919 | A | G | 54 | N/D;754 | missense | SNV | no |
|  | TC | ATP6V1C2 | 2 | 10917761 | C | A | 12 | H/Q;292 | missense | SNV | no |
|  | TC | BAI1 | 8 | 143623362 | C | T | 19 | P/L;1256 | missense | SNV | no |
|  | TC | BAI2 | 1 | 32201189 | C | A | 27 | Q/H;1115 | missense | SNV | no |
|  | TC | BAP1 | 3 | 52437651 | T | G | 86 | N/H;504 | missense | SNV | yes |
|  | TC | BAP1 | 3 | 52437866 | G | A | 81 | S/F;432 | missense | SNV | yes |
|  | TC | BCL6B | 17 | 6927445 | G | T | 45 | V/L;75 | missense | SNV | no |
|  | TC | BCL9L | 11 | 118772641 | G | A | 35 | P/L;604 | missense | SNV | no |
|  | TC | BEST3 | 12 | 70066670 | A | C | 35 | L/V;315 | missense | SNV | no |
|  | TC | BLK | 8 | 11406540 | G | T | 10 | D/Y;93 | missense | SNV | yes |
|  | TC | BLZF1 | 1 | 169349818 | G | C | 38 | Q/H;256 | missense | SNV | no |
|  | TC | BOD1L1 | 4 | 13606251 | T | C | 42 | E/G;758 | missense | SNV | no |
|  | TC | C10orf118 | 10 | 115934144 | G | T | 62 | ; | silent | SNV | no |
|  | TC | C1orf129 | 1 | 171033377 | T | C | 29 | F/L;828 | missense | SNV | no |
|  | TC | C22orf13 | 22 | 24942883 | A | T | 67 | C/S;129 | missense | SNV | no |
|  | TC | C2orf16 | 2 | 27802024 | G | T | 53 | G/V;862 | missense | SNV | no |
|  | TC | C5 | 9 | 123742397 | T | C | 59 | I/V;1208 | missense | SNV | no |
|  | TC | C6orf72 | 6 | 149893466 | A | G | 52 | I/V;56 | missense | SNV | no |
|  | TC | CAMSAP2 | 1 | 200709089 | G | A | 81 | G/E;45 | missense | SNV | no |
|  | TC | CAPN12 | 19 | 39224811 | G | A | 43 | Q/*;617 | nonsense | SNV | no |
|  | TC | CAPN5 | 11 | 76833089 | C | A | 36 | P/T;533 | missense | SNV | no |
|  | TC | CASP8AP2 | 6 | 90578267 | * | -ATC | 45 | CODON_DELETION;1753 | frameshift | DEL | no |
|  | TC | CD1C | 1 | 158261973 | G | T | 25 | S/I;143 | missense | SNV | no |
|  | TC | CD59 | 11 | 33731679 | T | A | 32 | H/L;127 | missense | SNV | no |
|  | TC | CDC40 | 6 | 110522764 | G | T | 11 | G/*;94 | nonsense | SNV | no |
|  | TC | CDH12 | 5 | 22078566 | A | T | 23 | Y/N;74 | missense | SNV | no |
|  | TC | CDH17 | 8 | 95178017 | G | T | 21 | Y/*;418 | nonsense | SNV | no |
|  | TC | CDHR4 | 3 | 49828279 | G | A | 30 | R/W;785 | missense | SNV | no |
|  | TC | CEACAM18 | 19 | 51986457 | C | T | 28 | A/V;348 | missense | SNV | no |
|  | TC | CELSR3 | 3 | 48698353 | C | T | 74 | R/Q;572 | missense | SNV | no |
|  | TC | CEP68 | 2 | 65301483 | G | A | 27 | G/E;651 | missense | SNV | no |
|  | TC | CEP97 | 3 | 101451414 | C | T | 28 | P/L;215 | missense | SNV | no |
|  | TC | CHD1L | 1 | 146757075 | * | -A | 46 | FRAME_SHIFT;643 | frameshift | DEL | no |
|  | TC | CHIT1 | 1 | 203194186 | C | T | 17 | G/S;102 | missense | SNV | no |
|  | TC | CHL1 | 3 | 369938 | A | T | 52 | N/Y;96 | missense | SNV | no |
|  | TC | CMYA5 | 5 | 79039688 | G | T | 58 | K/N;3559 | missense | SNV | no |
|  | TC | CNTFR | 9 | 34556276 | G | T | 28 | L/I;249 | missense | SNV | no |
|  | TC | COX8A | 11 | 63743765 | C | A | 11 | H/Q;61 | missense | SNV | no |
|  | TC | CPB1 | 3 | 148563303 | C | A | 29 | R/S;291 | missense | SNV | no |
|  | TC | CREB3L3 | 19 | 4157205 | C | A | 12 | L/I;124 | missense | SNV | no |
|  | TC | CREBBP | 16 | 3781226 | G | T | 13 | H/Q;1713 | missense | SNV | yes |
|  | TC | CTNND2 | 5 | 11364866 | G | T | 13 | S/R;438 | missense | SNV | no |
|  | TC | CUL5 | 11 | 107974965 | C | T | 28 | Q/*;733 | nonsense | SNV | no |
|  | TC | CYP3A7 | 7 | 99332727 | C | T | 23 | ; | silent | SNV | no |
|  | TC | CYP3A7-CYP3AP1 | 7 | 99332727 | C | T | 23 | ; | silent | SNV | no |
|  | TC | DAPK2 | 15 | 64332456 | A | C | 34 | F/C;2 | missense | SNV | no |
|  | TC | KAT6B | 10 | 76603142 | A | T | 50 | Y/F;176 | missense | SNV | yes |
|  | TC | DDX17 | 22 | 38890952 | G | C | 19 | A/G;327 | missense | SNV | no |
|  | TC | DEM1 | 1 | 40981246 | * | +G | 48 | FRAME_SHIFT;344 | frameshift | INS | no |
|  | TC | DIAPH3 | 13 | 60545105 | G | A | 70 | P/S;614 | missense | SNV | no |
|  | TC | DLG1 | 3 | 196863495 | T | C | 54 | N/S;346 | missense | SNV | no |
|  | TC | DPY19L4 | 8 | 95746908 | G | T | 34 | V/F;60 | missense | SNV | no |
|  | TC | DPYSL4 | 10 | 134012407 | G | A | 37 | C/Y;248 | missense | SNV | no |
|  | TC | DSG2 | 18 | 29115288 | G | T | 12 | E/*;446 | nonsense | SNV | no |
|  | TC | DUPD1 | 10 | 76797615 | C | A | 10 | E/D;214 | missense | SNV | no |
|  | TC | EFEMP2 | 11 | 65638649 | C | T | 60 | D/N;116 | missense | SNV | no |
|  | TC | EFR3A | 8 | 132997218 | C | T | 42 | R/C;594 | missense | SNV | no |
|  | TC | ENTHD1 | 22 | 40283459 | A | T | 13 | C/*;98 | nonsense | SNV | no |
|  | TC | EPPK1 | 8 | 144941495 | G | A | 60 | T/M;1976 | missense | SNV | no |
|  | TC | EPS8L3 | 1 | 110301047 | G | A | 53 | P/L;204 | missense | SNV | no |
|  | TC | ERI2 | 16 | 20809249 | C | T | 57 | G/R;625 | missense | SNV | no |
|  | TC | FAM53A | 4 | 1643177 | * | -G | 57 | FRAME_SHIFT;347 | frameshift | DEL | no |
|  | TC | FBXO10 | 9 | 37525169 | G | T | 34 | S/R;569 | missense | SNV | no |
|  | TC | FER1L6 | 8 | 125083796 | G | C | 12 | S/T;1339 | missense | SNV | no |
|  | TC | FN3KRP | 17 | 80678187 | C | G | 23 | H/Q;111 | missense | SNV | no |
|  | TC | FRMPD4 | X | 12734871 | A | C | 63 | M/L;765 | missense | SNV | no |
|  | TC | FSD2 | 15 | 83455835 | T | C | 53 | Y/C;103 | missense | SNV | no |
|  | TC | FTO | 16 | 53968017 | G | C | 44 | A/P;454 | missense | SNV | no |
|  | TC | FYB | 5 | 39202332 | T | C | 22 | D/G;254 | missense | SNV | no |
|  | TC | GAB4 | 22 | 17473034 | C | A | 51 | R/S;69 | missense | SNV | no |
|  | TC | GLB1L2 | 11 | 134217293 | T | G | 36 | F/C;175 | missense | SNV | no |
|  | TC | GOLGB1 | 3 | 121415391 | C | G | 33 | E/Q;1327 | missense | SNV | no |
|  | TC | GPR1 | 2 | 207041559 | T | C | 35 | H/R;138 | missense | SNV | no |
|  | TC | GPR144 | 9 | 127232777 | A | G | 14 | T/A;855 | missense | SNV | no |
|  | TC | GPR146 | 7 | 1097574 | * | +G | 44 | FRAME_SHIFT;141 | frameshift | INS | no |
|  | TC | GPRC6A | 6 | 117113762 | * | +GG | 33 | FRAME_SHIFT;775 | frameshift | INS | no |
|  | TC | GPRC6A | 6 | 117113763 | * | +A | 32 | FRAME_SHIFT;775 | frameshift | INS | no |
|  | TC | HDAC9 | 7 | 18767272 | G | C | 27 | D/H;601 | missense | SNV | no |
|  | TC | HIPK4 | 19 | 40885564 | C | T | 12 | R/H;594 | missense | SNV | no |
|  | TC | HIVEP1 | 6 | 12124081 | G | C | 47 | L/F;1351 | missense | SNV | no |
|  | TC | HPSE | 4 | 84240539 | G | A | 26 | R/*;153 | nonsense | SNV | no |
|  | TC | HSP90AA1 | 14 | 102551747 | G | T | 41 | T/K;306 | missense | SNV | no |
|  | TC | IGFALS | 16 | 1841643 | G | A | 88 | P/L;297 | missense | SNV | no |
|  | TC | IL3RA | X | 1497644 | G | C | 75 | V/L;323 | missense | SNV | no |
|  | TC | IL7R | 5 | 35861068 | T | C | 100 | I/T;66 | missense | SNV | no |
|  | TC | IL7R | 5 | 35871190 | G | A | 100 | V/I;138 | missense | SNV | no |
|  | TC | IRF4 | 6 | 397138 | G | C | 16 | D/H;175 | missense | SNV | no |
|  | TC | IRS1 | 2 | 227661163 | G | C | 32 | Y/*;764 | nonsense | SNV | yes |
|  | TC | ISX | 22 | 35463012 | G | T | 39 | ; | silent | SNV | no |
|  | TC | KAL1 | X | 8565226 | C | A | 10 | K/N;130 | missense | SNV | no |
|  | TC | LRP1B | 2 | 141751640 | G | T | 29 | H/Q;856 | missense | SNV | yes |
|  | TC | KIAA1614 | 1 | 180886998 | G | T | 46 | G/W;337 | missense | SNV | no |
|  | TC | KIF13A | 6 | 17779264 | T | G | 19 | T/P;1336 | missense | SNV | no |
|  | TC | KIF18A | 11 | 28119421 | T | C | 44 | E/G;25 | missense | SNV | no |
|  | TC | KLF4 | 9 | 110250475 | G | C | 52 | A/G;67 | missense | SNV | no |
|  | TC | KLHL2 | 4 | 166234414 | A | T | 39 | Y/F;459 | missense | SNV | no |
|  | TC | KLHL33 | 14 | 20897755 | C | A | 13 | A/S;319 | missense | SNV | no |
|  | TC | LMOD2 | 7 | 123296229 | C | A | 28 | A/D;71 | missense | SNV | no |
|  | TC | LRP5L | 22 | 25750586 | G | T | 11 | A/D;211 | missense | SNV | no |
|  | TC | LUZP4 | X | 114536587 | G | T | 12 | G/V;41 | missense | SNV | no |
|  | TC | MAN2A2 | 15 | 91449179 | G | A | 36 | A/T;214 | missense | SNV | no |
|  | TC | MAP2 | 2 | 210559845 | G | T | 26 | G/V;984 | missense | SNV | no |
|  | TC | MAP4K1 | 19 | 39087703 | G | C | 22 | Q/E;638 | missense | SNV | no |
|  | TC | MAP4K1 | 19 | 39087704 | G | T | 22 | Y/*;637 | nonsense | SNV | no |
|  | TC | MAP4K5 | 14 | 50923303 | T | C | 47 | I/V;316 | missense | SNV | no |
|  | TC | MAS1 | 6 | 160328414 | C | T | 65 | H/Y;143 | missense | SNV | no |
|  | TC | MEST | 7 | 130138274 | G | A | 54 | R/Q;164 | missense | SNV | no |
|  | TC | MICAL1 | 6 | 109770618 | C | T | 33 | G/R;501 | missense | SNV | no |
|  | TC | MTSS1 | 8 | 125565634 | C | T | 35 | V/I;623 | missense | SNV | no |
|  | TC | MVP | 16 | 29855872 | C | T | 55 | P/S;565 | missense | SNV | no |
|  | TC | MYBL1 | 8 | 67478411 | T | C | 36 | N/S;673 | missense | SNV | no |
|  | TC | NAB2 | 12 | 57487378 | G | A | 38 | A/T;489 | missense | SNV | no |
|  | TC | NAGPA | 16 | 5075484 | C | A | 53 | D/Y;515 | missense | SNV | no |
|  | TC | DHRSX | X | 2184897 | G | T | 27 | N/K;160 | missense | SNV | yes |
|  | TC | NCOR1 | 17 | 16062154 | C | T | 61 | E/K;218 | missense | SNV | no |
|  | TC | NETO2 | 16 | 47162307 | A | G | 22 | M/T;137 | missense | SNV | no |
|  | TC | NPW | 16 | 2070183 | G | T | 12 | W/L;94 | missense | SNV | no |
|  | TC | NUP93 | 16 | 56868311 | T | C | 53 | M/T;565 | missense | SNV | no |
|  | TC | ODF1 | 8 | 103573011 | * | -TGCAACCCCTGCAGCCCCTGCAACCCG | 88 | CODON_DELETION;218 | frameshift | DEL | no |
|  | TC | ODZ4 | 11 | 78412631 | T | C | 28 | H/R;1676 | missense | SNV | no |
|  | TC | OLFM4 | 13 | 53624819 | C | G | 66 | N/K;482 | missense | SNV | no |
|  | TC | PAMR1 | 11 | 35496217 | C | A | 29 | W/L;152 | missense | SNV | no |
|  | TC | PCIF1 | 20 | 44569165 | G | T | 17 | V/L;101 | missense | SNV | no |
|  | TC | NCAM2 | 21 | 22804428 | A | T | 27 | D/V;494 | missense | SNV | yes |
|  | TC | PCLO | 7 | 82545213 | G | C | 10 | S/C;4030 | missense | SNV | yes |
|  | TC | PCLO | 7 | 82585291 | C | T | 38 | G/R;1660 | missense | SNV | yes |
|  | TC | PER3 | 1 | 7879393 | G | C | 24 | S/T;516 | missense | SNV | no |
|  | TC | PEX2 | 8 | 77912370 | T | C | 66 | ; | silent | SNV | no |
|  | TC | PLCE1 | 10 | 96014764 | T | C | 20 | V/A;1171 | missense | SNV | no |
|  | TC | PITRM1 | 10 | 3182940 | C | T | 22 | G/S;905 | missense | SNV | no |
|  | TC | PLAC1L | 11 | 59811008 | T | A | 19 | L/Q;44 | missense | SNV | no |
|  | TC | PLEC | 8 | 145001445 | G | A | 59 | A/V;1409 | missense | SNV | no |
|  | TC | PLXNA1 | 3 | 126734042 | C | T | 13 | R/C;965 | missense | SNV | no |
|  | TC | PNRC1 | 6 | 89790532 | C | G | 34 | ; | silent | SNV | no |
|  | TC | POGZ | 1 | 151400650 | G | C | 18 | L/V;270 | missense | SNV | no |
|  | TC | POLQ | 3 | 121489813 | C | T | 12 | R/*;1175 | nonsense | SNV | no |
|  | TC | POLR1A | 2 | 86276157 | C | A | 35 | R/S;828 | missense | SNV | yes |
|  | TC | PPAP2B | 1 | 56989950 | T | C | 33 | R/G;192 | missense | SNV | no |
|  | TC | PPFIBP1 | 12 | 27844748 | T | C | 26 | Y/H;924 | missense | SNV | no |
|  | TC | PPP1R3F | X | 49143097 | G | A | 98 | D/N;649 | missense | SNV | no |
|  | TC | PPP2R3B | X | 299360 | G | A | 99 | A/V;519 | missense | SNV | no |
|  | TC | PRR15 | 7 | 29606265 | T | C | 71 | L/P;107 | missense | SNV | no |
|  | TC | PTBP2 | 1 | 97278863 | A | G | 11 | T/A;500 | missense | SNV | no |
|  | TC | PTPRH | 19 | 55708778 | T | C | 64 | N/S;566 | missense | SNV | no |
|  | TC | SPHKAP | 2 | 228973564 | G | T | 16 | S/Y;77 | missense | SNV | yes |
|  | TC | SSPO | 7 | 149481144 | * | -C | 50 | FRAME_SHIFT;876 | frameshift | DEL | yes |
|  | TC | TDRD7 | 9 | 100249540 | A | G | 35 | N/S;1001 | missense | SNV | yes |
|  | TC | TTN | 2 | 179632568 | A | G | 30 | V/A;3130 | missense | SNV | no |
|  | TC | PXDNL | 8 | 52233379 | A | G | 41 | W/R;1409 | missense | SNV | no |
|  | TC | QARS | 3 | 49136609 | * | -TG | 42 | FRAME_SHIFT;564 | frameshift | DEL | no |
|  | TC | RABGAP1 | 9 | 125864028 | G | A | 55 | E/K;1025 | missense | SNV | no |
|  | TC | RANBP3L | 5 | 36301439 | C | T | 34 | R/Q;27 | missense | SNV | no |
|  | TC | RANBP6 | 9 | 6014929 | G | A | 29 | P/S;227 | missense | SNV | no |
|  | TC | RAP1GDS1 | 4 | 99342439 | A | G | 53 | K/R;446 | missense | SNV | no |
|  | TC | RASA1 | 5 | 86672738 | C | G | 67 | S/*;742 | nonsense | SNV | no |
|  | TC | RASA3 | 13 | 114757996 | A | C | 29 | F/C;737 | missense | SNV | no |
|  | TC | TTN | 2 | 179640400 | A | G | 19 | F/S;2064 | missense | SNV | no |
|  | TC | RHCG | 15 | 90020047 | C | T | 25 | R/K;417 | missense | SNV | no |
|  | TC | RNF121 | 11 | 71693925 | G | A | 57 | R/Q;121 | missense | SNV | no |
|  | TC | RORA | 15 | 60803626 | G | A | 43 | H/Y;240 | missense | SNV | no |
|  | TC | RP1L1 | 8 | 10466004 | * | -CTGGGCCTCCCCTTCAGCCTC | 41 | CODON_DELETION;1862 | frameshift | DEL | no |
|  | TC | RXFP4 | 1 | 155912569 | C | T | 56 | R/W;357 | missense | SNV | no |
|  | TC | RYR3 | 15 | 33926842 | G | A | 46 | R/H;1028 | missense | SNV | no |
|  | TC | RYR3 | 15 | 34137080 | * | -AGA | 33 | CODON_DELETION;4438 | frameshift | DEL | no |
|  | TC | SAMD11 | 1 | 879444 | G | A | 14 | A/T;653 | missense | SNV | no |
|  | TC | SERPINI1 | 3 | 167507158 | T | C | 12 | L/P;81 | missense | SNV | no |
|  | TC | SETBP1 | 18 | 42530575 | A | C | 29 | I/L;424 | missense | SNV | no |
|  | TC | SH3BP1 | 22 | 38046687 | C | A | 10 | A/D;518 | missense | SNV | no |
|  | TC | SLITRK1 | 13 | 84454572 | C | G | 27 | M/I;357 | missense | SNV | no |
|  | TC | SLX4 | 16 | 3641175 | T | C | 53 | M/V;822 | missense | SNV | no |
|  | TC | SMPD1 | 11 | 6411931 | * | -CTGGTGCTGGCG | 52 | CODON_DELETION;35 | frameshift | DEL | no |
|  | TC | SPATA16 | 3 | 172835224 | T | C | 33 | I/V;100 | missense | SNV | no |
|  | TC | SPO11 | 20 | 55914080 | A | G | 81 | M/V;294 | missense | SNV | no |
|  | TC | SREBF2 | 22 | 42299103 | C | T | 27 | R/W;1013 | missense | SNV | no |
|  | TC | STRA8 | 7 | 134925404 | T | C | 35 | L/P;65 | missense | SNV | no |
|  | TC | STX10 | 19 | 13256152 | C | T | 51 | A/T;141 | missense | SNV | no |
|  | TC | TBCD | 17 | 80881606 | C | T | 38 | Q/*;753 | nonsense | SNV | no |
|  | TC | TBCK | 4 | 107168396 | * | +TA | 53 | FRAME_SHIFT;277 | frameshift | INS | no |
|  | TC | TBL1XR1 | 3 | 176756181 | C | G | 14 | A/P;323 | missense | SNV | no |
|  | TC | TCHH | 1 | 152082451 | C | T | 45 | R/Q;1081 | missense | SNV | no |
|  | TC | TDRD3 | 13 | 61057948 | G | T | 26 | G/C;179 | missense | SNV | no |
|  | TC | TH | 11 | 2193004 | C | T | 35 | D/N;5 | missense | SNV | no |
|  | TC | TNFRSF17 | 16 | 12059017 | C | T | 44 | ; | silent | SNV | no |
|  | TC | TNS3 | 7 | 47408378 | A | G | 57 | L/P;622 | missense | SNV | no |
|  | TC | TRDN | 6 | 123892134 | G | T | 42 | L/M;56 | missense | SNV | no |
|  | TC | TRHDE | 12 | 72969080 | G | T | 37 | G/V;681 | missense | SNV | no |
|  | TC | TRMT11 | 6 | 126333958 | C | T | 12 | Q/*;323 | nonsense | SNV | no |
|  | TC | TP53 | 17 | 7577108 | C | A | 47 | C/F;277 | missense | SNV | yes |
|  | TC | TUBA3D | 2 | 132238306 | G | T | 11 | C/F;347 | missense | SNV | no |
|  | TC | UBE2O | 17 | 74396376 | A | G | 88 | V/A;336 | missense | SNV | no |
|  | TC | UGT1A9 | 2 | 234580863 | C | T | 62 | H/Y;95 | missense | SNV | no |
|  | TC | UNC13A | 19 | 17778943 | T | A | 34 | M/L;151 | missense | SNV | no |
|  | TC | VWA5B1 | 1 | 20617464 | T | C | 38 | ; | silent | SNV | no |
|  | TC | WSCD2 | 12 | 108600180 | G | A | 52 | R/Q;166 | missense | SNV | no |
|  | TC | XPC | 3 | 14200110 | C | T | 47 | A/T;425 | missense | SNV | no |
|  | TC | ZAN | 7 | 100334609 | G | A | 22 | R/H;144 | missense | SNV | no |
|  | TC | ZAN | 7 | 100373320 | C | A | 41 | P/Q;2019 | missense | SNV | no |
|  | TC | ZNF187 | 6 | 28240012 | G | T | 49 | R/S;105 | missense | SNV | no |
|  | TC | ZPBP2 | 17 | 38027025 | T | A | 79 | V/E;66 | missense | SNV | no |
| 349 | TC | AHNAK2 | 14 | 105418898 | T | C | 39 | M/V;964 | missense | SNV | no |
|  | TC | ASCC3 | 6 | 101173525 | C | A | 15 | D/Y;598 | missense | SNV | no |
|  | TC | C2orf71 | 2 | 29293844 | G | A | 12 | P/L;1095 | missense | SNV | no |
|  | TC | CHCHD8 | 11 | 73587860 | C | G | 10 | ; | silent | SNV | no |
|  | TC | DMD | X | 31697533 | A | G | 10 | Y/H;2611 | missense | SNV | no |
|  | TC | EFR3A | 8 | 133023057 | G | T | 12 | G/V;794 | missense | SNV | no |
|  | TC | EPB41L3 | 18 | 5397170 | C | A | 10 | A/S;910 | missense | SNV | no |
|  | TC | EPHB2 | 1 | 23219500 | C | T | 10 | R/C;518 | missense | SNV | no |
|  | TC | ESRRG | 1 | 216824361 | C | A | 11 | Q/H;186 | missense | SNV | no |
|  | TC | FER1L6 | 8 | 125047606 | T | C | 11 | I/T;792 | missense | SNV | no |
|  | TC | FKBP14 | 7 | 30062371 | C | A | 13 | G/C;87 | missense | SNV | no |
|  | TC | FLJ43860 | 8 | 142459706 | T | A | 18 | Y/F;874 | missense | SNV | no |
|  | TC | HBP1 | 7 | 106826358 | A | G | 15 | K/E;181 | missense | SNV | no |
|  | TC | ICK | 6 | 52876885 | G | T | 15 | S/R;431 | missense | SNV | no |
|  | TC | INO80D | 2 | 206927700 | T | C | 11 | N/S;14 | missense | SNV | no |
|  | TC | NR1D1 | 17 | 38251255 | T | A | 11 | H/L;456 | missense | SNV | no |
|  | TC | NTRK3 | 15 | 88476408 | T | G | 15 | K/T;575 | missense | SNV | no |
|  | TC | ODZ4 | 11 | 78482171 | C | A | 12 | G/V;802 | missense | SNV | no |
|  | TC | PAIP1 | 5 | 43547981 | G | A | 11 | T/I;157 | missense | SNV | no |
|  | TC | PGS1 | 17 | 76400164 | G | T | 11 | A/S;466 | missense | SNV | no |
|  | TC | PTPN2 | 18 | 12794455 | C | T | 11 | R/K;357 | missense | SNV | no |
|  | TC | PTPRC | 1 | 198701642 | A | G | 34 | N/S;698 | missense | SNV | no |
|  | TC | RAP1GAP2 | 17 | 2894653 | C | G | 13 | D/E;292 | missense | SNV | no |
|  | TC | DHRSX | X | 2161129 | C | G | 12 | V/L;247 | missense | SNV | yes |
|  | TC | RHPN1 | 8 | 144462855 | C | T | 14 | A/V;438 | missense | SNV | no |
|  | TC | SSPO | 7 | 149523865 | A | G | 13 | Q/R;4892 | missense | SNV | yes |
|  | TC | SLITRK2 | X | 144904235 | T | A | 10 | L/I;98 | missense | SNV | no |
|  | TC | SPAM1 | 7 | 123594393 | A | T | 13 | T/S;257 | missense | SNV | no |
|  | TC | SPG7 | 16 | 89598913 | G | T | 12 | R/L;398 | missense | SNV | no |
|  | TC | SPRY2 | 13 | 80911599 | C | G | 13 | R/T;81 | missense | SNV | no |
|  | TC | UTS2 | 1 | 7907864 | G | A | 13 | R/C;128 | missense | SNV | no |
| 351 | TC | ADSSL1 | 14 | 105196459 | C | A | 12 | A/D;77 | missense | SNV | no |
|  | TC | ARHGEF7 | 13 | 111870177 | G | T | 10 | G/V;228 | missense | SNV | no |
|  | TC | C17orf107 | 17 | 4803418 | G | T | 11 | S/I;85 | missense | SNV | no |
|  | TC | CHAC1 | 15 | 41245866 | G | T | 10 | D/Y;71 | missense | SNV | no |
|  | TC | COMP | 19 | 18896535 | A | G | 30 | L/P;539 | missense | SNV | no |
|  | TC | DIP2A | 21 | 47918647 | C | T | 48 | H/Y;186 | missense | SNV | no |
|  | TC | DNAJC27 | 2 | 25170555 | G | T | 11 | A/E;251 | missense | SNV | no |
|  | TC | GFRA2 | 8 | 21562637 | C | A | 10 | G/V;302 | missense | SNV | no |
|  | TC | KLLN | 10 | 89622169 | C | T | 42 | V/I;26 | missense | SNV | no |
|  | TC | MEGF10 | 5 | 126791094 | C | A | 11 | D/E;1009 | missense | SNV | no |
|  | TC | NEFH | 22 | 29885586 | * | +AAGTCCCCTGAGAAGGCC | 81 | CODON_INSERTION;653 | frameshift | INS | no |
|  | TC | PTPRF | 1 | 44054405 | G | T | 11 | R/L;228 | missense | SNV | no |
|  | TC | CSMD3 | 8 | 113326713 | C | A | 10 | M/I;2498 | missense | SNV | yes |
|  | TC | DSCAML1 | 11 | 117651436 | G | T | 10 | P/T;106 | missense | SNV | yes |
|  | TC | RIPK4 | 21 | 43162065 | C | A | 10 | D/Y;430 | missense | SNV | no |
|  | TC | SBF1 | 22 | 50898547 | C | A | 10 | A/S;1109 | missense | SNV | no |
|  | TC | USP11 | X | 47102997 | C | A | 10 | L/I;639 | missense | SNV | no |
|  | TC | VPS8 | 3 | 184567809 | A | G | 48 | K/R;251 | missense | SNV | no |
| 384 | TC | ADAM10 | 15 | 59042056 | G | T | 10 | ; | silent | SNV | no |
|  | TC | ADAMTS4 | 1 | 161168612 | C | T | 16 | ; | silent | SNV | no |
|  | TC | ADCYAP1 | 18 | 905461 | C | A | 12 | S/*;25 | nonsense | SNV | no |
|  | TC | ALOXE3 | 17 | 8012661 | C | A | 12 | V/F;597 | missense | SNV | no |
|  | TC | BRCA1 | 17 | 41243483 | A | C | 17 | N/K;1355 | missense | SNV | yes |
|  | TC | BYSL | 6 | 41895158 | G | T | 11 | W/C;105 | missense | SNV | no |
|  | TC | C2orf47 | 2 | 200826641 | G | T | 12 | A/S;263 | missense | SNV | no |
|  | TC | C2orf81 | 2 | 74643352 | A | C | 16 | V/G;15 | missense | SNV | no |
|  | TC | CHST3 | 10 | 73768213 | C | A | 10 | T/N;475 | missense | SNV | no |
|  | TC | CXorf36 | X | 45011178 | C | T | 14 | G/S;341 | missense | SNV | no |
|  | TC | DCHS2 | 4 | 155250847 | C | A | 12 | W/L;794 | missense | SNV | no |
|  | TC | EPB42 | 15 | 43498556 | C | A | 11 | K/N;560 | missense | SNV | no |
|  | TC | EYA4 | 6 | 133802605 | G | T | 10 | E/D;325 | missense | SNV | no |
|  | TC | INSM1 | 20 | 20350400 | A | T | 11 | R/*;497 | nonsense | SNV | no |
|  | TC | NR4A1 | 12 | 52448116 | C | A | 12 | P/T;15 | missense | SNV | no |
|  | TC | RNF113B | 13 | 98829351 | C | A | 15 | S/I;47 | missense | SNV | no |
|  | TC | RYR1 | 19 | 38980896 | C | T | 25 | R/C;1999 | missense | SNV | no |
|  | TC | SMG1 | 16 | 18840660 | G | T | 28 | S/Y;3184 | missense | SNV | no |
|  | TC | SPTBN5 | 15 | 42162068 | C | A | 13 | A/S;1907 | missense | SNV | no |
|  | TC | TSPYL1 | 6 | 116600338 | C | A | 10 | G/V;219 | missense | SNV | no |
|  | TC | UNC93B1 | 11 | 67770523 | C | A | 13 | A/S;121 | missense | SNV | no |
| 402 | TC | AANAT | 17 | 74464945 | C | A | 11 | C/*;84 | nonsense | SNV | no |
|  | TC | AGL | 1 | 100327835 | T | C | 12 | Y/H;106 | missense | SNV | no |
|  | TC | BRSK2 | 11 | 1477840 | G | T | 11 | R/L;667 | missense | SNV | no |
|  | TC | GPRASP1 | X | 101908548 | G | T | 12 | ; | silent | SNV | no |
|  | TC | METTL13 | 1 | 171765689 | G | T | 12 | K/N;631 | missense | SNV | no |
|  | TC | SMG1 | 16 | 18937703 | G | T | 12 | ; | silent | SNV | no |
|  | TC | TEX11 | X | 69844693 | C | A | 13 | V/L;579 | missense | SNV | no |
|  | TC | TRIM7 | 5 | 180622393 | G | T | 12 | L/I;437 | missense | SNV | no |
| 467 | TC | ARID1A | 1 | 27057979 | C | T | 47 | Q/*;563 | nonsense | SNV | yes |
|  | TC | C1orf61 | 1 | 156384531 | G | T | 41 | A/E;29 | missense | SNV | no |
|  | TC | CDH26 | 20 | 58564209 | G | T | 11 | S/I;425 | missense | SNV | no |
|  | TC | CNNM1 | 10 | 101090577 | T | C | 33 | I/T;478 | missense | SNV | no |
|  | TC | FGG | 4 | 155527906 | C | G | 51 | W/C;360 | missense | SNV | no |
|  | TC | LCT | 2 | 136566013 | C | A | 11 | A/S;1302 | missense | SNV | no |
|  | TC | LELP1 | 1 | 153177371 | C | T | 35 | S/L;63 | missense | SNV | no |
|  | TC | NEFH | 22 | 29885583 | * | +GCCAAGTCCCCTGAGAAG | 74 | CODON_INSERTION;652 | frameshift | INS | no |
|  | TC | ODZ3 | 4 | 183601422 | G | A | 50 | R/Q;520 | missense | SNV | no |
|  | TC | PLK2 | 5 | 57750835 | G | A | 44 | P/L;590 | missense | SNV | no |
|  | TC | TCF20 | 22 | 42608962 | C | T | 48 | G/R;784 | missense | SNV | no |
|  | TC | TDRD1 | 10 | 115963255 | G | C | 17 | A/P;304 | missense | SNV | no |
|  | TC | TTN | 2 | 179412436 | A | G | 14 | I/T;29665 | missense | SNV | no |
|  | TC | TNC | 9 | 117825418 | C | A | 11 | A/S;1271 | missense | SNV | no |
|  | TC | YARS | 1 | 33252631 | T | A | 43 | K/*;244 | nonsense | SNV | no |
| 497 | TC | ADAT1 | 16 | 75646634 | T | C | 10 | R/G;184 | missense | SNV | no |
|  | TC | BEND6 | 6 | 56882195 | T | C | 23 | I/T;237 | missense | SNV | no |
|  | TC | CETN2 | X | 151996426 | G | T | 12 | Q/K;160 | missense | SNV | no |
|  | TC | CREBBP | 16 | 3781374 | C | A | 10 | R/L;1664 | missense | SNV | yes |
|  | TC | FARS2 | 6 | 5261670 | G | T | 10 | ; | silent | SNV | no |
|  | TC | FUK | 16 | 70508719 | G | T | 11 | A/S;728 | missense | SNV | no |
|  | TC | HERC2 | 15 | 28370216 | C | A | 11 | G/V;4309 | missense | SNV | no |
|  | TC | IL1RL2 | 2 | 102836343 | C | A | 12 | T/N;286 | missense | SNV | no |
|  | TC | PDE9A | 21 | 44180504 | C | A | 53 | Q/K;322 | missense | SNV | no |
|  | TC | SDR9C7 | 12 | 57324113 | C | A | 12 | G/C;153 | missense | SNV | no |
|  | TC | STRN3 | 14 | 31405717 | C | A | 13 | R/L;277 | missense | SNV | no |
|  | TC | XPNPEP2 | X | 128884540 | C | T | 14 | T/M;245 | missense | SNV | no |
| 034 | AC | ADAMTS6 | 5 | 64625307 | A | C | 29 | L/W;374 | missense | SNV | no |
|  | AC | ATAD5 | 17 | 29196317 | G | A | 43 | S/N;1122 | missense | SNV | no |
|  | AC | BCLAF1 | 6 | 136599804 | C | A | 14 | G/V;72 | missense | SNV | no |
|  | AC | EIF2AK2 | 2 | 37368696 | C | A | 13 | G/V;130 | missense | SNV | no |
|  | AC | IL21 | 4 | 123542046 | G | A | 55 | Q/*;41 | nonsense | SNV | no |
|  | AC | LRP1 | 12 | 57594835 | C | G | 27 | P/R;3415 | missense | SNV | no |
|  | AC | NR5A2 | 1 | 200014706 | C | A | 49 | L/I;153 | missense | SNV | no |
|  | AC | OSBPL5 | 11 | 3140788 | G | T | 43 | S/Y;227 | missense | SNV | no |
|  | AC | RC3H1 | 1 | 173933996 | G | A | 44 | P/S;533 | missense | SNV | no |
|  | AC | RNMT | 18 | 13734506 | A | G | 60 | N/S;154 | missense | SNV | no |
|  | AC | SMARCA4 | 19 | 11132519 | C | T | 27 | T/I;912 | missense | SNV | yes |
|  | AC | TATDN3 | 1 | 212965291 | G | A | 50 | D/N;10 | missense | SNV | no |
|  | AC | VPS52 | 6 | 33231579 | G | T | 42 | N/K;566 | missense | SNV | no |
|  | AC | YLPM1 | 14 | 75248778 | G | T | 43 | G/C;678 | missense | SNV | no |
| 364 | AC | ATHL1 | 11 | 293394 | G | A | 25 | D/N;458 | missense | SNV | no |
|  | AC | C10orf76 | 10 | 103607356 | C | T | 34 | V/I;667 | missense | SNV | no |
|  | AC | CLGN | 4 | 141321607 | G | A | 10 | P/S;200 | missense | SNV | no |
|  | AC | IL4I1 | 19 | 50393733 | G | A | 19 | Q/*;322 | nonsense | SNV | no |
|  | AC | PIK3R3 | 1 | 46532720 | C | T | 14 | G/S;120 | missense | SNV | no |
|  | AC | PRR25 | 16 | 863340 | G | T | 12 | A/S;230 | missense | SNV | no |
|  | AC | PSME4 | 2 | 54158972 | C | T | 44 | R/K;439 | missense | SNV | no |
|  | AC | SMAD5 | 5 | 135489478 | T | C | 24 | F/S;10 | missense | SNV | no |
|  | AC | TDRD7 | 9 | 100243111 | C | A | 12 | A/D;768 | missense | SNV | yes |
|  | AC | SPTBN2 | 11 | 66468587 | G | C | 11 | L/V;995 | missense | SNV | no |
|  | AC | SYNJ2 | 6 | 158485873 | G | T | 31 | D/Y;459 | missense | SNV | no |
|  | AC | TBC1D4 | 13 | 75936743 | C | G | 45 | V/L;167 | missense | SNV | no |
|  | AC | UBR4 | 1 | 19419877 | G | T | 24 | L/M;4681 | missense | SNV | no |
|  | AC | WEE2 | 7 | 141424973 | G | A | 12 | E/K;457 | missense | SNV | no |
|  | AC | ZYG11B | 1 | 53236937 | C | T | 22 | L/F;148 | missense | SNV | no |
| 389 | AC | AADAT | 4 | 170990306 | A | G | 10 | S/P;266 | missense | SNV | no |
|  | AC | AAGAB | 15 | 67546997 | G | A | 39 | ; | silent | SNV | no |
|  | AC | ABL1 | 9 | 133760764 | G | T | 33 | K/N;1048 | missense | SNV | no |
|  | AC | ABRA | 8 | 107773656 | C | T | 39 | W/*;252 | nonsense | SNV | no |
|  | AC | ACO1 | 9 | 32433765 | G | C | 13 | D/H;631 | missense | SNV | no |
|  | AC | ACTN2 | 1 | 236890988 | G | A | 58 | G/S;183 | missense | SNV | no |
|  | AC | ACTN2 | 1 | 236890989 | G | T | 58 | G/V;183 | missense | SNV | no |
|  | AC | ADAMTS12 | 5 | 33527364 | G | T | 34 | P/T;1572 | missense | SNV | no |
|  | AC | ADAMTS20 | 12 | 43846358 | C | G | 34 | G/A;634 | missense | SNV | yes |
|  | AC | ADAMTS5 | 21 | 28337933 | G | A | 68 | R/C;260 | missense | SNV | no |
|  | AC | ADCY10 | 1 | 167830234 | C | G | 26 | A/P;562 | missense | SNV | no |
|  | AC | AEBP1 | 7 | 44152242 | T | A | 97 | L/Q;768 | missense | SNV | no |
|  | AC | AGTPBP1 | 9 | 88162190 | T | C | 48 | Y/C;1132 | missense | SNV | no |
|  | AC | AHCYL2 | 7 | 129043236 | G | T | 64 | G/V;312 | missense | SNV | no |
|  | AC | ALDH1A3 | 15 | 101448616 | C | G | 24 | I/M;465 | missense | SNV | no |
|  | AC | ALOX5AP | 13 | 31338086 | C | A | 81 | P/H;167 | missense | SNV | no |
|  | AC | ALOXE3 | 17 | 8021365 | G | C | 97 | P/A;114 | missense | SNV | no |
|  | AC | AMOT | X | 112066113 | T | A | 41 | Q/L;81 | missense | SNV | no |
|  | AC | AMPH | 7 | 38514992 | G | T | 76 | Q/K;177 | missense | SNV | no |
|  | AC | AMY2B | 1 | 104116888 | G | T | 42 | D/Y;251 | missense | SNV | no |
|  | AC | ANGPTL1 | 1 | 178834232 | T | C | 28 | Q/R;227 | missense | SNV | no |
|  | AC | ANK2 | 4 | 114158194 | G | A | 38 | V/M;179 | missense | SNV | no |
|  | AC | ANK2 | 4 | 114275248 | C | A | 55 | P/H;1825 | missense | SNV | no |
|  | AC | ANPEP | 15 | 90335391 | C | A | 64 | R/M;843 | missense | SNV | no |
|  | AC | APC | 5 | 112176209 | C | T | 92 | R/W;1640 | missense | SNV | yes |
|  | AC | APOB | 2 | 21225123 | G | T | 30 | P/T;4391 | missense | SNV | no |
|  | AC | APOBR | 16 | 28508067 | G | T | 22 | G/C;569 | missense | SNV | no |
|  | AC | ARFIP2 | 11 | 6498991 | C | T | 39 | R/Q;309 | missense | SNV | no |
|  | AC | ARHGAP23 | 17 | 36623060 | G | T | 25 | G/V;379 | missense | SNV | no |
|  | AC | ARHGAP32 | 11 | 128840289 | T | A | 42 | M/L;1593 | missense | SNV | no |
|  | AC | ARHGAP35 | 19 | 47423745 | A | T | 92 | K/*;605 | nonsense | SNV | no |
|  | AC | ATHL1 | 11 | 290765 | C | G | 50 | D/E;186 | missense | SNV | no |
|  | AC | ATP6V1H | 8 | 54669210 | C | G | 53 | L/F;394 | missense | SNV | no |
|  | AC | BCMO1 | 16 | 81324086 | A | T | 42 | L/F;516 | missense | SNV | no |
|  | AC | BNC1 | 15 | 83935749 | C | T | 28 | D/N;92 | missense | SNV | no |
|  | AC | BOD1L1 | 4 | 13604053 | C | A | 67 | A/S;1491 | missense | SNV | no |
|  | AC | BPIFB2 | 20 | 31607525 | C | T | 34 | S/L;350 | missense | SNV | no |
|  | AC | BPIFB2 | 20 | 31607527 | G | T | 32 | A/S;351 | missense | SNV | no |
|  | AC | BRCA1 | 17 | 41245803 | G | A | 38 | T/M;582 | missense | SNV | yes |
|  | AC | BSCL2 | 11 | 62457984 | G | A | 40 | S/F;415 | missense | SNV | no |
|  | AC | BSN | 3 | 49692855 | C | T | 50 | R/W;1956 | missense | SNV | no |
|  | AC | BZRAP1 | 17 | 56386093 | T | C | 15 | I/V;1514 | missense | SNV | no |
|  | AC | C10orf140 | 10 | 21808457 | G | T | 30 | ; | silent | SNV | no |
|  | AC | C11orf24 | 11 | 68029429 | T | C | 36 | E/G;345 | missense | SNV | no |
|  | AC | C11orf63 | 11 | 122805710 | G | T | 59 | G/*;521 | nonsense | SNV | no |
|  | AC | C12orf42 | 12 | 103696037 | G | A | 18 | P/L;311 | missense | SNV | no |
|  | AC | C14orf126 | 14 | 31917507 | T | C | 91 | K/R;112 | missense | SNV | no |
|  | AC | C17orf105 | 17 | 41861230 | C | G | 30 | T/S;155 | missense | SNV | no |
|  | AC | C1S | 12 | 7177829 | C | A | 48 | F/L;647 | missense | SNV | no |
|  | AC | C20orf132 | 20 | 35731152 | C | T | 58 | R/Q;1023 | missense | SNV | no |
|  | AC | C20orf3 | 20 | 24959472 | T | C | 19 | K/E;87 | missense | SNV | no |
|  | AC | C2CD2L | 11 | 118985075 | G | T | 56 | V/L;638 | missense | SNV | no |
|  | AC | C2orf73 | 2 | 54562060 | G | T | 46 | G/*;45 | nonsense | SNV | no |
|  | AC | C3orf38 | 3 | 88205552 | A | G | 62 | I/V;253 | missense | SNV | no |
|  | AC | C3orf55 | 3 | 157289043 | G | A | 14 | G/E;54 | missense | SNV | no |
|  | AC | C3orf58 | 3 | 143704547 | C | G | 31 | L/V;274 | missense | SNV | no |
|  | AC | C3orf77 | 3 | 44286120 | G | C | 64 | E/Q;708 | missense | SNV | no |
|  | AC | C4orf21 | 4 | 113539566 | C | A | 33 | E/D;544 | missense | SNV | no |
|  | AC | C7 | 5 | 40981602 | C | G | 40 | A/G;820 | missense | SNV | no |
|  | AC | C7orf58 | 7 | 120884293 | C | A | 22 | N/K;737 | missense | SNV | no |
|  | AC | C8B | 1 | 57420381 | C | T | 50 | G/R;171 | missense | SNV | no |
|  | AC | C9orf3 | 9 | 97522435 | G | A | 60 | G/R;124 | missense | SNV | no |
|  | AC | CAMK1G | 1 | 209779014 | T | A | 33 | L/I;144 | missense | SNV | no |
|  | AC | CAMK1G | 1 | 209781262 | G | A | 62 | V/I;207 | missense | SNV | no |
|  | AC | CAP2 | 6 | 17543107 | A | G | 15 | T/A;348 | missense | SNV | no |
|  | AC | CCDC169-SOHLH2 | 13 | 36744834 | G | A | 93 | T/I;441 | missense | SNV | no |
|  | AC | CD248 | 11 | 66082878 | T | A | 65 | T/S;541 | missense | SNV | no |
|  | AC | CD300A | 17 | 72477888 | C | A | 43 | H/Q;230 | missense | SNV | no |
|  | AC | CD300LF | 17 | 72691938 | T | C | 54 | T/A;215 | missense | SNV | no |
|  | AC | CDC42BPB | 14 | 103412855 | T | A | 86 | Y/F;1233 | missense | SNV | no |
|  | AC | CDKL2 | 4 | 76551111 | C | A | 19 | C/F;21 | missense | SNV | no |
|  | AC | CEBPG | 19 | 33870429 | A | T | 69 | N/I;95 | missense | SNV | no |
|  | AC | CEL | 9 | 135946604 | C | A | 29 | P/H;575 | missense | SNV | no |
|  | AC | CELF4 | 18 | 34854812 | C | A | 34 | A/S;205 | missense | SNV | no |
|  | AC | CEP128 | 14 | 81259175 | G | T | 38 | H/N;497 | missense | SNV | no |
|  | AC | CEP350 | 1 | 179984989 | A | T | 28 | K/N;686 | missense | SNV | no |
|  | AC | CEP63 | 3 | 134214159 | G | C | 34 | ; | silent | SNV | no |
|  | AC | CEP85 | 1 | 26586128 | * | -T | 52 | FRAME_SHIFT;392 | frameshift | DEL | no |
|  | AC | CHD1 | 5 | 98193986 | T | C | 92 | Q/R;1562 | missense | SNV | no |
|  | AC | CHD6 | 20 | 40049149 | T | G | 33 | Q/H;2042 | missense | SNV | no |
|  | AC | CHST3 | 10 | 73767962 | * | -C | 39 | FRAME_SHIFT;391 | frameshift | DEL | no |
|  | AC | CHST4 | 16 | 71571025 | C | G | 64 | R/G;149 | missense | SNV | no |
|  | AC | CHUK | 10 | 101982683 | A | C | 54 | I/M;85 | missense | SNV | no |
|  | AC | CLDN9 | 16 | 3062935 | G | C | 63 | ; | silent | SNV | no |
|  | AC | COG2 | 1 | 230819369 | G | T | 33 | E/*;406 | nonsense | SNV | no |
|  | AC | CORO6 | 17 | 27943314 | T | C | 35 | M/V;349 | missense | SNV | no |
|  | AC | CPS1 | 2 | 211525341 | G | C | 36 | D/H;1303 | missense | SNV | no |
|  | AC | CRISPLD2 | 16 | 84914181 | C | G | 12 | I/M;432 | missense | SNV | no |
|  | AC | CRYGB | 2 | 209010621 | C | A | 48 | W/C;43 | missense | SNV | no |
|  | AC | CRYM | 16 | 21273360 | C | A | 34 | G/W;265 | missense | SNV | no |
|  | AC | CSGALNACT2 | 10 | 43659341 | G | C | 45 | L/F;336 | missense | SNV | no |
|  | AC | CTNNA2 | 2 | 80097059 | C | G | 29 | Q/E;195 | missense | SNV | no |
|  | AC | DCC | 18 | 50432530 | C | A | 58 | Q/K;177 | missense | SNV | no |
|  | AC | CTRC | 1 | 15772107 | C | T | 45 | P/S;219 | missense | SNV | no |
|  | AC | CUZD1 | 10 | 124598632 | C | A | 49 | E/*;117 | nonsense | SNV | no |
|  | AC | CYP2B6 | 19 | 41516029 | C | A | 15 | P/H;318 | missense | SNV | no |
|  | AC | CYTH1 | 17 | 76677067 | C | T | 34 | D/N;317 | missense | SNV | no |
|  | AC | DACH2 | X | 85994840 | G | C | 90 | V/L;399 | missense | SNV | no |
|  | AC | DBC1 | 9 | 121971010 | G | C | 36 | L/V;378 | missense | SNV | no |
|  | AC | DBT | 1 | 100680452 | A | G | 35 | L/P;287 | missense | SNV | no |
|  | AC | DCAF4L2 | 8 | 88885810 | C | T | 39 | M/I;130 | missense | SNV | no |
|  | AC | DCAF4L2 | 8 | 88885612 | * | -C | 39 | FRAME_SHIFT;196 | frameshift | DEL | no |
|  | AC | DCHS1 | 11 | 6646099 | C | A | 43 | V/L;2383 | missense | SNV | no |
|  | AC | DET1 | 15 | 89056329 | C | A | 26 | Q/H;513 | missense | SNV | no |
|  | AC | DGKG | 3 | 185990130 | A | G | 24 | C/R;305 | missense | SNV | no |
|  | AC | DHDH | 19 | 49442839 | G | A | 95 | R/Q;167 | missense | SNV | no |
|  | AC | DLGAP2 | 8 | 1626589 | A | G | 66 | Y/C;753 | missense | SNV | no |
|  | AC | DLL1 | 6 | 170597379 | C | A | 39 | E/D;206 | missense | SNV | no |
|  | AC | DLX2 | 2 | 172965347 | T | C | 23 | Q/R;304 | missense | SNV | no |
|  | AC | DNAJC25 | 9 | 114411850 | G | C | 51 | E/Q;203 | missense | SNV | no |
|  | AC | DNMT3A | 2 | 25469930 | T | A | 32 | E/V;371 | missense | SNV | yes |
|  | AC | DNTT | 10 | 98097951 | T | A | 39 | D/E;500 | missense | SNV | no |
|  | AC | DOCK1 | 10 | 128798488 | T | A | 41 | I/N;301 | missense | SNV | no |
|  | AC | DOCK10 | 2 | 225714241 | G | T | 36 | P/T;740 | missense | SNV | no |
|  | AC | DONSON | 21 | 34956958 | C | A | 29 | M/I;241 | missense | SNV | no |
|  | AC | DPCR1 | 6 | 30919516 | C | T | 94 | S/L;1092 | missense | SNV | no |
|  | AC | DPPA3 | 12 | 7869569 | T | A | 17 | S/T;126 | missense | SNV | no |
|  | AC | DYSF | 2 | 71839794 | C | G | 68 | Y/*;1415 | nonsense | SNV | no |
|  | AC | EAF2 | 3 | 121554208 | C | G | 65 | Q/E;26 | missense | SNV | no |
|  | AC | EBF1 | 5 | 158141154 | C | A | 82 | A/S;388 | missense | SNV | no |
|  | AC | EBLN2 | 3 | 73111482 | * | +A | 36 | FRAME_SHIFT;84 | frameshift | INS | no |
|  | AC | ECE2 | 3 | 183994728 | C | A | 24 | L/M;177 | missense | SNV | no |
|  | AC | EFCAB4B | 12 | 3747383 | C | A | 63 | Q/H;503 | missense | SNV | no |
|  | AC | EIF3E | 8 | 109226925 | G | T | 23 | F/L;324 | missense | SNV | no |
|  | AC | ELOVL2 | 6 | 10990004 | C | T | 96 | G/S;233 | missense | SNV | no |
|  | AC | ENGASE | 17 | 77073546 | G | T | 47 | E/*;61 | nonsense | SNV | no |
|  | AC | EPB41L3 | 18 | 5395092 | C | T | 48 | G/R;1043 | missense | SNV | no |
|  | AC | EPHB1 | 3 | 134670293 | G | C | 36 | Q/H;68 | missense | SNV | yes |
|  | AC | ERI1 | 8 | 8887404 | C | T | 91 | R/*;304 | nonsense | SNV | no |
|  | AC | ERLEC1 | 2 | 54014118 | C | T | 33 | ; | silent | SNV | no |
|  | AC | ERMN | 2 | 158182162 | G | A | 60 | P/L;11 | missense | SNV | no |
|  | AC | ETV3 | 1 | 157106117 | T | C | 55 | K/E;10 | missense | SNV | no |
|  | AC | EYS | 6 | 66205296 | T | G | 26 | D/A;3 | missense | SNV | no |
|  | AC | FAM126A | 7 | 23053693 | C | T | 82 | ; | silent | SNV | no |
|  | AC | FAM151A | 1 | 55076138 | * | -CACTCCACATTCAGACCGTCATCCCCAGG | 34 | FRAME_SHIFT;335 | frameshift | DEL | no |
|  | AC | FAM161A | 2 | 62066926 | C | T | 30 | G/R;405 | missense | SNV | no |
|  | AC | FAM181B | 11 | 82443685 | C | G | 45 | G/R;363 | missense | SNV | no |
|  | AC | FAM221B | 9 | 35825795 | C | G | 28 | D/H;122 | missense | SNV | no |
|  | AC | FAM5C | 1 | 190195353 | T | A | 56 | K/*;274 | nonsense | SNV | no |
|  | AC | FAM78B | 1 | 166039818 | G | T | 31 | A/E;149 | missense | SNV | no |
|  | AC | FAT3 | 11 | 92531065 | C | A | 32 | P/Q;1629 | missense | SNV | no |
|  | AC | FAT4 | 4 | 126412291 | C | T | 23 | Q/*;4772 | nonsense | SNV | no |
|  | AC | FBN2 | 5 | 127729055 | T | C | 91 | Y/C;413 | missense | SNV | no |
|  | AC | FBXO27 | 19 | 39516073 | A | G | 20 | I/T;277 | missense | SNV | no |
|  | AC | FCGR3A | 1 | 161512857 | G | C | 27 | T/R;273 | missense | SNV | no |
|  | AC | FLG | 1 | 152284898 | G | T | 55 | H/N;822 | missense | SNV | no |
|  | AC | FLNB | 3 | 58095876 | C | G | 39 | I/M;821 | missense | SNV | no |
|  | AC | FNDC1 | 6 | 159672487 | C | T | 84 | T/I;1663 | missense | SNV | no |
|  | AC | FNDC7 | 1 | 109270535 | T | A | 24 | V/E;406 | missense | SNV | no |
|  | AC | FRMPD1 | 9 | 37744595 | C | A | 22 | P/T;856 | missense | SNV | no |
|  | AC | FRRS1 | 1 | 100178043 | C | A | 43 | C/F;451 | missense | SNV | no |
|  | AC | FZD10 | 12 | 130648496 | G | T | 53 | A/S;337 | missense | SNV | no |
|  | AC | GABRB3 | 15 | 26825562 | C | A | 87 | G/W;196 | missense | SNV | no |
|  | AC | GABRE | X | 151123319 | T | C | 54 | M/V;459 | missense | SNV | no |
|  | AC | GABRE | X | 151123892 | T | A | 34 | N/I;362 | missense | SNV | no |
|  | AC | GART | 21 | 34889688 | C | A | 48 | D/Y;644 | missense | SNV | no |
|  | AC | GAS2L3 | 12 | 101018310 | A | T | 29 | Q/L;576 | missense | SNV | no |
|  | AC | GBA3 | 4 | 22749450 | C | A | 24 | A/D;273 | missense | SNV | no |
|  | AC | GFPT2 | 5 | 179757710 | A | G | 90 | I/T;175 | missense | SNV | no |
|  | AC | GJA1 | 6 | 121768066 | T | A | 84 | W/R;25 | missense | SNV | no |
|  | AC | GOLGA3 | 12 | 133374936 | T | A | 63 | Q/L;643 | missense | SNV | no |
|  | AC | GPR123 | 10 | 134942871 | G | A | 54 | M/I;513 | missense | SNV | no |
|  | AC | GPR128 | 3 | 100362117 | G | T | 26 | E/*;236 | nonsense | SNV | no |
|  | AC | GPR144 | 9 | 127231555 | T | A | 57 | V/E;788 | missense | SNV | no |
|  | AC | GPR152 | 11 | 67219849 | G | A | 30 | S/F;116 | missense | SNV | no |
|  | AC | GPR162 | 12 | 6936176 | C | G | 31 | P/R;525 | missense | SNV | no |
|  | AC | GPR17 | 2 | 128408875 | C | A | 59 | A/D;217 | missense | SNV | no |
|  | AC | GPR37 | 7 | 124404409 | C | G | 56 | E/Q;208 | missense | SNV | no |
|  | AC | GPRC5C | 17 | 72436014 | A | T | 55 | Q/H;78 | missense | SNV | no |
|  | AC | GRIA2 | 4 | 158284032 | G | T | 35 | V/L;830 | missense | SNV | no |
|  | AC | HABP2 | 10 | 115340445 | G | T | 54 | A/S;278 | missense | SNV | no |
|  | AC | HACL1 | 3 | 15642680 | T | C | 47 | I/V;35 | missense | SNV | no |
|  | AC | HECW2 | 2 | 197171267 | G | A | 39 | P/L;920 | missense | SNV | no |
|  | AC | HELB | 12 | 66715778 | * | -G | 39 | FRAME_SHIFT;736 | frameshift | DEL | no |
|  | AC | HHATL | 3 | 42741287 | G | T | 41 | S/Y;46 | missense | SNV | no |
|  | AC | HIP1 | 7 | 75183514 | T | C | 35 | S/G;686 | missense | SNV | no |
|  | AC | HLX | 1 | 221053410 | G | A | 28 | A/T;71 | missense | SNV | no |
|  | AC | HMGB4 | 1 | 34329885 | G | T | 52 | Q/H;31 | missense | SNV | no |
|  | AC | HMGXB3 | 5 | 149412153 | C | A | 84 | T/N;657 | missense | SNV | no |
|  | AC | HNMT | 2 | 138771448 | C | A | 33 | D/E;209 | missense | SNV | no |
|  | AC | HRH4 | 18 | 22057234 | A | T | 14 | E/V;294 | missense | SNV | no |
|  | AC | HRH4 | 18 | 22057233 | G | T | 15 | E/*;294 | nonsense | SNV | no |
|  | AC | HSPA6 | 1 | 161496356 | * | -C | 59 | FRAME_SHIFT;636 | frameshift | DEL | no |
|  | AC | HSPB2 | 11 | 111784375 | G | A | 42 | R/Q;102 | missense | SNV | no |
|  | AC | IFNAR1 | 21 | 34721752 | A | G | 28 | Y/C;349 | missense | SNV | no |
|  | AC | IFT172 | 2 | 27699524 | * | -AATATC | 67 | CODON_DELETION;464 | frameshift | DEL | no |
|  | AC | IL36A | 2 | 113763640 | C | A | 30 | P/T;34 | missense | SNV | no |
|  | AC | IL36A | 2 | 113765515 | C | T | 63 | P/L;124 | missense | SNV | no |
|  | AC | INSRR | 1 | 156814259 | C | A | 28 | G/V;911 | missense | SNV | no |
|  | AC | INTS2 | 17 | 59955359 | G | A | 31 | A/V;790 | missense | SNV | no |
|  | AC | IQSEC1 | 3 | 12977738 | C | A | 46 | A/S;260 | missense | SNV | no |
|  | AC | IQUB | 7 | 123143262 | G | A | 43 | Q/*;230 | nonsense | SNV | no |
|  | AC | IRAK2 | 3 | 10258727 | * | -GGTCA | 40 | FRAME_SHIFT;300 | frameshift | DEL | no |
|  | AC | IRX1 | 5 | 3599846 | G | C | 18 | A/P;262 | missense | SNV | no |
|  | AC | ITGA2B | 17 | 42455730 | C | A | 52 | E/D;698 | missense | SNV | no |
|  | AC | ITGAD | 16 | 31434689 | A | T | 45 | Q/L;959 | missense | SNV | no |
|  | AC | ITGAL | 16 | 30528381 | A | T | 23 | I/F;984 | missense | SNV | no |
|  | AC | ITPR1 | 3 | 4776859 | G | A | 69 | G/R;1774 | missense | SNV | no |
|  | AC | KDR | 4 | 55963874 | C | G | 25 | D/H;857 | missense | SNV | yes |
|  | AC | KDELR1 | 19 | 48892817 | G | C | 95 | P/R;115 | missense | SNV | no |
|  | AC | KDM4D | 11 | 94731918 | G | C | 51 | R/T;461 | missense | SNV | no |
|  | AC | KIAA0090 | 1 | 19550006 | G | T | 39 | R/S;754 | missense | SNV | no |
|  | AC | KIAA0146 | 8 | 48511616 | G | C | 21 | V/L;468 | missense | SNV | no |
|  | AC | KIAA0368 | 9 | 114140879 | C | A | 57 | R/L;1490 | missense | SNV | no |
|  | AC | KIAA0913 | 10 | 75552543 | G | T | 44 | G/V;749 | missense | SNV | no |
|  | AC | KIAA1211 | 4 | 57182583 | C | A | 55 | P/Q;972 | missense | SNV | no |
|  | AC | KIF17 | 1 | 21031048 | G | T | 45 | P/T;339 | missense | SNV | no |
|  | AC | KIF19 | 17 | 72346713 | G | T | 39 | G/C;463 | missense | SNV | no |
|  | AC | KLHL1 | 13 | 70549858 | C | A | 37 | V/F;192 | missense | SNV | no |
|  | AC | KLHL34 | X | 21674568 | C | T | 100 | D/N;447 | missense | SNV | no |
|  | AC | KLK10 | 19 | 51518038 | * | -TGGAGCGTAGCATCTGGATCAGT | 86 | FRAME_SHIFT;276 | frameshift | DEL | no |
|  | AC | LARP6 | 15 | 71125244 | C | A | 21 | R/M;208 | missense | SNV | no |
|  | AC | LCN2 | 9 | 130915381 | C | A | 32 | Q/K;194 | missense | SNV | no |
|  | AC | LCT | 2 | 136567131 | G | T | 63 | P/H;929 | missense | SNV | no |
|  | AC | LMNA | 1 | 156105026 | G | C | 51 | A/P;287 | missense | SNV | no |
|  | AC | LPO | 17 | 56326931 | A | C | 38 | K/Q;150 | missense | SNV | no |
|  | AC | MACC1 | 7 | 20201427 | T | A | 11 | E/V;20 | missense | SNV | no |
|  | AC | MAGEB3 | X | 30254850 | G | T | 91 | R/L;270 | missense | SNV | no |
|  | AC | MAP2 | 2 | 210543334 | G | T | 33 | E/*;101 | nonsense | SNV | no |
|  | AC | MAP2K5 | 15 | 67878245 | G | T | 24 | E/*;114 | nonsense | SNV | no |
|  | AC | MAP3K1 | 5 | 56161248 | C | A | 28 | P/T;373 | missense | SNV | no |
|  | AC | MAPK12 | 22 | 50691859 | C | A | 89 | A/S;359 | missense | SNV | no |
|  | AC | MARCH11 | 5 | 16067853 | C | A | 94 | W/C;312 | missense | SNV | no |
|  | AC | MBD5 | 2 | 149247052 | C | G | 64 | P/R;1051 | missense | SNV | no |
|  | AC | MCOLN3 | 1 | 85484879 | T | A | 51 | D/V;530 | missense | SNV | no |
|  | AC | MDGA2 | 14 | 47389358 | C | A | 77 | A/S;699 | missense | SNV | no |
|  | AC | MFGE8 | 15 | 89442937 | C | G | 73 | D/H;326 | missense | SNV | no |
|  | AC | MIB2 | 1 | 1563532 | A | C | 23 | H/P;720 | missense | SNV | no |
|  | AC | MLL3 | 7 | 151947998 | C | G | 21 | E/Q;559 | missense | SNV | yes |
|  | AC | MMP17 | 12 | 132335502 | T | C | 35 | W/R;499 | missense | SNV | no |
|  | AC | MMRN1 | 4 | 90856952 | G | T | 33 | Q/H;707 | missense | SNV | no |
|  | AC | MPG | 16 | 135616 | G | A | 68 | R/H;246 | missense | SNV | no |
|  | AC | MPL | 1 | 43803551 | C | G | 15 | S/C;11 | missense | SNV | yes |
|  | AC | MRVI1 | 11 | 10624729 | C | T | 30 | V/I;605 | missense | SNV | no |
|  | AC | MSR1 | 8 | 16012593 | C | T | 95 | R/Q;293 | missense | SNV | no |
|  | AC | MYT1 | 20 | 62839001 | C | T | 25 | A/V;151 | missense | SNV | no |
|  | AC | MYT1L | 2 | 1926207 | G | T | 55 | A/E;445 | missense | SNV | no |
|  | AC | NAP1L3 | X | 92928102 | T | G | 56 | T/P;68 | missense | SNV | no |
|  | AC | NARG2 | 15 | 60741755 | C | A | 48 | V/F;471 | missense | SNV | no |
|  | AC | NAV3 | 12 | 78443849 | G | A | 58 | M/I;700 | missense | SNV | no |
|  | AC | NAV3 | 12 | 78583895 | A | T | 29 | T/S;2041 | missense | SNV | no |
|  | AC | NBPF1 | 1 | 16935222 | C | T | 12 | ; | silent | SNV | no |
|  | AC | NDRG3 | 20 | 35310946 | C | T | 17 | G/E;140 | missense | SNV | no |
|  | AC | NDRG3 | 20 | 35310947 | C | T | 17 | G/R;140 | missense | SNV | no |
|  | AC | NEB | 2 | 152521975 | C | T | 23 | A/T;1704 | missense | SNV | no |
|  | AC | NEB | 2 | 152408267 | G | T | 64 | Y/*;6643 | nonsense | SNV | no |
|  | AC | NID1 | 1 | 236193050 | C | A | 40 | G/V;513 | missense | SNV | no |
|  | AC | NID2 | 14 | 52526877 | C | G | 43 | L/F;244 | missense | SNV | no |
|  | AC | NIM1 | 5 | 43280191 | G | T | 13 | G/V;224 | missense | SNV | no |
|  | AC | NIM1 | 5 | 43280388 | G | A | 39 | V/I;290 | missense | SNV | no |
|  | AC | NINL | 20 | 25456814 | T | A | 31 | Q/L;1038 | missense | SNV | no |
|  | AC | NIPBL | 5 | 36984847 | G | A | 12 | R/K;522 | missense | SNV | no |
|  | AC | NKD1 | 16 | 50583452 | G | T | 55 | G/C;60 | missense | SNV | no |
|  | AC | NLRC5 | 16 | 57060346 | C | G | 46 | F/L;497 | missense | SNV | no |
|  | AC | NLRC5 | 16 | 57060347 | T | G | 47 | C/G;498 | missense | SNV | no |
|  | AC | NLRP11 | 19 | 56312983 | A | G | 62 | L/P;709 | missense | SNV | no |
|  | AC | NLRP12 | 19 | 54313381 | C | T | 95 | S/N;511 | missense | SNV | no |
|  | AC | NLRP7 | 19 | 55439093 | G | A | 18 | A/V;954 | missense | SNV | no |
|  | AC | NOP2 | 12 | 6672533 | G | A | 62 | S/F;275 | missense | SNV | no |
|  | AC | NPC1L1 | 7 | 44579596 | T | A | 89 | S/C;134 | missense | SNV | no |
|  | AC | NPY5R | 4 | 164271471 | G | T | 56 | E/*;16 | nonsense | SNV | no |
|  | AC | NR2E3 | 15 | 72104149 | C | A | 66 | R/S;97 | missense | SNV | no |
|  | AC | NRG1 | 8 | 32616936 | C | T | 29 | T/I;353 | missense | SNV | no |
|  | AC | NRXN3 | 14 | 79454374 | C | A | 83 | T/N;678 | missense | SNV | no |
|  | AC | NTRK3 | 15 | 88670418 | T | C | 21 | H/R;423 | missense | SNV | no |
|  | AC | ODF1 | 8 | 103573011 | * | -TGCAACCCCTGCAGCCCCTGCAACCCG | 55 | CODON_DELETION;218 | frameshift | DEL | no |
|  | AC | ODZ2 | 5 | 167653121 | G | A | 90 | V/M;1704 | missense | SNV | no |
|  | AC | OGDHL | 10 | 50953460 | T | C | 42 | Q/R;520 | missense | SNV | no |
|  | AC | OLFM3 | 1 | 102290718 | C | A | 37 | Q/H;152 | missense | SNV | no |
|  | AC | OSBPL10 | 3 | 31725474 | C | T | 51 | E/K;460 | missense | SNV | no |
|  | AC | OTOP3 | 17 | 72937599 | G | A | 15 | R/Q;62 | missense | SNV | no |
|  | AC | OXSM | 3 | 25832687 | C | A | 52 | T/N;59 | missense | SNV | no |
|  | AC | PABPC4 | 1 | 40041563 | C | A | 47 | D/Y;21 | missense | SNV | no |
|  | AC | PAH | 12 | 103306610 | C | G | 31 | E/Q;43 | missense | SNV | no |
|  | AC | PARVG | 22 | 44577642 | G | T | 91 | ; | silent | SNV | no |
|  | AC | PDIA4 | 7 | 148702331 | T | A | 16 | N/I;475 | missense | SNV | no |
|  | AC | PDS5A | 4 | 39850578 | C | T | 49 | V/I;1078 | missense | SNV | no |
|  | AC | PDZRN4 | 12 | 41967687 | G | A | 37 | V/I;1036 | missense | SNV | no |
|  | AC | PEG10 | 7 | 94293534 | G | T | 29 | E/D;256 | missense | SNV | no |
|  | AC | PGBD3 | 10 | 50725041 | C | G | 44 | E/D;40 | missense | SNV | no |
|  | AC | PGLYRP4 | 1 | 153317828 | G | C | 29 | S/C;57 | missense | SNV | no |
|  | AC | PGM5 | 9 | 71114216 | C | T | 42 | T/I;518 | missense | SNV | no |
|  | AC | PHOSPHO2 | 2 | 170558140 | A | G | 17 | Y/C;220 | missense | SNV | no |
|  | AC | PIF1 | 15 | 65113397 | T | A | 36 | K/M;352 | missense | SNV | no |
|  | AC | PIP4K2B | 17 | 36940591 | C | T | 33 | E/K;87 | missense | SNV | no |
|  | AC | PKD1L3 | 16 | 72001871 | G | T | 43 | S/R;740 | missense | SNV | no |
|  | AC | PKNOX2 | 11 | 125298926 | G | T | 35 | D/Y;319 | missense | SNV | no |
|  | AC | PKP1 | 1 | 201252943 | G | A | 38 | R/K;38 | missense | SNV | no |
|  | AC | PLCD4 | 2 | 219486293 | G | T | 40 | D/Y;171 | missense | SNV | no |
|  | AC | PLEKHG7 | 12 | 93150103 | G | A | 62 | M/I;212 | missense | SNV | no |
|  | AC | PLXNA4 | 7 | 131815306 | G | T | 32 | Q/K;1873 | missense | SNV | no |
|  | AC | PNP | 14 | 20943050 | A | G | 81 | H/R;135 | missense | SNV | no |
|  | AC | POLL | 10 | 103339531 | C | A | 47 | Q/H;469 | missense | SNV | yes |
|  | AC | POLQ | 3 | 121207510 | * | -AATAGTA | 62 | FRAME_SHIFT;1421 | frameshift | DEL | yes |
|  | AC | PPP2R3A | 3 | 135768192 | C | T | 36 | P/S;820 | missense | SNV | no |
|  | AC | PPP3CA | 4 | 101984420 | * | +A | 48 | FRAME_SHIFT;350 | frameshift | INS | no |
|  | AC | PPP6R3 | 11 | 68363657 | A | G | 34 | E/G;670 | missense | SNV | no |
|  | AC | PREX2 | 8 | 68864694 | G | C | 12 | R/P;22 | missense | SNV | yes |
|  | AC | PRG4 | 1 | 186277738 | * | -ACC | 60 | CODON_DELETION;963 | frameshift | DEL | no |
|  | AC | PRKAR1B | 7 | 720261 | G | A | 95 | R/C;94 | missense | SNV | no |
|  | AC | PRKCZ | 1 | 2075712 | A | G | 65 | I/V;162 | missense | SNV | no |
|  | AC | PRKD2 | 19 | 47219561 | G | A | 94 | P/S;23 | missense | SNV | no |
|  | AC | PRKDC | 8 | 48701540 | T | A | 15 | M/L;3610 | missense | SNV | yes |
|  | AC | PROKR2 | 20 | 5294930 | C | G | 27 | S/T;29 | missense | SNV | no |
|  | AC | PRR23C | 3 | 138762927 | T | C | 56 | Y/C;179 | missense | SNV | no |
|  | AC | PTGER3 | 1 | 71418670 | C | T | 32 | V/M;393 | missense | SNV | no |
|  | AC | PTTG2 | 4 | 37962233 | A | G | 62 | T/A;60 | missense | SNV | no |
|  | AC | PWWP2A | 5 | 159520302 | G | C | 91 | A/G;452 | missense | SNV | no |
|  | AC | PYDC1 | 16 | 31228238 | G | T | 49 | R/S;38 | missense | SNV | no |
|  | AC | QSER1 | 11 | 32956552 | * | +T | 40 | FRAME_SHIFT;1121 | frameshift | INS | no |
|  | AC | RAB21 | 12 | 72148833 | C | T | 56 | ; | silent | SNV | no |
|  | AC | RAB3GAP2 | 1 | 220383808 | C | A | 54 | Q/H;178 | missense | SNV | no |
|  | AC | RAB3IP | 12 | 70133227 | C | T | 10 | ; | silent | SNV | no |
|  | AC | RAB3IP | 12 | 70188265 | G | T | 37 | E/*;235 | nonsense | SNV | no |
|  | AC | RACGAP1 | 12 | 50393506 | G | C | 36 | S/C;214 | missense | SNV | no |
|  | AC | RAD21L1 | 20 | 1221024 | A | G | 51 | I/V;268 | missense | SNV | no |
|  | AC | RAI1 | 17 | 17697102 | * | -G | 49 | FRAME_SHIFT;280 | frameshift | DEL | yes |
|  | AC | RALGDS | 9 | 135987455 | C | T | 68 | G/S;90 | missense | SNV | no |
|  | AC | RASGRF1 | 15 | 79298742 | T | G | 18 | I/L;634 | missense | SNV | no |
|  | AC | RB1 | 13 | 49027182 | * | -TGATCACCTTGAATCTGCT | 73 | FRAME_SHIFT;583 | frameshift | DEL | yes |
|  | AC | RBBP8 | 18 | 20562237 | A | T | 19 | D/V;162 | missense | SNV | no |
|  | AC | RBL1 | 20 | 35672641 | * | -C | 57 | FRAME_SHIFT;540 | frameshift | DEL | no |
|  | AC | RBM44 | 2 | 238726707 | C | T | 56 | T/I;383 | missense | SNV | no |
|  | AC | RETN | 19 | 7734250 | G | A | 30 | G/E;13 | missense | SNV | no |
|  | AC | RGS12 | 4 | 3418743 | C | T | 24 | S/L;844 | missense | SNV | no |
|  | AC | RGS7 | 1 | 240975240 | C | A | 32 | E/*;354 | nonsense | SNV | no |
|  | AC | RIOK2 | 5 | 96503604 | C | A | 78 | D/Y;322 | missense | SNV | no |
|  | AC | RIPK4 | 21 | 43176968 | A | C | 22 | M/R;64 | missense | SNV | no |
|  | AC | RNF149 | 2 | 101911512 | C | G | 25 | G/R;198 | missense | SNV | no |
|  | AC | ROS1 | 6 | 117707014 | C | A | 15 | W/C;712 | missense | SNV | yes |
|  | AC | RP1L1 | 8 | 10465720 | G | C | 21 | S/C;1963 | missense | SNV | no |
|  | AC | RPGRIP1L | 16 | 53686557 | T | A | 32 | E/V;681 | missense | SNV | no |
|  | AC | RPGRIP1L | 16 | 53690456 | C | T | 47 | D/N;543 | missense | SNV | no |
|  | AC | RSPO4 | 20 | 944591 | C | A | 75 | R/S;194 | missense | SNV | no |
|  | AC | RYR2 | 1 | 237813285 | C | G | 27 | H/D;2541 | missense | SNV | no |
|  | AC | RYR2 | 1 | 237941999 | C | G | 36 | H/D;3937 | missense | SNV | no |
|  | AC | SATB2 | 2 | 200193573 | G | C | 49 | L/V;412 | missense | SNV | no |
|  | AC | SCAP | 3 | 47456124 | A | T | 43 | S/T;1109 | missense | SNV | no |
|  | AC | SAMD9 | 7 | 92731877 | C | G | 57 | K/N;1178 | missense | SNV | yes |
|  | AC | SECISBP2L | 15 | 49293263 | C | T | 61 | D/N;687 | missense | SNV | no |
|  | AC | SEH1L | 18 | 12984108 | C | T | 45 | S/F;330 | missense | SNV | no |
|  | AC | SERPINA12 | 14 | 94982205 | C | T | 96 | ; | silent | SNV | no |
|  | AC | SERPINB7 | 18 | 61471733 | C | A | 46 | A/D;336 | missense | SNV | no |
|  | AC | SERPINE1 | 7 | 100771687 | C | T | 50 | P/S;5 | missense | SNV | no |
|  | AC | SETD1B | 12 | 122247884 | G | A | 61 | A/T;345 | missense | SNV | no |
|  | AC | SHISA9 | 16 | 12996441 | G | C | 29 | G/R;174 | missense | SNV | no |
|  | AC | SHKBP1 | 19 | 41084374 | G | A | 28 | R/H;109 | missense | SNV | no |
|  | AC | SI | 3 | 164735625 | C | A | 14 | R/L;1186 | missense | SNV | no |
|  | AC | SLFN11 | 17 | 33689799 | G | A | 18 | T/I;343 | missense | SNV | no |
|  | AC | SLIT2 | 4 | 20258301 | A | T | 32 | L/F;62 | missense | SNV | no |
|  | AC | DHRSX | X | 2139186 | C | T | 69 | E/K;297 | missense | SNV | yes |
|  | AC | DSCAML1 | 11 | 117306468 | T | C | 11 | K/E;1650 | missense | SNV | yes |
|  | AC | NCAM2 | 21 | 22849772 | C | A | 32 | P/Q;686 | missense | SNV | yes |
|  | AC | SMARCC2 | 12 | 56558477 | T | C | 20 | M/V;1060 | missense | SNV | yes |
|  | AC | SOCS6 | 18 | 67993325 | G | C | 44 | R/T;474 | missense | SNV | no |
|  | AC | SOD1 | 21 | 33032141 | A | G | 58 | N/S;20 | missense | SNV | no |
|  | AC | SOHLH2 | 13 | 36744834 | G | A | 93 | T/I;364 | missense | SNV | no |
|  | AC | SORCS1 | 10 | 108431059 | G | C | 36 | Q/E;709 | missense | SNV | no |
|  | AC | SORCS1 | 10 | 108466383 | C | G | 36 | G/R;385 | missense | SNV | no |
|  | AC | SPARCL1 | 4 | 88415453 | G | C | 27 | P/A;167 | missense | SNV | no |
|  | AC | SPEF2 | 5 | 35692784 | A | T | 54 | E/D;619 | missense | SNV | no |
|  | AC | SPERT | 13 | 46288254 | C | G | 83 | A/G;365 | missense | SNV | no |
|  | AC | PCLO | 7 | 82453602 | G | C | 34 | P/R;4849 | missense | SNV | yes |
|  | AC | PTPRZ1 | 7 | 121698982 | G | A | 57 | M/I;2219 | missense | SNV | yes |
|  | AC | SAMD9 | 7 | 92731876 | C | A | 56 | E/*;1179 | nonsense | SNV | no |
|  | AC | SPICE1 | 3 | 113187011 | C | G | 60 | C/S;377 | missense | SNV | no |
|  | AC | SPRY3 | X | 155003874 | G | T | 24 | R/L;114 | missense | SNV | no |
|  | AC | SPRY3 | X | 155004014 | G | A | 68 | A/T;161 | missense | SNV | no |
|  | AC | SPTBN5 | 15 | 42153646 | C | T | 88 | E/K;2561 | missense | SNV | no |
|  | AC | SRSF3 | 6 | 36564701 | A | T | 94 | E/D;54 | missense | SNV | no |
|  | AC | SPHKAP | 2 | 228881750 | C | G | 42 | V/L;1274 | missense | SNV | yes |
|  | AC | SPHKAP | 2 | 228884528 | T | C | 41 | M/V;348 | missense | SNV | yes |
|  | AC | SSPO | 7 | 149493549 | G | A | 73 | D/N;2209 | missense | SNV | yes |
|  | AC | ST8SIA4 | 5 | 100231418 | G | A | 81 | S/L;62 | missense | SNV | no |
|  | AC | STAG1 | 3 | 136323177 | C | A | 51 | V/L;91 | missense | SNV | no |
|  | AC | STAT2 | 12 | 56745079 | T | A | 23 | H/L;313 | missense | SNV | no |
|  | AC | STAU1 | 20 | 47782626 | G | C | 38 | T/S;38 | missense | SNV | no |
|  | AC | SUSD4 | 1 | 223537474 | G | T | 33 | ; | silent | SNV | no |
|  | AC | SUV420H2 | 19 | 55858566 | C | G | 43 | P/A;380 | missense | SNV | no |
|  | AC | SYT3 | 19 | 51133420 | G | T | 89 | A/D;228 | missense | SNV | no |
|  | AC | TAB2 | 6 | 149699636 | G | T | 85 | L/F;195 | missense | SNV | no |
|  | AC | TAF1L | 9 | 32633773 | G | C | 44 | T/S;602 | missense | SNV | yes |
|  | AC | TAF8 | 6 | 42023338 | C | G | 47 | S/C;91 | missense | SNV | no |
|  | AC | TAL1 | 1 | 47685498 | C | A | 43 | G/V;297 | missense | SNV | no |
|  | AC | TAOK3 | 12 | 118639243 | G | A | 59 | P/L;282 | missense | SNV | no |
|  | AC | TAS2R4 | 7 | 141478473 | T | G | 27 | F/C;62 | missense | SNV | no |
|  | AC | TBC1D10C | 11 | 67177191 | G | A | 69 | R/Q;436 | missense | SNV | no |
|  | AC | TCEA3 | 1 | 23720482 | G | A | 18 | R/C;237 | missense | SNV | no |
|  | AC | TCF7L1 | 2 | 85536331 | C | G | 40 | L/V;505 | missense | SNV | yes |
|  | AC | TCHH | 1 | 152082734 | C | A | 54 | E/*;987 | nonsense | SNV | no |
|  | AC | SSPO | 7 | 149499218 | G | C | 29 | R/P;2529 | missense | SNV | yes |
|  | AC | TDRD7 | 9 | 100245329 | G | T | 38 | G/C;871 | missense | SNV | yes |
|  | AC | TTN | 2 | 179426083 | G | C | 65 | P/R;26618 | missense | SNV | no |
|  | AC | TTN | 2 | 179440867 | G | T | 61 | P/Q;21690 | missense | SNV | no |
|  | AC | TEAD4 | 12 | 3131160 | G | T | 55 | A/S;292 | missense | SNV | no |
|  | AC | TENC1 | 12 | 53454755 | G | T | 29 | G/V;1032 | missense | SNV | no |
|  | AC | TGFBR3 | 1 | 92177930 | G | T | 35 | P/Q;679 | missense | SNV | no |
|  | AC | THAP11 | 16 | 67877101 | C | G | 45 | T/S;215 | missense | SNV | no |
|  | AC | TTN | 2 | 179470426 | G | C | 28 | P/A;16225 | missense | SNV | no |
|  | AC | TTN | 2 | 179408323 | C | T | 60 | W/*;30485 | nonsense | SNV | no |
|  | AC | TIMELESS | 12 | 56827696 | G | C | 25 | L/V;38 | missense | SNV | no |
|  | AC | TMOD1 | 9 | 100286492 | G | C | 37 | E/Q;8 | missense | SNV | no |
|  | AC | TNR | 1 | 175372457 | C | G | 28 | R/S;265 | missense | SNV | no |
|  | AC | SMARCA4 | 19 | 11100016 | G | T | 38 | R/L;381 | missense | SNV | yes |
|  | AC | TP53 | 17 | 7577124 | C | A | 80 | V/L272 | missense | SNV | yes |
|  | AC | TP53TG5 | 20 | 44003902 | G | T | 58 | P/H;182 | missense | SNV | no |
|  | AC | TPR | 1 | 186330793 | C | A | 27 | V/L;307 | missense | SNV | no |
|  | AC | TRAK1 | 3 | 42234633 | G | A | 52 | R/H;279 | missense | SNV | no |
|  | AC | TRDN | 6 | 123892232 | G | T | 85 | S/Y;23 | missense | SNV | no |
|  | AC | TRIM10 | 6 | 30121995 | C | A | 90 | E/D;399 | missense | SNV | no |
|  | AC | TRIM10 | 6 | 30121994 | C | A | 90 | E/*;400 | nonsense | SNV | no |
|  | AC | TRIM59 | 3 | 160155862 | * | -AGAT | 53 | FRAME_SHIFT;369 | frameshift | DEL | no |
|  | AC | TRIM63 | 1 | 26387738 | * | -G | 35 | FRAME_SHIFT;140 | frameshift | DEL | no |
|  | AC | TRIP6 | 7 | 100465514 | * | +C | 45 | FRAME_SHIFT;47 | frameshift | INS | no |
|  | AC | SMARCA4 | 19 | 11100015 | C | T | 93 | R/*;381 | nonsense | SNV | yes |
|  | AC | UBQLN3 | 11 | 5530737 | C | A | 41 | D/Y;18 | missense | SNV | no |
|  | AC | UBQLN3 | 11 | 5530738 | C | A | 41 | Q/H;17 | missense | SNV | no |
|  | AC | UGT8 | 4 | 115544811 | G | T | 10 | V/F;259 | missense | SNV | no |
|  | AC | UNC5CL | 6 | 40999485 | C | A | 88 | D/Y;352 | missense | SNV | no |
|  | AC | UNC79 | 14 | 93994949 | C | A | 86 | P/T;160 | missense | SNV | no |
|  | AC | UNC80 | 2 | 210778594 | G | T | 48 | M/I;1587 | missense | SNV | no |
|  | AC | URB2 | 1 | 229773739 | G | C | 29 | D/H;1127 | missense | SNV | no |
|  | AC | UROC1 | 3 | 126226851 | T | A | 31 | S/C;167 | missense | SNV | no |
|  | AC | UTP15 | 5 | 72864287 | T | A | 89 | F/I;76 | missense | SNV | no |
|  | AC | UTP6 | 17 | 30211489 | G | C | 18 | Q/E;247 | missense | SNV | no |
|  | AC | VCAM1 | 1 | 101198189 | G | T | 22 | G/*;581 | nonsense | SNV | no |
|  | AC | VPS13C | 15 | 62176430 | C | T | 54 | W/*;3127 | nonsense | SNV | no |
|  | AC | VPS54 | 2 | 64189430 | T | A | 32 | K/*;258 | nonsense | SNV | no |
|  | AC | VPS8 | 3 | 184571751 | C | G | 39 | H/D;310 | missense | SNV | no |
|  | AC | VWA5B1 | 1 | 20678634 | C | T | 24 | S/F;1037 | missense | SNV | no |
|  | AC | WDFY4 | 10 | 49951476 | G | A | 45 | R/H;781 | missense | SNV | no |
|  | AC | XYLT1 | 16 | 17353016 | C | T | 77 | E/K;248 | missense | SNV | no |
|  | AC | YAE1D1 | 7 | 39606081 | G | C | 79 | E/Q;22 | missense | SNV | no |
|  | AC | ZAN | 7 | 100345175 | A | C | 36 | S/R;312 | missense | SNV | no |
|  | AC | ZP4 | 1 | 238049079 | G | T | 31 | T/N;316 | missense | SNV | no |
| 450 | AC | APBA3 | 19 | 3754333 | C | A | 10 | G/C;208 | missense | SNV | no |
|  | AC | ARHGAP29 | 1 | 94643186 | A | T | 46 | C/S;963 | missense | SNV | no |
|  | AC | C17orf104 | 17 | 42745515 | A | C | 15 | T/P;746 | missense | SNV | no |
|  | AC | C19orf44 | 19 | 16613885 | C | A | 10 | Q/K;257 | missense | SNV | no |
|  | AC | FBL | 19 | 40329687 | G | T | 11 | D/E;179 | missense | SNV | no |
|  | AC | FOXN1 | 17 | 26851066 | C | A | 12 | L/I;27 | missense | SNV | no |
|  | AC | HDAC10 | 22 | 50685379 | G | A | 10 | A/V;480 | missense | SNV | no |
|  | AC | IGDCC3 | 15 | 65625604 | C | A | 13 | V/L;325 | missense | SNV | no |
|  | AC | ITGAE | 17 | 3651344 | G | T | 11 | S/Y;676 | missense | SNV | no |
|  | AC | KLHL20 | 1 | 173702915 | C | A | 11 | D/E;29 | missense | SNV | no |
|  | AC | KLHL5 | 4 | 39083753 | C | A | 11 | H/N;338 | missense | SNV | no |
|  | AC | L1TD1 | 1 | 62675645 | * | -CCTCAGGGCTGGAGGAGGAGGAGGAAGAGC | 38 | CODON_DELETION;400 | frameshift | DEL | no |
|  | AC | MAPKBP1 | 15 | 42117449 | G | T | 11 | A/S;1454 | missense | SNV | no |
|  | AC | MED12L | 3 | 150873989 | G | T | 11 | A/S;200 | missense | SNV | no |
|  | AC | RNF31 | 14 | 24618781 | C | A | 11 | H/Q;266 | missense | SNV | no |
|  | AC | SETDB2 | 13 | 50051154 | G | T | 13 | C/F;295 | missense | SNV | no |
|  | AC | STARD13 | 13 | 33679749 | C | A | 10 | G/V;1108 | missense | SNV | no |
|  | AC | VAT1 | 17 | 41168565 | C | A | 10 | G/V;286 | missense | SNV | no |
| 122 | LCNEC | APOBEC3H | 22 | 39498005 | T | A | 14 | D/E;167 | missense | SNV | no |
|  | LCNEC | ARHGAP42 | 11 | 100730341 | G | C | 33 | Q/H;123 | missense | SNV | no |
|  | LCNEC | C4orf21 | 4 | 113539261 | T | A | 27 | E/V;646 | missense | SNV | no |
|  | LCNEC | C8orf45 | 8 | 67786786 | C | T | 28 | L/F;84 | missense | SNV | no |
|  | LCNEC | CCL2 | 17 | 32582441 | C | T | 18 | P/S;25 | missense | SNV | no |
|  | LCNEC | CEP120 | 5 | 122720721 | G | T | 29 | R/S;563 | missense | SNV | no |
|  | LCNEC | CHRM2 | 7 | 136553858 | G | T | 31 | ; | silent | SNV | no |
|  | LCNEC | CKMT2 | 5 | 80550232 | T | G | 17 | F/L;123 | missense | SNV | no |
|  | LCNEC | COASY | 17 | 40716056 | C | A | 28 | P/T;289 | missense | SNV | no |
|  | LCNEC | CSMD2 | 1 | 34128631 | G | T | 10 | P/T;1332 | missense | SNV | no |
|  | LCNEC | DMD | X | 32834714 | G | T | 45 | T/N;134 | missense | SNV | no |
|  | LCNEC | ELMO2 | 20 | 45022237 | C | T | 28 | W/*;41 | nonsense | SNV | no |
|  | LCNEC | EXOC4 | 7 | 133041180 | C | G | 22 | A/G;287 | missense | SNV | no |
|  | LCNEC | FNBP4 | 11 | 47754213 | C | A | 10 | A/S;566 | missense | SNV | no |
|  | LCNEC | IL2RB | 22 | 37524815 | T | C | 22 | E/G;326 | missense | SNV | no |
|  | LCNEC | IQGAP2 | 5 | 75902118 | A | T | 20 | E/D;449 | missense | SNV | no |
|  | LCNEC | MEN1 | 11 | 64572605 | G | T | 32 | Y/*;422 | nonsense | SNV | yes |
|  | LCNEC | NOTCH3 | 19 | 15273308 | T | C | 26 | N/D;1961 | missense | SNV | no |
|  | LCNEC | PI4K2A | 10 | 99416587 | T | C | 19 | F/L;260 | missense | SNV | no |
|  | LCNEC | PLEKHG1 | 6 | 151161337 | G | A | 24 | E/K;1155 | missense | SNV | no |
|  | LCNEC | PPP1CA | 11 | 67166584 | G | T | 13 | P/T;203 | missense | SNV | no |
|  | LCNEC | PRPF8 | 17 | 1563731 | A | G | 25 | C/R;1594 | missense | SNV | no |
|  | LCNEC | PTHLH | 12 | 28116617 | A | T | 25 | L/Q;63 | missense | SNV | no |
|  | LCNEC | RBM44 | 2 | 238726634 | C | T | 22 | P/S;359 | missense | SNV | no |
|  | LCNEC | SAPCD1 | 6 | 31731294 | G | A | 28 | A/T;73 | missense | SNV | no |
|  | LCNEC | SGSM1 | 22 | 25294441 | G | T | 10 | S/I;897 | missense | SNV | no |
|  | LCNEC | SH3PXD2B | 5 | 171765672 | C | G | 23 | G/R;813 | missense | SNV | no |
|  | LCNEC | STAB2 | 12 | 104100730 | G | T | 11 | C/F;1386 | missense | SNV | no |
|  | LCNEC | SYT15 | 10 | 46967698 | T | A | 16 | I/F;127 | missense | SNV | no |
|  | LCNEC | TFAP2B | 6 | 50810969 | C | A | 13 | A/D;416 | missense | SNV | no |
|  | LCNEC | TYK2 | 19 | 10475582 | T | C | 32 | H/R;385 | missense | SNV | no |
|  | LCNEC | USP24 | 1 | 55548956 | G | T | 10 | Q/K;2322 | missense | SNV | no |
|  | LCNEC | USP40 | 2 | 234442322 | T | C | 25 | N/S;436 | missense | SNV | no |
| 302 | LCNEC | ABCC2 | 10 | 101606853 | C | T | 24 | H/Y;1428 | missense | SNV | no |
|  | LCNEC | ACAN | 15 | 89383354 | A | T | 31 | Q/L;189 | missense | SNV | no |
|  | LCNEC | ACTR3C | 7 | 149981924 | G | A | 23 | P/L;161 | missense | SNV | no |
|  | LCNEC | ACTR6 | 12 | 100606270 | G | T | 37 | L/F;238 | missense | SNV | no |
|  | LCNEC | ADAM12 | 10 | 127737977 | G | A | 11 | R/W;591 | missense | SNV | no |
|  | LCNEC | ADAM7 | 8 | 24323260 | G | T | 44 | A/S;121 | missense | SNV | no |
|  | LCNEC | ADCY8 | 8 | 131792671 | G | T | 34 | Q/K;1241 | missense | SNV | no |
|  | LCNEC | ADRBK1 | 11 | 67047368 | A | G | 17 | E/G;167 | missense | SNV | no |
|  | LCNEC | ANGPT4 | 20 | 870957 | G | A | 27 | Q/*;122 | nonsense | SNV | no |
|  | LCNEC | ANKRD5 | 20 | 10019203 | C | T | 44 | T/I;85 | missense | SNV | no |
|  | LCNEC | ARHGAP40 | 20 | 37270381 | T | A | 63 | L/Q;430 | missense | SNV | no |
|  | LCNEC | ARID2 | 12 | 46231437 | C | G | 19 | T/R;426 | missense | SNV | yes |
|  | LCNEC | C12orf35 | 12 | 32133898 | G | T | 27 | W/C;3 | missense | SNV | no |
|  | LCNEC | C12orf77 | 12 | 25148936 | G | A | 27 | T/M;71 | missense | SNV | no |
|  | LCNEC | C17orf104 | 17 | 42745515 | A | C | 13 | T/P;746 | missense | SNV | no |
|  | LCNEC | C20orf166 | 20 | 61147941 | C | G | 11 | ; | silent | SNV | no |
|  | LCNEC | CHODL | 21 | 19629116 | G | T | 67 | G/*;124 | nonsense | SNV | no |
|  | LCNEC | CLASP2 | 3 | 33614683 | C | G | 28 | R/T;882 | missense | SNV | no |
|  | LCNEC | COLEC12 | 18 | 346546 | G | A | 15 | T/M;359 | missense | SNV | no |
|  | LCNEC | CSMD2 | 1 | 34102047 | A | T | 23 | Y/N;1588 | missense | SNV | no |
|  | LCNEC | CTC1 | 17 | 8139262 | C | A | 43 | V/L;365 | missense | SNV | no |
|  | LCNEC | CTNNA3 | 10 | 67680290 | G | A | 63 | S/L;829 | missense | SNV | no |
|  | LCNEC | DAXX | 6 | 33288991 | C | A | 30 | Q/H;199 | missense | SNV | yes |
|  | LCNEC | CWH43 | 4 | 49032968 | A | G | 37 | H/R;500 | missense | SNV | no |
|  | LCNEC | CYSLTR1 | X | 77528540 | A | T | 71 | I/N;235 | missense | SNV | no |
|  | LCNEC | DDHD1 | 14 | 53527909 | T | G | 21 | I/L;593 | missense | SNV | no |
|  | LCNEC | DDI1 | 11 | 103908539 | T | C | 49 | L/P;330 | missense | SNV | no |
|  | LCNEC | DIP2B | 12 | 51122340 | T | C | 31 | S/P;1174 | missense | SNV | no |
|  | LCNEC | DLG2 | 11 | 84245693 | C | T | 41 | V/M;147 | missense | SNV | no |
|  | LCNEC | EFCAB6 | 22 | 44027987 | G | A | 47 | R/W;744 | missense | SNV | no |
|  | LCNEC | ESPL1 | 12 | 53680029 | T | G | 11 | V/G;1170 | missense | SNV | no |
|  | LCNEC | FAM160A2 | 11 | 6244468 | C | A | 42 | V/L;260 | missense | SNV | no |
|  | LCNEC | FAM75E1 | 9 | 90503375 | C | T | 50 | Q/*;1325 | nonsense | SNV | no |
|  | LCNEC | FAT2 | 5 | 150922011 | G | A | 64 | Q/*;2893 | nonsense | SNV | no |
|  | LCNEC | FLNC | 7 | 128493829 | G | C | 56 | R/P;2141 | missense | SNV | no |
|  | LCNEC | FRAS1 | 4 | 79372970 | T | G | 36 | F/V;2170 | missense | SNV | no |
|  | LCNEC | FUT7 | 9 | 139925847 | G | T | 58 | P/H;115 | missense | SNV | no |
|  | LCNEC | FZD1 | 7 | 90895866 | C | G | 40 | Y/*;557 | nonsense | SNV | no |
|  | LCNEC | GPR110 | 6 | 46979899 | C | A | 33 | E/D;320 | missense | SNV | no |
|  | LCNEC | GPR78 | 4 | 8588920 | A | G | 30 | M/V;308 | missense | SNV | no |
|  | LCNEC | GRIN2A | 16 | 10273871 | A | G | 33 | M/T;133 | missense | SNV | yes |
|  | LCNEC | HELLS | 10 | 96342780 | A | T | 14 | K/N;430 | missense | SNV | no |
|  | LCNEC | HERPUD2 | 7 | 35673313 | T | A | 18 | Q/L;403 | missense | SNV | no |
|  | LCNEC | HOXD10 | 2 | 176983745 | G | A | 82 | R/K;270 | missense | SNV | no |
|  | LCNEC | IQSEC1 | 3 | 12977231 | T | A | 52 | R/W;429 | missense | SNV | no |
|  | LCNEC | KIF20A | 5 | 137518955 | C | A | 23 | N/K;310 | missense | SNV | no |
|  | LCNEC | KIF21A | 12 | 39734802 | C | A | 48 | Q/H;672 | missense | SNV | no |
|  | LCNEC | KLF15 | 3 | 126071384 | C | A | 56 | G/C;128 | missense | SNV | no |
|  | LCNEC | PTPRZ1 | 7 | 121652018 | A | C | 16 | K/T;973 | missense | SNV | yes |
|  | LCNEC | RIN3 | 14 | 93119291 | C | T | 20 | R/C;633 | missense | SNV | yes |
|  | LCNEC | MAGEC2 | X | 141290821 | * | -A | 42 | FRAME_SHIFT;318 | frameshift | DEL | no |
|  | LCNEC | MAGIX | X | 49022686 | G | A | 46 | R/Q;318 | missense | SNV | no |
|  | LCNEC | MARCH9 | 12 | 58152397 | A | T | 41 | Q/L;253 | missense | SNV | no |
|  | LCNEC | MED12 | X | 70341593 | C | T | 92 | S/L;343 | missense | SNV | no |
|  | LCNEC | MLL2 | 12 | 49443797 | C | A | 45 | V/L;1192 | missense | SNV | yes |
|  | LCNEC | MLL3 | 7 | 151949158 | C | T | 25 | C/Y;496 | missense | SNV | yes |
|  | LCNEC | MSH4 | 1 | 76344798 | * | -T | 54 | FRAME_SHIFT;554 | frameshift | DEL | yes |
|  | LCNEC | MTMR11 | 1 | 149902417 | T | C | 17 | Y/C;496 | missense | SNV | no |
|  | LCNEC | THSD7B | 2 | 137814405 | C | A | 45 | F/L;154 | missense | SNV | yes |
|  | LCNEC | NCOA6 | 20 | 33337453 | G | T | 19 | H/N;849 | missense | SNV | no |
|  | LCNEC | NFATC2 | 20 | 50140520 | C | A | 28 | G/V;87 | missense | SNV | no |
|  | LCNEC | NID1 | 1 | 236189353 | C | A | 52 | W/C;609 | missense | SNV | no |
|  | LCNEC | NRXN3 | 14 | 79423641 | A | G | 53 | T/A;405 | missense | SNV | no |
|  | LCNEC | OAS3 | 12 | 113385830 | G | T | 57 | E/*;319 | nonsense | SNV | no |
|  | LCNEC | PBXIP1 | 1 | 154924274 | C | G | 14 | E/Q;59 | missense | SNV | no |
|  | LCNEC | PCBP3 | 21 | 47316206 | G | A | 67 | R/K;32 | missense | SNV | no |
|  | LCNEC | PLCB1 | 20 | 8609033 | C | A | 36 | N/K;113 | missense | SNV | no |
|  | LCNEC | RABEP1 | 17 | 5235256 | G | T | 72 | R/M;59 | missense | SNV | no |
|  | LCNEC | RAPGEF2 | 4 | 160189162 | A | T | 50 | ; | silent | SNV | no |
|  | LCNEC | RETNLB | 3 | 108475382 | G | T | 59 | Q/K;61 | missense | SNV | no |
|  | LCNEC | RG9MTD1 | 3 | 101284687 | T | A | 44 | D/E;354 | missense | SNV | no |
|  | LCNEC | RGS12 | 4 | 3319503 | C | T | 24 | R/C;536 | missense | SNV | no |
|  | LCNEC | RNF213 | 17 | 78318790 | G | A | 16 | A/T;2219 | missense | SNV | no |
|  | LCNEC | RPN2 | 20 | 35857146 | T | C | 21 | V/A;498 | missense | SNV | no |
|  | LCNEC | RUNX1T1 | 8 | 93003925 | G | C | 52 | Y/*;370 | nonsense | SNV | no |
|  | LCNEC | SEPP1 | 5 | 42804849 | C | A | 25 | G/V;178 | missense | SNV | no |
|  | LCNEC | SERPINA9 | 14 | 94933654 | G | T | 28 | Q/K;250 | missense | SNV | no |
|  | LCNEC | SIM1 | 6 | 100841407 | T | A | 10 | Y/F;509 | missense | SNV | no |
|  | LCNEC | SLMO2 | 20 | 57610147 | A | T | 41 | L/*;167 | nonsense | SNV | no |
|  | LCNEC | SMC2 | 9 | 106875640 | A | C | 63 | K/T;433 | missense | SNV | no |
|  | LCNEC | STRADB | 2 | 202344238 | A | T | 16 | H/L;366 | missense | SNV | no |
|  | LCNEC | TCF4 | 18 | 52896102 | G | T | 70 | L/I;721 | missense | SNV | no |
|  | LCNEC | TCF4 | 18 | 52927185 | A | T | 13 | L/H;457 | missense | SNV | no |
|  | LCNEC | TDRD5 | 1 | 179659996 | C | T | 21 | A/V;1009 | missense | SNV | no |
|  | LCNEC | SMARCA4 | 19 | 11134266 | C | T | 79 | R/*;978 | nonsense | SNV | yes |
|  | LCNEC | TP53 | 17 | 7577046 | G | T | 61 | R/*;298 | nonsense | SNV | yes |
|  | LCNEC | TRIML2 | 4 | 189013065 | T | A | 29 | H/L;209 | missense | SNV | no |
|  | LCNEC | UFSP1 | 7 | 100486771 | A | T | 22 | V/E;41 | missense | SNV | no |
|  | LCNEC | UGT3A2 | 5 | 36051981 | A | G | 17 | L/S;101 | missense | SNV | no |
|  | LCNEC | URB1 | 21 | 33717058 | C | T | 21 | V/M;1360 | missense | SNV | no |
|  | LCNEC | VCPIP1 | 8 | 67577367 | C | A | 23 | W/C;609 | missense | SNV | no |
| 509 | LCNEC | ABCB1 | 7 | 87165851 | T | A | 66 | S/C;802 | missense | SNV | no |
|  | LCNEC | ACOX3 | 4 | 8394175 | C | A | 24 | E/D;395 | missense | SNV | no |
|  | LCNEC | ADAMTSL1 | 9 | 18889686 | C | A | 46 | A/E;1528 | missense | SNV | no |
|  | LCNEC | ALMS1 | 2 | 73680043 | A | T | 28 | Q/L;2129 | missense | SNV | no |
|  | LCNEC | ANK2 | 4 | 114279934 | G | T | 79 | R/I;3387 | missense | SNV | no |
|  | LCNEC | AP3B1 | 5 | 77512059 | C | A | 73 | L/F;202 | missense | SNV | no |
|  | LCNEC | ARHGAP11A | 15 | 32928862 | A | G | 56 | T/A;630 | missense | SNV | no |
|  | LCNEC | BARX1 | 9 | 96715182 | C | A | 48 | D/Y;171 | missense | SNV | no |
|  | LCNEC | BMPER | 7 | 34118714 | G | A | 67 | G/S;442 | missense | SNV | no |
|  | LCNEC | C1orf201 | 1 | 24706252 | G | A | 47 | S/L;118 | missense | SNV | no |
|  | LCNEC | C7orf63 | 7 | 89887418 | T | A | 66 | L/M;63 | missense | SNV | no |
|  | LCNEC | CARD6 | 5 | 40852581 | A | G | 28 | M/V;383 | missense | SNV | no |
|  | LCNEC | CD28 | 2 | 204599536 | C | A | 10 | H/Q;188 | missense | SNV | no |
|  | LCNEC | CD44 | 11 | 35223223 | G | T | 10 | D/Y;348 | missense | SNV | no |
|  | LCNEC | CDH10 | 5 | 24492944 | T | G | 52 | T/P;536 | missense | SNV | no |
|  | LCNEC | CPA3 | 3 | 148614426 | C | A | 33 | P/T;396 | missense | SNV | no |
|  | LCNEC | CSNK1A1 | 5 | 148892718 | G | A | 50 | Q/*;199 | nonsense | SNV | no |
|  | LCNEC | EPHA5 | 4 | 66230783 | G | C | 80 | Q/E;730 | missense | SNV | yes |
|  | LCNEC | EPHA6 | 3 | 96962944 | T | A | 25 | C/*;473 | nonsense | SNV | yes |
|  | LCNEC | ERF | 19 | 42754508 | A | T | 14 | Y/N;78 | missense | SNV | no |
|  | LCNEC | ETV3 | 1 | 157108132 | G | T | 12 | ; | silent | SNV | no |
|  | LCNEC | FAM124B | 2 | 225244749 | G | T | 22 | S/R;303 | missense | SNV | no |
|  | LCNEC | FMN2 | 1 | 240371776 | G | T | 35 | A/S;1222 | missense | SNV | no |
|  | LCNEC | FN1 | 2 | 216289994 | T | G | 33 | T/P;287 | missense | SNV | no |
|  | LCNEC | FOXN4 | 12 | 109719389 | G | T | 54 | P/T;373 | missense | SNV | no |
|  | LCNEC | FUCA1 | 1 | 24189750 | T | C | 61 | Y/C;179 | missense | SNV | no |
|  | LCNEC | GABRA2 | 4 | 46252585 | C | T | 51 | A/T;366 | missense | SNV | no |
|  | LCNEC | H6PD | 1 | 9324397 | C | A | 33 | H/Q;615 | missense | SNV | no |
|  | LCNEC | HDAC4 | 2 | 240029774 | T | C | 47 | Q/R;690 | missense | SNV | no |
|  | LCNEC | HEATR8 | 1 | 55118616 | G | T | 30 | G/V;6 | missense | SNV | no |
|  | LCNEC | HMCN1 | 1 | 186024722 | C | A | 23 | H/N;2354 | missense | SNV | no |
|  | LCNEC | HTR5A | 7 | 154875912 | C | A | 69 | H/Q;263 | missense | SNV | no |
|  | LCNEC | IFRD1 | 7 | 112112854 | G | T | 16 | G/*;402 | nonsense | SNV | no |
|  | LCNEC | KIAA1524 | 3 | 108300964 | A | T | 56 | I/K;148 | missense | SNV | no |
|  | LCNEC | L1CAM | X | 153135018 | G | C | 96 | N/K;408 | missense | SNV | no |
|  | LCNEC | LDB2 | 4 | 16504439 | C | G | 67 | E/Q;317 | missense | SNV | no |
|  | LCNEC | LOXL2 | 8 | 23191114 | G | A | 10 | Q/*;256 | nonsense | SNV | no |
|  | LCNEC | LRP1B | 2 | 141079598 | C | A | 43 | A/S;4192 | missense | SNV | yes |
|  | LCNEC | MIER1 | 1 | 67411940 | G | A | 25 | E/K;101 | missense | SNV | no |
|  | LCNEC | MOS | 8 | 57026175 | C | T | 25 | A/T;123 | missense | SNV | no |
|  | LCNEC | MOXD1 | 6 | 132636865 | G | C | 84 | P/A;473 | missense | SNV | no |
|  | LCNEC | NCOA6 | 20 | 33328785 | G | A | 38 | P/S;1759 | missense | SNV | no |
|  | LCNEC | NEDD9 | 6 | 11213865 | G | T | 11 | N/K;36 | missense | SNV | no |
|  | LCNEC | NLGN4X | X | 5810905 | G | T | 84 | Q/K;802 | missense | SNV | no |
|  | LCNEC | NOTCH2 | 1 | 120462024 | C | A | 47 | D/Y;1898 | missense | SNV | yes |
|  | LCNEC | NUDT12 | 5 | 102890488 | C | G | 65 | R/T;344 | missense | SNV | no |
|  | LCNEC | NUP43 | 6 | 150067518 | G | T | 15 | D/E;38 | missense | SNV | no |
|  | LCNEC | ORAI2 | 7 | 102079483 | G | A | 63 | R/Q;27 | missense | SNV | no |
|  | LCNEC | PAPPA2 | 1 | 176564365 | T | A | 39 | V/E;542 | missense | SNV | yes |
|  | LCNEC | PRTG | 15 | 55964657 | G | T | 40 | T/N;676 | missense | SNV | no |
|  | LCNEC | PSMB9 | 6 | 32825108 | C | T | 71 | S/F;66 | missense | SNV | no |
|  | LCNEC | RIMS1 | 6 | 73110376 | C | G | 87 | S/C;1680 | missense | SNV | no |
|  | LCNEC | RNASE3 | 14 | 21360133 | C | A | 46 | N/K;96 | missense | SNV | no |
|  | LCNEC | ROR1 | 1 | 64608253 | C | A | 25 | P/Q;365 | missense | SNV | no |
|  | LCNEC | SBK2 | 19 | 56047562 | G | T | 17 | Q/K;34 | missense | SNV | no |
|  | LCNEC | SI | 3 | 164781249 | T | G | 19 | L/F;296 | missense | SNV | no |
|  | LCNEC | SIGLEC7 | 19 | 51656413 | C | A | 69 | P/T;439 | missense | SNV | no |
|  | LCNEC | SIPA1L3 | 19 | 38652940 | G | T | 25 | G/*;1237 | nonsense | SNV | no |
|  | LCNEC | SOX4 | 6 | 21595920 | * | -CTC | 49 | CODON_DELETION;385 | frameshift | DEL | no |
|  | LCNEC | SDHA | 5 | 218480 | G | T | 12 | V/F;4 | missense | SNV | yes |
|  | LCNEC | SPTA1 | 1 | 158650476 | C | A | 31 | R/L;192 | missense | SNV | no |
|  | LCNEC | SRCRB4D | 7 | 76019524 | T | C | 62 | H/R;527 | missense | SNV | no |
|  | LCNEC | TBP | 6 | 170871014 | * | +CAG | 39 | CODON_INSERTION;64 | frameshift | INS | no |
|  | LCNEC | TNFRSF8 | 1 | 12157274 | G | A | 25 | D/N;90 | missense | SNV | no |
|  | LCNEC | TP53 | 17 | 7577529 | A | T | 74 | I/N251 | missense | SNV | yes |
|  | LCNEC | TRIM24 | 7 | 138268730 | G | T | 72 | A/S;977 | missense | SNV | no |
|  | LCNEC | RIN3 | 14 | 93118129 | C | A | 10 | S/R;245 | missense | SNV | yes |
|  | LCNEC | THSD7B | 2 | 138434094 | C | A | 10 | A/E;1551 | missense | SNV | yes |
|  | LCNEC | TTN | 2 | 179639818 | C | A | 51 | W/L;2207 | missense | SNV | no |
|  | LCNEC | UBR5 | 8 | 103293746 | G | A | 18 | R/C;1900 | missense | SNV | yes |
|  | LCNEC | UGT3A1 | 5 | 35955956 | G | T | 46 | S/R;362 | missense | SNV | no |
|  | LCNEC | UNC80 | 2 | 210650809 | G | T | 35 | R/L;207 | missense | SNV | no |
|  | LCNEC | WNT11 | 11 | 75905637 | G | C | 65 | L/V;191 | missense | SNV | no |
| 080 | SCLC | ACTRT1 | X | 127185960 | G | C | 100 | R/G;76 | missense | SNV | no |
|  | SCLC | ADAMTS17 | 15 | 100801725 | C | A | 10 | K/N;330 | missense | SNV | yes |
|  | SCLC | ADAMTS20 | 12 | 43771233 | G | T | 34 | Q/K;1644 | missense | SNV | yes |
|  | SCLC | ADCY1 | 7 | 45753581 | G | T | 64 | G/V;1116 | missense | SNV | no |
|  | SCLC | ADGB | 6 | 146985374 | C | A | 24 | P/H;217 | missense | SNV | no |
|  | SCLC | ALG10 | 12 | 34175297 | C | T | 44 | ; | silent | SNV | no |
|  | SCLC | ALLC | 2 | 3743885 | A | G | 56 | M/V;230 | missense | SNV | no |
|  | SCLC | ANO5 | 11 | 22249050 | C | A | 39 | A/E;189 | missense | SNV | no |
|  | SCLC | AP5B1 | 11 | 65545695 | G | T | 12 | P/T;757 | missense | SNV | no |
|  | SCLC | APOC4 | 19 | 45445539 | C | T | 37 | S/F;2 | missense | SNV | no |
|  | SCLC | ART4 | 12 | 14993650 | A | T | 21 | F/L;194 | missense | SNV | no |
|  | SCLC | BAI3 | 6 | 70071061 | C | A | 28 | P/H;1299 | missense | SNV | yes |
|  | SCLC | BRDT | 1 | 92446448 | A | T | 27 | Q/L;492 | missense | SNV | no |
|  | SCLC | C11orf41 | 11 | 33682463 | C | A | 32 | T/N;1724 | missense | SNV | no |
|  | SCLC | C12orf51 | 12 | 112696382 | T | G | 25 | T/P;872 | missense | SNV | no |
|  | SCLC | CHST5 | 16 | 75563503 | G | T | 96 | H/Q;260 | missense | SNV | no |
|  | SCLC | CLLU1 | 12 | 92818494 | G | T | 52 | R/I;13 | missense | SNV | no |
|  | SCLC | CLLU1 | 12 | 92818493 | A | T | 50 | R/*;13 | nonsense | SNV | no |
|  | SCLC | NCAM2 | 21 | 22849645 | G | C | 97 | V/L;644 | missense | SNV | yes |
|  | SCLC | DACT1 | 14 | 59112441 | C | A | 52 | T/K;367 | missense | SNV | no |
|  | SCLC | DAGLB | 7 | 6485734 | A | C | 23 | W/G;33 | missense | SNV | no |
|  | SCLC | KAT6A | 8 | 41798422 | C | A | 32 | E/*;993 | nonsense | SNV | yes |
|  | SCLC | DDX60L | 4 | 169343034 | C | A | 49 | K/N;757 | missense | SNV | no |
|  | SCLC | PCLO | 7 | 82585626 | C | A | 26 | G/V;1548 | missense | SNV | yes |
|  | SCLC | PTPRZ1 | 7 | 121650412 | G | C | 11 | E/Q;438 | missense | SNV | yes |
|  | SCLC | SPHKAP | 2 | 228881950 | C | T | 48 | R/K;1207 | missense | SNV | yes |
|  | SCLC | DIABLO | 12 | 122711931 | C | T | 22 | ; | silent | SNV | no |
|  | SCLC | DNMT1 | 19 | 10257186 | C | A | 13 | C/F;912 | missense | SNV | no |
|  | SCLC | DROSHA | 5 | 31515286 | C | A | 62 | E/*;367 | nonsense | SNV | no |
|  | SCLC | TDRD7 | 9 | 100222478 | G | T | 12 | G/C;292 | missense | SNV | yes |
|  | SCLC | TTN | 2 | 179479241 | C | T | 50 | V/M;14693 | missense | SNV | no |
|  | SCLC | EIF2B2 | 14 | 75470095 | G | T | 11 | G/V;94 | missense | SNV | no |
|  | SCLC | EP400 | 12 | 132512737 | G | T | 22 | G/V;1762 | missense | SNV | yes |
|  | SCLC | EPHA3 | 3 | 89259506 | C | A | 77 | P/H;217 | missense | SNV | yes |
|  | SCLC | FAM176A | 2 | 75720723 | C | A | 38 | R/L;33 | missense | SNV | no |
|  | SCLC | FAM210A | 18 | 13681896 | C | A | 12 | A/S;61 | missense | SNV | no |
|  | SCLC | FAM46C | 1 | 118165695 | G | A | 38 | V/I;69 | missense | SNV | no |
|  | SCLC | FAM49B | 8 | 130874549 | T | A | 37 | Q/L;76 | missense | SNV | no |
|  | SCLC | FIG4 | 6 | 110064438 | G | T | 34 | Q/H;334 | missense | SNV | no |
|  | SCLC | GAB4 | 22 | 17468869 | G | T | 11 | Q/K;223 | missense | SNV | no |
|  | SCLC | GALNTL6 | 4 | 172735773 | G | C | 28 | L/F;14 | missense | SNV | no |
|  | SCLC | GIMAP6 | 7 | 150325428 | C | T | 30 | W/*;156 | nonsense | SNV | no |
|  | SCLC | GPR19 | 12 | 12814997 | C | G | 27 | G/A;129 | missense | SNV | no |
|  | SCLC | GREB1 | 2 | 11758537 | C | A | 11 | P/H;1179 | missense | SNV | no |
|  | SCLC | GRIA2 | 4 | 158257722 | G | T | 70 | G/V;556 | missense | SNV | no |
|  | SCLC | GRIK3 | 1 | 37324761 | T | C | 33 | H/R;351 | missense | SNV | no |
|  | SCLC | HELQ | 4 | 84337960 | G | C | 38 | A/G;1041 | missense | SNV | no |
|  | SCLC | HERC6 | 4 | 89318016 | G | T | 10 | A/S;301 | missense | SNV | no |
|  | SCLC | HHLA1 | 8 | 133112322 | C | A | 47 | E/*;67 | nonsense | SNV | no |
|  | SCLC | HIST2H2AC | 1 | 149858661 | C | T | 10 | A/V;46 | missense | SNV | no |
|  | SCLC | HOXD4 | 2 | 177017566 | T | C | 42 | S/P;222 | missense | SNV | no |
|  | SCLC | IFI44L | 1 | 79106800 | A | T | 33 | Q/H;381 | missense | SNV | no |
|  | SCLC | LRP1B | 2 | 141707848 | C | A | 50 | C/F;1031 | missense | SNV | yes |
|  | SCLC | KIAA1731 | 11 | 93430374 | C | T | 15 | Q/*;766 | nonsense | SNV | no |
|  | SCLC | KIAA2026 | 9 | 5921398 | G | A | 89 | P/L;1533 | missense | SNV | no |
|  | SCLC | KIF17 | 1 | 21012574 | C | T | 58 | E/K;662 | missense | SNV | no |
|  | SCLC | KIF6 | 6 | 39545914 | C | T | 38 | E/K;367 | missense | SNV | no |
|  | SCLC | LENG8 | 19 | 54966687 | G | T | 37 | Q/H;322 | missense | SNV | no |
|  | SCLC | LOC100130452 | 2 | 197577454 | C | G | 43 | ; | silent | SNV | no |
|  | SCLC | LRBA | 4 | 151789345 | T | A | 64 | R/S;854 | missense | SNV | no |
|  | SCLC | LSR | 19 | 35749961 | G | A | 26 | V/I;238 | missense | SNV | no |
|  | SCLC | LZTR1 | 22 | 21346513 | C | A | 12 | A/D;335 | missense | SNV | no |
|  | SCLC | MKRN1 | 7 | 140158895 | C | A | 29 | C/F;228 | missense | SNV | no |
|  | SCLC | MTOR | 1 | 11205060 | C | A | 45 | A/S;1577 | missense | SNV | yes |
|  | SCLC | MTOR | 1 | 11205065 | A | C | 41 | L/*;1575 | nonsense | SNV | yes |
|  | SCLC | NBAS | 2 | 15614317 | C | A | 48 | Q/H;491 | missense | SNV | no |
|  | SCLC | ODZ3 | 4 | 183659609 | G | T | 32 | R/S;1097 | missense | SNV | no |
|  | SCLC | PAK4 | 19 | 39668326 | G | C | 62 | W/C;499 | missense | SNV | no |
|  | SCLC | PAQR3 | 4 | 79851461 | C | A | 50 | V/L;123 | missense | SNV | no |
|  | SCLC | PCMTD2 | 20 | 62899333 | G | A | 93 | E/K;226 | missense | SNV | no |
|  | SCLC | PDK3 | X | 24512955 | C | G | 43 | P/R;68 | missense | SNV | no |
|  | SCLC | PHYHD1 | 9 | 131703749 | A | T | 86 | K/M;236 | missense | SNV | no |
|  | SCLC | PKHD1L1 | 8 | 110471901 | C | A | 45 | P/Q;2361 | missense | SNV | no |
|  | SCLC | PKIB | 6 | 123039044 | T | G | 69 | S/R;35 | missense | SNV | no |
|  | SCLC | PMS1 | 2 | 190728731 | A | T | 35 | K/*;707 | nonsense | SNV | yes |
|  | SCLC | PLEKHG6 | 12 | 6427911 | C | A | 11 | L/M;426 | missense | SNV | no |
|  | SCLC | PLEKHH3 | 17 | 40821492 | C | A | 11 | A/S;721 | missense | SNV | no |
|  | SCLC | PLSCR5 | 3 | 146323233 | G | A | 50 | ; | silent | SNV | no |
|  | SCLC | PMPCB | 7 | 102937909 | * | -G | 79 | FRAME_SHIFT;1 | frameshift | DEL | no |
|  | SCLC | PPHLN1 | 12 | 42729717 | G | T | 53 | G/*;5 | nonsense | SNV | no |
|  | SCLC | PRMT2 | 21 | 48069519 | G | T | 12 | Q/H;174 | missense | SNV | no |
|  | SCLC | PTPN13 | 4 | 87637686 | C | G | 50 | P/R;400 | missense | SNV | no |
|  | SCLC | PTPRB | 12 | 70988472 | T | A | 35 | T/S;431 | missense | SNV | no |
|  | SCLC | PTPRQ | 12 | 81072478 | A | G | 71 | K/R;2107 | missense | SNV | no |
|  | SCLC | RANBP3L | 5 | 36251501 | C | T | 46 | S/N;448 | missense | SNV | no |
|  | SCLC | RIMS1 | 6 | 72952017 | G | T | 11 | G/V;653 | missense | SNV | no |
|  | SCLC | SGSM1 | 22 | 25243657 | C | T | 58 | R/C;66 | missense | SNV | no |
|  | SCLC | SMCHD1 | 18 | 2694567 | C | G | 60 | L/V;306 | missense | SNV | no |
|  | SCLC | SPTBN1 | 2 | 54876194 | G | A | 38 | R/K;1690 | missense | SNV | no |
|  | SCLC | SYNM | 15 | 99653868 | C | G | 53 | I/M;294 | missense | SNV | no |
|  | SCLC | TENC1 | 12 | 53455048 | G | T | 12 | E/*;1130 | nonsense | SNV | no |
|  | SCLC | THUMPD2 | 2 | 39982471 | C | T | 41 | E/K;348 | missense | SNV | no |
|  | SCLC | TOP2B | 3 | 25654167 | C | A | 10 | A/S;1204 | missense | SNV | no |
|  | SCLC | TRIM66 | 11 | 8642729 | G | A | 47 | T/I;956 | missense | SNV | no |
|  | SCLC | TRIM69 | 15 | 45047237 | G | C | 50 | R/P;49 | missense | SNV | no |
|  | SCLC | TYR | 11 | 88911563 | G | T | 54 | D/Y;148 | missense | SNV | no |
|  | SCLC | USH1C | 11 | 17552986 | G | T | 10 | H/N;70 | missense | SNV | no |
|  | SCLC | USP37 | 2 | 219394700 | G | C | 40 | S/C;281 | missense | SNV | no |
|  | SCLC | USP9Y | Y | 14838644 | G | A | 39 | R/H;192 | missense | SNV | no |
|  | SCLC | WWP1 | 8 | 87414300 | C | G | 38 | L/V;198 | missense | SNV | no |
|  | SCLC | YES1 | 18 | 724454 | C | A | 19 | E/D;534 | missense | SNV | no |
|  | SCLC | YLPM1 | 14 | 75248841 | A | C | 43 | N/H;699 | missense | SNV | no |
| 348 | SCLC | ADCY8 | 8 | 131812794 | C | A | 68 | A/S;980 | missense | SNV | no |
|  | SCLC | BAG4 | 8 | 38066754 | C | A | 13 | S/*;286 | nonsense | SNV | no |
|  | SCLC | CD163L1 | 12 | 7531612 | G | A | 45 | A/V;778 | missense | SNV | no |
|  | SCLC | CTNND2 | 5 | 11346613 | G | A | 23 | A/V;500 | missense | SNV | no |
|  | SCLC | DCST2 | 1 | 154991127 | G | T | 50 | P/T;739 | missense | SNV | no |
|  | SCLC | GABRR3 | 3 | 97705738 | G | A | 53 | S/F;398 | missense | SNV | no |
|  | SCLC | GPR133 | 12 | 131471868 | * | +T | 44 | FRAME_SHIFT;240 | frameshift | INS | no |
|  | SCLC | GPR133 | 12 | 131471871 | * | +CT | 43 | FRAME_SHIFT;241 | frameshift | INS | no |
|  | SCLC | HDAC7 | 12 | 48190019 | G | A | 47 | H/Y;256 | missense | SNV | no |
|  | SCLC | HIST1H4L | 6 | 27841088 | G | C | 35 | I/M;67 | missense | SNV | no |
|  | SCLC | IL16 | 15 | 81598775 | G | T | 11 | V/F;1232 | missense | SNV | no |
|  | SCLC | MEFV | 16 | 3294267 | C | A | 62 | M/I;582 | missense | SNV | no |
|  | SCLC | MGAT2 | 14 | 50088203 | G | T | 15 | A/S;73 | missense | SNV | no |
|  | SCLC | MGAT4C | 12 | 86383267 | G | T | 60 | L/M;20 | missense | SNV | no |
|  | SCLC | MGAT4C | 12 | 86383268 | G | T | 61 | C/*;19 | nonsense | SNV | no |
|  | SCLC | NAB2 | 12 | 57485536 | G | A | 10 | E/K;238 | missense | SNV | no |
|  | SCLC | PGM1 | 1 | 64100680 | A | G | 57 | D/G;306 | missense | SNV | no |
|  | SCLC | PIKFYVE | 2 | 209212723 | G | T | 53 | E/*;1784 | nonsense | SNV | no |
|  | SCLC | PLEKHB1 | 11 | 73357660 | C | A | 51 | P/T;3 | missense | SNV | no |
|  | SCLC | PLEKHB1 | 11 | 73357661 | C | G | 52 | P/R;3 | missense | SNV | no |
|  | SCLC | RB1 | 13 | 48954353 | G | T | 48 | E/*;492 | nonsense | SNV | yes |
|  | SCLC | RAB31 | 18 | 9859236 | C | A | 35 | P/T;168 | missense | SNV | no |
|  | SCLC | RAB3GAP2 | 1 | 220327342 | T | C | 42 | M/V;1205 | missense | SNV | no |
|  | SCLC | CSMD3 | 8 | 113504743 | C | A | 54 | W/C;1751 | missense | SNV | yes |
|  | SCLC | RTN2 | 19 | 45996454 | G | A | 54 | P/S;333 | missense | SNV | no |
|  | SCLC | RYR2 | 1 | 237936868 | G | A | 43 | D/N;3899 | missense | SNV | no |
|  | SCLC | SH2B1 | 16 | 28884996 | C | A | 13 | A/D;709 | missense | SNV | no |
|  | SCLC | TCF7 | 5 | 133451702 | A | C | 21 | H/P;140 | missense | SNV | yes |
|  | SCLC | TP53 | 17 | 7578524 | G | C | 16 | Q/E;136 | missense | SNV | yes |
|  | SCLC | VWA1 | 1 | 1372421 | C | T | 18 | P/L;63 | missense | SNV | no |
| 477 | SCLC | ABCC5 | 3 | 183707072 | G | A | 33 | P/S;77 | missense | SNV | no |
|  | SCLC | ADAMTS5 | 21 | 28337912 | G | T | 10 | R/S;267 | missense | SNV | yes |
|  | SCLC | ARID2 | 12 | 46233150 | T | C | 51 | F/L;457 | missense | SNV | yes |
|  | SCLC | ARL14 | 3 | 160395349 | G | C | 34 | R/T;72 | missense | SNV | no |
|  | SCLC | ARL4D | 17 | 41477618 | G | T | 43 | G/V;173 | missense | SNV | no |
|  | SCLC | ARSH | X | 2928153 | C | G | 54 | R/G;59 | missense | SNV | no |
|  | SCLC | ASXL3 | 18 | 31326090 | G | T | 46 | S/I;2093 | missense | SNV | no |
|  | SCLC | ATP10A | 15 | 25925364 | G | A | 48 | P/L;1257 | missense | SNV | no |
|  | SCLC | ATP10A | 15 | 25966830 | C | G | 46 | G/A;446 | missense | SNV | no |
|  | SCLC | BCAP31 | X | 152969451 | G | T | 23 | A/D;214 | missense | SNV | no |
|  | SCLC | BCLAF1 | 6 | 136599800 | G | T | 13 | Y/*;73 | nonsense | SNV | no |
|  | SCLC | BCORL1 | X | 129149045 | G | T | 34 | C/F;766 | missense | SNV | no |
|  | SCLC | BRIP1 | 17 | 59761457 | C | T | 39 | V/M;984 | missense | SNV | yes |
|  | SCLC | C16orf96 | 16 | 4624769 | G | A | 45 | M/I;195 | missense | SNV | no |
|  | SCLC | C19orf6 | 19 | 1011102 | A | T | 52 | F/Y;437 | missense | SNV | no |
|  | SCLC | C1GALT1C1 | X | 119760268 | C | T | 46 | G/R;252 | missense | SNV | no |
|  | SCLC | C1orf51 | 1 | 150255924 | C | T | 44 | R/W;83 | missense | SNV | no |
|  | SCLC | C2orf71 | 2 | 29295455 | G | A | 42 | P/L;558 | missense | SNV | no |
|  | SCLC | C4orf29 | 4 | 128938520 | G | T | 35 | R/M;158 | missense | SNV | no |
|  | SCLC | CASP1 | 11 | 104905009 | T | A | 52 | Q/L;67 | missense | SNV | no |
|  | SCLC | CASP1 | 11 | 104905010 | G | T | 52 | Q/K;67 | missense | SNV | no |
|  | SCLC | CD244 | 1 | 160803846 | T | A | 36 | R/W;324 | missense | SNV | no |
|  | SCLC | CDKL2 | 4 | 76539602 | A | G | 41 | L/S;67 | missense | SNV | no |
|  | SCLC | CELSR2 | 1 | 109816148 | G | T | 54 | G/V;2867 | missense | SNV | no |
|  | SCLC | CHRDL1 | X | 109964741 | C | A | 38 | V/L;107 | missense | SNV | no |
|  | SCLC | CITED1 | X | 71521702 | G | T | 41 | D/E;177 | missense | SNV | no |
|  | SCLC | COLEC11 | 2 | 3691604 | G | A | 51 | D/N;252 | missense | SNV | no |
|  | SCLC | CPO | 2 | 207833979 | G | T | 58 | G/V;315 | missense | SNV | no |
|  | SCLC | CPT2 | 1 | 53666464 | C | G | 46 | Q/E;76 | missense | SNV | no |
|  | SCLC | CRHR1 | 17 | 43898762 | G | T | 45 | A/S;95 | missense | SNV | no |
|  | SCLC | CUX2 | 12 | 111733207 | C | G | 53 | H/D;211 | missense | SNV | no |
|  | SCLC | CYBB | X | 37663143 | C | A | 10 | P/H;304 | missense | SNV | no |
|  | SCLC | CYP2C8 | 10 | 96827345 | C | A | 33 | G/V;91 | missense | SNV | no |
|  | SCLC | DBC1 | 9 | 121929805 | C | A | 60 | V/F;615 | missense | SNV | no |
|  | SCLC | DCHS1 | 11 | 6653750 | G | C | 37 | P/R;998 | missense | SNV | no |
|  | SCLC | DDX20 | 1 | 112298540 | G | T | 47 | ; | silent | SNV | no |
|  | SCLC | DNM3 | 1 | 171956945 | G | T | 50 | V/L;129 | missense | SNV | no |
|  | SCLC | DOCK11 | X | 117809950 | G | T | 32 | E/*;1751 | nonsense | SNV | no |
|  | SCLC | DPP6 | 7 | 154237658 | C | A | 41 | L/M;167 | missense | SNV | no |
|  | SCLC | E2F3 | 6 | 20402737 | G | A | 74 | E/K;92 | missense | SNV | no |
|  | SCLC | EDNRB | 13 | 78492698 | G | T | 87 | P/H;94 | missense | SNV | no |
|  | SCLC | EFHC2 | X | 44171999 | C | G | 46 | G/R;16 | missense | SNV | no |
|  | SCLC | ERCC6L | X | 71424959 | G | T | 52 | L/I;1220 | missense | SNV | no |
|  | SCLC | FAM58A | X | 152858018 | C | G | 24 | G/R;201 | missense | SNV | no |
|  | SCLC | FAM5C | 1 | 190068134 | G | T | 45 | L/M;439 | missense | SNV | no |
|  | SCLC | FAM71B | 5 | 156593066 | G | C | 35 | Y/*;38 | nonsense | SNV | no |
|  | SCLC | FBN3 | 19 | 8173074 | C | T | 42 | R/H;1556 | missense | SNV | no |
|  | SCLC | FCGBP | 19 | 40362795 | C | G | 42 | R/P;5092 | missense | SNV | no |
|  | SCLC | FIG4 | 6 | 110048410 | A | G | 50 | T/A;130 | missense | SNV | no |
|  | SCLC | FMN2 | 1 | 240371798 | G | T | 48 | G/V;1229 | missense | SNV | no |
|  | SCLC | FMN2 | 1 | 240374431 | C | A | 39 | P/T;1321 | missense | SNV | no |
|  | SCLC | FOLR4 | 11 | 94040756 | C | A | 37 | N/K;217 | missense | SNV | no |
|  | SCLC | FOXA2 | 20 | 22563521 | C | A | 47 | G/V;120 | missense | SNV | no |
|  | SCLC | FSHR | 2 | 49196000 | G | T | 56 | H/N;231 | missense | SNV | no |
|  | SCLC | GFRAL | 6 | 55216111 | G | C | 42 | C/S;144 | missense | SNV | no |
|  | SCLC | GLRA4 | X | 102983482 | G | T | 40 | ; | silent | SNV | no |
|  | SCLC | GPR26 | 10 | 125447458 | T | A | 45 | F/I;266 | missense | SNV | no |
|  | SCLC | GPR50 | X | 150349298 | T | C | 61 | S/P;415 | missense | SNV | no |
|  | SCLC | HBD | 11 | 5254238 | C | A | 45 | V/L;134 | missense | SNV | no |
|  | SCLC | HCFC1 | X | 153220753 | C | A | 34 | V/F;1033 | missense | SNV | no |
|  | SCLC | HDX | X | 83723912 | G | T | 49 | Y/*;273 | nonsense | SNV | no |
|  | SCLC | HEPH | X | 65409689 | C | A | 47 | N/K;378 | missense | SNV | no |
|  | SCLC | HIST1H2AA | 6 | 25726545 | A | G | 61 | S/P;71 | missense | SNV | no |
|  | SCLC | IGSF11 | 3 | 118645070 | C | A | 61 | G/V;153 | missense | SNV | no |
|  | SCLC | IL16 | 15 | 81552158 | G | T | 49 | A/S;120 | missense | SNV | no |
|  | SCLC | IL36RN | 2 | 113820124 | C | A | 52 | S/*;113 | nonsense | SNV | no |
|  | SCLC | ING1 | 13 | 111367828 | A | C | 44 | E/A;13 | missense | SNV | no |
|  | SCLC | IPO7 | 11 | 9442013 | A | T | 38 | K/M;261 | missense | SNV | no |
|  | SCLC | ISL1 | 5 | 50687161 | C | G | 48 | H/Q;273 | missense | SNV | no |
|  | SCLC | KHDRBS2 | 6 | 62611221 | C | A | 39 | G/V;180 | missense | SNV | no |
|  | SCLC | KHDRBS2 | 6 | 62611222 | C | A | 39 | G/C;180 | missense | SNV | no |
|  | SCLC | KIAA1107 | 1 | 92648092 | G | A | 43 | G/S;1180 | missense | SNV | no |
|  | SCLC | KIAA1217 | 10 | 24810850 | G | T | 40 | M/I;816 | missense | SNV | no |
|  | SCLC | KIF7 | 15 | 90191947 | G | C | 68 | P/A;328 | missense | SNV | no |
|  | SCLC | LPPR4 | 1 | 99767368 | G | T | 44 | G/V;294 | missense | SNV | no |
|  | SCLC | MACF1 | 1 | 39916881 | A | G | 37 | K/E;4747 | missense | SNV | no |
|  | SCLC | MAGEL2 | 15 | 23890650 | T | A | 47 | K/I;747 | missense | SNV | no |
|  | SCLC | MBOAT1 | 6 | 20152909 | C | T | 64 | R/Q;64 | missense | SNV | no |
|  | SCLC | MIOS | 7 | 7634723 | A | G | 49 | H/R;719 | missense | SNV | no |
|  | SCLC | MLL2 | 12 | 49445217 | G | A | 44 | P/L;750 | missense | SNV | yes |
|  | SCLC | MRGPRX4 | 11 | 18194890 | C | A | 49 | S/R;29 | missense | SNV | no |
|  | SCLC | MYSM1 | 1 | 59147672 | T | A | 46 | K/N;348 | missense | SNV | no |
|  | SCLC | NADKD1 | 5 | 36219775 | C | G | 56 | E/D;189 | missense | SNV | no |
|  | SCLC | NAV3 | 12 | 78531026 | C | A | 38 | A/D;1504 | missense | SNV | no |
|  | SCLC | NDST4 | 4 | 115751025 | C | A | 43 | W/L;807 | missense | SNV | no |
|  | SCLC | NDUFB11 | X | 47003949 | C | A | 50 | A/S;44 | missense | SNV | no |
|  | SCLC | NEB | 2 | 152466369 | T | A | 62 | Q/L;4095 | missense | SNV | no |
|  | SCLC | NEUROD4 | 12 | 55421026 | A | G | 45 | D/G;268 | missense | SNV | no |
|  | SCLC | NLRP12 | 19 | 54313971 | C | T | 53 | W/*;314 | nonsense | SNV | no |
|  | SCLC | NOL6 | 9 | 33472211 | C | T | 47 | R/H;85 | missense | SNV | no |
|  | SCLC | NPY2R | 4 | 156135215 | G | A | 50 | D/N;42 | missense | SNV | no |
|  | SCLC | NRG3 | 10 | 84738854 | C | A | 41 | Q/K;521 | missense | SNV | no |
|  | SCLC | NRP2 | 2 | 206588568 | A | C | 26 | T/P;242 | missense | SNV | no |
|  | SCLC | NTNG1 | 1 | 107691331 | C | A | 49 | T/K;39 | missense | SNV | no |
|  | SCLC | NUP205 | 7 | 135322683 | A | G | 41 | Q/R;1714 | missense | SNV | no |
|  | SCLC | OBSCN | 1 | 228528905 | G | A | 43 | R/Q;5936 | missense | SNV | no |
|  | SCLC | P2RY10 | X | 78216532 | G | T | 46 | S/I;172 | missense | SNV | no |
|  | SCLC | PALB2 | 16 | 23640978 | T | A | 56 | K/*;833 | nonsense | SNV | yes |
|  | SCLC | PCGF1 | 2 | 74733163 | C | A | 44 | G/V;149 | missense | SNV | no |
|  | SCLC | PIK3C2A | 11 | 17153571 | T | C | 52 | Y/C;708 | missense | SNV | no |
|  | SCLC | PCNT | 21 | 47786749 | G | T | 49 | A/S;954 | missense | SNV | no |
|  | SCLC | PDHA1 | X | 19375824 | G | T | 46 | D/Y;334 | missense | SNV | no |
|  | SCLC | PDZD4 | X | 153069682 | C | A | 38 | S/I;479 | missense | SNV | no |
|  | SCLC | PHKA1 | X | 71800935 | A | C | 21 | F/V;1197 | missense | SNV | no |
|  | SCLC | PIK3CA | 3 | 178916930 | G | T | 38 | G/V;106 | missense | SNV | yes |
|  | SCLC | PKD1L3 | 16 | 71981447 | A | G | 50 | M/T;1221 | missense | SNV | no |
|  | SCLC | PLCE1 | 10 | 95790933 | G | T | 50 | V/F;44 | missense | SNV | no |
|  | SCLC | PNN | 14 | 39650931 | G | A | 26 | R/K;673 | missense | SNV | no |
|  | SCLC | POLA1 | X | 24839658 | A | T | 55 | R/S;1167 | missense | SNV | no |
|  | SCLC | PRR12 | 19 | 50097764 | G | T | 52 | D/Y;85 | missense | SNV | no |
|  | SCLC | RAD21L1 | 20 | 1223442 | C | T | 50 | L/F;346 | missense | SNV | no |
|  | SCLC | RBMXL2 | 11 | 7111484 | G | A | 51 | R/H;378 | missense | SNV | no |
|  | SCLC | RBP1 | 3 | 139257700 | T | A | 30 | R/W;121 | missense | SNV | no |
|  | SCLC | RBPJL | 20 | 43945220 | G | A | 50 | R/K;427 | missense | SNV | no |
|  | SCLC | RHOXF1 | X | 119243172 | C | G | 41 | C/S;178 | missense | SNV | no |
|  | SCLC | RNF20 | 9 | 104317120 | A | C | 50 | K/Q;722 | missense | SNV | no |
|  | SCLC | RYR1 | 19 | 38976781 | C | T | 66 | P/L;1829 | missense | SNV | no |
|  | SCLC | RB1 | 13 | 48934179 | C | G | 85 | L/V;212 | missense | SNV | yes |
|  | SCLC | RB1 | 13 | 48934197 | T | G | 83 | L/V;218 | missense | SNV | yes |
|  | SCLC | SCG2 | 2 | 224463858 | T | A | 50 | Q/L;48 | missense | SNV | no |
|  | SCLC | SIGLEC11 | 19 | 50461704 | C | A | 40 | G/V;496 | missense | SNV | no |
|  | SCLC | SMC4 | 3 | 160122217 | G | C | 22 | K/N;204 | missense | SNV | no |
|  | SCLC | SPATA2L | 16 | 89764122 | C | G | 55 | G/R;299 | missense | SNV | no |
|  | SCLC | SPEG | 2 | 220334090 | G | T | 61 | R/L;1235 | missense | SNV | no |
|  | SCLC | STAG1 | 3 | 136139936 | T | A | 64 | N/Y;703 | missense | SNV | no |
|  | SCLC | STAG3 | 7 | 99796169 | G | C | 42 | G/A;439 | missense | SNV | no |
|  | SCLC | STK38 | 6 | 36475378 | C | G | 31 | G/A;224 | missense | SNV | no |
|  | SCLC | STXBP5L | 3 | 121037345 | C | A | 33 | P/T;736 | missense | SNV | no |
|  | SCLC | SYTL5 | X | 37948749 | C | T | 48 | T/I;247 | missense | SNV | no |
|  | SCLC | TAF1A | 1 | 222743915 | G | C | 45 | P/A;233 | missense | SNV | no |
|  | SCLC | TAT | 16 | 71609873 | T | C | 45 | K/E;98 | missense | SNV | no |
|  | SCLC | TBP | 6 | 170871014 | * | +CAG | 61 | CODON_INSERTION;64 | frameshift | INS | no |
|  | SCLC | TCEAL5 | X | 102529477 | G | T | 36 | Y/*;5 | nonsense | SNV | no |
|  | SCLC | THEG | 19 | 375733 | G | A | 49 | P/S;80 | missense | SNV | no |
|  | SCLC | TLR8 | X | 12938871 | C | G | 48 | A/G;571 | missense | SNV | no |
|  | SCLC | TNIP3 | 4 | 122068205 | T | A | 63 | Q/L;322 | missense | SNV | no |
|  | SCLC | TOPORS | 9 | 32542047 | G | C | 51 | Q/E;826 | missense | SNV | no |
|  | SCLC | TRIM48 | 11 | 55032437 | A | T | 44 | N/Y;36 | missense | SNV | no |
|  | SCLC | CSMD3 | 8 | 114186070 | C | A | 48 | G/V;197 | missense | SNV | yes |
|  | SCLC | PTPRZ1 | 7 | 121653629 | G | T | 39 | G/V;1510 | missense | SNV | yes |
|  | SCLC | RIN3 | 14 | 93043712 | T | A | 22 | L/Q;86 | missense | SNV | yes |
|  | SCLC | THSD7B | 2 | 138373780 | C | A | 44 | A/D;1124 | missense | SNV | yes |
|  | SCLC | TTN | 2 | 179429432 | A | G | 54 | Y/H;25502 | missense | SNV | no |
|  | SCLC | TTN | 2 | 179452017 | C | A | 46 | E/D;19666 | missense | SNV | no |
|  | SCLC | TP53 | 17 | 7577551 | C | A | 93 | G/C;244 | missense | SNV | yes |
|  | SCLC | UBQLNL | 11 | 5536576 | T | C | 43 | K/E;366 | missense | SNV | no |
|  | SCLC | USH2A | 1 | 216348707 | C | A | 40 | R/I;1505 | missense | SNV | no |
|  | SCLC | VPS25 | 17 | 40925519 | G | A | 59 | W/*;9 | nonsense | SNV | no |
|  | SCLC | VWA3A | 16 | 22108969 | G | A | 49 | D/N;60 | missense | SNV | no |
|  | SCLC | WAPAL | 10 | 88227230 | T | C | 52 | I/V;726 | missense | SNV | no |
|  | SCLC | XRCC6 | 22 | 42057384 | G | C | 38 | E/D;524 | missense | SNV | no |

**Note:** TC, typical carcinoid; AC, atypical carcinoid; LCNEC, large-cell neuroendocrine carcinoma; SCLC, small-cell lung carcinoma. SNV, single nucleotide variant; DEL, deletion; INS, insertion.
