# Supplementary material for: Lung neuroendocrine tumours: deep sequencing of the four World Health Organization histotypes reveals chromatin‐remodelling genes as major players and a prognostic role for TERT, RB1, MEN1 and KMT2D
Source: J Pathol. 2016 Dec 29;241(4):488–500. doi: 10.1002/path.4853 (PMC5324596; doi:10.1002/path.4853)
Supplement: Supplementary file 9 — Table S4A. Discovery screen, whole exome sequencing: list of mutations found in 20 lung neuroendocrine tumours. Related to Supplementary Figure S1A. Table S4B. Discovery screen, high coverage targeted sequencing of 418 genes: list of mutations found in 46 lung neuroendocrine tumours. Related to Supplementary Figure S1B. Table S4C. Discovery screen, integration of whole exome sequencing and high coverage targeted sequencing of 418 genes: list of 36 genes mutated in at least two cases of 46 lung neuroendocrine tumours. Related to Supplementary Figures S1A and S1B. [file PATH-241-488-s010.zip › PATH_4853_TableS4B.docx]

**Supplementary Table S4B**. Discovery screen, high coverage targeted sequencing of 418 genes: list of mutations found in 46 lung neuroendocrine tumours. Related to Supplementary Figure S2B.

| **ID** | **Histotype** | **Gene** | **Chr** | **Pos** | **Ref** | **Alt** | **Var.Freq** | **Effect** | **effect type** | **change type** | **Reference** |
| --- | --- | --- | --- | --- | --- | --- | --- | --- | --- | --- | --- |
| **017** | TC |  |  |  |  |  |  |  |  |  |  |
| **018** | TC | JAK2 | chr9 | 5081775 | GCCCT | GCCTA | 20 | L830* | nonsense | SNV | NM_004972.3 |
|  | TC | TRRAP | chr7 | 98586542 | G | A | 40 | A3186T | missense | SNV | NM_001244580.1 |
| **021** | AC | SAMD9 | chr7 | 92732184 | T | C | 31 | H1076R | missense | SNV | NM_001193307.1 |
|  | AC | WRN | chr8 | 30969135 | C | T | 28 | P698L | missense | SNV | NM_000553.4 |
| **034** | AC | SMARCA4 | chr19 | 11132519 | C | T | 28 | T912I | missense | SNV | NM_001128849.1 |
| **057** | TC | CREB1 | chr2 | 208442316 | C | T | 18 | A273V | missense | SNV | NM_134442.3 |
| **080** | SCLC | ADAMTS20 | chr12 | 43771233 | G | T | 38 | Q1644K | missense | SNV | NM_025003.3 |
|  | SCLC | BAI3 | chr6 | 70071061 | C | A | 33 | P1299H | missense | SNV | NM_001704.2 |
|  | SCLC | DCC | chr18 | 51013217 | G | T | 84 | G1263* | nonsense | SNV | NM_005215.3 |
|  | SCLC | EP400 | chr12 | 132512737 | G | T | 39 | G1762V | missense | SNV | NM_015409.4 |
|  | SCLC | IL7R | chr5 | 35874559 | G | C | 51 | D239H | missense | SNV | NM_002185.3 |
|  | SCLC | KAT6A | chr8 | 41798422 | C | A | 36 | E993* | nonsense | SNV | NM_001099413.1 |
|  | SCLC | LRP1B | chr2 | 141707848 | C | A | 45 | C1031F | missense | SNV | NM_018557.2 |
|  | SCLC | LTK | chr15 | 41797725 | C | A | 47 | K567N | missense | SNV | NM_002344.5 |
|  | SCLC | MAGEA1 | chrX | 152482917 | T | A | 99 | T32S | missense | SNV | NM_004988.4 |
|  | SCLC | MTOR | chr1 | 11205060 | C | A | 64 | A1577S | missense | SNV | NM_004958.3 |
|  | SCLC | MTOR | chr1 | 11205065 | A | C | 63 | L1575* | nonsense | SNV | NM_004958.3 |
|  | SCLC | PIK3CG | chr7 | 106509607 | C | T | 55 | P534L | missense | SNV | NM_002649.2 |
|  | SCLC | NCAM2 | chr21 | 22849645 | G | C | 97 | V644L | missense | SNV | XM_005260987.1 |
|  | SCLC | PCLO | chr7 | 82585626 | C | A | 26 | G1548V | missense | SNV | NM_033026.5 |
|  | SCLC | PTPRZ1 | chr7 | 121650412 | G | C | 11 | E438Q | missense | SNV | NM_002851.2 |
|  | SCLC | SPHKAP | chr2 | 228881950 | C | T | 48 | R1207K | missense | SNV | XM_005246867.1 |
|  | SCLC | TDRD7 | chr9 | 100222478 | G | T | 12 | G292C | missense | SNV | NM_014290.2 |
|  | SCLC | SYNE1 | chr6 | 152734543 | C | G | 58 | R2058S | missense | SNV | NM_182961.3 |
|  | SCLC | SYNE1 | chr6 | 152765547 | T | A | 38 | Q1279L | missense | SNV | NM_182961.3 |
|  | SCLC | TAF1 | chrX | 70603851 | A | T | 11 | M683L | missense | SNV | NM_004606.3 |
|  | SCLC | TCF7L2 | chr10 | 114911491 | C | G | 95 | Q337E | missense | SNV | NM_001146274.1 |
|  | SCLC | TP53 | chr17 | 7577120 | C | A | 93 | R273L | missense | SNV | NM_000546.5 |
| **122** | LCNEC | LRP1B | chr2 | 141707975 | G | A | 21 |  | splice site alteration | SNV | NM_018557.2 |
|  | LCNEC | MEN1 | chr11 | 64572605 | G | T | 29 | Y422* | nonsense | SNV | NM_130803.2 |
|  | LCNEC | PIK3CA | chr3 | 178952085 | A | G | 23 | H1047R | missense | SNV | NM_006218.2 |
|  | LCNEC | PLEKHG5 | chr1 | 6529188 | C | A | 100 | E800D | missense | SNV | NM_001265592.1 |
| **154** | TC |  |  |  |  |  |  |  |  |  |  |
| **166** | AC |  |  |  |  |  |  |  |  |  |  |
| **205** | SCLC | CSMD3 | chr8 | 113599392 | T | C | 49 | N1263S | missense | SNV | NM_198123.1 |
|  | SCLC | GDNF | chr5 | 37815974 | C | T | 73 | E156K | missense | SNV | NM_001190468.1 |
|  | SCLC | KDR | chr4 | 55979642 | G | A | 35 | H269Y | missense | SNV | NM_002253.2 |
|  | SCLC | MLL2 | chr12 | 49424692 | AGCAAGGC | A | 21 | -4550 | frameshift | DEL | NM_003482.3 |
|  | SCLC | LRP1B | chr2 | 141458082 | C | T | 16 | G2179E | missense | SNV | NM_018557.2 |
|  | SCLC | LRP1B | chr2 | 141806783 | T | C | 39 | N521D | missense | SNV | NM_018557.2 |
|  | SCLC | MTRR | chr5 | 7869253 | G | C | 24 | A3P | missense | SNV | NM_024010.2 |
|  | SCLC | MUTYH | chr1 | 45798475 | T | C | 39 | Y179C | missense | SNV | NM_001128425.1 |
|  | SCLC | NTRK1 | chr1 | 156834576 | C | A | 48 | P115H | missense | SNV | NM_002529.3 |
|  | SCLC | PTPRT | chr20 | 41101007 | C | T | 56 | R450Q | missense | SNV | NM_133170.3 |
|  | SCLC | TNFAIP3 | chr6 | 138200216 | C | T | 61 | A545V | missense | SNV | NM_001270508.1 |
|  | SCLC | TP53 | chr17 | 7578538 | T | A | 81 | N131I | missense | SNV | NM_000546.5 |
|  | SCLC | ZNF521 | chr18 | 22806017 | G | A | 68 | A622V | missense | SNV | NM_015461.2 |
| **209** | TC | DSCAML1 | chr11 | 117651410 | G | T | 10 | S114R | missense | SNV | NM_020693.2 |
| **223** | LCNEC | KAT6B | chr10 | 76788807 | G | A | 47 | E1409K | missense | SNV | NM_012330.3 |
|  | LCNEC | MLL2 | chr12 | 49420816 | C | T | 39 | R4978H | missense | SNV | NM_003482.3 |
|  | LCNEC | MET | chr7 | 116411646 | C | T | 51 | S960L | missense | SNV | NM_001127500.1 |
|  | LCNEC | NOTCH2 | chr1 | 120471800 | G | A | 51 | R1231W | missense | SNV | NM_024408.3 |
|  | LCNEC | PDE4DIP | chr1 | 144879054 | T | C | 16 | R1466G | missense | SNV | NM_001198834.3 |
| **243** |  |  |  |  |  |  |  |  |  |  |  |
| **245** |  |  |  |  |  |  |  |  |  |  |  |
| **261** | TC | EPHA3 | chr3 | 89468373 | G | T | 11 | C636F | missense | SNV | NM_005233.5 |
|  | TC | EXT2 | chr11 | 44219469 | G | T | 24 | V499L | missense | SNV | NM_000401.3 |
| **288** | AC | HNF1A | chr12 | 121435427 | G | C | 97 | S487T | missense | SNV | NM_000545.5 |
|  | AC | KDM5C | chrX | 53239900 | T | C | 20 | H514R | missense | SNV | NM_004187.3 |
|  | AC | MEN1 | chr11 | 64575544 | G | T | 95 | A163D | missense | SNV | NM_130803.2 |
|  | AC | NOTCH2 | chr1 | 120548030 | G | C | 55 | R113G | missense | SNV | NM_024408.3 |
|  | AC | PTPRT | chr20 | 41306600 | A | T | 73 | D353E | missense | SNV | NM_133170.3 |
|  | AC | THBS1 | chr15 | 39876248 | C | T | 51 | R255C | missense | SNV | NM_003246.2 |
| **291** | TC |  |  |  |  |  |  |  |  |  |  |
| **302** | LCNEC | ARID2 | chr12 | 46231437 | C | G | 25 | T426R | missense | SNV | NM_152641.2 |
|  | LCNEC | PTPRZ1 | chr7 | 121652018 | A | C | 16 | K973T | missense | SNV | NM_002851.2 |
|  | LCNEC | RIN3 | chr14 | 93119291 | C | T | 20 | R633C | missense | SNV | NM_024832.3 |
|  | LCNEC | THSD7B | chr2 | 137814405 | C | A | 45 | F154L | missense | SNV | XM_005263802.1 |
|  | LCNEC | COL1A1 | chr17 | 48266906 | G | A | 80 |  | splice site alteration | SNV | NM_000088.3 |
|  | LCNEC | DAXX | chr6 | 33288991 | C | A | 20 | Q199H | missense | SNV | NM_001141970.1 |
|  | LCNEC | ERBB4 | chr2 | 212576872 | T | A | 83 | M343L | missense | SNV | NM_005235.2 |
|  | LCNEC | MLL3 | chr7 | 151949158 | C | T | 20 | C496Y | missense | SNV | NM_170606.2 |
|  | LCNEC | MLL2 | chr12 | 49443797 | C | A | 48 | V1192L | missense | SNV | NM_003482.3 |
|  | LCNEC | RNF213 | chr17 | 78318790 | G | A | 21 | A2219T | missense | SNV | NM_001256071.1 |
|  | LCNEC | RUNX1T1 | chr8 | 93003925 | G | C | 51 | Y370* | nonsense | SNV | NM_001198679.1 |
|  | LCNEC | SMARCA4 | chr19 | 11134266 | C | T | 62 | R978* | nonsense | SNV | NM_001128849.1 |
|  | LCNEC | TP53 | chr17 | 7577046 | C | A | 61 | E298* | nonsense | SNV | NM_000546.5 |
| **304** | TC | ATF1 | chr12 | 51189708 | A | G | 41 | E37G | missense | SNV | NM_005171.4 |
|  | TC | BAP1 | chr3 | 52437651 | T | G | 70 | N504H | missense | SNV | NM_004656.2 |
|  | TC | BAP1 | chr3 | 52437866 | G | A | 70 | S432F | missense | SNV | NM_004656.2 |
|  | TC | CIC | chr19 | 42791024 | GAGGCCCAGCA | G | 11 | -57 | frameshift | DEL | NM_015125.3 |
|  | TC | CIC | chr19 | 42794526 | G | A | 11 | A536T | missense | SNV | NM_015125.3 |
|  | TC | CYP2C19 | chr10 | 96602617 | C | T | 27 | R329C | missense | SNV | NM_000769.1 |
|  | TC | DST | chr6 | 56458507 | C | T | 43 | R2108H | missense | SNV | NM_001144769.2 |
|  | TC | NCAM2 | chr21 | 22804428 | A | T | 27 | D494V | missense | SNV | XM_005260987.1 |
|  | TC | PCLO | chr7 | 82545213 | G | C | 10 | S4030C | missense | SNV | NM_033026.5 |
|  | TC | PCLO | chr7 | 82585291 | C | T | 38 | G1660R | missense | SNV | NM_033026.5 |
|  | TC | SPHKAP | chr2 | 228973564 | G | T | 16 | S77Y | missense | SNV | XM_005246867.1 |
|  | TC | TDRD7 | chr9 | 100249540 | A | G | 35 | N1001S | missense | SNV | NM_014290.2 |
|  | TC | FLT1 | chr13 | 28883048 | TGAA | T | 75 | F1217- | deletion | DEL | NM_014290.2 |
|  | TC | FLT3 | chr13 | 28578283 | G | A | 66 | S963L | missense | SNV | NM_014290.2 |
|  | TC | HSP90AA1 | chr14 | 102551747 | G | T | 32 | T306K | missense | SNV | NM_014290.2 |
|  | TC | IRF4 | chr6 | 397138 | G | C | 17 | D175H | missense | SNV | NM_014290.2 |
|  | TC | KAT6B | chr10 | 76603142 | A | T | 45 | Y176F | missense | SNV | NM_014290.2 |
|  | TC | LRP1B | chr2 | 141751640 | G | T | 36 | H856Q | missense | SNV | NM_014290.2 |
|  | TC | NF1 | chr17 | 29676270 | G | A | 46 |  | splice site alteration | SNV | NM_014290.2 |
|  | TC | PIK3R2 | chr19 | 18277076 | G | A | 49 | R508H | missense | SNV | NM_014290.2 |
|  | TC | PKHD1 | chr6 | 51889738 | G | A | 36 | R1624W | missense | SNV | NM_014290.2 |
|  | TC | SAMD9 | chr7 | 92731309 | C | T | 53 | E1368K | missense | SNV | NM_014290.2 |
|  | TC | SGK1 | chr6 | 134493807 | C | T | 14 | V314I | missense | SNV | NM_014290.2 |
|  | TC | SYNE1 | chr6 | 152456274 | C | A | 20 | A8585S | missense | SNV | NM_014290.2 |
|  | TC | TP53 | chr17 | 7577108 | C | A | 43 | C277F | missense | SNV | NM_014290.2 |
|  | TC | XPC | chr3 | 14200110 | C | T | 45 | A425T | missense | SNV | NM_014290.2 |
| **308** | LCNEC | DDIT3 | chr12 | 57910755 | C | G | 51 | G139A | missense | SNV | NM_014290.2 |
|  | LCNEC | EGFR | chr7 | 55240780 | G | A | 51 | R675Q | missense | SNV | NM_014290.2 |
|  | LCNEC | EPHB1 | chr3 | 134670702 | G | T | 42 | V205L | missense | SNV | NM_014290.2 |
|  | LCNEC | FANCD2 | chr3 | 10116239 | C | T | 48 | T914I | missense | SNV | NM_014290.2 |
|  | LCNEC | RNF213 | chr17 | 78350326 | T | G | 60 | S4471A | missense | SNV | NM_014290.2 |
|  | LCNEC | STK11 | chr19 | 1226555 | C | T | 65 | S404F | missense | SNV | NM_014290.2 |
|  | LCNEC | TCF7L1 | chr2 | 85529625 | G | A | 49 | V182I | missense | SNV | NM_014290.2 |
|  | LCNEC | TP53 | chr17 | 7577091 | G | A | 51 | R283C | missense | SNV | NM_014290.2 |
|  | LCNEC | UBR5 | chr8 | 103271302 | G | A | 47 | T2671M | missense | SNV | NM_014290.2 |
| **348** | SCLC | ERCC5 | chr13 | 103498627 | A | G | 64 | Q4R | missense | SNV | NM_014290.2 |
|  | SCLC | PDE4DIP | chr1 | 144856941 | C | T | 21 | G2182S | missense | SNV | NM_014290.2 |
|  | SCLC | RB1 | chr13 | 48954353 | G | T | 50 | E492* | nonsense | SNV | NM_014290.2 |
|  | SCLC | CSMD3 | chr8 | 113504743 | C | A | 54 | W1751C | missense | SNV | NM_014290.2 |
|  | SCLC | TP53 | chr17 | 7578524 | G | C | 16 | Q136E |  | SNV | NM_014290.2 |
| **349** | TC | CDH5 | chr16 | 66420974 | C | T | 10 | A158V | missense | SNV | NM_014290.2 |
|  | TC | EPHB6 | chr7 | 142562451 | G | T | 11 | G298V | missense | SNV | NM_014290.2 |
|  | TC | MLL3 | chr7 | 151848538 | G | C | 12 | L4219V | missense | SNV | NM_014290.2 |
| **351** | TC | CSMD3 | chr8 | 113326713 | C | A | 10 | M2498I | missense | SNV | NM_014290.2 |
|  | TC | DSCAML1 | chr11 | 117651436 | G | T | 10 | P106T | missense | SNV | NM_014290.2 |
| **364** | AC | TDRD7 | chr9 | 100243111 | C | A | 12 | A768D | missense | SNV | NM_014290.2 |
| **384** | TC |  |  |  |  |  |  |  |  |  | NM_014290.2 |
| **389** | AC | ADAMTS20 | chr12 | 43846358 | C | G | 41 | G634A | missense | SNV | NM_014290.2 |
|  | AC | APC | chr5 | 112176209 | C | T | 100 | R1640W | missense | SNV | NM_014290.2 |
|  | AC | DCC | chr18 | 50432530 | C | A | 44 | Q177K | missense | SNV | NM_014290.2 |
|  | AC | RAI1 | chr17 | 17697102 | * | G | 49 | -280 | frameshift | DEL | NM_014290.2 |
|  | AC | DSCAML1 | chr11 | 117306468 | T | C | 11 | K1650E | missense | SNV | NM_014290.2 |
|  | AC | NCAM2 | chr21 | 22849772 | C | A | 32 | P686Q | missense | SNV | NM_014290.2 |
|  | AC | PCLO | chr7 | 82453602 | G | C | 34 | P4849R | missense | SNV | NM_014290.2 |
|  | AC | PTPRZ1 | chr7 | 121698982 | G | A | 57 | M2219I | missense | SNV | NM_014290.2 |
|  | AC | SPHKAP | chr2 | 228881750 | C | G | 42 | V1274L | missense | SNV | NM_014290.2 |
|  | AC | SPHKAP | chr2 | 228884528 | T | C | 41 | M348V | missense | SNV | NM_014290.2 |
|  | AC | TDRD7 | chr9 | 100245329 | G | T | 38 | G871C | missense | SNV | NM_014290.2 |
|  | AC | EPHB1 | chr3 | 134670293 | G | C | 35 | Q68H | missense | SNV | NM_004441.4 |
|  | AC | KDR | chr4 | 55963874 | C | G | 25 | D857H | missense | SNV | NM_002253.2 |
|  | AC | NTRK3 | chr15 | 88670418 | T | C | 23 | H423R | missense | SNV | NM_001012338.2 |
|  | AC | NUP98 | chr11 | 3726529 | G | A | 48 | R995C | missense | SNV | NM_016320.4 |
|  | AC | PDGFRA | chr4 | 55139799 | G | T | 44 | R487L | missense | SNV | NM_006206.4 |
|  | AC | PMS2 | chr7 | 6037009 | C | G | 77 | V251L | missense | SNV | NM_000535.5 |
|  | AC | RALGDS | chr9 | 135987455 | C | T | 59 | G90S | missense | SNV | NM_006266.3 |
|  | AC | SMARCA4 | chr19 | 11100015 | C | T | 94 | R381* | nonsense | SNV | NM_001128849.1 |
|  | AC | TCF7L1 | chr2 | 85536331 | C | G | 50 | L505V | missense | SNV | NM_031283.2 |
|  | AC | TP53 | chr17 | 7577124 | C | A | 80 | V272L | missense | SNV | NM_000546.5 |
|  | AC | TPR | chr1 | 186330793 | C | A | 25 | V307L | missense | SNV | NM_003292.2 |
|  | AC | MLL3 | chr7 | 151947998 | C | G | 21 | E559Q | missense | SNV | NM_170606.2 |
| **393** | TC |  |  |  |  |  |  |  |  |  |  |
| **394** | AC | FANCA | chr16 | 89816249 | G | A | 47 | S1043F | missense | SNV | NM_000135.2 |
|  | AC | FLT4 | chr5 | 180030313 | C | G | 62 | R1324P | missense | SNV | NM_182925.4 |
|  | AC | MLL | chr11 | 118344600 | G | A | 100 | G909N | missense | SNV | NM_001197104.1 |
|  | AC | PDGFRA | chr4 | 55133534 | G | A | 47 | A280T | missense | SNV | NM_006206.4 |
|  | AC | PIK3C2B | chr1 | 204438018 | G | A | 37 | R305C | missense | SNV | NM_002646.3 |
|  | AC | RNF213 | chr17 | 78346427 | G | A | 55 | R4215Q | missense | SNV | NM_001256071.1 |
| **400** | AC | ARID2 | chr12 | 46246321 | A | G | 45 | H1472R | missense | SNV | NM_152641.2 |
|  | AC | ATRX | chrX | 76778767 | C | A | 11 | R2271I | missense | SNV | NM_000489.3 |
|  | AC | MDM4 | chr1 | 204495492 | G | A | 38 | R28Q | missense | SNV | NM_002393.4 |
|  | AC | NOTCH2 | chr1 | 120469232 | G | A | 26 | R1299W | missense | SNV | NM_024408.3 |
|  | AC | RNASEL | chr1 | 182544648 | T | C | 72 | Y702C | missense | SNV | NM_021133.3 |
| **402** | TC |  |  |  |  |  |  |  |  |  |  |
| **419** | TC | LPP | chr3 | 188426172 | G | A | 11 | E411K | missense | SNV | NM_005578.3 |
| **436** | AC |  |  |  |  |  |  |  |  |  |  |
| **450** | AC |  |  |  |  |  |  |  |  |  |  |
| **467** | TC | ARID1A | chr1 | 27057979 | C | T | 43 | Q563* | nonsense | SNV | NM_006015.4 |
|  | TC | BIRC2 | chr11 | 102248419 | C | G | 46 | A520G | missense | SNV | NM_001256163.1 |
| **477** | SCLC | AKT3 | chr1 | 243809341 | T | A | 50 |  | splice site alteration | SNV | NM_005465.4 |
|  | SCLC | ARID2 | chr12 | 46233150 | T | C | 45 | F457L | missense | SNV | NM_152641.2 |
|  | SCLC | BRIP1 | chr17 | 59761457 | C | T | 42 | V984M | missense | SNV | NM_032043.2 |
|  | SCLC | CSMD3 | chr8 | 113275869 | T | A | 43 | L3287F | missense | SNV | NM_198123.1 |
|  | SCLC | CSMD3 | chr8 | 114186070 | C | A | 44 | G197V | missense | SNV | NM_198123.1 |
|  | SCLC | PTPRZ1 | chr7 | 121653629 | G | T | 39 | G1510V | missense | SNV | NM_002851.2 |
|  | SCLC | RIN3 | chr14 | 93043712 | T | A | 22 | L86Q | missense | SNV | NM_024832.3 |
|  | SCLC | THSD7B | chr2 | 138373780 | C | A | 44 | A1124D | missense | SNV | XM_005263802.1 |
|  | SCLC | DST | chr6 | 56418053 | C | G | 45 | W3060C | missense | SNV | NM_001144769.2 |
|  | SCLC | FLT4 | chr5 | 180057722 | G | A | 45 | T78M | missense | SNV | NM_182925.4 |
|  | SCLC | MLL2 | chr12 | 49445217 | G | A | 51 | P750L | missense | SNV | NM_003482.3 |
|  | SCLC | PALB2 | chr16 | 23640978 | T | A | 47 | K833* | nonsense | SNV | NM_024675.3 |
|  | SCLC | PIK3CA | chr3 | 178916930 | G | T | 29 | G106V | missense | SNV | NM_006218.2 |
|  | SCLC | PIK3CA | chr3 | 178952072 | A | G | 31 | M1043V | missense | SNV | NM_006218.2 |
|  | SCLC | RB1 | chr13 | 48934179 | C | G | 89 | L212V | missense | SNV | NM_000321.2 |
|  | SCLC | RB1 | chr13 | 48934197 | T | G | 88 | L218V | missense | SNV | NM_000321.2 |
|  | SCLC | TP53 | chr17 | 7577551 | C | A | 86 | G244C | missense | SNV | NM_000546.5 |
| **497** | TC | NOTCH2 | chr1 | 120468210 | C | T | 56 | R1410H | missense | SNV | NM_024408.3 |
|  | TC | PLCG1 | chr20 | 39792114 | G | A | 56 | D296N | missense | SNV | NM_002660.2 |
|  | TC | ROS1 | chr6 | 117631301 | C | T | 46 | R2126Q | missense | SNV | NM_002944.2 |
|  | TC | RPS6KA2 | chr6 | 166864686 | G | A | 49 | H379Y | missense | SNV | NM_001006932.1 |
| **502** | AC |  |  |  |  |  |  |  |  |  |  |
| **509** | LCNEC | CDC73 | chr1 | 193205386 | G | A | 55 | W439* | nonsense | SNV | NM_024529.4 |
|  | LCNEC | MLL | chr11 | 118390672 | G | C | 17 | R3774S | missense | SNV | NM_001197104.1 |
|  | LCNEC | RIN3 | chr14 | 93118129 | C | A | 10 | S245R | missense | SNV | NM_024832.3 |
|  | LCNEC | THSD7B | chr2 | 138434094 | C | A | 10 | A1551E | missense | SNV | XM_005263802.1 |
|  | LCNEC | NOTCH2 | chr1 | 120462024 | C | A | 39 | D1898Y | missense | SNV | NM_024408.3 |
|  | LCNEC | RB1 | chr13 | 49039339 | A | T | 76 |  | splice site alteration | SNV | NM_000321.2 |
|  | LCNEC | TP53 | chr17 | 7577529 | A | T | 74 | I251N | missense | SNV | NM_000546.5 |
|  | LCNEC | TRIM24 | chr7 | 138268730 | G | T | 61 | A977S | missense | SNV | NM_015905.2 |
|  | LCNEC | LRP1B | chr2 | 141079598 | C | A | 50 | A4192S | missense | SNV | NM_018557.2 |
| **549** | TC |  |  |  |  |  |  |  |  |  |  |
| **552** | TC |  |  |  |  |  |  |  |  |  |  |
| **563** | AC | EPHB4 | chr7 | 100410597 | G | C | 76 | C630W | missense | SNV | NM_004444.4 |
|  | AC | SETD2 | chr3 | 47164711 | C | T | 47 | R472H | missense | SNV | NM_014159.6 |
|  | AC | TRRAP | chr7 | 98559964 | G | A | 11 | C2241Y | missense | SNV | NM_001244580.1 |
| **593** | AC |  |  |  |  |  |  |  |  |  |  |
| **607** | AC |  |  |  |  |  |  |  |  |  |  |
| **608** | TC | LRP1B | chr2 | 141055451 | G | C | 53 | T4298S | missense | SNV | NM_018557.2 |
| **609** | TC | APC | chr5 | 112177860 | G | C | 53 | G2190A | missense | SNV | NM_001127510.2 |
|  | TC | FANCA | chr16 | 89836362 | G | A | 36 | S796F | missense | SNV | NM_000135.2 |
| **611** | TC | FOXP4 | chr6 | 41559047 | C | T | 49 | T508I | missense | SNV | NM_001012426.1 |
|  | TC | MTRR | chr5 | 7878160 | G | A | 48 | V196M | missense | SNV | NM_024010.2 |
|  | TC | PIK3C2B | chr1 | 204429727 | C | T | 42 | R458Q | missense | SNV | NM_002646.3 |
|  | TC | TRIP11 | chr14 | 92471645 | G | C | 54 | T892S | missense | SNV | NM_004239.3 |
|  | TC | UGT1A8 | chr2 | 234675726 | T | C | 51 | V301A | missense | SNV | NM_019076.4 |

**Note:** TC, typical carcinoid; AC, atypical carcinoid; LCNEC, large-cell neuroendocrine carcinoma; SCLC, small-cell lung carcinoma. SNV, single nucleotide variant; DEL, deletion; INS, insertion.
