# Supplementary material for: Lung neuroendocrine tumours: deep sequencing of the four World Health Organization histotypes reveals chromatin‐remodelling genes as major players and a prognostic role for TERT, RB1, MEN1 and KMT2D
Source: J Pathol. 2016 Dec 29;241(4):488–500. doi: 10.1002/path.4853 (PMC5324596; doi:10.1002/path.4853)
Supplement: Supplementary file 10 — Table S5A. Discovery screen, copy number alterations by whole exome sequencing: histotype‐specific distribution in 20 lung neuroendocrine tumours. Related to Figure 3. Table S5B. Discovery screen, copy number alterations by high coverage targeted sequencing of 418 genes: histotype‐specific distribution in 46 lung neuroendocrine tumours. Related to Supplementary Figure S1B. [file PATH-241-488-s005.zip › PATH_4853_TableS5A.docx]

**Supplementary Table S5A.** Discovery screen, copy number alterations by whole exome sequencing: histotype-specific distribution in 20 lung neuroendocrine tumours. Related to Figure 2.

|  |  |  | **TC** | **n=10** |  |  |  |  | **AC** | **n=4** |  |  |  | **LCNEC** | **n=3** |  |  |  |  |  | **SCLC** | **n=3** |  |  |
| --- | --- | --- | --- | --- | --- | --- | --- | --- | --- | --- | --- | --- | --- | --- | --- | --- | --- | --- | --- | --- | --- | --- | --- | --- |
| ***GENE*** | *Homoz. Deletion* | *[%]* | *LOH* | *[%]* | *GAIN* | *[%]* | *Homoz. Deletion* | *[%]* | *LOH* | *[%]* | *GAIN* | *[%]* | *Homoz. Deletion* | *[%]* | *LOH* | *[%]* | *GAIN* | *[%]* | *Homoz. Deletion* | *[%]* | *LOH* | *[%]* | *GAIN* | *[%]* |
| *ABCA3* |  |  |  |  |  |  |  |  |  |  |  |  |  |  | 1 | [33.3] | |  |  |  |  |  |  |  |
| *ACTL6B* |  |  |  |  |  |  |  |  |  |  |  |  |  |  | 1 | [33.3] | |  |  |  |  |  |  |  |
| *AMOTL1* |  |  |  |  |  |  |  |  | 1 | [25.0] | |  |  |  |  |  |  |  |  |  |  |  |  |  |
| *ARID1A* |  |  |  |  |  |  |  |  | 1 | [25.0] | |  |  |  |  |  |  |  |  |  |  |  | 1 | [33.3] |
| *ARID2* |  |  |  |  |  |  |  |  |  |  |  |  |  |  |  |  | 1 | [33.3] |  |  |  |  |  |  |
| *ARID4B* |  |  |  |  |  |  |  |  |  |  |  |  |  |  |  |  | 1 | [33.3] |  |  |  |  |  |  |
| *ARID5B* |  |  |  |  |  |  |  |  |  |  |  |  |  |  |  |  |  |  |  |  | 1 | [33.3] |  |  |
| *ATM* |  |  |  |  |  |  |  |  | 1 | [25.0] | |  |  |  |  |  |  |  |  |  |  |  |  |  |
| *ATR* |  |  |  |  | 1 | [10.0] |  |  |  |  |  |  |  |  |  |  | 1 | [33.3] |  |  |  |  | 1 | [33.3] |
| *BAI1* |  |  |  |  | 1 | [10.0] |  |  |  |  | 1 | [25.0] |  |  |  |  | 2 | [66.7] |  |  |  |  | 1 | [33.3] |
| *BCL2* |  |  |  |  |  |  |  |  |  |  |  |  |  |  |  |  | 1 | [33.3] |  |  |  |  | 1 | [33.3] |
| *BCL2L1* |  |  |  |  | 1 | [10.0] |  |  |  |  |  |  |  |  |  |  | 1 | [33.3] |  |  |  |  |  |  |
| *BCL7C* |  |  |  |  |  |  |  |  |  |  |  |  |  |  |  |  | 1 | [33.3] |  |  |  |  |  |  |
| *BCL9* |  |  |  |  | 1 | [10.0] |  |  |  |  |  |  |  |  |  |  | 1 | [33.3] |  |  |  |  |  |  |
| *BIRC2* |  |  |  |  |  |  |  |  | 1 | [25.0] | |  |  |  |  |  |  |  |  |  |  |  |  |  |
| *BRCA2* |  |  |  |  |  |  |  |  |  |  |  |  |  |  | 1 | [33.3] | |  |  |  | 1 | [33.3] |  |  |
| *BRD7* |  |  |  |  |  |  |  |  |  |  |  |  |  |  |  |  | 1 | [33.3] |  |  | 1 | [33.3] |  |  |
| *BRD9* |  |  |  |  | 1 | [10.0] |  |  |  |  |  |  |  |  |  |  | 2 | [66.7] |  |  |  |  | 2 | [66.7] |
| *CAMK2A* |  |  |  |  |  |  |  |  |  |  |  |  |  |  | 1 | [33.3] | |  |  |  |  |  |  |  |
| *CCNE1* |  |  |  |  |  |  |  |  |  |  |  |  |  |  | 1 | [33.3] | |  |  |  |  |  | 1 | [33.3] |
| *CCNE2* |  |  |  |  |  |  |  |  |  |  | 1 | [25.0] |  |  |  |  |  |  |  |  |  |  |  |  |
| *CCNF* |  |  |  |  |  |  |  |  |  |  |  |  |  |  | 1 | [33.3] | |  |  |  |  |  |  |  |
| *CDC42* |  |  |  |  |  |  |  |  |  |  |  |  |  |  |  |  |  |  |  |  |  |  | 1 | [33.3] |
| *CDKN1B* |  |  |  |  |  |  |  |  |  |  |  |  |  |  |  |  |  |  |  |  | 1 | [33.3] |  |  |
| *CDX1* |  |  |  |  |  |  |  |  |  |  |  |  |  |  | 1 | [33.3] | |  |  |  |  |  |  |  |
| *CDX2* |  |  |  |  |  |  |  |  |  |  |  |  |  |  | 1 | [33.3] | |  |  |  | 1 | [33.3] |  |  |
| *CHEK2* |  |  |  |  |  |  |  |  | 1 | [25.0] | |  |  |  |  |  |  |  |  |  |  |  |  |  |
| *CRB1* |  |  |  |  |  |  |  |  |  |  |  |  |  |  |  |  | 1 | [33.3] |  |  |  |  |  |  |
| *CSKN1A1L* |  |  |  |  |  |  |  |  |  |  |  |  |  |  |  |  |  |  |  |  | 1 | [33.3] |  |  |
| *CTNND2* |  |  |  |  | 1 | [10.0] |  |  |  |  |  |  |  |  |  |  | 2 | [66.7] |  |  |  |  | 2 | [66.7] |
| *DAAM1* |  |  |  |  | 1 | [10.0] |  |  |  |  |  |  |  |  |  |  |  |  |  |  |  |  |  |  |
| *DICER* |  |  |  |  | 1 | [10.0] |  |  |  |  |  |  |  |  |  |  | 1 | [33.3] |  |  |  |  |  |  |
| *DLG2* |  |  |  |  |  |  |  |  | 1 | [25.0] | |  |  |  |  |  |  |  |  |  |  |  |  |  |
| *DPF2* |  |  |  |  |  |  |  |  | 1 | [25.0] | |  |  |  |  |  |  |  |  |  |  |  |  |  |
| *DPF3* |  |  |  |  | 1 | [10.0] |  |  |  |  |  |  |  |  |  |  |  |  |  |  |  |  |  |  |
| *DVL3* |  |  |  |  |  |  |  |  | 1 | [25.0] | |  |  |  |  |  |  |  |  |  |  |  |  |  |
| *ECAD* |  |  |  |  |  |  |  |  |  |  |  |  |  |  |  |  |  |  |  |  | 1 | [33.3] |  |  |
| *EP300* |  |  |  |  |  |  |  |  |  |  |  |  |  |  | 1 | [33.3] | |  |  |  |  |  |  |  |
| *FANCG* |  |  |  |  |  |  |  |  | 1 | [25.0] | |  |  |  |  |  |  |  |  |  |  |  |  |  |
| *FGFR1* |  |  |  |  |  |  |  |  |  |  |  |  |  |  |  |  | 1 | [33.3] |  |  |  |  |  |  |
| *FHIT* |  |  |  |  |  |  |  |  |  |  |  |  |  |  |  |  |  |  |  |  | 1 | [33.3] |  |  |
| *FOXA2* |  |  |  |  |  |  |  |  |  |  |  |  |  |  |  |  | 1 | [33.3] |  |  |  |  |  |  |
| *FZD4* |  |  |  |  |  |  |  |  | 1 | [25.0] | |  |  |  |  |  |  |  |  |  |  |  |  |  |
| *GATA2* |  |  |  |  |  |  |  |  |  |  |  |  |  |  |  |  | 1 | [33.3] |  |  |  |  | 1 | [33.3] |
| *GSK3A* |  |  |  |  |  |  |  |  |  |  |  |  |  |  |  |  |  |  |  |  |  |  | 1 | [33.3] |
| *GSK3B* |  |  |  |  |  |  |  |  | 1 | [25.0] | |  |  |  |  |  |  |  |  |  |  |  |  |  |
| *GSTT1* | 2 | [20.0] | 1 | [10.0] | |  | 1 | [25.0] | 2 | [50.0] | |  | 1 | [33.3] |  |  |  |  |  |  |  |  |  |  |
| *HDAC6* |  |  |  |  |  |  |  |  |  |  |  |  |  |  |  |  |  |  |  |  |  |  | 1 | [33.3] |
| *HIPK2* |  |  |  |  |  |  |  |  |  |  |  |  |  |  |  |  | 1 | [33.3] |  |  |  |  |  |  |
| *HIRA* |  |  |  |  |  |  |  |  | 1 | [25.0] | |  |  |  |  |  |  |  |  |  | 1 | [33.3] |  |  |
| *HRAS* |  |  |  |  |  |  |  |  |  |  |  |  |  |  |  |  | 1 | [33.3] |  |  |  |  |  |  |
| *HSP90AA1* |  |  |  |  | 1 | [10.0] |  |  |  |  |  |  |  |  |  |  | 1 | [33.3] |  |  |  |  |  |  |
| *KAT6A* |  |  |  |  |  |  |  |  |  |  |  |  | 1 | [33.3] |  |  |  |  |  |  |  |  |  |  |
| *KDM2A* |  |  |  |  |  |  |  |  | 1 | [25.0] | |  |  |  |  |  |  |  |  |  |  |  |  |  |
| *KDM3A* |  |  |  |  |  |  |  |  | 1 | [25.0] | |  |  |  |  |  |  |  |  |  |  |  |  |  |
| *KDM4B* |  |  |  |  |  |  |  |  |  |  |  |  |  |  | 1 | [33.3] | |  |  |  |  |  |  |  |
| *KDM4D* |  |  |  |  |  |  |  |  | 1 | [25.0] | |  |  |  |  |  |  |  |  |  |  |  |  |  |
| *KDM4E* |  |  |  |  |  |  |  |  | 1 | [25.0] | |  |  |  |  |  |  |  |  |  |  |  |  |  |
| *KDM5C* |  |  |  |  |  |  |  |  |  |  |  |  |  |  |  |  |  |  |  |  |  |  | 1 | [33.3] |
| *KDM6A* |  |  |  |  |  |  |  |  |  |  |  |  |  |  |  |  |  |  |  |  |  |  | 1 | [33.3] |
| *KMT2D* |  |  |  |  |  |  |  |  |  |  |  |  |  |  | 1 | [33.3] | |  |  |  |  |  |  |  |
| *KMT2E* |  |  |  |  |  |  |  |  |  |  |  |  |  |  | 1 | [33.3] | |  |  |  |  |  |  |  |
| *KREMEN2* |  |  |  |  |  |  |  |  |  |  |  |  |  |  | 1 | [33.3] | |  |  |  |  |  |  |  |
| *LATS2* |  |  |  |  |  |  |  |  | 1 | [25.0] | |  |  |  | 1 | [33.3] | |  |  |  | 1 | [33.3] |  |  |
| *LGR4* |  |  |  |  |  |  |  |  |  |  |  |  |  |  |  |  | 1 | [33.3] |  |  |  |  |  |  |
| *LRP1B* |  |  |  |  |  |  |  |  | 1 | [25.0] | |  |  |  |  |  |  |  |  |  |  |  |  |  |
| *LRP5* |  |  |  |  |  |  |  |  | 1 | [25.0] | |  |  |  |  |  |  |  |  |  |  |  |  |  |
| *MAPK1* |  |  |  |  |  |  |  |  | 1 | [25.0] | |  |  |  |  |  |  |  |  |  |  |  |  |  |
| *MAPK9* |  |  |  |  |  |  |  |  |  |  |  |  |  |  | 1 | [33.3] | |  |  |  |  |  |  |  |
| *MARK1* |  |  |  |  |  |  |  |  |  |  |  |  |  |  |  |  | 1 | [33.3] |  |  |  |  |  |  |
| *MEN1* |  |  |  |  |  |  |  |  | 1 | [25.0] | |  |  |  |  |  |  |  |  |  |  |  |  |  |
| *MOB1A* |  |  |  |  |  |  |  |  | 1 | [25.0] | |  |  |  |  |  |  |  |  |  |  |  |  |  |
| *MSI2* |  |  |  |  |  |  |  |  |  |  | 1 | [25.0] |  |  |  |  | 1 | [33.3] |  |  |  |  |  |  |
| *MST1* |  |  |  |  |  |  |  |  | 1 | [25.0] | |  |  |  | 1 | [33.3] | |  |  |  | 2 | [66.7] |  |  |
| *MST2* |  |  |  |  | 1 | [10.0] |  |  |  |  | 1 | [25.0] |  |  |  |  | 1 | [33.3] |  |  |  |  | 1 | [33.3] |
| *MTOR* |  |  |  |  |  |  |  |  |  |  |  |  |  |  | 1 | [33.3] | 1 | [33.3] |  |  |  |  |  |  |
| *MYC* |  |  |  |  | 1 | [10.0] |  |  |  |  | 1 | [25.0] |  |  |  |  | 2 | [66.7] |  |  |  |  | 1 | [33.3] |
| *NCOA2* |  |  |  |  |  |  |  |  |  |  | 1 | [25.0] |  |  |  |  | 1 | [33.3] |  |  |  |  | 1 | [33.3] |
| *NDN* |  |  |  |  |  |  |  |  |  |  |  |  |  |  | 1 | [33.3] | |  |  |  | 1 | [33.3] |  |  |
| *NEIL3* |  |  |  |  |  |  |  |  | 1 | [25.0] | |  |  |  |  |  |  |  |  |  |  |  |  |  |
| *NEK2* |  |  |  |  |  |  |  |  |  |  |  |  |  |  |  |  | 1 | [33.3] |  |  |  |  |  |  |
| *NEK5* |  |  |  |  |  |  |  |  |  |  |  |  |  |  |  |  | 1 | [33.3] |  |  |  |  |  |  |
| *NF2* |  |  |  |  |  |  |  |  | 1 | [25.0] | |  |  |  |  |  |  |  |  |  |  |  |  |  |
| *PBRM1* |  |  |  |  |  |  |  |  | 1 | [25.0] | |  |  |  | 1 | [33.3] | |  |  |  | 1 | [33.3] |  |  |
| *PIK3R1* |  |  |  |  |  |  |  |  |  |  |  |  |  |  | 1 | [33.3] | |  |  |  |  |  |  |  |
| *PIK3R2* |  |  |  |  |  |  |  |  |  |  |  |  |  |  | 1 | [33.3] | |  |  |  |  |  |  |  |
| *PIP5K1B* |  |  |  |  |  |  |  |  | 1 | [25.0] | |  |  |  |  |  |  |  |  |  |  |  |  |  |
| *PLCB2* |  |  | 1 | [10.0] | |  |  |  |  |  |  |  |  |  |  |  |  |  |  |  |  |  |  |  |
| *PP2A* |  |  |  |  |  |  |  |  |  |  |  |  |  |  | 1 | [33.3] | |  |  |  |  |  |  |  |
| *PTPN14* |  |  |  |  |  |  |  |  |  |  |  |  |  |  |  |  | 1 | [33.3] |  |  |  |  |  |  |
| *RASSF3* |  |  |  |  |  |  |  |  | 1 | [25.0] | |  |  |  |  |  |  |  |  |  |  |  |  |  |
| *RASSF5* |  |  |  |  |  |  |  |  |  |  |  |  |  |  |  |  | 1 | [33.3] |  |  |  |  |  |  |
| *RB1* |  |  | 3 | [30.0] | |  |  |  | 2 | [50.0] | |  | 1 | [33.3] | 1 | [33.3] | |  |  |  | 2 | [66.7] |  |  |
| *RICTOR* |  |  |  |  | 1 | [10.0] |  |  |  |  |  |  |  |  |  |  | 2 | [66.7] |  |  |  |  | 2 | [66.7] |
| *RNF213* |  |  |  |  |  |  |  |  |  |  | 1 | [25.0] |  |  |  |  | 1 | [33.3] |  |  |  |  |  |  |
| *ROR2* |  |  |  |  |  |  |  |  | 1 | [25.0] | |  |  |  |  |  |  |  |  |  |  |  |  |  |
| *RPS6KB1* |  |  |  |  |  |  |  |  |  |  |  |  |  |  |  |  | 1 | [33.3] |  |  |  |  |  |  |
| *RRAGC* |  |  |  |  |  |  |  |  |  |  | 1 | [25.0] |  |  |  |  |  |  |  |  |  |  |  |  |
| *RYK* |  |  |  |  |  |  |  |  | 1 | [25.0] | |  |  |  |  |  |  |  |  |  |  |  |  |  |
| *SCAI* |  |  |  |  |  |  |  |  |  |  |  |  |  |  | 1 | [33.3] | |  |  |  |  |  |  |  |
| *SDHA* |  |  |  |  | 1 | [10.0] |  |  |  |  |  |  |  |  |  |  | 2 | [66.7] |  |  |  |  | 2 | [66.7] |
| *SETD1A* |  |  |  |  |  |  |  |  |  |  |  |  |  |  |  |  | 1 | [33.3] |  |  |  |  |  |  |
| *SETD9* |  |  |  |  |  |  |  |  |  |  |  |  |  |  | 1 | [33.3] | |  |  |  |  |  |  |  |
| *SMARCA1* |  |  |  |  |  |  |  |  |  |  |  |  |  |  |  |  |  |  |  |  |  |  | 1 | [33.3] |
| *SMARCA4* |  |  |  |  |  |  |  |  |  |  |  |  |  |  | 1 | [33.3] | |  |  |  |  |  |  |  |
| *SMARCC1* |  |  |  |  |  |  |  |  | 1 | [25.0] | |  |  |  | 1 | [33.3] | |  |  |  | 1 | [33.3] |  |  |
| *SMARCD2* |  |  |  |  |  |  |  |  |  |  |  |  |  |  |  |  | 1 | [33.3] |  |  |  |  |  |  |
| *SOX1* |  |  |  |  |  |  |  |  |  |  |  |  |  |  |  |  | 1 | [33.3] |  |  |  |  |  |  |
| *SOX2* |  |  |  |  | 1 | [10.0] |  |  |  |  |  |  |  |  |  |  | 1 | [33.3] |  |  |  |  | 1 | [33.3] |
| *SRC* |  |  |  |  |  |  |  |  |  |  |  |  |  |  |  |  | 2 | [66.7] |  |  |  |  |  |  |
| *STK11* |  |  |  |  |  |  |  |  |  |  |  |  |  |  | 1 | [33.3] | |  |  |  |  |  | 1 | [33.3] |
| *TAB1* |  |  |  |  |  |  |  |  |  |  |  |  |  |  | 1 | [33.3] | |  |  |  |  |  |  |  |
| *TAO1* |  |  |  |  |  |  |  |  |  |  | 1 | [25.0] |  |  |  |  |  |  |  |  |  |  |  |  |
| *TAZ* |  |  |  |  |  |  |  |  |  |  |  |  |  |  |  |  |  |  |  |  |  |  | 3 | [100.0] |
| *TEAD3* |  |  |  |  |  |  |  |  |  |  |  |  |  |  |  |  |  |  |  |  |  |  | 1 | [33.3] |
| *TEAD4* |  |  |  |  |  |  |  |  |  |  |  |  |  |  |  |  |  |  |  |  | 1 | [33.3] |  |  |
| *TERT* |  |  |  |  | 1 | [10.0] |  |  |  |  |  |  |  |  |  |  | 2 | [66.7] |  |  |  |  | 2 | [66.7] |
| *TNF* |  |  |  |  |  |  |  |  |  |  |  |  |  |  |  |  |  |  |  |  |  |  | 1 | [33.3] |
| *TP53* |  |  | 2 | [20.0] | |  |  |  | 1 | [25.0] | |  |  |  |  |  |  |  |  |  | 3 | [100.0] | |  |
| *TSC2* |  |  |  |  |  |  |  |  |  |  |  |  |  |  | 1 | [33.3] | |  |  |  |  |  |  |  |
| *UBR5* |  |  |  |  |  |  |  |  |  |  | 1 | [25.0] |  |  |  |  | 1 | [33.3] |  |  |  |  | 1 | [33.3] |
| *ULK3* |  |  |  |  |  |  |  |  |  |  |  |  |  |  |  |  |  |  |  |  | 1 | [33.3] |  |  |
| *VHL* |  |  |  |  |  |  |  |  |  |  |  |  |  |  | 1 | [33.3] | |  |  |  | 1 | [33.3] |  |  |
| *WNT3A* |  |  |  |  |  |  |  |  |  |  |  |  |  |  |  |  | 1 | [33.3] |  |  |  |  |  |  |
| *WTIP* |  |  |  |  |  |  |  |  |  |  |  |  |  |  |  |  |  |  |  |  |  |  | 1 | [33.3] |
| *YAP* |  |  |  |  |  |  |  |  | 1 | [25.0] | |  |  |  |  |  |  |  |  |  |  |  |  |  |
| *YES1* |  |  |  |  |  |  |  |  |  |  | 1 | [25.0] |  |  |  |  | 1 | [33.3] |  |  |  |  |  |  |
| *YWHAQ* |  |  |  |  |  |  |  |  | 1 | [25.0] | |  |  |  |  |  |  |  |  |  | 1 | [33.3] |  |  |
| *ZIC2* |  |  |  |  |  |  |  |  |  |  |  |  |  |  |  |  | 1 | [33.3] |  |  |  |  |  |  |
| *ZO1* |  |  |  |  |  |  |  |  |  |  |  |  |  |  |  |  |  |  |  |  | 1 | [33.3] |  |  |

**Note:** TC, typical carcinoid; AC, atypical carcinoid; LCNEC, large-cell neuroendocrine carcinoma; SCLC, small-cell lung carcinoma.
