# Supplementary material for: Lung neuroendocrine tumours: deep sequencing of the four World Health Organization histotypes reveals chromatin‐remodelling genes as major players and a prognostic role for TERT, RB1, MEN1 and KMT2D
Source: J Pathol. 2016 Dec 29;241(4):488–500. doi: 10.1002/path.4853 (PMC5324596; doi:10.1002/path.4853)
Supplement: Supplementary file 10 — Table S5A. Discovery screen, copy number alterations by whole exome sequencing: histotype‐specific distribution in 20 lung neuroendocrine tumours. Related to Figure 3. Table S5B. Discovery screen, copy number alterations by high coverage targeted sequencing of 418 genes: histotype‐specific distribution in 46 lung neuroendocrine tumours. Related to Supplementary Figure S1B. [file PATH-241-488-s005.zip › PATH_4853_TableS5B.docx]

**Supplementary Table S5B.** Discovery screen, copy number alterations by high coverage targeted sequencing of 418 genes: histotype-specific distribution in 46 lung neuroendocrine tumours. Related to Supplementary Figure S2B.

|  |  |  | **TC** | **n=23** | |  |  |  | **AC** | **n=14** | |  |  |  | **LCNEC** | **n=5** |  |  |  |  | **SCLC** | **n=4** |  |  |
| --- | --- | --- | --- | --- | --- | --- | --- | --- | --- | --- | --- | --- | --- | --- | --- | --- | --- | --- | --- | --- | --- | --- | --- | --- |
| ***GENE*** | *Homoz. Deletion* | [%] | LOH | [%] | GAIN | [%] | *Homoz. Deletion* | [%] | LOH | [%] | GAIN | [%] | *Homoz. Deletion* | [%] | LOH | [%] | GAIN | [%] | *Homoz. Deletion* | [%] | LOH | [%] | GAIN | [%] |
| *ARID1A* |  |  |  |  |  |  |  |  |  |  |  |  |  |  |  |  | 1 | [20.0] |  |  |  |  | 2 | [50.0] |
| *ATR* |  |  |  |  | 1 | [4.3] |  |  |  |  | 1 | [7.1] |  |  |  |  | 1 | [20.0] |  |  |  |  | 1 | [25.0] |
| *BAI1* |  |  |  |  | 1 | [4.3] |  |  |  |  | 1 | [7.1] |  |  |  |  | 2 | [40.0] |  |  |  |  | 1 | [25.0] |
| *BCL2* |  |  |  |  |  |  |  |  |  |  |  |  |  |  |  |  |  |  |  |  |  |  | 2 | [50.0] |
| *BRD9* |  |  |  |  | 2 | [8.7] |  |  |  |  | 2 | [14.3] |  |  |  |  | 2 | [40.0] |  |  |  |  | 3 | [75.0] |
| *CDH2* |  |  |  |  |  |  |  |  |  |  |  |  |  |  |  |  | 1 | [20.0] |  |  |  |  |  |  |
| *CDKN2A* |  |  |  |  |  |  |  |  |  |  |  |  |  |  | 1 | [20.0] | |  |  |  |  |  |  |  |
| *CHEK2* |  |  |  |  |  |  |  |  | 1 | [7.1] | |  |  |  | 1 | [20.0] | |  |  |  |  |  |  |  |
| *CTNND2* |  |  |  |  | 2 | [8.7] |  |  |  |  | 2 | [14.3] |  |  |  |  | 2 | [40.0] |  |  |  |  | 3 | [75.0] |
| *DAXX* |  |  |  |  |  |  |  |  |  |  |  |  |  |  |  |  | 1 | [20.0] |  |  |  |  | 2 | [50.0] |
| *DICER* |  |  |  |  | 1 | [4.3] |  |  |  |  |  |  |  |  |  |  | 1 | [20.0] |  |  |  |  | 1 | [25.0] |
| *FGFR1* |  |  |  |  |  |  |  |  |  |  |  |  |  |  |  |  | 1 | [20.0] |  |  |  |  | 2 | [50.0] |
| *FGFR3* |  |  |  |  | 1 | [4.3] |  |  |  |  |  |  |  |  |  |  | 1 | [20.0] |  |  |  |  |  |  |
| *HSP90AA1* |  |  |  |  | 1 | [4.3] |  |  |  |  |  |  |  |  |  |  |  |  |  |  |  |  | 1 | [25.0] |
| *IRS2* |  |  |  |  |  |  |  |  |  |  |  |  |  |  |  |  |  |  |  |  |  |  | 3 | [75.0] |
| *KAT6A* |  |  |  |  |  |  |  |  |  |  | 1 | [7.1] |  |  |  |  | 2 | [40.0] |  |  |  |  | 1 | [25.0] |
| *LATS2* |  |  |  |  |  |  |  |  | 1 | [7.1] | |  |  |  | 1 | [20.0] | |  |  |  | 1 | [25.0] |  |  |
| *MEN1* |  |  | 1 | [4.3] | |  |  |  |  |  |  |  |  |  | 1 | [20.0] | |  |  |  |  |  |  |  |
| *MST1* |  |  | 1 | [4.3] | |  |  |  | 1 | [7.1] | |  |  |  | 1 | [20.0] | |  |  |  | 3 | [75.0] |  |  |
| *MST2* |  |  |  |  | 2 | [8.7] |  |  |  |  | 2 | [14.3] |  |  |  |  | 1 | [20.0] |  |  |  |  | 1 | [25.0] |
| *MTOR* |  |  |  |  |  |  |  |  |  |  |  |  |  |  |  |  | 1 | [20.0] |  |  |  |  | 1 | [25.0] |
| *MYC* |  |  |  |  | 1 | [4.3] |  |  |  |  | 1 | [7.1] |  |  |  |  | 4 | [80.0] |  |  |  |  | 2 | [50.0] |
| *MYCL* |  |  |  |  |  |  |  |  |  |  |  |  |  |  |  |  | 2 | [40.0] |  |  |  |  | 3 | [75.0] |
| *NCOA2* |  |  |  |  |  |  |  |  |  |  | 1 | [7.1] |  |  |  |  | 2 | [40.0] |  |  |  |  | 1 | [25.0] |
| *NOTCH2* |  |  |  |  |  |  |  |  |  |  |  |  |  |  |  |  | 1 | [20.0] |  |  |  |  | 1 | [25.0] |
| *NOTCH4* |  |  |  |  |  |  |  |  |  |  |  |  |  |  |  |  |  |  |  |  |  |  | 2 | [50.0] |
| *PRKDC* |  |  |  |  |  |  |  |  |  |  | 1 | [7.1] |  |  |  |  |  |  |  |  |  |  |  |  |
| *RB1* |  |  | 3 | [13.0] | |  |  |  | 2 | [14.3] | |  | 1 | [20.0] | 2 | [40.0] |  |  |  |  | 2 | [50.0] |  |  |
| *RET* |  |  |  |  |  |  |  |  |  |  |  |  |  |  |  |  |  |  |  |  | 1 | [25.0] |  |  |
| *RICTOR* |  |  |  |  | 2 | [8.7] |  |  |  |  | 2 | [14.3] |  |  |  |  | 2 | [40.0] |  |  |  |  | 2 | [50.0] |
| *RNF213* |  |  |  |  |  |  |  |  |  |  | 1 | [7.1] |  |  |  |  | 2 | [40.0] |  |  |  |  |  |  |
| *SDHA* |  |  |  |  | 1 | [4.3] |  |  |  |  | 1 | [7.1] |  |  |  |  | 3 | [60.0] |  |  |  |  | 3 | [75.0] |
| *SOX2* |  |  |  |  | 1 | [4.3] |  |  |  |  |  |  |  |  |  |  | 1 | [20.0] |  |  |  |  | 1 | [25.0] |
| *SRC* |  |  |  |  |  |  |  |  |  |  |  |  |  |  |  |  | 3 | [60.0] |  |  |  |  | 1 | [25.0] |
| *TAZ* |  |  |  |  | 1 | [4.3] |  |  |  |  |  |  |  |  |  |  | 1 | [20.0] |  |  |  |  | 3 | [75.0] |
| *TERT* |  |  |  |  | 2 | [8.7] |  |  |  |  | 2 | [14.3] |  |  |  |  | 2 | [40.0] |  |  |  |  | 3 | [75.0] |
| *TP53* |  |  | 2 | [8.7] | |  |  |  | 1 | [7.1] | |  |  |  | 1 | [20.0] | |  |  |  | 3 | [75.0] |  |  |
| *TSHR* |  |  |  |  | 1 | [4.3] |  |  |  |  |  |  |  |  |  |  | 1 | [20.0] |  |  |  |  | 1 | [25.0] |
| *UBR5* |  |  |  |  |  |  |  |  |  |  | 1 | [7.1] |  |  |  |  | 2 | [40.0] |  |  |  |  | 2 | [50.0] |
| *WT1* |  |  |  |  |  |  |  |  |  |  |  |  |  |  | 1 | [20.0] | |  |  |  |  |  |  |  |

**Note:** TC, typical carcinoid; AC, atypical carcinoid; LCNEC, large-cell neuroendocrine carcinoma; SCLC, small-cell lung carcinoma.
