# Supplementary material for: Lung neuroendocrine tumours: deep sequencing of the four World Health Organization histotypes reveals chromatin‐remodelling genes as major players and a prognostic role for TERT, RB1, MEN1 and KMT2D
Source: J Pathol. 2016 Dec 29;241(4):488–500. doi: 10.1002/path.4853 (PMC5324596; doi:10.1002/path.4853)
Supplement: Supplementary file 11 — Table S6. Univariate analysis on 78 lung neuroendocrine tumours for selection of candidate molecular prognostic predictors to be included in multivariate survival analysis. Related to Supplementary Figure S3. [file PATH-241-488-s007.docx]

**Supplementary Table S6.** Univariate analysis on 78 lung neuroendocrine tumours for selection of candidate molecular prognostic predictors to be included in multivariate survival analysis.

| **Molecular Marker** | **Univariate Hazard Ratio** | **95% CI** | **p-value*** |
| --- | --- | --- | --- |
| *RB1*: Homozygous deletion | 1.55 | 0.77 – 3.14 |  |
| *RB1*: Mutation | 3.29 | 0.96 – 11.3 | **0.014^#^** |
| TERT: Gain | 1.56 | 0.80 – 3.02 | **0.16** |
| *TP53*: Mutation | 1.19 | 0.64 – 2.23 | 0.57 |
| *TP53*: Loss of Heterozygosity | 2.01 | 1.06 – 3.80 | **0.024** |
| *KMT2D*: Mutation | 0.73 | 0.35 – 1.54 | 0.56 |
| *MYC*: Gain | 1.24 | 0.56 – 2.75 | 0.34 |
| *RICTOR*: Gain | 1.27 | 0.65 – 2.47 | 0.55 |
| *PIK3CA*: Gain | 2.04 | 1.01 – 4.12 | **0.021** |

**Note:** cases comprised 28 atypical carcinoids, 22 large cell neuroendocrine carcinomas, and 28 small cell lung cancers. The cut-off *p*-value for inclusion in multivariate analysis was *p*<0.2. Variables selected for multivariate analysis were *RB1* homozygous deletion, *RB1* mutation, *TERT* gain, *TP53* loss of heterozygosity and *PIK3CA* gain. Only *RB1* mutation and *TERT* gain resulted significant predictors at multivariate analysis.

*Mantel-Cox log-rank test

^#^ Comparison of each of the two *RB1* alterations (homozygous deletion and mutation) against wild-type cases yields a single *p*-value, but hazard ratios are computed for each alteration against the control (wild-type *RB1*). See also Supplementary Figure S3.
